# Tunable Methacrylamides for Covalent Ligand Directed Release Chemistry

**DOI:** 10.1021/jacs.0c10644

**Published:** 2021-03-25

**Authors:** Rambabu N. Reddi, Efrat Resnick, Adi Rogel, Boddu Venkateswara Rao, Ronen Gabizon, Kim Goldenberg, Neta Gurwicz, Daniel Zaidman, Alexander Plotnikov, Haim Barr, Ziv Shulman, Nir London

**Affiliations:** † Department of Organic Chemistry, The Weizmann Institute of Science, Rehovot, 7610001, Israel; ‡ Department of Immunology, The Weizmann Institute of Science, Rehovot, 7610001, Israel; § Wohl Institute for Drug Discovery of the Nancy and Stephen Grand Israel National Center for Personalized Medicine, The Weizmann Institute of Science, Rehovot, 7610001, Israel

## Abstract

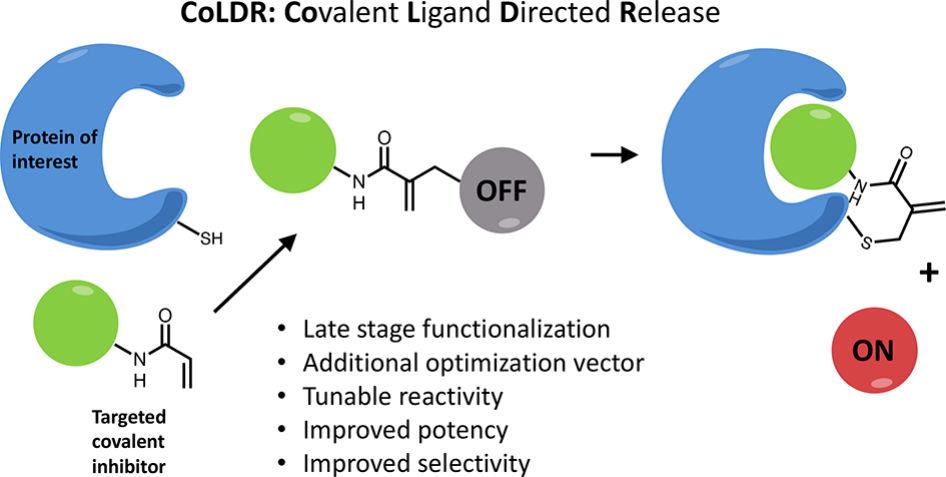

Targeted covalent inhibitors are an important class of drugs and chemical probes. However, relatively few electrophiles meet the criteria for successful covalent inhibitor design. Here we describe α-substituted methacrylamides as a new class of electrophiles suitable for targeted covalent inhibitors. While typically α-substitutions inactivate acrylamides, we show that hetero α-substituted methacrylamides have higher thiol reactivity and undergo a conjugated addition–elimination reaction ultimately releasing the substituent. Their reactivity toward thiols is tunable and correlates with the p*K*_a_/p*K*_b_ of the leaving group. In the context of the BTK inhibitor ibrutinib, these electrophiles showed lower intrinsic thiol reactivity than the unsubstituted ibrutinib acrylamide. This translated to comparable potency in protein labeling, in vitro kinase assays, and functional cellular assays, with improved selectivity. The conjugate addition–elimination reaction upon covalent binding to their target cysteine allows functionalizing α-substituted methacrylamides as turn-on probes. To demonstrate this, we prepared covalent ligand directed release (CoLDR) turn-on fluorescent probes for BTK, EGFR, and K-Ras^G12C^. We further demonstrate a BTK CoLDR chemiluminescent probe that enabled a high-throughput screen for BTK inhibitors. Altogether we show that α-substituted methacrylamides represent a new and versatile addition to the toolbox of targeted covalent inhibitor design.

## Introduction

**Figure 1:**
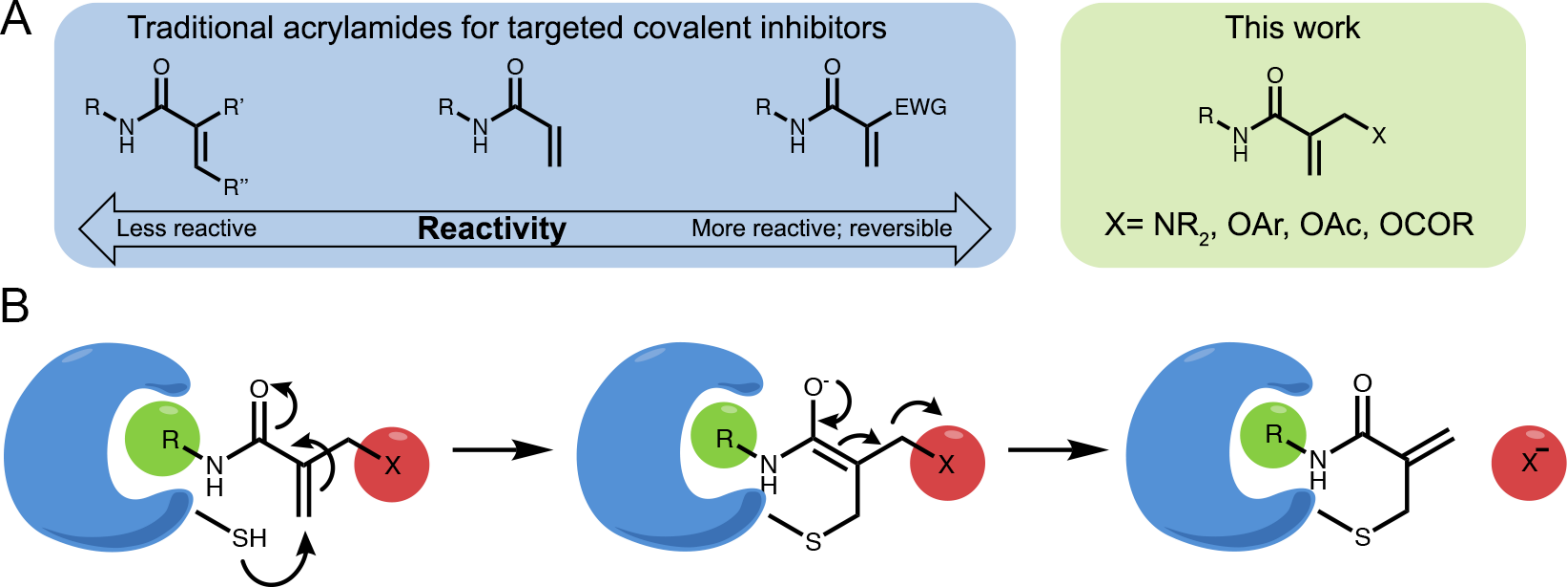
New type of acrylamide-based electrophiles for covalent inhibitors. (A) Various acrylamide substitutions can modify its properties, including both intrinsic reactivity and reversibility. (B) Schematic representation of the reaction of a target cysteine with a substituted α-methacrylamide through CoLDR (covalent ligand directed release) chemistry.

Acrylamides have been widely used as electrophiles for irreversible covalent inhibitors for many proteins bearing noncatalytic cysteines.^1−5^ For example, afatinib, ibrutinib, and AMG-510 are acrylamide-based inhibitors of EGFR, BTK, and K-Ras^G12C^, respectively (Figure S1). Such irreversible inhibitors have the advantages of nonequilibrium kinetics, full target occupancy, and flexibility to modify the structure for ADME (absorption, distribution, metabolism, and excretion) issues without sacrificing potency and selectivity.^6−8^ The efficiency of a covalent inhibitor depends upon initial reversible binding with the protein and the rate of subsequent covalent bond formation with the target nucleophile.^9,10^ The former depends on its reversible binding kinetics, whereas the latter depends on the reactivity of the electrophile^11,12^ and its accurate positioning. The intrinsic reactivity of acrylamides is strongly affected by the nature of their amine precursor,^12^ which is complicated to modify without affecting the reversible binding of the ligand. Furthermore, substitution at α- or β-positions usually reduces the reactivity of the acrylamides (Figure 1A). On the other hand, electron-withdrawing groups (EWGs) at the α-position increase the reactivity of the acrylamide while endowing reversibility to the formation of the covalent bond.^6,13−15^ The tunability of acrylamide reactivity is important for designing targeted covalent inhibitors. Recently, acrylamide analogues such as allenomides,^16^ propargylamides,^17^ alkynyl benzoxazines, and dihydroquinazolines^18^ have been reported as covalent reactive groups. However, they differ significantly from acrylamides in their structure and geometry, and therefore the reactive moiety cannot be simply switched without requiring the modification of the reversible binding scaffold. Here we describe an approach that enables the tuning of electrophile reactivity while maintaining a geometry similar to the original acrylamide and can also be used to modify targeted covalent inhibitors into turn-on fluorogenic, chemiluminescent, or otherwise functionalized probes.

We explored α-substituted methacrylamides as electrophilic warheads with varied reactivity, in the context of targeted covalent inhibitors. These compounds form a covalent bond with the nucleophile, which may be followed by the concomitant release of a leaving group that was present at the β-position (Figure 1). Similar chemistry has been reported in the context of bioconjugation^19,20^ of lysines and cysteines in proteins, but not in the context of targeted inhibitors. Here we show it can be used to modulate the reactivity of selective covalent inhibitors. Moreover, we used the release of the leaving group to functionalize covalent inhibitors and turn them into covalent ligand directed releasing (CoLDR) probes. We demonstrate this concept with turn-on fluorogenic probes against BTK, EGFR, and K-Ras^G12C^ and with a turn-on chemiluminescent CoLDR probe for BTK. The latter allowed us to perform a high-throughput screen that validated its use as an alternative to traditional kinase in vitro assays.

## Results

### Reactivity and Substitution Propensity of α-Methacrylamides Can Be Tuned by Different Substitutions

**Figure 2:**
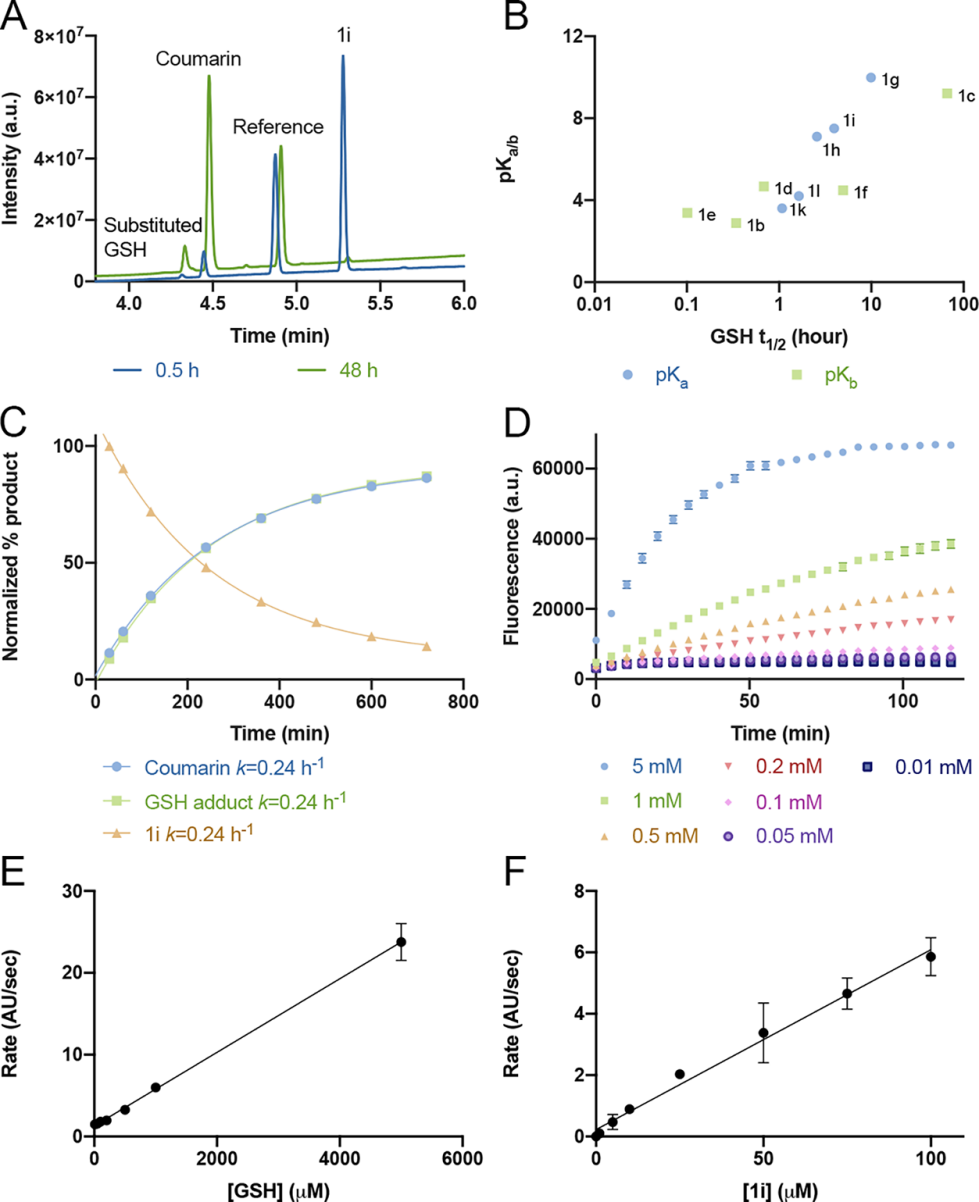
GSH reactivity correlates to the p*K*_**a/b**_ of the leaving group. (A) Example LC chromatogram showing monitoring of the reaction of **1i** (100 μM) with GSH (5 mM) at 30 min (blue) and 48 h (green). GSH adduct: retention time (RT) = 4.3 min, *m*/*z* = 480; coumarin: RT = 4.5 min; reference: RT = 4.8 min; **1i**: RT = 5.3 min; *m*/*z* = 332. UV absorption measured between 220 and 400 nm. (B) GSH *t*_1/2_ vs p*K*_a_ of the protonated leaving group (p*K*_b_ for amines). (C) Rates of formation in LC-MS (absorption 220–400 nm) of coumarin and GSH adduct and depletion of **1i** in a reaction between 100 μM **1i** and 5 mM GSH in PBS buffer, pH 8, 37 °C (*n* = 3). (D) Fluorescence intensity of **1i** (100 μM, *n* = 4) as a function of incubation time with different GSH concentrations (PBS buffer pH 8, 37 °C, Ex/Em = 385/435 nm). (E) Rates of the turn-on fluorescence reaction of **1i** (100 μM; *n* = 4) as a function of GSH concentration presented in D. (F) Rates of the turn-on fluorescence reaction of GSH (5 mM; PBS buffer pH 8, 37 °C, Ex/Em = 385/435 nm; *n* = 4) as a function of **1i** concentration. The linearity in E and F indicates that the first step of the reaction (thiol addition) is the rate-limiting step.

To investigate the reactivity and leaving ability of α-substituted methacrylamides, we synthesized a set of 12 model compounds of various α-substituted *N*-benzylmethacrylamides (**1b**–**1m**; Table 1) from the corresponding *N*-benzyl-2-(bromomethyl) acrylamide (Figure S2), as well as the unsubstituted acrylamide (**BnA**) and methacrylamide (**1a**). We reacted these electrophiles with reduced glutathione (GSH), as a model thiol, and monitored the reaction over time via liquid chromatography/mass spectrometry (LC/MS). As an example, analysis of the reaction of **1i** (which has coumarin as a substituent) after 0.5 and 48 h (Figure 2A) clearly indicates the formation of a substitution product, the formation of 7-hydroxycoumarin, and decrease of starting material. We quantified the depletion of starting material via LC/MS of all model compounds and assessed the reaction rates (5 mM GSH; 100 μM acrylamide; 37 °C; Table 1, Figure 2A, Figure S3).

**Table 1:**
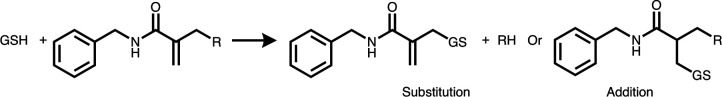

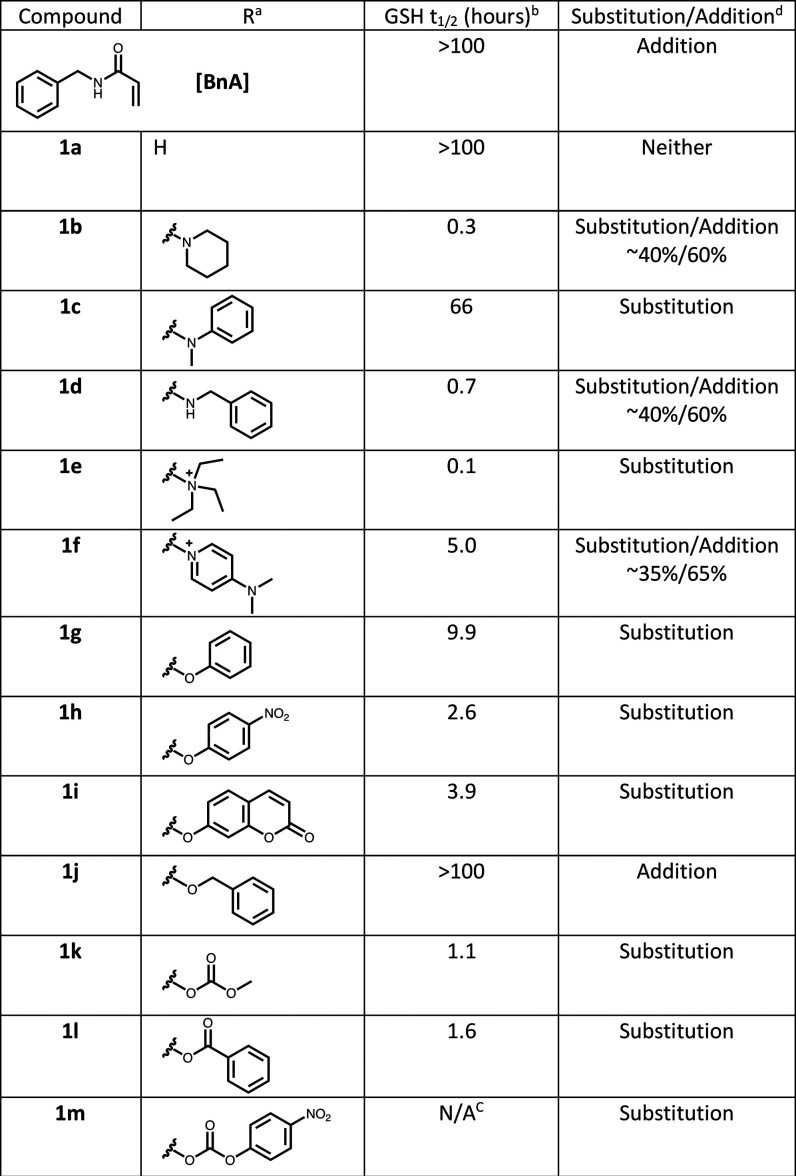
Various Heterosubstitutions of α-Methacrylamides Span 2.5 Orders of Magnitude in Reactivity toward GSH^d^

a Model-substituted α-methacrylamides.

b Reactivity toward GSH (*t*_1/2_) and reaction type was assessed via LC/MS (Figure 2A; Figure S3).

c This compound reacts through a two-step mechanism (see Figure S4).

d The reaction of substituted α-methacrylamides with GSH can result in either a substitution or addition product.

Of the 12 model compounds, eight preferably underwent the substitution reaction. These include the following leaving groups: phenoxy (**1g**, **1h**, and **1i**), benzoic acid (**1l**), carbonates (**1k** and **1m**), aniline (**1c**), and aliphatic quaternary amine (**1e**). The aliphatic amines and aromatic quaternary amine substituted methacrylamides (**1b**, **1d**, and **1f**) gave a mixture of substitution and addition products, whereas the aliphatic alcohol (**1j**) did not act as a leaving group and formed only a small amount of the addition product. Under the same conditions, we could not detect any reaction adduct of GSH with the methacrylamide **1a**, while for the unsubstituted acrylamide (**BnA**) the adduct formation was too low to quantify (Figure S3).

Concerning reaction rates, the effect of varying the leaving group on reactivity with GSH spans two and a half orders of magnitude. Three compounds with a basic amine substitution, **1b** (*t*_1/2_ = 0.3 h), **1d** (*t*_1/2_ = 0.7 h), and **1e** (*t*_1/2_ = 0.1 h), were among the most reactive, perhaps due to the basicity of the amines facilitating deprotonation of the cysteine thiol, which accelerated the reaction,^21^ or stabilization of the high-energy intermediate.^22^ In contrast, the dimethylaminopyridine (DMAP)-based methacryamide (**1f**) has a higher half-life (5 h) compared to other amines, which may be due to delocalization of the positive charge on the aromatic ring. Of the methacrylamides that showed complete substitution, the 4-nitrophenyl carbonate and methyl carbonate were the most reactive (**1k**; *t*_1/2_ = 1.1 h) followed by the benzoic acid (**1l**; *t*_1/2_ = 1.6 h). However, compound **1m** undergoes a two-step process (attack of GSH on the carbonyl of the carbonate group and then attack of another GSH on the acrylamide) for the formation of the GSH adduct (Figure S4). The compounds with an −OAr linkage (**1h**, **1i**, and **1g**) showed slightly lower reactivity, with *t*_1/2_ = 2.6 h, *t*_1/2_ = 3.9 h, and *t*_1/2_ = 9.9 h, respectively. The aniline **1c** was the least reactive, with *t*_1/2_ = 66 h. Finally, **1j**, which only underwent addition without elimination of its primary alcohol, was very slow to react with *t*_1/2_ ≥ 100 h. Counterintuitively, the substituted methacrylamides were more reactive than the unsubstituted acrylamide. We observed a clear correlation between the p*K*_a_ of the leaving group (p*K*_b_ in the case of amines)^23−25^ and the *t*_1/2_ of the model compounds’ reaction with GSH (Figure 2B). In these reactions with GSH, we found no decomposition of the compounds under the reaction conditions (within the duration of the assay; >12 h). Further, we have also checked the buffer stability of these compounds at pH 8, 37 °C. All compounds except **1k** and **1l** did not show significant decomposition after 5 days (Figure S5). Compounds **1k** and **1l**, which respectively contain carbonate and ester as leaving groups, undergo slow hydrolysis (<5%) after 6 and 24 h, respectively. Since the GSH half-life values for these compounds are very short (1.1 and 1.6 h), they do not degrade significantly in the course of the reaction.

Compound **1i** releases coumarin as the leaving group upon reaction with GSH, therefore enabling us to follow the reaction by a turn-on fluorescent readout. We took advantage of this property to follow the reaction kinetics. First, using LC/MS, we quantified the rates of coumarin formation, GSH adduct formation, and depletion of **1i** and show they are identical (Figure 2C), thus validating that we can follow the reaction rate by following coumarin fluorescence (Figure 2D). We show that the rate of coumarin release linearly increases with increasing GSH concentration (at a fixed concentration of 100 μM **1i**; pH 8; Figure 2D,E). Further, the rate of the reaction also linearly increases with concentrations of the **1i** at a fixed GSH concentration (5 mM; Figure 2F). This linearity indicates that the first step of the reaction (thiol addition) appears to be the rate-limiting step. Both the fluorescence and the reaction rate may be affected by pH. Fixing the concentration of the reactants (5 mM GSH; 100 μM **1i**) and increasing the pH showed a linear increase in the fluorescent signal as a function of pH (Figure S6).

### Proteomic Reactivity of Substituted α-Methacrylamides

**Figure 3:**
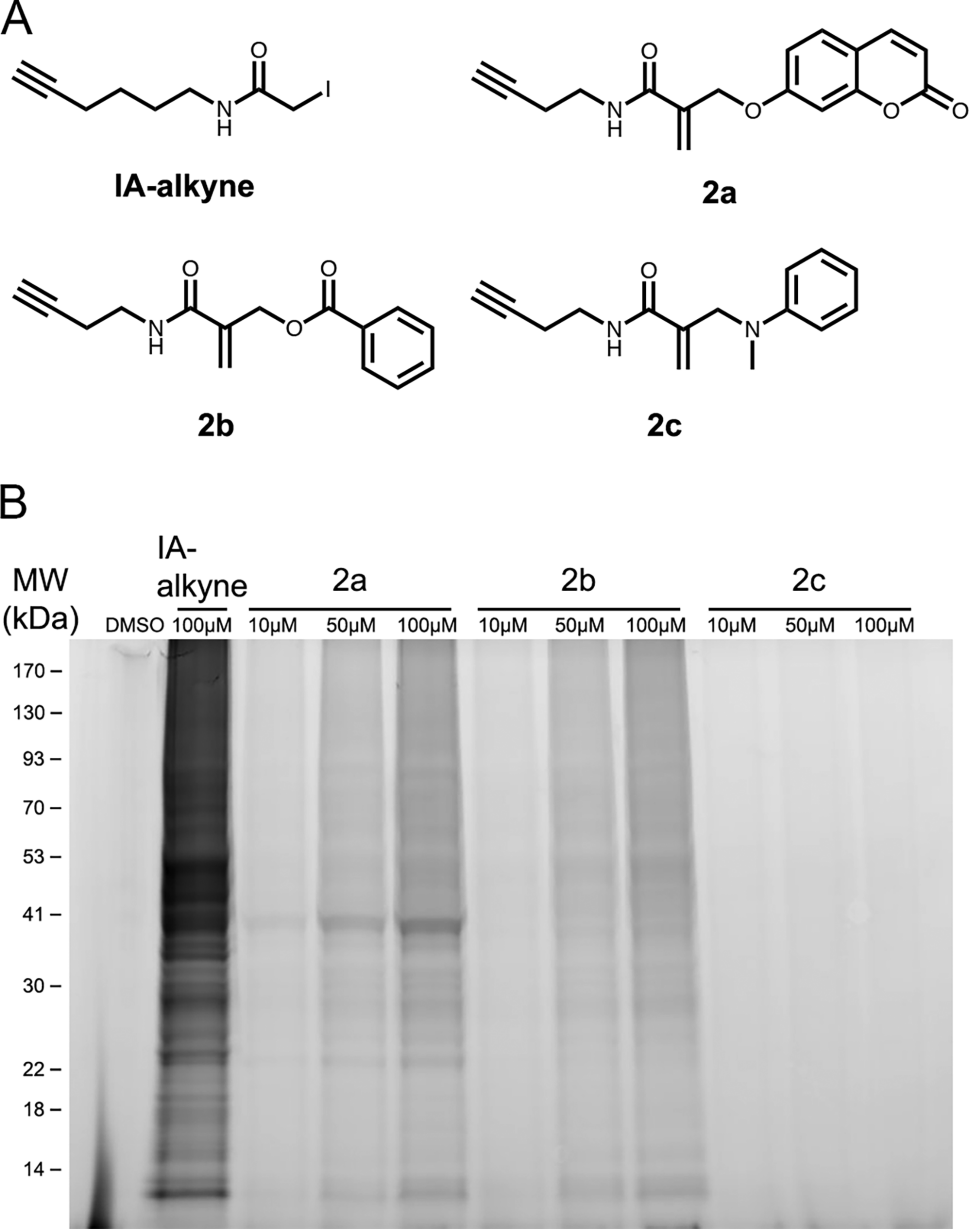
α-Methacrylamides show varied proteomic reactivity. (A) Chemical structures of model electrophilic alkyne probes. (B) In situ proteomic labeling with the alkyne probes. Mino cells were treated for 2 h with either DMSO, IA-alkyne, or **2a**–**2c**, then lysed, reacted with TAMRA-azide using Cu-AAC, and imaged via in-gel fluorescence (532 nm).

To assess the proteomic reactivity of this new electrophile, we have synthesized three model alkynes (Figure S7) bearing an α-methacrylamide substituted with either coumarin, benzoic acid, or *N*-methylaniline (**2a**–**2c**; Figure 3A). The coumarin-derivatized alkyne **2a** shows similar reactivity to **1i** in a GSH-triggered fluorescence assay (Figure S8). We treated Mino cells for 2 h with either DMSO, iodoacetamide alkyne (IA-alkyne), or **2a**–**2c**. We then lysed the cells, labeled the alkynes via copper-catalyzed “click chemistry” with TAMRA-azide, and imaged the adducts via in-gel fluorescence (Figure 3B). In cells, compound **2a** labeled more proteins compared to **2b** in spite of having lower reactivity in the GSH assay, possibly due to additional molecular recognition of the coumarin of **2a** with cellular proteins, or hydrolysis of the ester group of **2b** (most likely by cellular esterases, as spontaneous hydrolysis of **1l** was negligible at these time scales).^26^ Compound **2c**, however, seemed completely inactive in this experiment, which corresponds to the low reactivity of **1c** in the GSH experiment. All of the acrylamides were markedly less reactive than IA-alkyne.

### Late-Stage Functionalization of Irreversible Kinase Inhibitors

**Figure 4:**
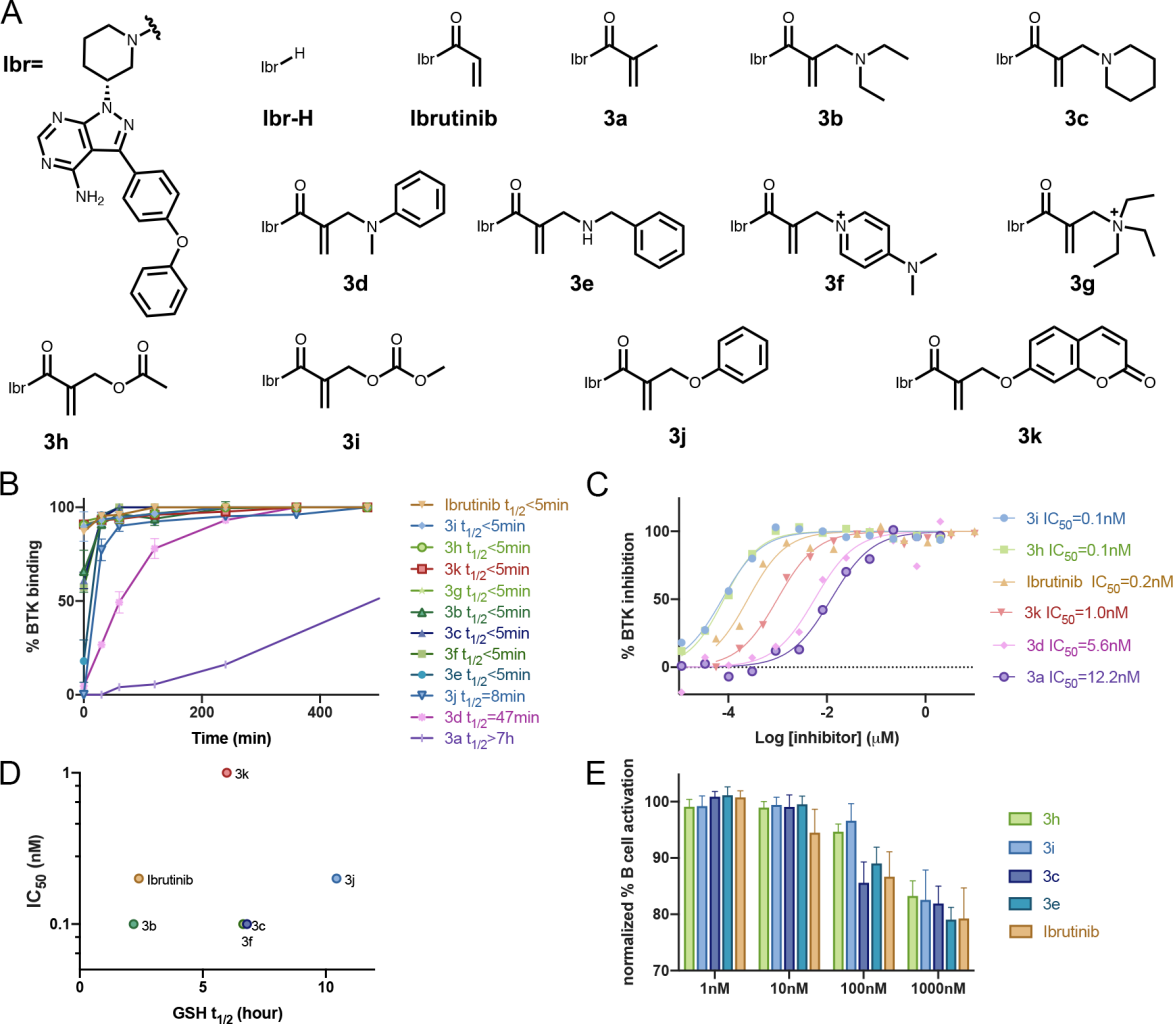
α-Substituted derivatives of ibrutinib as potential inhibitors. (A) Chemical structures of the ibrutinib derivatives. (B) Time course LC-MS binding assay (2 μM compound, ibrutinib or **3a**–**3k**, and 2 μM BTK at room temperature; *n* = 3; error bars indicate standard deviation). (C) In vitro kinase activity assay using wild-type BTK (0.6 nM BTK, 5 μM ATP) for selected analogues (see Figure S11 for all). (D) GSH half-life (*t*_1/2_) of ibrutinib derivatives does not correlate to measured IC_50_ values. Note that **3d** and **3e** are not presented since their GSH *t*_1/2_ > 100 h (E) Dose-dependent inhibition of B cell response after anti-IgM-induced activation and treatment with ibrutinib analogues for 24 h (*n* = 6; error bars indicate standard deviation).

To assess this chemistry in the context of irreversible covalent inhibitors, we selected ibrutinib as a model compound. Ibrutinib is an irreversible inhibitor of Bruton’s tyrosine kinase (BTK) and is FDA approved for several B cell oncogenic malignancies.^27^ Starting from the parent ibrutinib, we used the Morita–Baylis–Hillmann reaction to functionalize the acrylamide (Figure S9) and synthesized various ibrutinib-based methacrylamide derivatives with different leaving groups including phenols, acids, carbonates, amines, and quaternary ammonium salts (**3a**–**3k**; Figure 4A). All of these compounds exhibited covalent binding of the recombinant BTK kinase domain as assessed by intact protein LC/MS (Figure 4B; Figure S10; Table S1).

Similar to the model compounds, phenols, acids, carbonates, aniline, and quaternary aliphatic amine derivatives (**3k**, **3h**, **3i**, **3d**, and **3g**) showed 100% labeling through the substitution mechanism within 30 min. Basic amine derivatives such as **3b** and **3f** showed mixed binding with about 35% binding by substitution and 65% binding through Michael addition after 2 h of incubation (Figure S10). Finally, **3c** and **3e** label BTK exclusively through addition with no substitution product.

We next examined BTK labeling rates, which now may depend both on tuned intrinsic thiol reactivity and on potentially modified reversible protein recognition. Most compounds were comparable to ibrutinib, less than 2-fold higher or lower, regardless of the reaction mechanism observed (Figure 4B). Two of the compounds that labeled BTK the slowest, **3j** and **3d**, correspond to two of the slowest model compounds, **1g** and **1c**, respectively. Amine modifications that react solely through the addition mechanism, such as **3e** and **3c**, were among the fastest reacting (Figure 4B; Table S1).

To understand the potential of these compounds as inhibitors, we conducted in vitro kinase activity assays for all the ibrutinib derivatives against BTK. The IC_50_ values of these compounds (Figure 4C; Figure S11) closely mirrored the BTK kinetic labeling experiments, with some of the ester, carbonate, and basic amine substituted inhibitors such as **3c**, **3h**, **3i**, and **3e**, showing IC_50_ values in the <100 pM range, better than ibrutinib (IC_50_ = 288 pM). Most other compounds inhibited BTK, with IC_50_ < 1 nM (Figure 4C; Figure S11; Table S1).

Further, we have conducted a GSH-based reactivity assay for all the ibrutinib derivatives (Figure 4D; Figure S12). Surprisingly, despite being more potent in the in vitro kinase activity assay and LC/MS reactivity assay with BTK, these compounds showed lower reactivity than ibrutinib toward GSH. The low reactivity of these compounds with GSH may be due to the increased steric hindrance around the Michael acceptor, which also perhaps locked the acrylamide in a fixed conformation (Figure S13). The fixed geometry of the acrylamide may also help these compounds to react efficiently with BTK. We have found no significant decomposition of these compounds under the reaction conditions even after 72 h except for **3h** and **3i** (Figure S12).

To assess the compatibility of this chemistry with cellular conditions, we evaluated B cell receptor signaling inhibition in primary mouse B cells by ibrutinib as well as four of our new inhibitors. Mouse splenocytes were incubated (24 h; 37 °C) with the inhibitors at various concentrations and treated with anti-IgM. To examine the effect specifically on B cells, we gated on B220+ cells and assessed activation by flow cytometry detection of CD86 expression (Figure S14). All four inhibitors with substituted methacrylamides (**3c**, **3e**, **3h**, and **3i**) showed similar activity to ibrutinib, indicating both cellular engagement as well as stability to cellular conditions.

**Figure 5:**
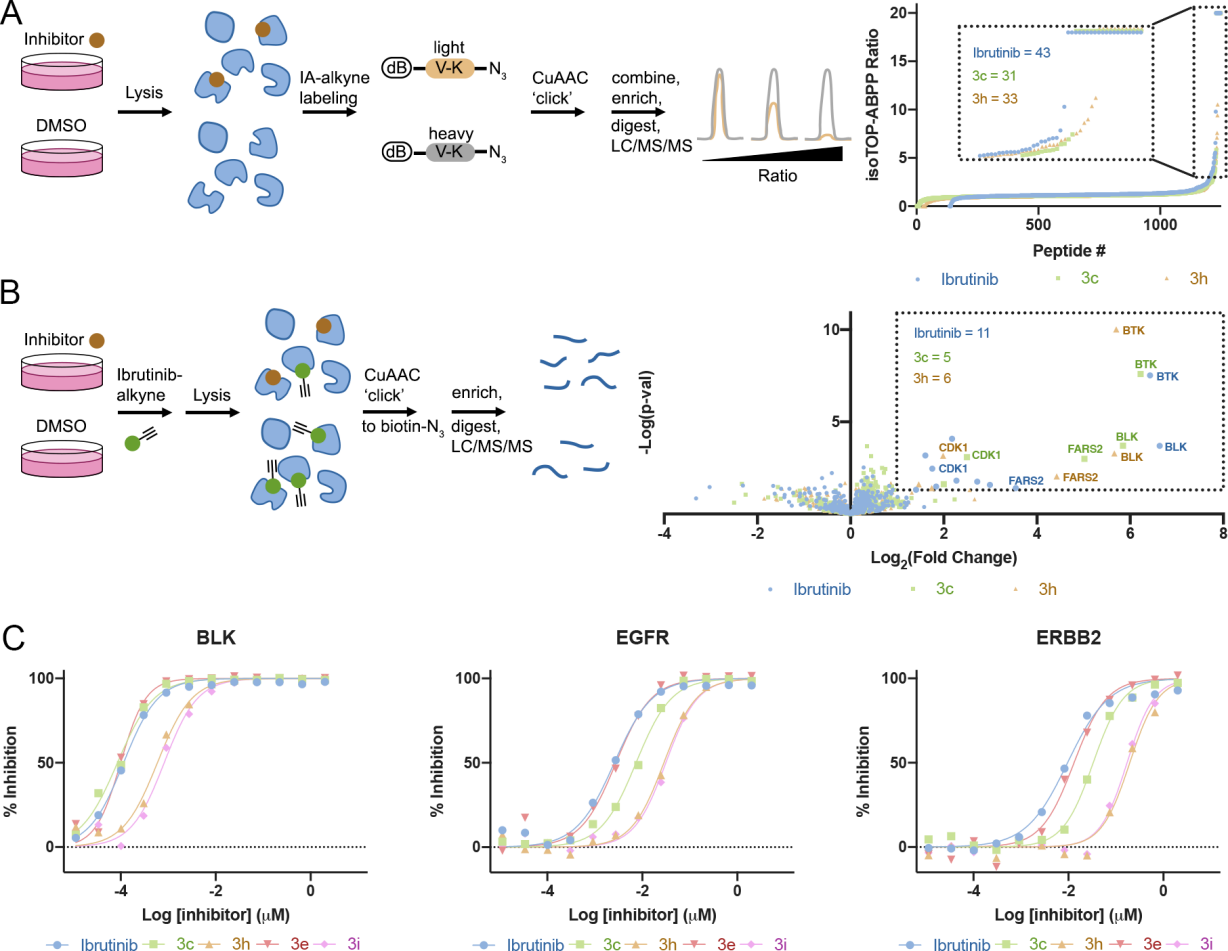
Selectivity of ibrutinib derivatives. (A) isoTOP ABPP using desthiobiotin–valine–azidolysine light or heavy peptides, schematic description, and result summary. Mino cells treated with 1 μM compound for 2 h (*n* = 4). Proteins in the box have a heavy to light (H/L) ratio ≥ 3. (B) Pull-down proteomics schematic description and result summary. Mino cells treated with 1 μM compound for 1 h and 10 μM ibrutinib-alkyne for an additional 1 h (*n* = 4). Proteins in the box show significant change (fold change > 2; *p* < 0.05). (C) In vitro kinase activity assays with selected kinases; see additional plots for BTK, BMX, and ITK in Figure S15.

To assess the selectivity of some of our ibrutinib derivatives, we performed a competitive isoTOP-ABPP^28^ experiment in Mino cells (Figure 5A, Data Set S1) using ibrutinib, **3c**, and **3h** (1 μM) as the tested molecules and IA-alkyne as a cysteine probe followed by copper-catalyzed cycloaddition (Cu-AAC) conjugation to a desthiobiotin-containing isotopically labeled peptide probe. This experiment indicated a slightly higher number of proteins significantly labeled by ibrutinib (*n* = 43 with heavy to light, H/L ratio >3) compared to **3c** and **3h** (*n* = 31 and 33, respectively), but was not able to detect BTK or other known off-targets of ibrutinib, likely due to their relatively low abundance.

In order to better identify relevant kinase targets, we performed a pull-down (Figure 5B, Data Set S2) experiment in which we first incubated Mino cells with either ibrutinib, **3c**, **3h** (1 μM), or DMSO control, followed by an incubation with an ibrutinib-alkyne probe (“probe 4”,^29^ 10 μM). This was followed by reaction with biotin-azide using Cu-AAC and pull-down of the labeled proteins. Here we were able to detect BTK as well as the ibrutinib off-targets BLK, TEC, and CDK1. All three compounds outcompeted the probe off BTK and the other off-targets. Here, too, ibrutinib had labeled a slightly higher number of significant targets (*n* = 11 at enrichment >2-fold and *p* < 0.05) compared to **3c** and **3h** (*n* = 5 and 6, respectively). We should note that BTK was the most significant target for all three inhibitors (Figure 5B).

To gain a more quantitative assessment of the selectivity of the compounds compared to ibrutinib, we selected five prominent kinase off-targets of ibrutinib and measured the in vitro IC_50_ of ibrutinib compared to **3c**, **3h**, **3e**, and **3i** (Figure 5C, Figure S15, Table 2). For all enzymes our compounds showed improved selectivity compared to ibrutinib, **3h** and **3i** being particularly selective, with almost 2 orders of magnitude higher selectivity for ERBB2 and ITK.

**Table 2:** IC_50_ (Rounded) and Selectivity of Ibrutinib Derivatives against Selected Ibrutinib Kinase Off-Targets

	BTK	BLK		BMX		EGFR		ERBB2		ITK	
compound	IC_50_ (nM)	IC_50_ (nM)	BLK/BTK	IC_50_ (nM)	BMX/BTK	IC_50_ (nM)	EGFR/BTK	IC_50_ (nM)	ERBB2/BTK	IC_50_ (nM)	ITK/BTK
ibrutinib	0.3	0.1	0	0.2	1	3	10	10	38	78	311
**3c**	0.1	0.1	1	0.3	5	7	105	33	472	43	607
**3h**	0.1	0.6	7	0.3	4	28	348	196	2400	295	3613
**3e**	0.1	0.1	1	0.4	5	3	36	13	169	30	383
**3i**	0.1	0.8	11	0.4	5	31	417	172	2292	232	3087

### Covalent Ligand Directed Release Chemistry for Functionalization of Irreversible Inhibitors

**Figure 6:**
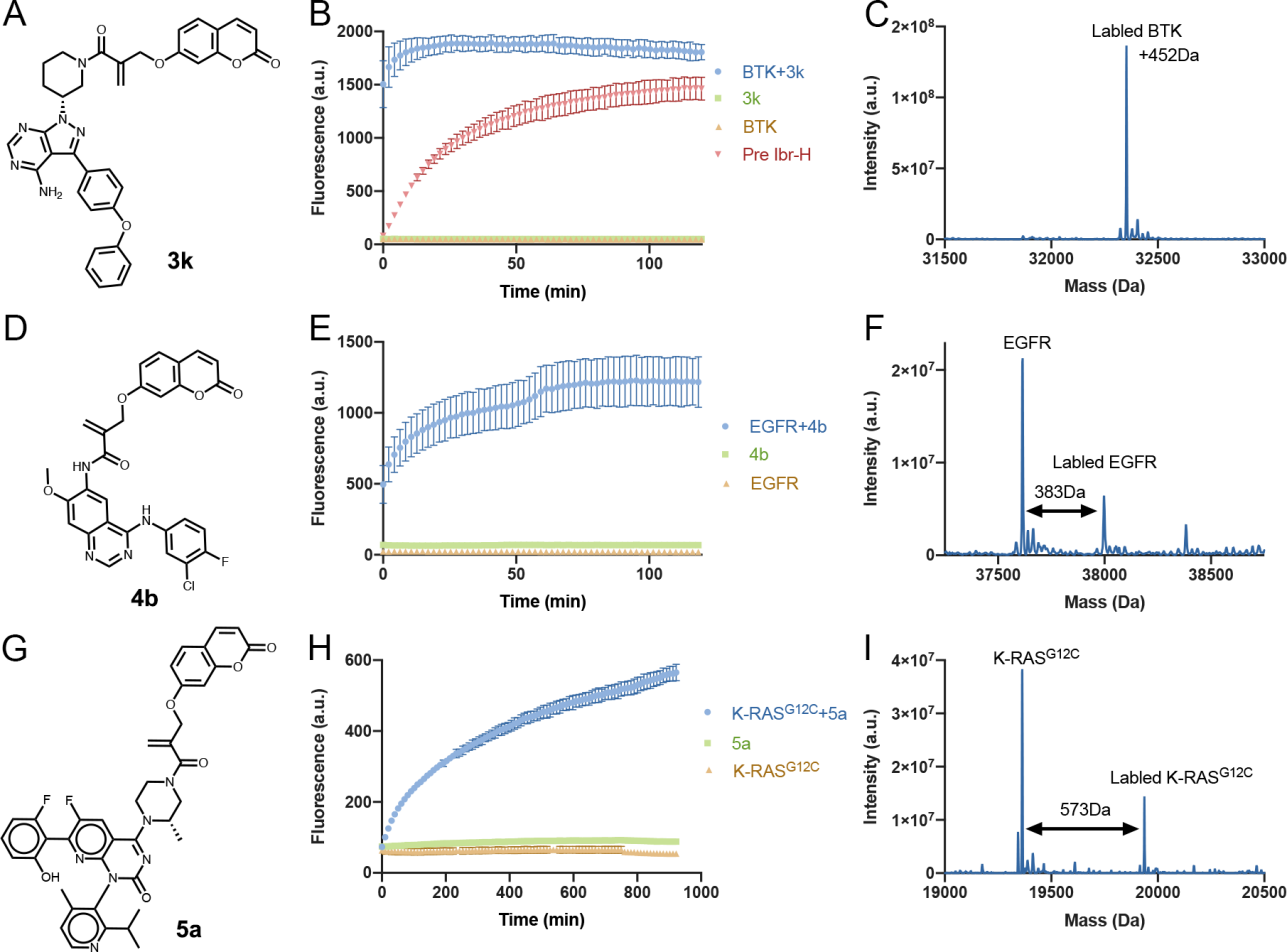
Turn-on fluorescent probes using CoLDR chemistry. (A, D, G) Structures of turn-on fluorescent probes for BTK, EGFR, and K-Ras^G12C^, respectively. (B, E, H) Time dependence of fluorescence intensity (representing the release of the coumarin moiety) measured at Ex/Em = 385/435 nm (*n* = 3). Green curves show that the compounds in and of themselves (2 μM) are not fluorescent. Orange curves show that the proteins themselves (2 μM) are also not fluorescent. Only upon mixing of probe and target (blue curves) do we see an increase in fluorescence. (C, F, I) Deconvoluted LC/MS spectra for BTK, EGFR, and K-Ras^G12C^ incubated with **3k**, **4b**, and **5a** at the end of each plate reader measurement. The adduct mass corresponds to a labeling event in which the coumarin moiety was released, validating the proposed mechanism. For BTK (D) we completed a reversible version of ibrutinib **Ibr-H** (2 μM; 0.5 h preincubation; Figure 4A) with **3k** (red curve). This considerably slowed the release of coumarin and the corresponding increase in fluorescence. All error bars indicate standard deviation.

The fact that a specific leaving group is released as a function of selective binding of a target protein can be used to functionalize irreversible inhibitors, for example as turn-on fluorescent probes. To assess the applicability and generality of this approach, we chose three therapeutic targets for which acrylamide inhibitors are available: BTK, EGFR, and K-RAS^G12C^ as model systems. Initially, we treated **3k** (Figure 6A) with BTK, measured the released coumarin fluorescence, and validated the labeling via LC/MS (Figure 6B,C). The fluorescence intensity of **3k** at 435 nm increased 30-fold upon the addition of BTK within a few seconds, reaching saturation within 10 min. To validate that the increase in fluorescence is due to the release of coumarin after binding to BTK, we repeated the experiment with BTK that was preincubated with a noncovalent analogue of ibrutinib (**Ibr-H**). In this experiment, the increase in fluorescence was significantly slower due to the gradual displacement of the **Ibr-H** by **3k**. We could also lower the rate of the reaction by using 20:1 equiv of protein:probe (Figure S16). We showed that the fluorescence increase is specific to the interaction with BTK, since incubating **3k** with BSA does not result in increased fluorescence (Figure S17A). The release can also be inhibited by preincubation of BTK with iodoacetamide alkyne (IAA; Figure S17B).

Similarly, we treated **4b** (Figure 6D–F, Figure S18; afatinib derivative functionalized with coumarin) and **5a** (Figure 6G–I, Figure S18; AMG-510 derivative functionalized with coumarin) with EGFR and K-RAS^G12C^, respectively, and measured the released coumarin fluorescence (Figure 6E,H). A significant increase in fluorescence intensity was observed in both cases with slower kinetics compared to BTK. LC/MS measurements at the end of the fluorescence measurements showed a shift in the molecular weight of the protein correlating to the size of the labeled compound without the released coumarin (Figure 6C,F,I). We should mention that in an EGFR kinase activity assay, while **4b** was slightly less potent than the unsubstituted **4a** (Figure S19), it still showed an impressive IC_50_ = 3.3 nM against EGFR.

**Figure 7:**
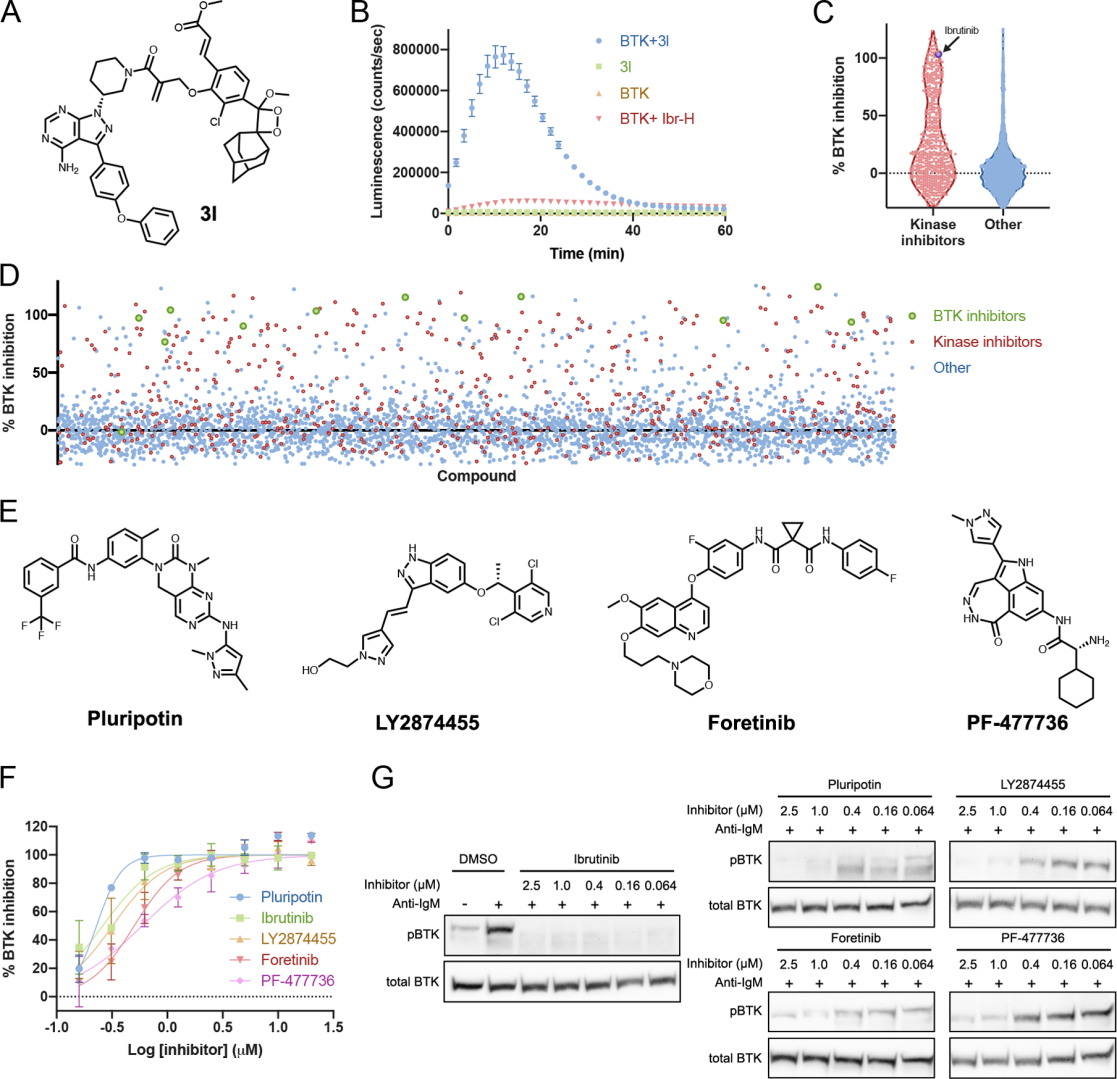
Chemiluminescent BTK probe allows high-throughput screening for BTK inhibitors. (A) Structure of the chemiluminescent probe **3l**. (B) Time dependence of the luminescence signal (representing the release of the chemiluminescent moiety, *n* = 3). The compound in and of itself (2 μM; green) is not luminescent. The protein itself (2 μM; orange) is also not luminescent. Only upon mixing of probe and target (blue) do we see an increase in luminescence. Preincubation of BTK with a reversible version of ibrutinib **Ibr-H** (2 μM; 0.5 h; red) inhibits luminescence (100 ms integration). (C) Schematic summary of %BTK binding inhibition in HTS using **3l** showing an enrichment of known kinase inhibitors in the library to bind BTK compared to nonkinase inhibitors. (D) Overall view of %BTK binding inhibition in the HTS. Known kinase inhibitors in red and known BTK inhibitors in green. (E) Structures of selected hits from HTS with **3l**. (F) Dose response (*n* = 2) of %BTK binding inhibition of selected hits from HTS with **3l**. (G) Inhibition of BTK phosphorylation in Mino cells with hit compounds. Cells were incubated for 1 h with inhibitors followed by 10 min activation with anti-IgM (full gels Figure S24). All error bars indicate standard deviation.

Recently, adamantylidene-dioxetane-based chemiluminescent turn-on probes for the sensing and imaging of enzymes, reactive oxygen species, and other analytes were reported.^30−34^ These probes, upon activation by analytes, release a phenolate-dioxetane intermediate, which subsequently decomposes with the emission of a photon in the visible spectrum (Figure S20). Indeed, these probes show high sensitivity and signal-to-background ratios. Accordingly, we have designed and synthesized an ibrutinib-derived chemiluminescent probe (**3l**) for activation by BTK (Figure 7A, Figure S21).

We measured the emission profile of probe **3l** (Figure 7A) in the absence and presence of BTK (2 μM; Figure 7B). The kinetic profile in the presence of BTK was typical of a chemiluminescent probe with an initial signal increase to a maximum within 20 min, followed by a slow decrease. BTK significantly enhanced the chemiluminescence of **3l** to 90-fold higher than the total photon counts emitted by probe **3l** in the absence of BTK. Preincubation of BTK with **Ibr-H** showed a significant decrease in the luminescence detected, indicating that this probe can be used to measure BTK binding.

To demonstrate the possible usage of such compounds, we conducted a high-throughput screen of 3725 bioactive compounds. The collection was assembled by merging of the commercially available and in-stock sets of anticancer, inflammation, and kinase inhibitors from Selleck Chemicals (2019; Data Set S3).

Due to the high fold-difference in the signal, we were able to run the screen at low volumes (10 μL) and low concentrations of both BTK and probe (0.75 and 1.5 μM, respectively). Overall, 488 compounds (13%) showed some inhibition of BTK, of which 216 (6%) inhibited at least 70% of the signal; 121 out of the 216 strong hit compounds are known kinase inhibitors, and 11 out of the 12 known BTK inhibitors in the library were identified as strong hits (Figure 7C,D; Data Set S3). We selected 25 hits that fully inhibited BTK and were not reported as BTK inhibitors, to be validated through dose response (Figure S22). Nine compounds that showed comparable inhibition to ibrutinib were further tested for cellular inhibition of BTK (Figure S23) at 500 nM for 1 h. Four of the compounds (Figure 7E,F), all kinase inhibitors, were further tested in a cellular dose response and showed promising cellular inhibition of BTK phosphorylation (Figure 7G). Pluripotin, a reported ERK1 and RasGAP inhibitor, fully inhibited pBTK at all tested concentrations (down to 64 nM; Figure 7G, Figure S24).

## Discussion

We have identified and characterized a new class of irreversible covalent warheads suitable for targeted covalent inhibitors. These offer several advantages for drug discovery and chemical biology including predictable attenuation of reactivity, late-stage installation with no additional modifications to the core scaffold, and importantly the ability to functionalize compounds as turn-on probes.

We showed that substituted methacrylamides in the context of model compounds span a wide window of thiol reactivity (as evaluated by *t*_1/2_ for their reaction with GSH; Table 1), which is predictable and depends on the p*K*_a/b_ of their respective leaving group (Figure 2B). We also showed that these types of electrophiles are suitable for chemoproteomic applications with various proteomic reactivities (Figure 3). As such, these will join a growing collection of cellular-compatible, cysteine-targeting electrophiles^5,8,16−18,35−37^ that may expand the scope of the targetable cysteinome.^38−42^ We note that since these methacrylamides leave an identical adduct on proteomically labeled cysteines, mixtures of such compounds may serve in the future as convenient probes for quantitative chemoproteomics with potentially increased coverage.

An important finding is that in the context of targeted covalent inhibitors their intrinsic thiol reactivity is significantly reduced (Figure 4C), and the vast majority of compounds showed lower GSH reactivity than the parent unsubstituted acrylamide. This may confer improved selectivity for such targeted covalent inhibitors, by lowering the number of possible off-targets as was previously shown for lower-reactivity covalent analogues of ibrutinib.^29^ Indeed, in two chemical proteomics experiments derivatives **3c** and **3h** of ibrutinib displayed slightly improved proteomic selectivity (Figure 5A,B). A more dramatic improvement in selectivity emerged in direct biochemical comparison of selectivity against prominent off-targets of ibrutinib in which all compounds, and **3h** and **3i** in particular, displayed up to 1.5 orders of magnitude higher selectivity (Table 2).

In this context, it is also interesting to note the cellular reactivity of the ester probes (e.g., **2b** and **3h**), which may also confer kinetic selectivity, as was previously shown for fumarate esters.^26^ We should note that in the context of ibrutinib there is also no longer a correlation between the p*K*_a/b_ of the leaving group and their GSH *t*_1/2_ (Figure S25), which may be related to steric effects and local conformation of the electrophile, as well as possibly to solubility or aggregation properties of the compounds.

Several of these compounds showed improved inhibition of BTK over ibrutinib, which is already a highly optimized BTK inhibitor (Figure 4B–E). A possible explanation for this is increased reactivity, but we have shown it is likely not the case. Another possible explanation is increased recognition mediated by the substitution at the α-position. A third possibility is that the substitution is locking the electrophile in a conformation more compatible with covalent bond formation (Figure S13). To assess the feasibility of these explanations, we modeled the prereacted compounds in complex with BTK in both their “cis” and “trans” conformations (Figure S26). We show that, indeed, all of the substitutions can fit in the binding site in both conformations, and some of them mediate additional contacts with the protein. In the future, cocrystal structures with C481S mutants may shed additional light on this aspect.

In any case, this finding suggests that this class of electrophiles can be useful for late-stage optimization of targeted covalent inhibitors, particularly since they can be installed directly on the acrylamide (Figure S9). Functional assays for B cell receptor signaling inhibition, in primary B cells, showed that they are active in a cellular context with comparable potency to ibrutinib (Figure 4E, Figure S27) and, as mentioned, improved selectivity. Future work is still necessary to understand the in vivo behavior of such electrophiles.

Perhaps the most exciting aspect of this new class of electrophiles is the ability to trigger the release of a chemical cargo, facilitated by a specific target cysteine. Most of the previously reported turn-on approaches are based on enzymatic functions by reductases, glycosidases, proteases, and lactamases.^43−47^ In the context of covalent labeling, acyloxymethyl ketones were used to generate FRET-based turn-on fluorescent probes for proteases,^48^ and quinone methide chemistry was also used for quenched activity-based probes.^49^ Recently, PET-based and cysteine reactive turn-on fluorescent probes have also been reported.^50−52^ Relatedly, Hamachi and colleagues reported several ligand-directed chemistries, in which a guiding ligand leaves the active site after the probe reacts with random nucleophilic residues (lysine, serine, and histidine) on the protein surface. These methods have been used to develop turn-on fluorescent probes,^44,53−56^ but require the ligand to retain high affinity and selectivity toward its target protein after modification with relatively large reactive groups.

Here we show that we can trigger turn-on release of a fluorophore by noncatalytic cysteines in a selective fashion (Figure 6; Figure S17). We have demonstrated the generality of this approach, coined CoLDR chemistry, by applying it to three various targeted covalent inhibitors, including against the challenging K-Ras^G12C^ oncogenic mutant. This approach is of course not limited to fluorophores. Since there is a wide scope of compatible leaving group functionalities (phenols, amines, carboxylic acids), many cargoes should be available for targeted release such as pro-drugs,^57−59^ chemotherapeutic agents,^60,61^ imaging agents,^62−64^ or self-immolative linkers^59^ potentially useful for both diagnostics as well as therapeutics. We should note that since we have shown that this type of chemistry works in cells, as discussed above, these turn-on probes should also be applicable to cells; however in the preliminary characterization of our coumarin-based probes the signal-to-noise ratio is very low, and their optimization is the subject of ongoing research.

We have demonstrated that CoLDR chemistry is also applicable for the generation of turn-on chemiluminescence (Figure 7) and has used this novel functional probe to facilitate a small high-throughput screen against BTK, resulting in the identification of known BTK inhibitors and nonselective kinase inhibitors. This assay is considerably simpler than the typical enzymatic-based assay, as it does not require any substrate or enzymatic reaction optimization. Moreover, it has the benefit of site-selective screening, since only inhibitors that will compete with the probe binding next to its target cysteine will reduce the signal. Using this screen, we were able to identify potent kinase inhibitors that are able to inhibit BTK in cells, although they were not previously annotated as such. A similar screen with the K-Ras^G12C^ probe for instance is expected to identify mainly switch-II pocket binders. This allows a convenient method to screen, for example, for allosteric binders if a suitable cysteine is present near the target pocket.^65−67^

In summary, we present a new class of substituted methacrylamides that will greatly expand the scope of targeted covalent inhibitors and will allow their functionalization for various applications.

## Methods

### LC/MS Measurements

LC/MS runs were performed on a Waters ACUITY UPLC class H instrument, in positive ion mode using electrospray ionization. UPLC separation for small molecules used a C18 column of 1.7 μm, 2.1 mm × 50 mm, for all the LC-MS-based assays. The GSH assay for compounds **1e**, **1f**, **1k**, and **3g** and buffer stability experiments for **1a**–**1l** were performed on LC-MS, C18 columns (1.7 μm, 2.1 mm × 100 mm). The column was held at 40 °C, and the autosampler at 10 °C. Mobile phase A was 0.1% formic acid in the water, and mobile phase B was 0.1% formic acid in acetonitrile. The run flow was 0.3 mL/min. The gradient used was 100% A for 2 min, increasing linearly to 90% B for 5 min, holding at 90% B for 1 min, changing to 0% B in 0.1 min, and holding at 0% for 1.9 min (for **1b**, the gradient started from 100% A and decreased linearly to 60% A for 2 min, 60–40% A for 2.0–6.0 min, 40–10% A in 0.5 min, and 10–100% A for 1.5 min; for **1k**, the gradient started from 100% A, decreased linearly to 5% A for 4 min, and run for 4 more minutes at 95% B). UPLC separation for protein used a C4 column (300 Å, 1.7 μm, 2.1 mm × 100 mm). The column was held at 40 °C, and the autosampler at 10 °C. Mobile solution A was 0.1% formic acid in the water, and mobile phase B was 0.1% formic acid in acetonitrile. The run flow was 0.4 mL/min with gradient 20% B for 2 min, increasing linearly to 60% B for 3 min, holding at 60% B for 1.5 min, changing to 0% B in 0.1 min, and holding at 0% for 1.4 min (for the kinetic labeling experiment, the gradient used was 90% A for 0.5 min, 90–40% A for 0.50–2.30 min, 40–10% A for 2.60–3.20 min, 10% A for 0.2 min, 10–90% A for another 0.2 min, and 90% A for 0.6 min). The mass data were collected on a Waters SQD2 detector with an *m*/*z* range of 2–3071.98 at a range of *m*/*z* of 800–1500 Da for BTK, 900–1800 Da for EFGR, and 750–1550 Da for K-RAS^G12C^.

### Plate Reader Fluorescence and Luminescence Measurements

Plate reader measurements were performed on a Tecan Spark Control 10 M fluorescent system using 384 black well plates with clear bottoms. Excitation was measured with a 360 ± 35 nm filter and emission with a 485 ± 20 nm filter. Luminescence measurements were performed using 384 white well plates, with integration for 100 and 1 ms settle times.

### GSH Reactivity Assay for Model Compounds

A 100 μM (5 μL of 20 mM stock) sample of the electrophile (**1a**–**1m**) was incubated with 5 mM GSH (50 μL of 100 mM stock) and 100 μM 4-nitrocyanobenzene (5 μL of 20 mM stock solution) as an internal standard in 100 mM potassium phosphate buffer of pH 8.0 (940 μL), respectively. All solvents were bubbled with argon. Reaction mixtures were kept at 37 °C with shaking. At various times 5 μL from the reaction mixture was injected into the LC/MS. The reaction was followed by the peak area of the electrophile normalized by the area of the 4-nitrocyanobenzene (i.e., by disappearance of the starting material). Natural logarithm of the results was fitted to linear regression, and *t*_1/2_ was calculated as *t*_1/2_ = ln 2/–slope.

### Buffer Stability Assay for Model Compounds

A 100 μM sample of the electrophile (**1a**–**1l**) was incubated with 100 μM 4-nitrocyano benzene as an internal standard in 100 mM potassium phosphate buffer of pH 8.0. All solvents were bubbled with argon. Reaction mixtures were kept at 37 °C with shaking. After 5 days, 5 μL from the reaction mixture was injected into the LC/MS. The reaction was followed by the peak area of the electrophile normalized by the area of the 4-nitrocyanobenzene.

### Effect of GSH/**1i** Concentration on the Rate of Reaction

GSH (5 mM) was added separately to 0, 1, 5, 10, 25, 50, 75, and 100 μM **1i**, or 100 μM **1i** was added separately to 10, 50, 100, 200, 500, 1000, and 5000 μM GSH in 100 mM potassium phosphate buffer pH 8.0 (titrated after the addition of GSH). Immediately fluorescence intensity measurements at 435 nm at 37 °C were acquired every 5 min for 10 h. The assay was performed in a 384-well plate using a Tecan Spark10M plate reader. Compounds were measured in quadruplicate. Control experiments were also conducted without GSH and **1i**. To obtain initial rates, for each sample, gradually increasing subsets of the fluorescence vs time data (starting from the first 4 data points) were fitted to linear fits. The longest subset that gave an *R*^2^ value above 0.99 was selected, and the rate was obtained for the sample from the slope. A quadruplicate was measured for each concentration. The average rate was calculated, and the 95% confidence interval was calculated based on Student’s *t* test.

### Effect of pH on the Reactivity of **1i** with GSH

A 100 μM concentration of **1i** was added to 5 mM GSH in 20 mM potassium phosphate buffer of various pH (6.2, 7.0, 7.5, and 8.0) and 20 mM Tris buffer of various pH (8.0, 8.5, and 9.0). Immediately fluorescence intensity measurements at 435 nm at 37 °C were acquired every 10 min for 1 h and every 1 h for 24 h. The assay was performed in a 384-well plate using a Tecan Spark10M plate reader. Compounds were measured in triplicate. Due to the variation in intrinsic coumarin fluorescence as a function of pH, reaction rates cannot be estimated directly from fluorescence values. Exploiting the fact that GSH is in excess, we fitted the fluorescence data to pseudo-first-order rate equations to obtain reaction rates.

### GSH Reactivity Assay for Ibrutinib Derivatives

A 100 μM concentration of the electrophile (**3a**–**3k**) was incubated with 100 μM 4-nitrocyano benzene as internal standard and 5 mM GSH in 100 mM potassium phosphate buffer pH 8.0 (titrated after the addition of GSH) and DMF at a ratio of 9:1, respectively. All solvents were bubbled with argon. Reaction mixtures were kept at 37 °C with shaking. After certain intervals of time as shown in the graph (1.5, 4, 8, 12, 24, 48, 72 h), 50 μL from the reaction mixture was immediately injected into the LC/MS. The reaction was followed by the peak area of the electrophile normalized by the area of the 4-nitrocyanobenzene. Natural logarithms of the results were fitted to linear regression, and *t*_1/2_ was calculated as *t*_1/2_ = ln 2/–slope.

### Kinetic Labeling Experiments of Ibrutinib Derivatives with BTK

The BTK kinase domain was expressed and purified as previously reported.^68^ Binding experiments were performed in 20 mM Tris pH 8.0, 50 mM NaCl, and 1 mM DTT. The BTK kinase domain was diluted to 2 μM in the buffer, and 2 μM ibrutinib derivatives were added by adding 1/100th volume from a 200 μM solution. The reaction mixtures, at room temperature for various times, were injected into the LC/MS. For data analysis, the raw spectra were deconvoluted using a 20 000:40 000 Da window and 1 Da resolution. The labeling percentage for a compound was determined as the labeling of a specific compound (alone or together with other compounds) divided by the overall detected protein species. Compounds were measured in triplicates.

### In-Gel Fluorescence Activity-Based Profiling

Mino cells were treated for 2 h with either 0.1% DMSO or the indicated concentrations of IA-alkyne, **2a**, **2b**, and **2c**. The cells were lysed with RIPA buffer (Sigma), and protein concentration was determined using the BCA protein assay (Thermo Fisher Scientific). Lysates were then diluted to 2 mg/mL in PBS and clicked to TAMRA-azide (click chemistry tools). Click reaction was performed using a final concentration of 40 μM TAMRA-azide, 3 mM CuSO_4_, 3 mM tris(3-hydroxypropyltriazolylmethyl)amine (THPTA, Sigma), and 3.7 mM sodium l-ascorbate (Sigma) in a final volume of 60 μL. The samples were incubated at 25 °C for 2 h. A 20 μL amount of 4× LDS sample buffer (NuPAGE, Thermo Fischer Scientific) was added followed by a 10 min incubation at 70 °C. The samples were then loaded on a 4–20% Bis-Tris gel (SurePAGE, GeneScript) and imaged using a Typhoon FLA 9500 scanner.

### In Vitro Kinase Activity (Carried out by Nanosyn, Santa Clara, CA, USA)

Kinase reactions are assembled in 384-well plates (Greiner) in a total volume of 20 μL. Test compounds were diluted in DMSO to a final concentration, while the final concentration of DMSO in all assays was kept at 1%. The compounds were incubated with the kinases for 2 h. A 1.2 nM concentration of BTK, 0.4 nM BLK, 2.4 nM BMX, 0.8 nM ITK in 100 mM HEPES, pH 7.5; 0.1% BSA, 0.01% Triton X-100, 1 mM DTT, 5 mM MgCl_2_, 0.75 nM EFGR in 100 mM HEPES, pH 7.5; 0.1% BSA, 0.01% Triton X-100, 1 mM DTT, 10 mM MnCl_2_, 2 nM ERBB2 in 100 mM HEPES, pH 7.5; and 0.1% BSA, 0.01% Triton X-100, 1 mM DTT, and 5 mM MnCl_2_ were used. The reaction was initiated by 2-fold dilution into a solution containing 5 μM ATP and 1 μM substrate in the kinase buffer

### B-Cell Response Experiment

Splenic cells from C57BL/6 mice were isolated by forcing spleen tissue through the mesh into PBS containing 2% fetal calf serum and 1 mM EDTA, and red blood cells were depleted by lysis buffer. Cells were cultured in 96-well U-bottom dishes (1 × 10^6^ cells/mL in RPMI 10% FCS) and incubated with BTK inhibitors in different concentrations (1, 10, 100, 1000 nM) for 24 h at 37 °C in 5% humidified CO_2_. Following a 24 h incubation, cells were stimulated with anti-IgM overnight (5 μg/mL, Sigma-Aldrich). Subsequently, cells were stained with anti-B220 (clone RA3-6B2, Biolegend) and anti-CD86 (clone GL-1, Biolegend) antibodies (anti-mouse CD86 Biolegend 105008 1:400, anti-mouse/human CD45R/B220 Biolegend 103212 1:400) for 30 min at 4 °C. Single-cell suspensions were analyzed by a flow cytometer (CytoFlex, Beckman Coulter).

### Fluorescence Intensity Measurements for CoLDR Turn-on Probes

A 2 μM concentration of BTK, EGFR, or K-RAS^G12C^ was added to 2 μM **3k**, **4b**, or **5a**, respectively. Control measurements were performed without either protein or compound and for BTK with preincubation with 2 μM noncovalent ibrutinib for 30 min. Each condition was done in triplicates in 20 mM Tris pH 8.0 and 50 mM NaCl for BTK and K-RAS^G12C^ and in 50 mM Tris pH 8.0 and 100 mM NaCl for EGFR at 32 °C. Fluorescent measurements were taken every 2 min for 2 h for BTK and EGFR and every 10 min for 15 h for K-RAS^G12C^. At the end of the measurements, samples were injected directly into the LC/MS for labeling quantification. K-Ras^G12C^ was expressed and purified as previously described,^69^ EGFR kinase domain was a generous gift from Prof. Michael Eck.

### HTS with the Chemiluminescent Probe

High-throughput screening was performed with the Selleck compound collection (the collection was composed by merging of the commercially available and in-stock sets of anticancer, inflammation, and kinase inhibitors from Selleck Chemicals (2019; Data Set S3) at 10 μM for the initial screen and 20–0.156 μM for the follow-up dose response in 1536-well white plates (Nunc, cat 264712), using GNF WDII washer/dispenser (Novartis, USA). BTK was preincubated with compounds for 15 min followed by the addition of a **3l** luminescence probe. The screen was performed with 0.75 μM BTK and 1.5 μM probe in 20 mM Tris pH 8.0, 50 mM NaCl 0.05% BSA, and 1 mM DTT final concentration. Luminescence was recorded after 30 min using a BMG PheraStar plate reader.

### Cell Culture and Reagents

Mino cells (acquired from the ATCC) were grown at 37 °C in a 5% CO_2_ humidified incubator and cultured in RPMI-1640 (Biological Industries), supplemented with 15% fetal bovine serum (Biological Industries) and 1% pen–strep solution (Biological Industries).

### BTK Activity in Cells

Mino cells were treated with 500 nM ibrutinib and compounds for 1 h. The cells were then incubated with 10 μg/mL anti-human IgM (Jackson ImmunoResearch, 109-006-129) for 10 min at 37 °C and harvested. Pellets were washed with ice-cold PBS and lysed using RIPA-buffer (Sigma, R0278). Lysates were clarified at 21000*g* for 15 min at 4 °C, and protein concentration was determined using the BCA protein assay (Thermo Fisher Scientific, 23225). A 50 μg amount was loaded on a 4–20% Bis-Tris gel (GeneScript SurePAGE, M00657), and proteins were separated by electrophoresis at 140 V and were transferred to a nitrocellulose membrane (Bio-Rad, 1704158) using a Trans-Blot Turbo system (Bio-Rad). The membrane was blocked using 5% BSA in TBS-T (w/v) for 1 h at room temperature, washed three times for 5 min with TBS-T, and incubated with the following primary antibodies: rabbit anti-phospho-BTK (#87141s, Cell Signaling, 1:500, overnight at 4 °C), mouse anti-BTK (#56044s, Cell Signaling, 1:1000, overnight at 4 °C), and mouse anti-b-actin (#3700, Cell Signaling, 1:1000, 1 h at room temperature). Membrane was washed three times for 5 min with TBS-T and incubated with the corresponding HRP-linked secondary antibody (mouse #7076/rabbit #7074, Cell Signaling) for 1 h at room temperature. The EZ-ECL kit (Biological Industries, 20-500-1000) was used to detect HRP activity. The membrane was stripped using Restore stripping buffer (Thermo Fisher Scientific, 21059) after each primary antibody before blotting with the next one.

### Preparation of IsoTOP DesThioTag Probe Peptide

The probe peptide was synthesized using standard solid phase synthesis on rink amide resin. The resin was swelled in dichloromethane for 30 min, washed with DMF, and deprotected using 20% piperidine/DMF (3 × 5 min). Two equivalents of Fmoc-(azidolysine)-OH was coupled in DMF using 2 equiv of HATU and 4 equiv of diisopropylethylamine for 2 h with tumbling, followed by three washes with DMF and Fmoc deprotection using the same method used above. At this step, 2 equiv of Fmoc-Val-OH (for the light probe) or Fmoc-Val-OH(13C5, 99%, 15N, 99%; Cambridge Isotope Laboratories) was coupled using the same method as before, followed by Fmoc deprotection. This was followed by coupling to 2 equiv of desthiobiotin (using the same method), followed by three washes with DMF, three washes with dichloromethane, and drying in a vacuum desiccator. The peptides were cleaved from the resin using 95% TFA, 2.5% TIPS, and 2.5% water for 3 h, followed by thorough evaporation of the cleavage mixture using nitrogen bubbling and purification by reverse-phase HPLC. The purified peptides were dissolved in DMSO to a concentration of 5 mM and used directly.

### IsoTOP ABPP Experiments

Mino cells were incubated for 2 h with 1 μM compound or DMSO. The cells were lysed and incubated with iodoacetamide-alkyne probe, followed by CuAAC reaction with an isotopically labeled desthiobiotin–valine–azidolysine peptide. Heavy and light probe samples were pooled, precipitated with methanol/chloroform, bound to streptavidin beads, and digested with trypsin, and the streptavidin-bound peptides were analyzed by LC-MSMS. Peptide identification and quantitation were performed using MaxQuant. A detailed description of the experimental procedure and data analysis methods is given in the Supporting Information.

### Pull-Down Experiments

Mino cells were incubated for 1 h with 1 μM compounds or DMSO, followed by an additional 1 h of incubation with 10 μM ibrutinib-alkyne probe.^29^ Following lysis, the samples were conjugated using CuAAC to biotin-azide. The proteins were precipitated by methanol/chloroform, bound to streptavidin beads, and the biotinylated proteins were eluted by boiling the samples in 5% SDS solution. The recovered proteins were reduced with DTT, alkylated with iodoacetamide, digested with trypsin, and analyzed using LC-MSMS. Identification and quantitation of recovered proteins were performed using MaxQuant. A detailed description of the experimental procedure and data analysis methods is given in the Supporting Information.

## Supporting Information Available

Additional information including synthesis schemes of all α-substituted methacrylamides, GSH consumption assay results, potential reaction mechanisms, buffer stabilities, time dependence pH effects and GSH concentration effects on reaction rates, intact protein MS of compound binding to BTK, in vitro kinase activity assays against BTK and off-targets, FACS analysis and primary B cell assay results, EGFR kinase activity assays, characterisation of BTK HTS hits including in vitro dose response, cellular single-point inhibition and cellular dose responses, molecular modeling of ibrutinib analogue binding, binding and reaction properties of ibrutinib analogues, and supplementary chemoproteomic methods (PDF)
Detailed synthetic protocols for preparation of compounds with high-resolution mass spectrometry and NMR analysis (PDF)
Chemoproteomics results and BTK HTS results (XLSX)

## References

[ref1] Zhao Z.; Bourne P. E. Progress with Covalent Small-Molecule Kinase Inhibitors. Drug Discovery Today 2018, 23 (3), 727–735. 10.1016/j.drudis.2018.01.035. 29337202

[ref2] Liu Q.; Sabnis Y.; Zhao Z.; Zhang T.; Buhrlage S. J.; Jones L. H.; Gray N. S. Developing Irreversible Inhibitors of the Protein Kinase Cysteinome. Chem. Biol. 2013, 20 (2), 146–159. 10.1016/j.chembiol.2012.12.006. 23438744 PMC3583020

[ref3] Baillie T. A. Targeted Covalent Inhibitors for Drug Design. Angew. Chem., Int. Ed. 2016, 55 (43), 13408–13421. 10.1002/anie.201601091. 27539547

[ref4] Singh J.; Petter R. C.; Baillie T. A.; Whitty A. The Resurgence of Covalent Drugs. Nat. Rev. Drug Discovery 2011, 10 (4), 307–317. 10.1038/nrd3410. 21455239

[ref5] Shannon D. A.; Weerapana E. Covalent Protein Modification: The Current Landscape of Residue-Specific Electrophiles. Curr. Opin. Chem. Biol. 2015, 24, 18–26. 10.1016/j.cbpa.2014.10.021. 25461720

[ref6] Jackson P. A.; Widen J. C.; Harki D. A.; Brummond K. M. Covalent Modifiers: A Chemical Perspective on the Reactivity of α,β-Unsaturated Carbonyls with Thiols via Hetero-Michael Addition Reactions. J. Med. Chem. 2017, 60 (3), 839–885. 10.1021/acs.jmedchem.6b00788. 27996267 PMC5308545

[ref7] Abdeldayem A.; Raouf Y. S.; Constantinescu S. N.; Moriggl R.; Gunning P. T. Advances in Covalent Kinase Inhibitors. Chem. Soc. Rev. 2020, 49 (9), 2617–2687. 10.1039/C9CS00720B. 32227030

[ref8] Gehringer M.; Laufer S. A. Emerging and Re-Emerging Warheads for Targeted Covalent Inhibitors: Applications in Medicinal Chemistry and Chemical Biology. J. Med. Chem. 2019, 62 (12), 5673–5724. 10.1021/acs.jmedchem.8b01153. 30565923

[ref9] Miyahisa I.; Sameshima T.; Hixon M. S. Rapid Determination of the Specificity Constant of Irreversible Inhibitors (kinact/KI) by Means of an Endpoint Competition Assay. Angew. Chem., Int. Ed. 2015, 54 (47), 14099–14102. 10.1002/anie.201505800. 26426864

[ref10] Strelow J. M. A Perspective on the Kinetics of Covalent and Irreversible Inhibition. J. Biomol. Screen. 2016. 10.1177/1087057116671509 27703080

[ref11] Martin J. S.; MacKenzie C. J.; Fletcher D.; Gilbert I. H. Characterising Covalent Warhead Reactivity. Bioorg. Med. Chem. 2019, 27 (10), 2066–2074. 10.1016/j.bmc.2019.04.002. 30975501 PMC6538824

[ref12] Flanagan M. E.; Abramite J. A.; Anderson D. P.; Aulabaugh A.; Dahal U. P.; Gilbert A. M.; Li C.; Montgomery J.; Oppenheimer S. R.; Ryder T.; Schuff B. P.; Uccello D. P.; Walker G. S.; Wu Y.; Brown M. F.; Chen J. M.; Hayward M. M.; Noe M. C.; Obach R. S.; Philippe L.; Shanmugasundaram V.; Shapiro M. J.; Starr J.; Stroh J.; Che Y. Chemical and Computational Methods for the Characterization of Covalent Reactive Groups for the Prospective Design of Irreversible Inhibitors. J. Med. Chem. 2014, 57 (23), 10072–10079. 10.1021/jm501412a. 25375838

[ref13] Serafimova I. M.; Pufall M. A.; Krishnan S.; Duda K.; Cohen M. S.; Maglathlin R. L.; McFarland J. M.; Miller R. M.; Frödin M.; Taunton J. Reversible Targeting of Noncatalytic Cysteines with Chemically Tuned Electrophiles. Nat. Chem. Biol. 2012, 8 (5), 471–476. 10.1038/nchembio.925. 22466421 PMC3657615

[ref14] Krishnan S.; Miller R. M.; Tian B.; Mullins R. D.; Jacobson M. P.; Taunton J. Design of Reversible, Cysteine-Targeted Michael Acceptors Guided by Kinetic and Computational Analysis. J. Am. Chem. Soc. 2014, 136 (36), 12624–12630. 10.1021/ja505194w. 25153195 PMC4160273

[ref15] Bradshaw J. M.; McFarland J. M.; Paavilainen V. O.; Bisconte A.; Tam D.; Phan V. T.; Romanov S.; Finkle D.; Shu J.; Patel V.; Ton T.; Li X.; Loughhead D. G.; Nunn P. A.; Karr D. E.; Gerritsen M. E.; Funk J. O.; Owens T. D.; Verner E.; Brameld K. A.; Hill R. J.; Goldstein D. M.; Taunton J. Prolonged and Tunable Residence Time Using Reversible Covalent Kinase Inhibitors. Nat. Chem. Biol. 2015, 11 (7), 525–531. 10.1038/nchembio.1817. 26006010 PMC4472506

[ref16] Chen D.; Guo D.; Yan Z.; Zhao Y. Allenamide as a Bioisostere of Acrylamide in the Design and Synthesis of Targeted Covalent Inhibitors. MedChemComm 2018, 9 (2), 244–253. 10.1039/C7MD00571G. 30108918 PMC6083791

[ref17] Wu J.; Zhang M.; Liu D. Acalabrutinib (ACP-196): A Selective Second-Generation BTK Inhibitor. J. Hematol. Oncol. 2016, 9, 21 10.1186/s13045-016-0250-9. 26957112 PMC4784459

[ref18] McAulay K.; Hoyt E. A.; Thomas M.; Schimpl M.; Bodnarchuk M. S.; Lewis H. J.; Barratt D.; Bhavsar D.; Robinson D. M.; Deery M. J.; Ogg D. J.; Bernardes G. J. L.; Ward R. A.; Waring M. J.; Kettle J. G. Alkynyl Benzoxazines and Dihydroquinazolines as Cysteine Targeting Covalent Warheads and Their Application in Identification of Selective Irreversible Kinase Inhibitors. J. Am. Chem. Soc. 2020, 142 (23), 10358–10372. 10.1021/jacs.9b13391. 32412754

[ref19] Matos M. J.; Oliveira B. L.; Martínez-Sáez N.; Guerreiro A.; Cal P. M. S. D.; Bertoldo J.; Maneiro M.; Perkins E.; Howard J.; Deery M. J.; Chalker J. M.; Corzana F.; Jiménez-Osés G.; Bernardes G. J. L. Chemo- and Regioselective Lysine Modification on Native Proteins. J. Am. Chem. Soc. 2018, 140 (11), 4004–4017. 10.1021/jacs.7b12874. 29473744 PMC5880509

[ref20] Zhuang J.; Zhao B.; Meng X.; Schiffman J. D.; Perry S. L.; Vachet R. W.; Thayumanavan S. A Programmable Chemical Switch Based on Triggerable Michael Acceptors. Chem. Sci. 2020, 11 (8), 2103–2111. 10.1039/C9SC05841A. PMC8150097 34123298

[ref21] Tsou H. R.; Mamuya N.; Johnson B. D.; Reich M. F.; Gruber B. C.; Ye F.; Nilakantan R.; Shen R.; Discafani C.; DeBlanc R.; Davis R.; Koehn F. E.; Greenberger L. M.; Wang Y. F.; Wissner A. 6-Substituted-4-(3-Bromophenylamino)quinazolines as Putative Irreversible Inhibitors of the Epidermal Growth Factor Receptor (EGFR) and Human Epidermal Growth Factor Receptor (HER-2) Tyrosine Kinases with Enhanced Antitumor Activity. J. Med. Chem. 2001, 44 (17), 2719–2734. 10.1021/jm0005555. 11495584

[ref22] Birkholz A.; Kopecky D. J.; Volak L. P.; Bartberger M. D.; Chen Y.; Tegley C.; Arvedson T. L.; McCarter J. D.; Fotsch C. H.; Cee V. J. Systematic Study of the Glutathione (GSH) Reactivity of N-Phenylacrylamides: 2. Effects of Acrylamide Substitution. J. Med. Chem. 2020, 63, 11602 10.1021/acs.jmedchem.0c00749. 32965113

[ref23] Fujino Y.; Yokoyama S. Surface Active Properties of Simple Cyclic and Heterocyclic Amines in Water. Chem. Pharm. Bull. 2000, 48 (2), 298–300. 10.1248/cpb.48.298. 10705526

[ref24] Zeng Y.; Chen X.; Zhao D.; Li H.; Zhang Y.; Xiao X. Estimation of pKa Values for Carboxylic Acids, Alcohols, Phenols and Amines Using Changes in the Relative Gibbs Free Energy. Fluid Phase Equilib. 2012, 313, 148–155. 10.1016/j.fluid.2011.09.022.

[ref25] Adamczyk K.; Prémont-Schwarz M.; Pines D.; Pines E.; Nibbering E. T. J. Real-Time Observation of Carbonic Acid Formation in Aqueous Solution. Science 2009, 326 (5960), 1690–1694. 10.1126/science.1180060. 19965381

[ref26] Zaro B. W.; Whitby L. R.; Lum K. M.; Cravatt B. F. Metabolically Labile Fumarate Esters Impart Kinetic Selectivity to Irreversible Inhibitors. J. Am. Chem. Soc. 2016, 138 (49), 15841–15844. 10.1021/jacs.6b10589. 27960302 PMC5273863

[ref27] Honigberg L. A.; Smith A. M.; Sirisawad M.; Verner E.; Loury D.; Chang B.; Li S.; Pan Z.; Thamm D. H.; Miller R. A.; Buggy J. J. The Bruton Tyrosine Kinase Inhibitor PCI-32765 Blocks B-Cell Activation and Is Efficacious in Models of Autoimmune Disease and B-Cell Malignancy. Proc. Natl. Acad. Sci. U. S. A. 2010, 107 (29), 13075–13080. 10.1073/pnas.1004594107. 20615965 PMC2919935

[ref28] Zanon P. R. A.; Lewald L.; Hacker S. M. Isotopically Labeled Desthiobiotin Azide (isoDTB) Tags Enable Global Profiling of the Bacterial Cysteinome. Angew. Chem., Int. Ed. 2020, 59 (7), 2829–2836. 10.1002/anie.201912075. PMC7027453 31782878

[ref29] Lanning B. R.; Whitby L. R.; Dix M. M.; Douhan J.; Gilbert A. M.; Hett E. C.; Johnson T. O.; Joslyn C.; Kath J. C.; Niessen S.; Roberts L. R.; Schnute M. E.; Wang C.; Hulce J. J.; Wei B.; Whiteley L. O.; Hayward M. M.; Cravatt B. F. A Road Map to Evaluate the Proteome-Wide Selectivity of Covalent Kinase Inhibitors. Nat. Chem. Biol. 2014, 10 (9), 760–767. 10.1038/nchembio.1582. 25038787 PMC4138289

[ref30] Son S.; Won M.; Green O.; Hananya N.; Sharma A.; Jeon Y.; Kwak J. H.; Sessler J. L.; Shabat D.; Kim J. S. Chemiluminescent Probe for the In Vitro and In Vivo Imaging of Cancers Over-Expressing NQO1. Angew. Chem., Int. Ed. 2019, 58 (6), 1739–1743. 10.1002/anie.201813032. 30561862

[ref31] Ye S.; Hananya N.; Green O.; Chen H.; Zhao A. Q.; Shen J.; Shabat D.; Yang D. A Highly Selective and Sensitive Chemiluminescent Probe for Real-Time Monitoring of Hydrogen Peroxide in Cells and Animals. Angew. Chem. 2020, 132, 23522 10.1002/ange.202011300. 32472602

[ref32] Hananya N.; Eldar Boock A.; Bauer C. R.; Satchi-Fainaro R.; Shabat D. Remarkable Enhancement of Chemiluminescent Signal by Dioxetane-Fluorophore Conjugates: Turn-ON Chemiluminescence Probes with Color Modulation for Sensing and Imaging. J. Am. Chem. Soc. 2016, 138 (40), 13438–13446. 10.1021/jacs.6b09173. 27652602

[ref33] Hananya N.; Shabat D. Recent Advances and Challenges in Luminescent Imaging: Bright Outlook for Chemiluminescence of Dioxetanes in Water. ACS Cent. Sci. 2019, 5 (6), 949–959. 10.1021/acscentsci.9b00372. 31263754 PMC6598152

[ref34] An R.; Wei S.; Huang Z.; Liu F.; Ye D. An Activatable Chemiluminescent Probe for Sensitive Detection of γ-Glutamyl Transpeptidase Activity in Vivo. Anal. Chem. 2019, 91 (21), 13639–13646. 10.1021/acs.analchem.9b02839. 31560193

[ref35] Mons E.; Jansen I. D. C.; Loboda J.; van Doodewaerd B. R.; Hermans J.; Verdoes M.; van Boeckel C. A. A.; van Veelen P. A.; Turk B.; Turk D.; Ovaa H. The Alkyne Moiety as a Latent Electrophile in Irreversible Covalent Small Molecule Inhibitors of Cathepsin K. J. Am. Chem. Soc. 2019, 141 (8), 3507–3514. 10.1021/jacs.8b11027. 30689386 PMC6396318

[ref36] Backus K. M. Applications of Reactive Cysteine Profiling. Curr. Top. Microbiol. Immunol. 2018, 420, 375–417. 10.1007/82_2018_120. 30105421

[ref37] Tokunaga K.; Sato M.; Kuwata K.; Miura C.; Fuchida H.; Matsunaga N.; Koyanagi S.; Ohdo S.; Shindo N.; Ojida A. Bicyclobutane Carboxylic Amide as a Cysteine-Directed Strained Electrophile for Selective Targeting of Proteins. J. Am. Chem. Soc. 2020, 142 (43), 18522–18531. 10.1021/jacs.0c07490. 33047956

[ref38] Weerapana E.; Wang C.; Simon G. M.; Richter F.; Khare S.; Dillon M. B. D.; Bachovchin D. A.; Mowen K.; Baker D.; Cravatt B. F. Quantitative Reactivity Profiling Predicts Functional Cysteines in Proteomes. Nature 2010, 468 (7325), 790–795. 10.1038/nature09472. 21085121 PMC3058684

[ref39] Parker C. G.; Galmozzi A.; Wang Y.; Correia B. E.; Sasaki K.; Joslyn C. M.; Kim A. S.; Cavallaro C. L.; Lawrence R. M.; Johnson S. R.; Narvaiza I.; Saez E.; Cravatt B. F. Ligand and Target Discovery by Fragment-Based Screening in Human Cells. Cell 2017, 168 (3), 527–541. 10.1016/j.cell.2016.12.029. 28111073 PMC5632530

[ref40] Bar-Peled L.; Kemper E. K.; Suciu R. M.; Vinogradova E. V.; Backus K. M.; Horning B. D.; Paul T. A.; Ichu T.-A.; Svensson R. U.; Olucha J.; Chang M. W.; Kok B. P.; Zhu Z.; Ihle N. T.; Dix M. M.; Jiang P.; Hayward M. M.; Saez E.; Shaw R. J.; Cravatt B. F. Chemical Proteomics Identifies Druggable Vulnerabilities in a Genetically Defined. Cancer. Cell 2017, 171 (3), 696–709. 10.1016/j.cell.2017.08.051 PMC5728659 28965760

[ref41] Vinogradova E. V.; Zhang X.; Remillard D.; Lazar D. C.; Suciu R. M.; Wang Y.; Bianco G.; Yamashita Y.; Crowley V. M.; Schafroth M. A.; Yokoyama M.; Konrad D. B.; Lum K. M.; Simon G. M.; Kemper E. K.; Lazear M. R.; Yin S.; Blewett M. M.; Dix M. M.; Nguyen N.; Shokhirev M. N.; Chin E. N.; Lairson L. L.; Melillo B.; Schreiber S. L.; Forli S.; Teijaro J. R.; Cravatt B. F. An Activity-Guided Map of Electrophile-Cysteine Interactions in Primary Human T Cells. Cell 2020, 182, 1009–1026. 10.1016/j.cell.2020.07.001. 32730809 PMC7775622

[ref42] Backus K. M.; Correia B. E.; Lum K. M.; Forli S.; Horning B. D.; González-Páez G. E.; Chatterjee S.; Lanning B. R.; Teijaro J. R.; Olson A. J.; Wolan D. W.; Cravatt B. F. Proteome-Wide Covalent Ligand Discovery in Native Biological Systems. Nature 2016, 534 (7608), 570–574. 10.1038/nature18002. 27309814 PMC4919207

[ref43] Zhu H.; Hamachi I. Fluorescence Imaging of Drug Target Proteins Using Chemical Probes. J. Pharm. Biomed. Anal. 2020. 10.1016/j.jpha.2020.05.013 PMC7591783 33133726

[ref44] Kojima H.; Fujita Y.; Takeuchi R.; Ikebe Y.; Ohashi N.; Yamamoto K.; Itoh T. Cyclization Reaction-Based Turn-on Probe for Covalent Labeling of Target Proteins. Cell Chem. Biol. 2020, 27 (3), 334–349. 10.1016/j.chembiol.2020.01.006. 31991094

[ref45] Chyan W.; Raines R. T. Enzyme-Activated Fluorogenic Probes for Live-Cell and in Vivo Imaging. ACS Chem. Biol. 2018, 13 (7), 1810–1823. 10.1021/acschembio.8b00371. 29924581 PMC6056172

[ref46] Yamaguchi T.; Asanuma M.; Nakanishi S.; Saito Y.; Okazaki M.; Dodo K.; Sodeoka M. Turn-ON Fluorescent Affinity Labeling Using a Small Bifunctional O-Nitrobenzoxadiazole Unit. Chem. Sci. 2014, 5, 1021–1029. 10.1039/C3SC52704B.

[ref47] Kobayashi H.; Ogawa M.; Alford R.; Choyke P. L.; Urano Y. New Strategies for Fluorescent Probe Design in Medical Diagnostic Imaging. Chem. Rev. 2010, 110 (5), 2620–2640. 10.1021/cr900263j. 20000749 PMC3241938

[ref48] Blum G.; Mullins S. R.; Keren K.; Fonovic M.; Jedeszko C.; Rice M. J.; Sloane B. F.; Bogyo M. Dynamic Imaging of Protease Activity with Fluorescently Quenched Activity-Based Probes. Nat. Chem. Biol. 2005, 1 (4), 203–209. 10.1038/nchembio728. 16408036

[ref49] Hu M.; Li L.; Wu H.; Su Y.; Yang P.-Y.; Uttamchandani M.; Xu Q.-H.; Yao S. Q. Multicolor, One- and Two-Photon Imaging of Enzymatic Activities in Live Cells with Fluorescently Quenched Activity-Based Probes (qABPs). J. Am. Chem. Soc. 2011, 133 (31), 12009–12020. 10.1021/ja200808y. 21732629

[ref50] Ma X.; Wu G.; Zhao Y.; Yuan Z.; Zhang Y.; Xia N.; Yang M.; Liu L. A Turn-On Fluorescent Probe for Sensitive Detection of Cysteine in a Fully Aqueous Environment and in Living Cells. J. Anal. Methods Chem. 2018, 2018, 1986468 10.1155/2018/1986468. 30647984 PMC6311829

[ref51] Xu K.; He L.; Yang Y.; Lin W. A PET-Based Turn-on Fluorescent Probe for Sensitive Detection of Thiols and H 2 S and Its Bioimaging Application in Living Cells, Tissues and Zebrafish. New J. Chem. 2019, 43 (7), 2865–2869. 10.1039/C8NJ04926B.

[ref52] Gao J.; Tao Y.; Wang N.; He J.; Zhang J.; Zhao W. BODIPY-Based Turn-on Fluorescent Probes for Cysteine and Homocysteine. Spectrochim. Acta, Part A 2018, 203, 77–84. 10.1016/j.saa.2018.05.114. 29860171

[ref53] Fujishima S.-H.; Yasui R.; Miki T.; Ojida A.; Hamachi I. Ligand-Directed Acyl Imidazole Chemistry for Labeling of Membrane-Bound Proteins on Live Cells. J. Am. Chem. Soc. 2012, 134 (9), 3961–3964. 10.1021/ja2108855. 22352855

[ref54] Tamura T.; Hamachi I. Chemistry for Covalent Modification of Endogenous/Native Proteins: From Test Tubes to Complex Biological Systems. J. Am. Chem. Soc. 2019, 141 (7), 2782–2799. 10.1021/jacs.8b11747. 30592612

[ref55] Tamura T.; Ueda T.; Goto T.; Tsukidate T.; Shapira Y.; Nishikawa Y.; Fujisawa A.; Hamachi I. Rapid Labelling and Covalent Inhibition of Intracellular Native Proteins Using Ligand-Directed N-Acyl-N-Alkyl Sulfonamide. Nat. Commun. 2018, 9 (1), 1870 10.1038/s41467-018-04343-0. 29760386 PMC5951806

[ref56] Tamura T.; Song Z.; Amaike K.; Lee S.; Yin S.; Kiyonaka S.; Hamachi I. Affinity-Guided Oxime Chemistry for Selective Protein Acylation in Live Tissue Systems. J. Am. Chem. Soc. 2017, 139 (40), 14181–14191. 10.1021/jacs.7b07339. 28915034

[ref57] Rautio J.; Meanwell N. A.; Di L.; Hageman M. J. The Expanding Role of Prodrugs in Contemporary Drug Design and Development. Nat. Rev. Drug Discovery 2018, 17 (8), 559–587. 10.1038/nrd.2018.46. 29700501

[ref58] Najjar A.; Najjar A.; Karaman R. Newly Developed Prodrugs and Prodrugs in Development; an Insight of the Recent Years. Molecules 2020, 25 (4), 884 10.3390/molecules25040884. PMC7070911 32079289

[ref59] Gnaim S.; Shabat D. Activity-Based Optical Sensing Enabled by Self-Immolative Scaffolds: Monitoring of Release Events by Fluorescence or Chemiluminescence Output. Acc. Chem. Res. 2019, 52 (10), 2806–2817. 10.1021/acs.accounts.9b00338. 31483607

[ref60] Giang I.; Boland E. L.; Poon G. M. K. Prodrug Applications for Targeted Cancer Therapy. AAPS J. 2014, 16 (5), 899–913. 10.1208/s12248-014-9638-z. 25004822 PMC4147050

[ref61] Zhang X.; Li X.; You Q.; Zhang X. Prodrug Strategy for Cancer Cell-Specific Targeting: A Recent Overview. Eur. J. Med. Chem. 2017, 139, 542–563. 10.1016/j.ejmech.2017.08.010. 28837920

[ref62] Kang J. J.; Toma I.; Sipos A.; Peti-Peterdi J. From in Vitro to in Vivo: Imaging from the Single Cell to the Whole Organism. Curr. Protoc. Cytom. 2008, 44, 12.12. 10.1002/0471142956.cy1212s44 18770644

[ref63] Dias G. G.; King A.; de Moliner F.; Vendrell M.; da Silva Júnior E. N. Quinone-Based Fluorophores for Imaging Biological Processes. Chem. Soc. Rev. 2018, 47 (1), 12–27. 10.1039/C7CS00553A. 29099127

[ref64] Haeusler D.; Decristoforo C.; Frost J.; Gobalakrishnan S.; Huang Y. Y. Molecular Imaging: In Vivo Agents for the Diagnosis and Treatment of Cancer. Contrast Media Mol. Imaging 2018, 2018, 8541915. 30425608 10.1155/2018/8541915 PMC6217884

[ref65] Weisner J.; Gontla R.; van der Westhuizen L.; Oeck S.; Ketzer J.; Janning P.; Richters A.; Mühlenberg T.; Fang Z.; Taher A.; et al. Covalent-Allosteric Kinase Inhibitors. Angew. Chem., Int. Ed. 2015, 54 (35), 10313–10316. 10.1002/anie.201502142. 26110718

[ref66] Quambusch L.; Landel I.; Depta L.; Weisner J.; Uhlenbrock N.; Müller M. P.; Glanemann F.; Althoff K.; Siveke J. T.; Rauh D. Covalent-Allosteric Inhibitors to Achieve Akt Isoform-Selectivity. Angew. Chem., Int. Ed. 2019, 58 (52), 18823–18829. 10.1002/anie.201909857. PMC6972997 31584233

[ref67] Uhlenbrock N.; Smith S.; Weisner J.; Landel I.; Lindemann M.; Le T. A.; Hardick J.; Gontla R.; Scheinpflug R.; Czodrowski P.; Janning P.; Depta L.; Quambusch L.; Müller M. P.; Engels B.; Rauh D. Structural and Chemical Insights into the Covalent-Allosteric Inhibition of the Protein Kinase Akt. Chem. Sci. 2019, 10 (12), 3573–3585. 10.1039/C8SC05212C. 30996949 PMC6430017

[ref68] Gabizon R.; Shraga A.; Gehrtz P.; Livnah E.; Shorer Y.; Gurwicz N.; Avram L.; Unger T.; Aharoni H.; Albeck S.; Brandis A.; Shulman Z.; Katz B.-Z.; Herishanu Y.; London N. Efficient Targeted Degradation via Reversible and Irreversible Covalent PROTACs. J. Am. Chem. Soc. 2020, 142, 11734 10.1021/jacs.9b13907. 32369353 PMC7349657

[ref69] Nnadi C. I.; Jenkins M. L.; Gentile D. R.; Bateman L. A.; Zaidman D.; Balius T. E.; Nomura D. K.; Burke J. E.; Shokat K. M.; London N. Novel K-Ras G12C Switch-II Covalent Binders Destabilize Ras and Accelerate Nucleotide Exchange. J. Chem. Inf. Model. 2018, 58 (2), 464–471. 10.1021/acs.jcim.7b00399. 29320178 PMC6179444

