# Supplementary material for: Tunable Methacrylamides for Covalent Ligand Directed Release Chemistry
Source: J Am Chem Soc. 2021 Mar 25;143(13):4979–92. doi: 10.1021/jacs.0c10644 (PMC8041284; doi:10.1021/jacs.0c10644)
Supplement: Supplementary file 1 — ja0c10644_si_001.pdf [file ja0c10644_si_001.pdf]

Tunable methacrylamides for covalent ligand directed release chemistry

Rambabu N. Reddi[1,*], Efrat Resnick[1,*], Adi Rogel[1], Boddu Venkateswara Rao[1], Ronen Gabizon[1], Kim Goldenberg[1,2], Neta Gurwicz[2], Daniel Zaidman[1], Alexander Plotnikov[3], Haim Barr[3], Ziv Shulman[2], Nir London[1,#]

[1] Dept. of Organic Chemistry, The Weizmann Institute of Science, Rehovot, 7610001, Israel.

[2] Dept. of Immunology, The Weizmann Institute of Science, Rehovot, 7610001, Israel.

[3] Wohl Institute for Drug Discovery of the Nancy and Stephen Grand Israel National Center for Personalized Medicine, The Weizmann Institute of Science, Rehovot, 7610001, Israel.

[*] Equal contribution

[#] To whom correspondence should be addressed, nir.london@weizmann.ac.il

Keywords: Acrylamides, Turn-on fluorescence, Targeted Covalent inhibitors, Bioconjugation, Electrophiles

Supplementary information

Supplementary figures

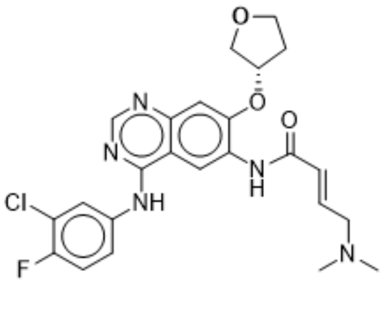

Afatinib

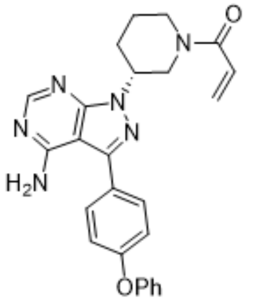

Ibrutinib

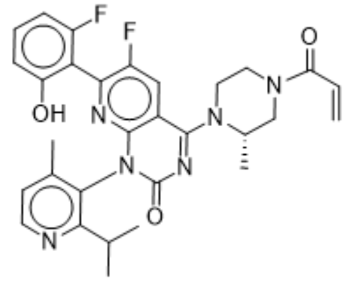

Amg-510

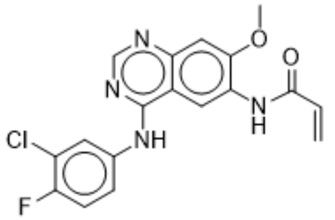

4a

Figure S1. Chemical structures of Afatinib, Ibrutinib, AMG-510 and **4a.**

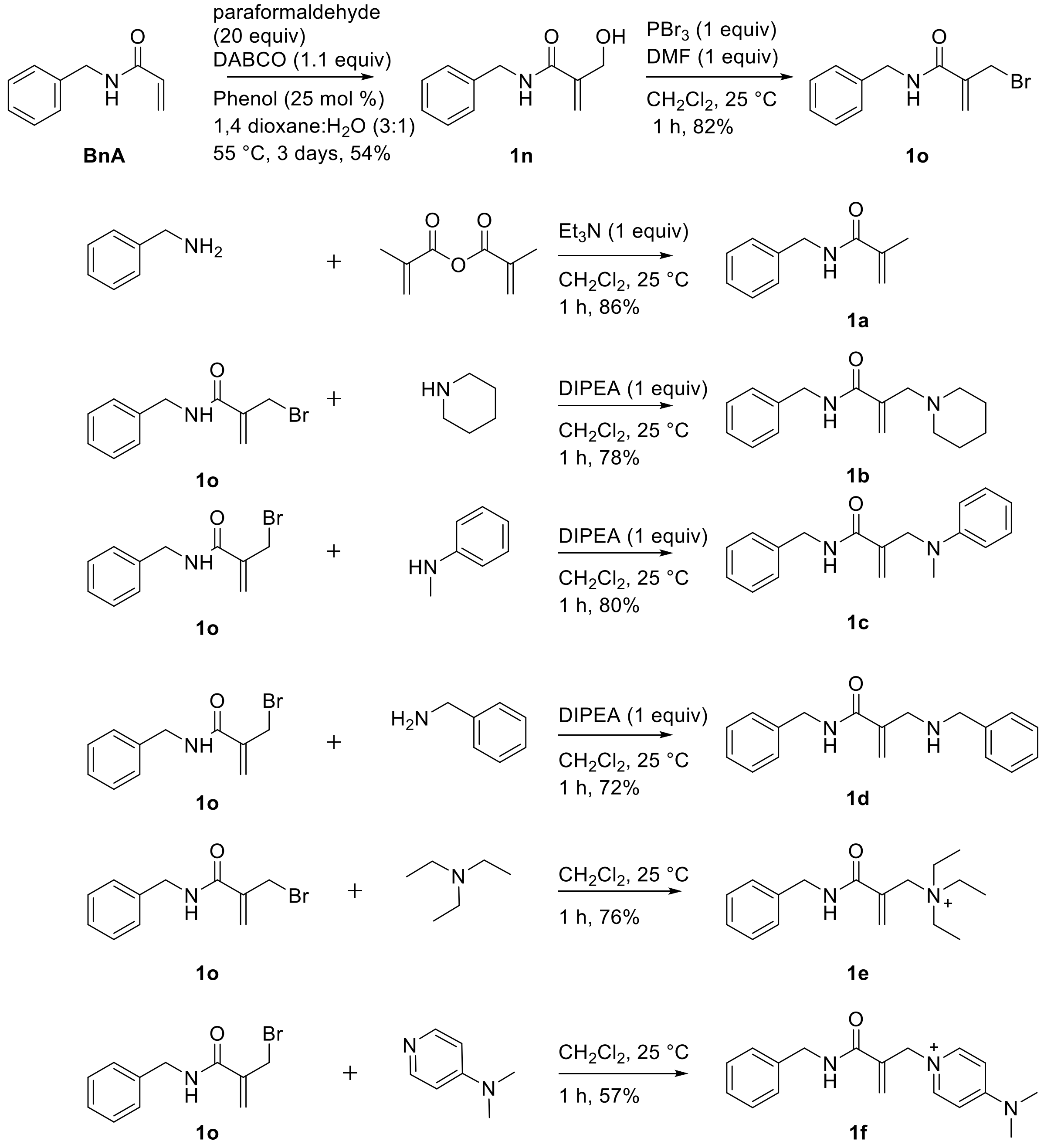
BnA
paraformaldehyde (20 equiv)
DABCO (1.1 equiv)
Phenol (25 mol %)
1,4 dioxane:H_2O (3:1)
55 °C, 3 days, 54%
1n
PBr_3 (1 equiv)
DMF (1 equiv)
CH_2Cl_2, 25 °C
1 h, 82%
1o
Et_3N (1 equiv)
CH_2Cl_2, 25 °C
1 h, 86%
1a
DIPEA (1 equiv)
CH_2Cl_2, 25 °C
1 h, 78%
1b
DIPEA (1 equiv)
CH_2Cl_2, 25 °C
1 h, 80%
1c
DIPEA (1 equiv)
CH_2Cl_2, 25 °C
1 h, 72%
1d
CH_2Cl_2, 25 °C
1 h, 76%
1e
CH_2Cl_2, 25 °C
1 h, 57%
1f

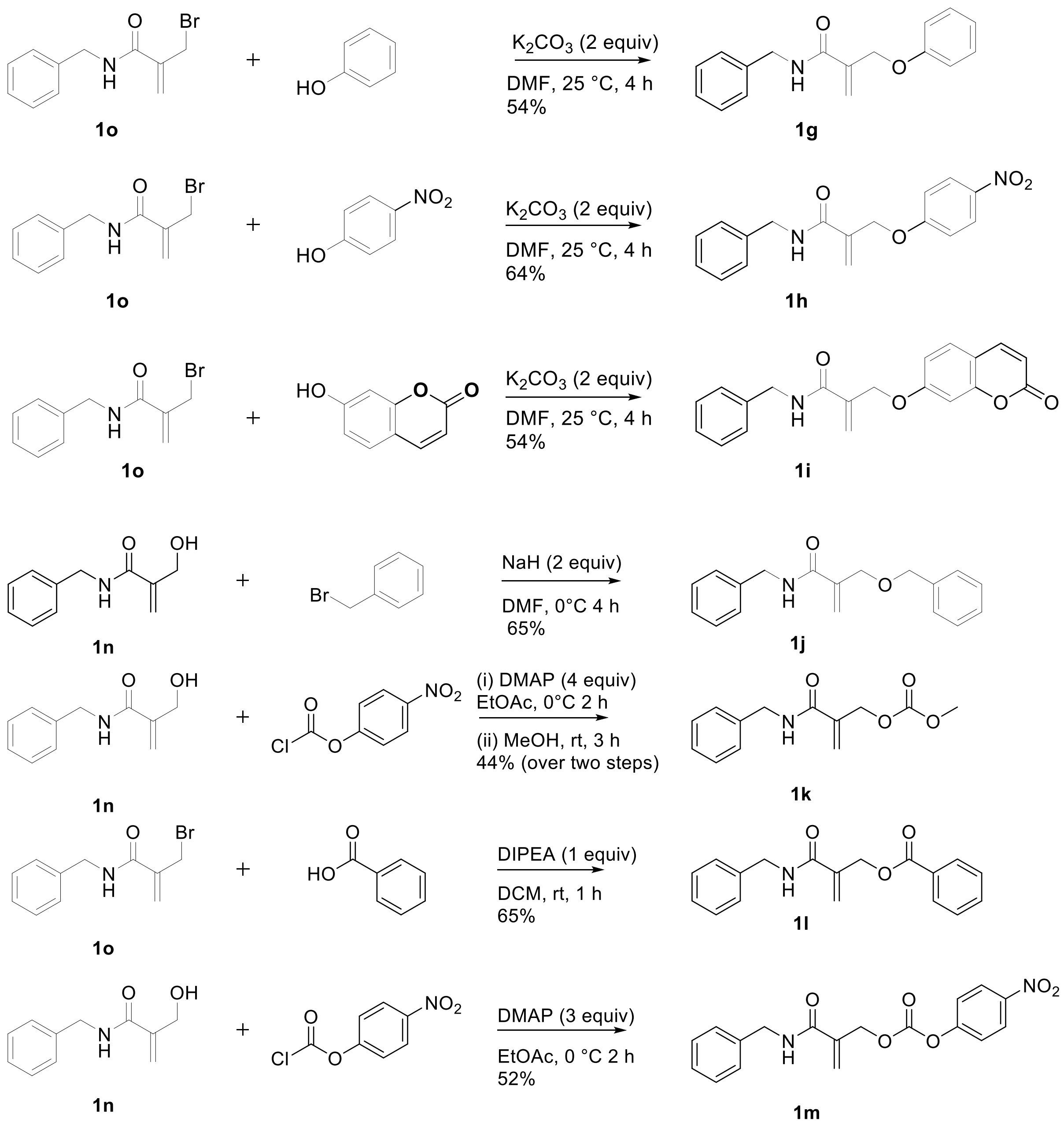

Figure S2. Synthesis of α-substituted N-benzyl-methacrylamides (**1a**-**1m**).

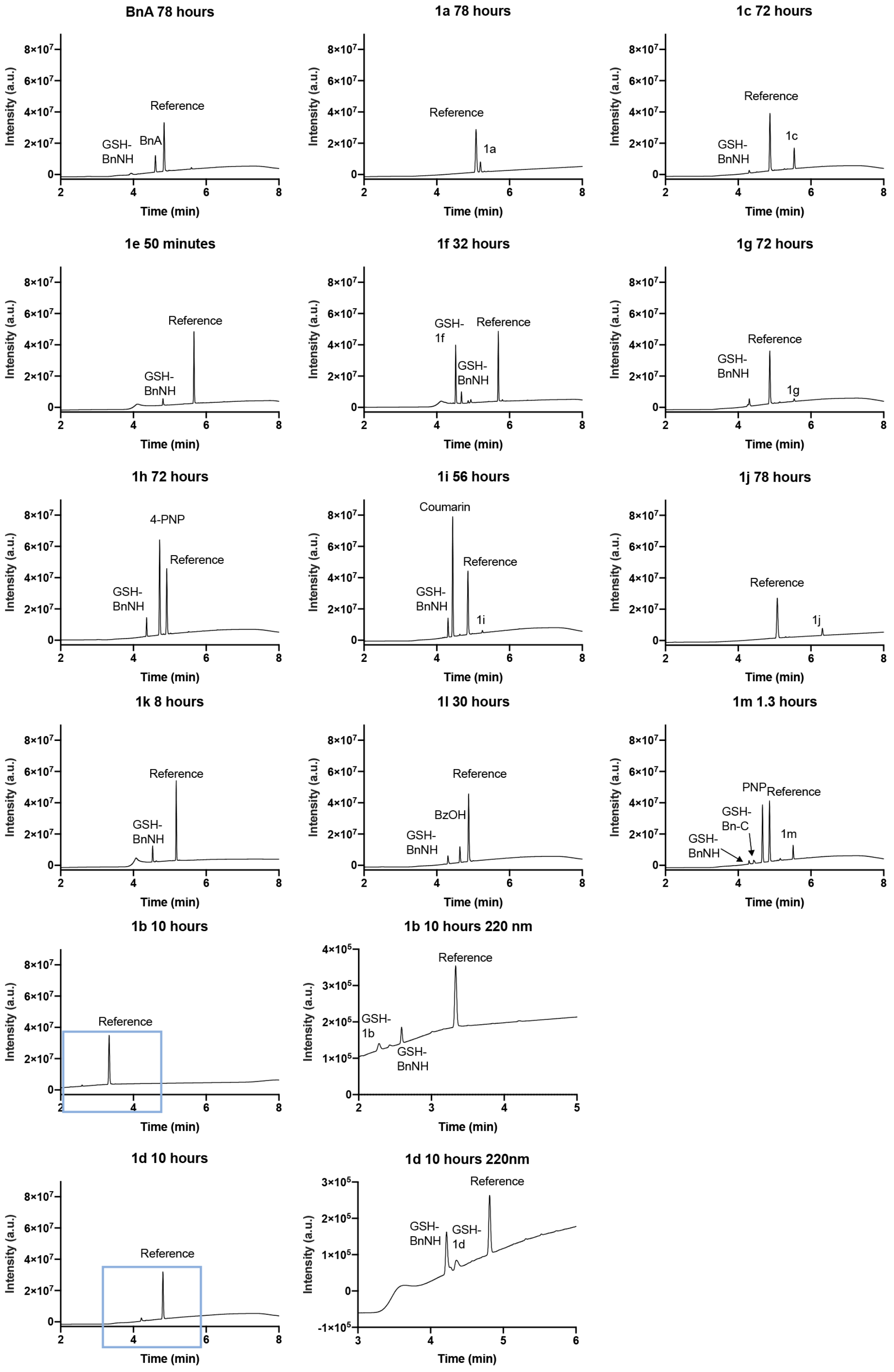

Figure S3. GSH consumption assay with model compounds. UV spectra (220-400 nm) of the LC/MS analysis of 5 mM GSH incubated with 100 µM of each model compound (**1a-1m**) at the last measured point of the GSH $t_{1/2}$ experiment (Figure 2B, Table 1). Peak at 4 min in compounds **1e**, **1f** and **1k** is a contamination stuck on the column. Compounds **1b** and **1d** have weak UV absorbance, an additional zoomed spectrum at 220 nm is included.

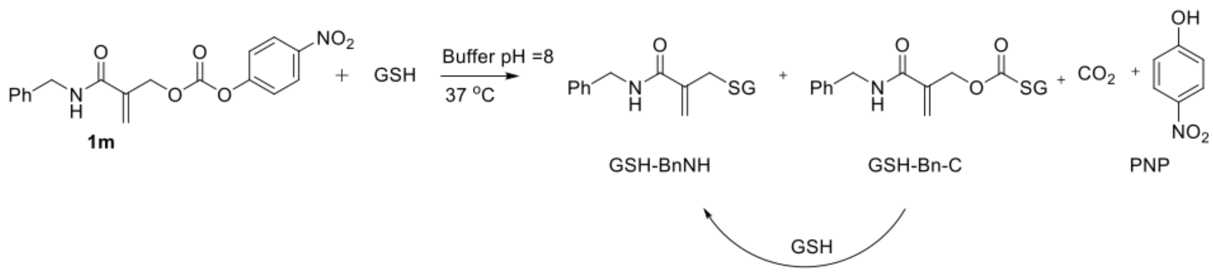

Figure S4. Two step reaction pathway for the reaction of GSH with compound **1m** at pH = 8, 37 ºC. See Fig. S3 for identification of the intermediate adduct.

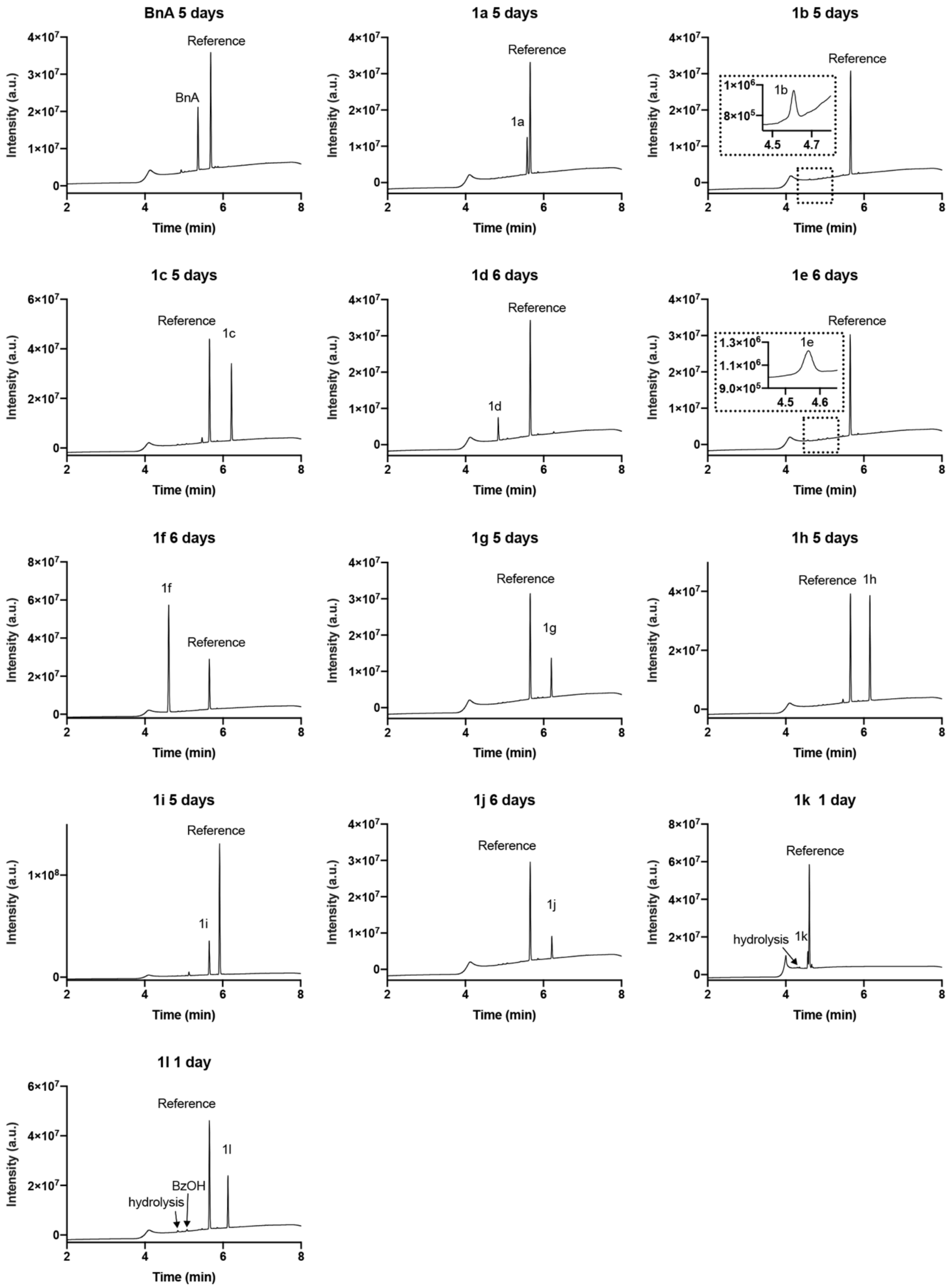

Figure S5. Buffer stability of model compounds. UV spectra (220-400 nm) of the LC/MS traces of model compounds (100 µM;**1a**-**1l**) incubated with 4-nitrocyano benzene (100 µM) in PBS buffer at pH 8, 37 ºC. Compounds **1b** and **1e** have weak UV absorbance, an additional zoomed spectrum is added. Peak at 4 min is a contamination on the column.

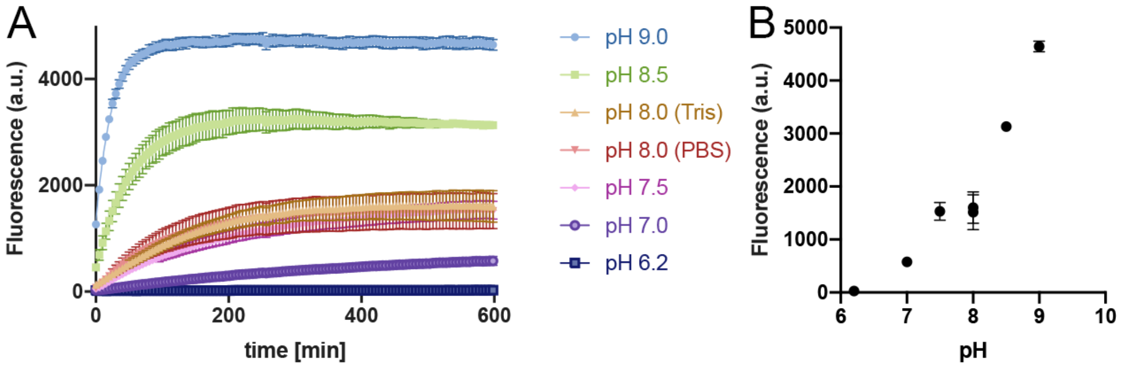

Figure S6. Effect of pH on the release and fluorescence of coumarin by the reaction between **1i** and GSH **A.** Time dependent coumarin release from **1i** (100 µM, n=4) at a fixed GSH concentration (5 mM) in PBS buffer (pH 6.2, 7.0, 7.5 and 8.0) and in Tris buffer (pH 8.0, 8.5 and 9.0) at Ex/Em = 385/435 nm, pH 8 and 37 °C **B.** Effect of pH on the reaction of 5 mM GSH with 100 µM **1i** after 10 h (n= 4), 37 °C (Ex/Em = 385/435 nm).

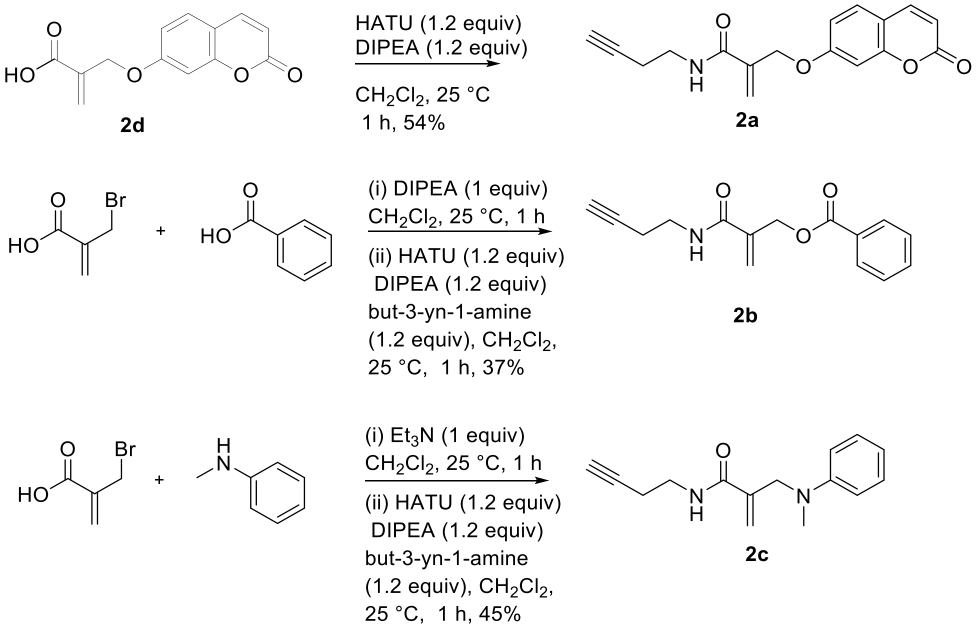

Figure S7. Synthesis scheme of α-substituted N-alkynyl-methacrylamides (**2a**-**2c**).

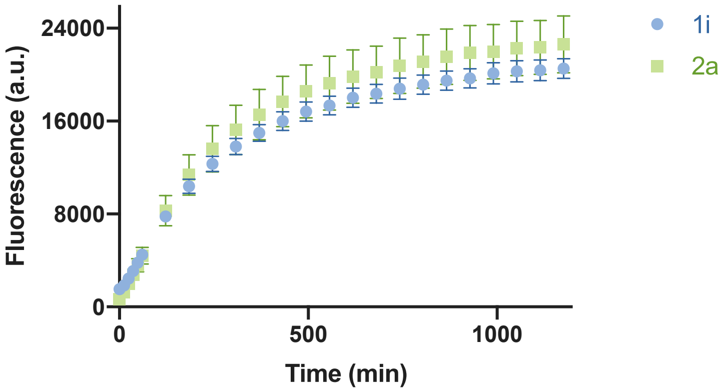

Figure S8. Release of 7-hydroxy coumarin triggered by the addition of 5mM GSH to 100 µM of either **1i** and **2a** at pH 8, 37 ºC, n=4, shows almost identical release rates. Fluorescence was measured at Ex/Em = 385/435 nm.

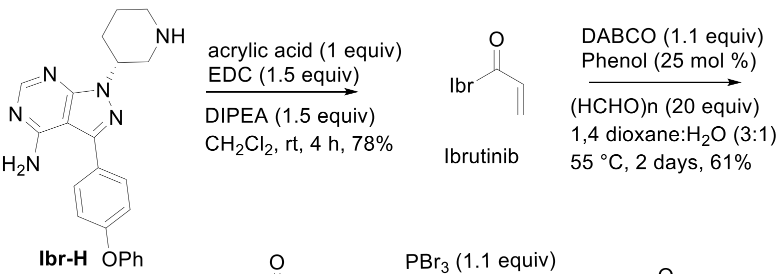

acrylic acid (1 equiv)
EDC (1.5 equiv)
DIPEA (1.5 equiv)
CH2Cl2, rt, 4 h, 78%
Ibr-H
Ibrutinib
DABCO (1.1 equiv)
Phenol (25 mol %)
(HCHO)n (20 equiv)
1,4 dioxane:H2O (3:1)
55 °C, 2 days, 61%

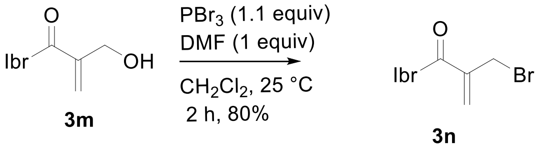

PBr3 (1.1 equiv)
DMF (1 equiv)
CH2Cl2, 25 °C
2 h, 80%
3m
3n

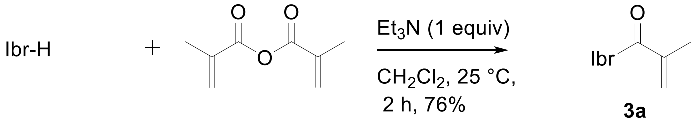

Ibr-H
Et3N (1 equiv)
CH2Cl2, 25 °C,
2 h, 76%
3a

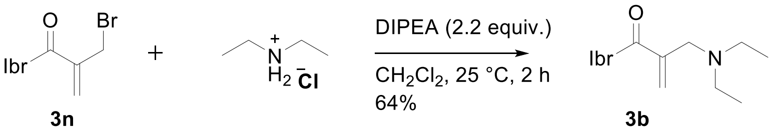

DIPEA (2.2 equiv.)
CH2Cl2, 25 °C, 2 h
64%
3n
3b

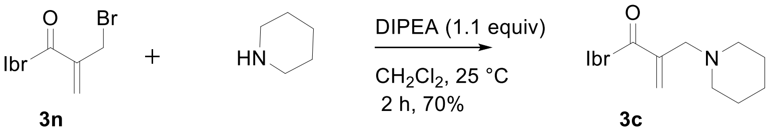

DIPEA (1.1 equiv)
CH2Cl2, 25 °C
2 h, 70%
3n
3c

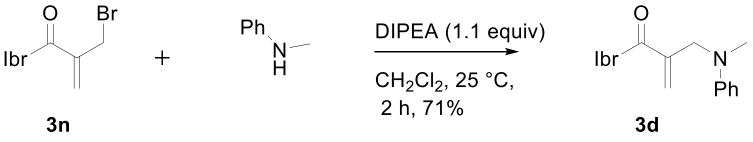

DIPEA (1.1 equiv)
CH2Cl2, 25 °C,
2 h, 71%
3n
3d

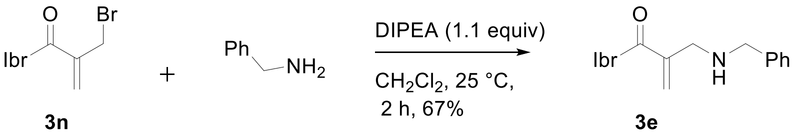

DIPEA (1.1 equiv)
CH2Cl2, 25 °C,
2 h, 67%
3n
3e

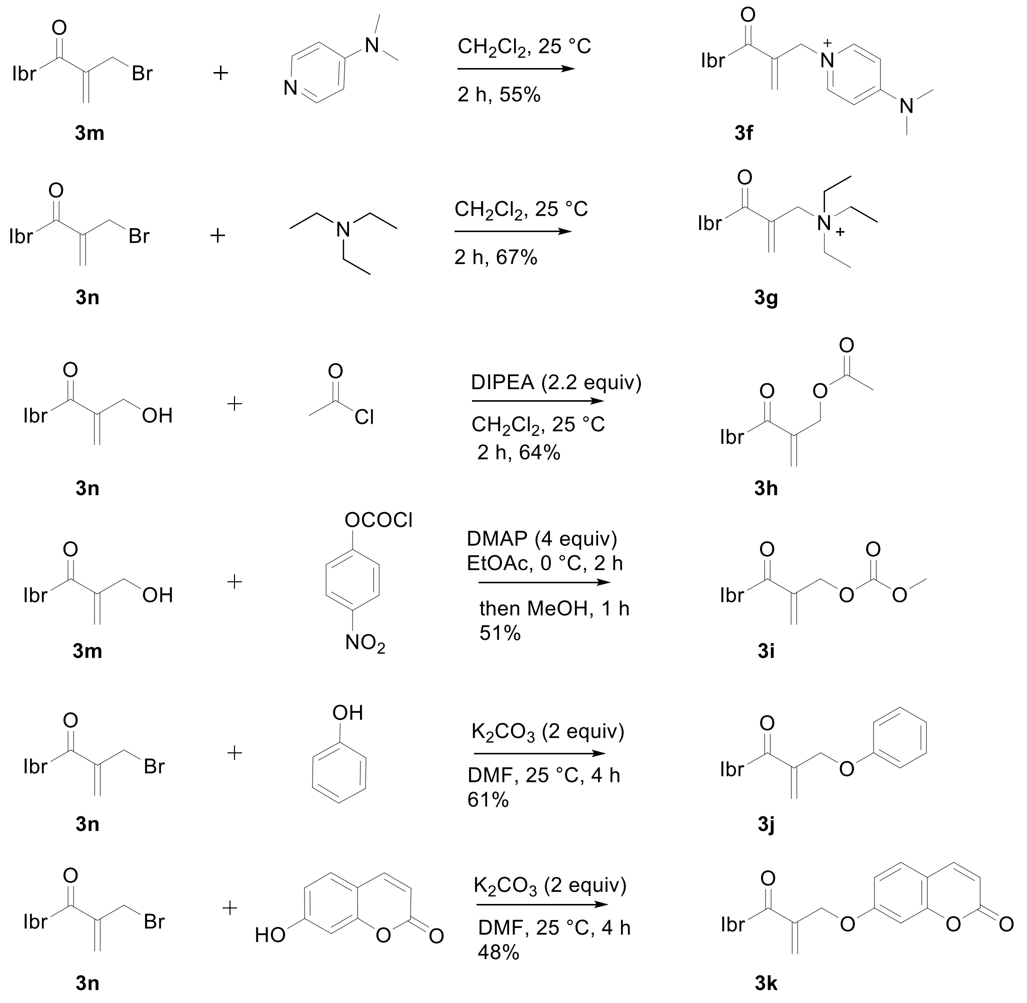

Figure S9. Synthesis scheme of α-substituted Ibrutinib derivatives.

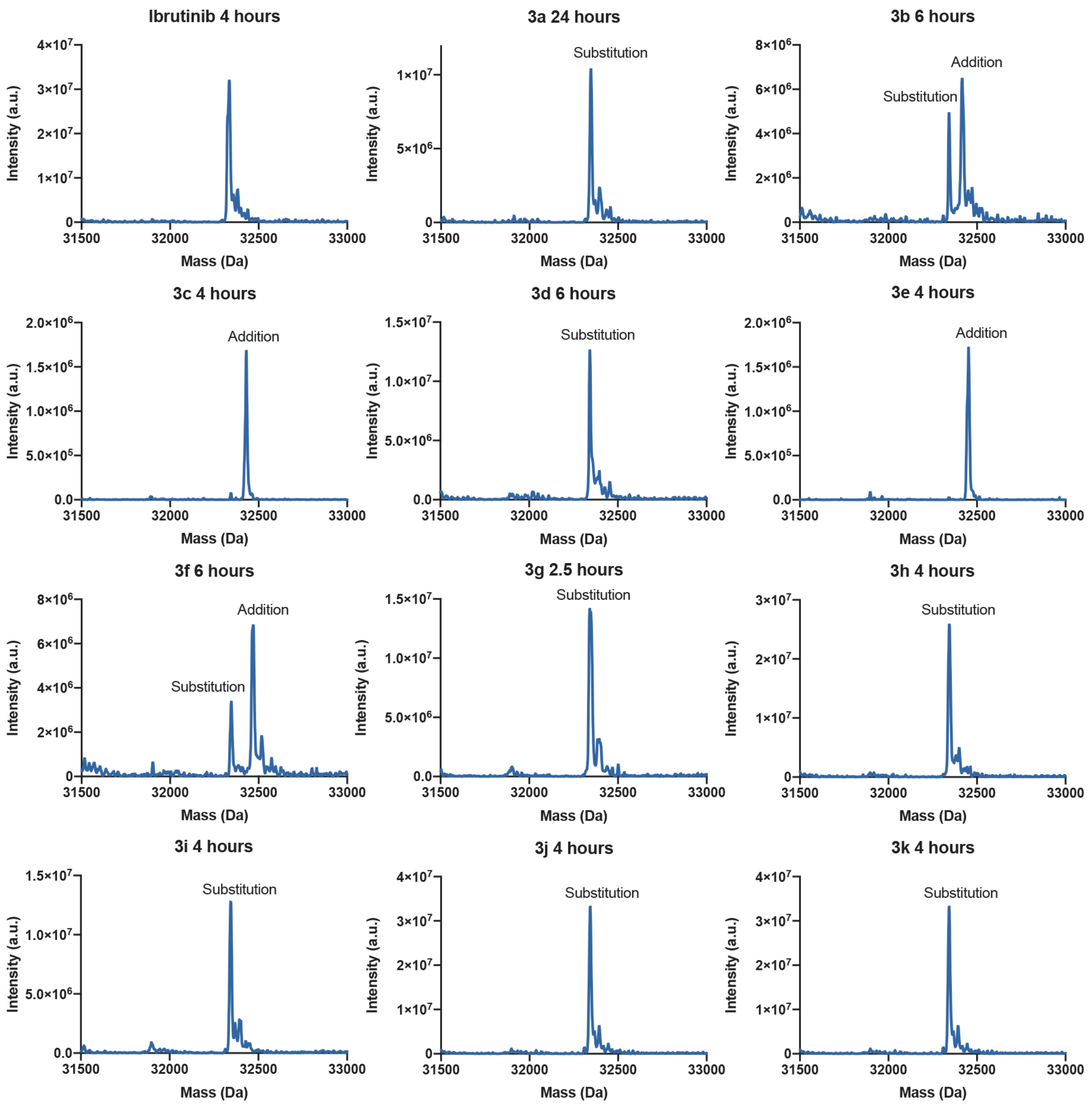

Figure S10. Deconvoluted MS spectra (intact protein LC/MS) of 2 μM BTK incubated with 2 μM ibrutinib derivatives **3a-3k** at pH 8.0, 25 ºC. Experiment was performed in triplicate, representative plots are presented.

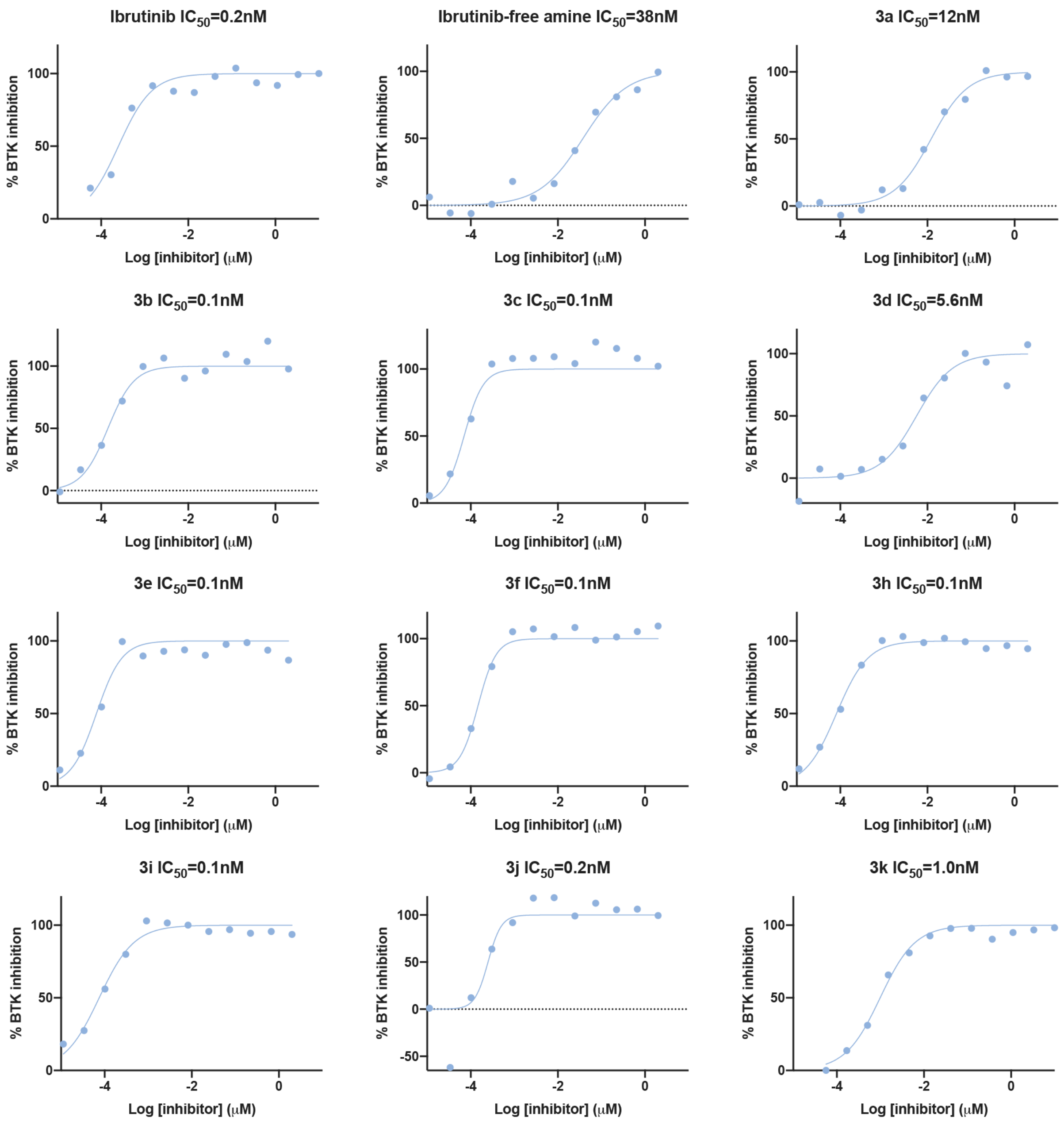

Figure S11. *In vitro* kinase activity assay performed by Nanosyn (Santa Clara, CA) with 0.6 nM BTK, 5 μM ATP against various ibrutinib analogs.

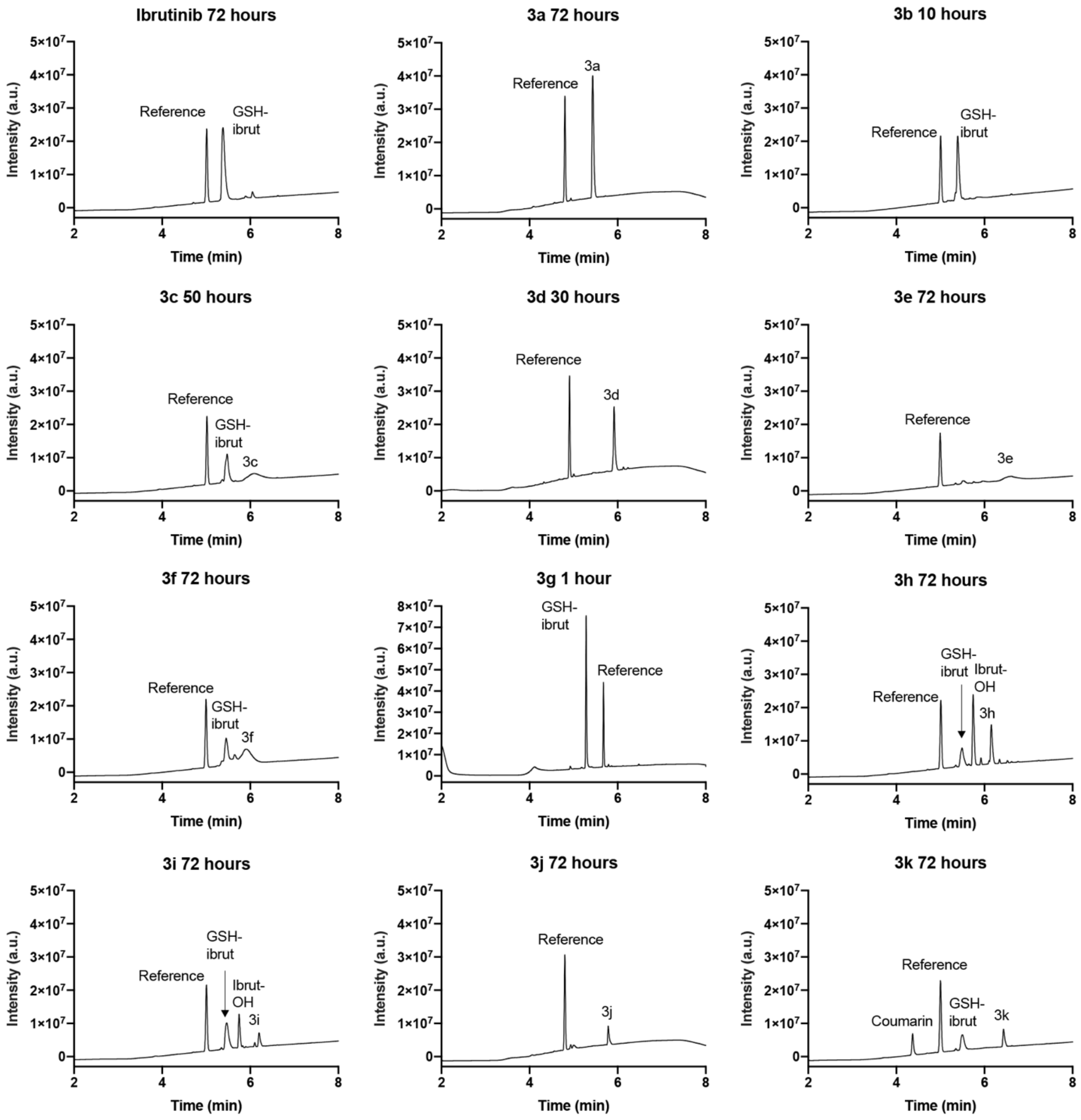

Figure S12. GSH consumption assay with ibrutinib analogs. UV spectra (220-400 nm) in LC/MS of 5 mM GSH incubated with 100 µM ibrutinib derivatives in PBS buffer:DMF (9:1), pH 8.0 (titrated after the addition of GSH) at 37 °C. We should note that compounds **3h** and **3i** are hydrolyzed to the corresponding alcohol after 72 h incubation.

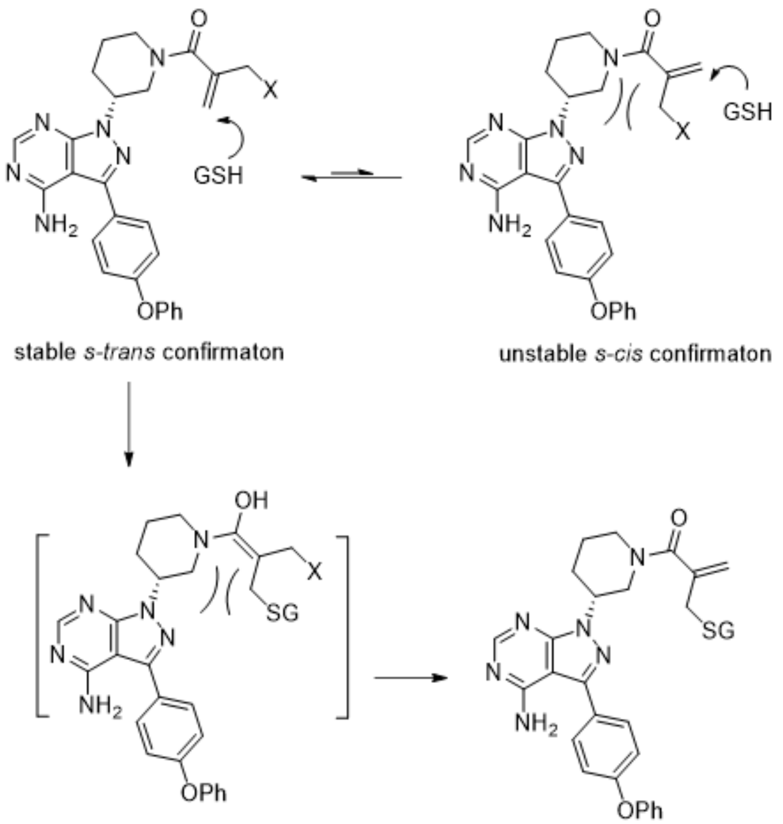

Figure S13. Possible conformations of **3a**-**3k** and GSH reaction intermediate. We hypothesize that the fixed geometry of the acrylamide facilitates faster reactions with BTK for these ibrutinib derivatives while making it difficult for GSH to reach the 'sandwiched' acrylamide which forms the sterically hindered conformation in which the piperidine and GSH are proximal in the transition state[1] .

1. Matos MJ, Oliveira BL, Martínez-Sáez N, *et al.* Chemo- and Regioselective Lysine Modification on Native Proteins. *J Am Chem Soc*. 2018;140(11):4004–4017.

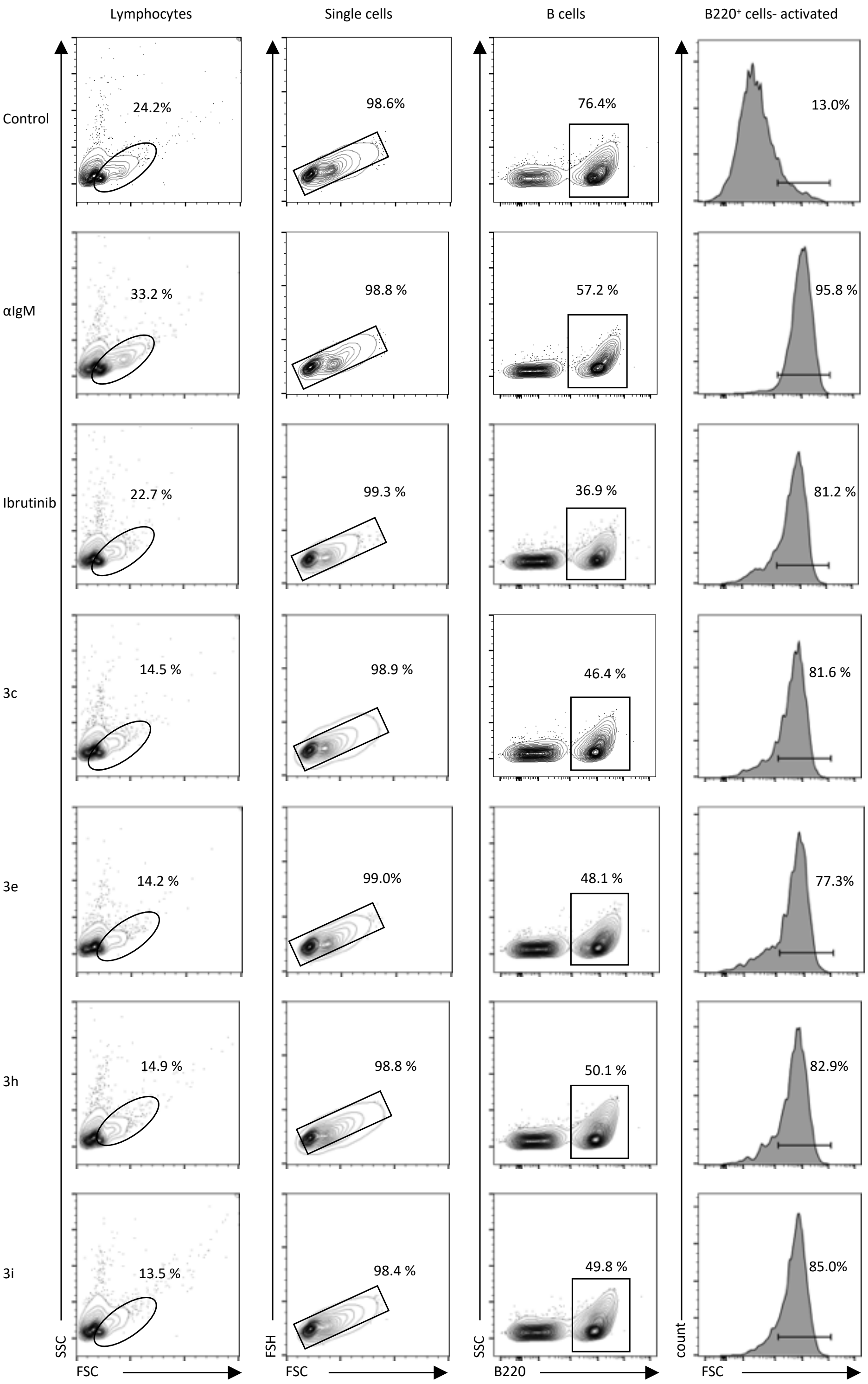

Figure S14. Representative FACS plots and gating strategies. Populations of cells were distinguished based on their forward and side scatter properties. Forward and side scatter give an estimation of the size and granularity of the cells respectively. Lymphocytes were gated at the bottom middle part of the plot, and debris and dead cells at the bottom left corner of the density plot were excluded. Using the FSC to FSH gating, population of singular cells were chosen from the lymphocytes population. B cells were defined as B220 positive lymphocytes with expression of over 10^4. Activated B cells were defined as CD86 positive B cells were determined for untreated cells and aIgM treated cells.

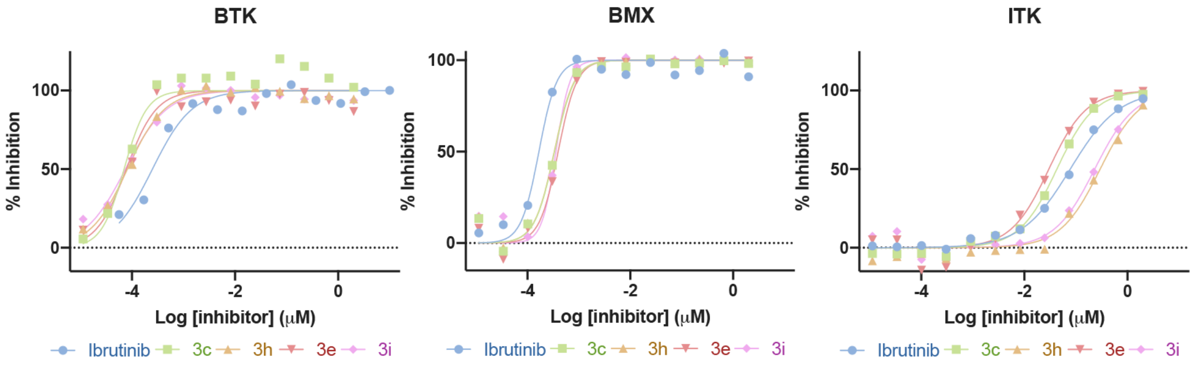

Figure S15. *in-vitro* kinase activity assays with selected kinases see additional plots for BLK, EGFR and ERBB2 in Figure 5C of the main text.

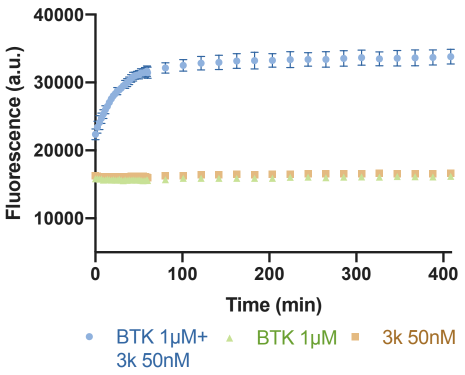

Figure S16. Incubation of **3k** with BTK at low equivalents (1 μM BTK; 50 nM **3k**; Ex/Em = 385/435 nm) still shows a detectable increase in fluorescence, but considerably slows down the reaction, to a point that the initial kinetics can be observed.

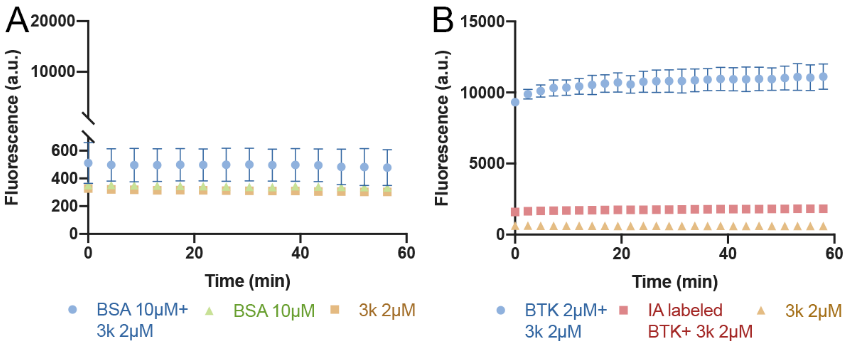

Figure S17. Time dependence of turn-on fluorescence with **3k** (Ex/Em = 385/435 nm). **A.** 10 µM BSA with 2 µM **3k** shows no reaction indicating the probes selectivity. **B.** 2 µM BTK fully labeled with IAA (red) compared to 2 µM non labeled BTK (blue) with 2 µM **3k**. The lack of signal for the labeled BTK indicated the fluorescence is triggered by a free cysteine.

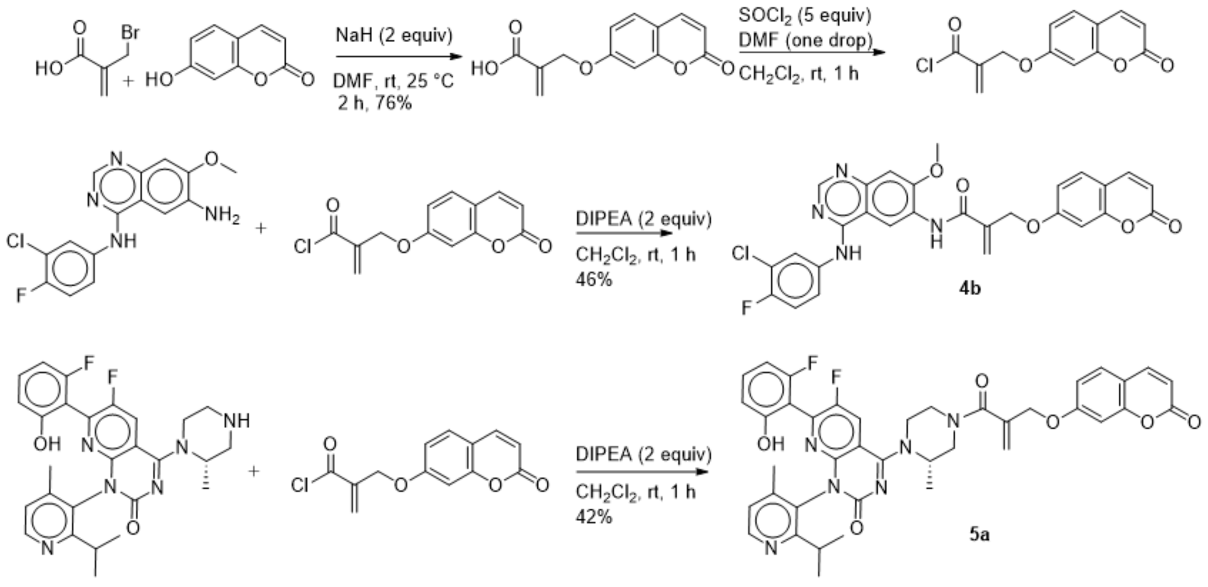

Figure S18. Synthesis scheme of **4b** and **5a**.

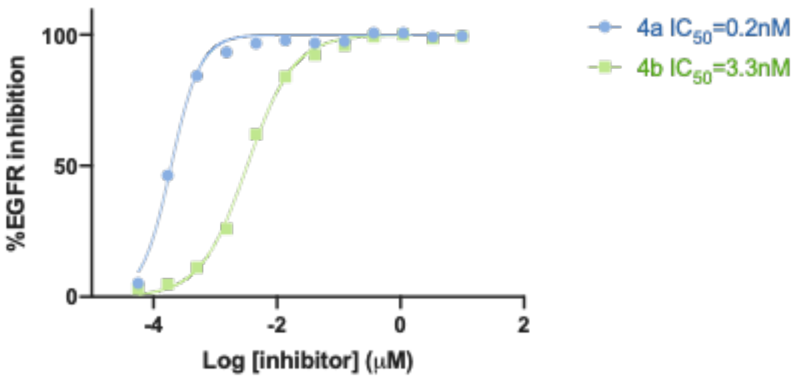

Figure S19. EGFR kinase activity assay for two afatinib analogs **4a** and **4b**. The assay contained 0.75 nM EFGR in 100 mM HEPES, pH 7.5, 0.1% BSA, 0.01% Triton X-100, 1 mM DTT, 10 mM $MnCl_2$. The reaction was initiated by 2-fold dilution into a solution containing 5 μM ATP and 1 μM substrate in the kinase buffer.

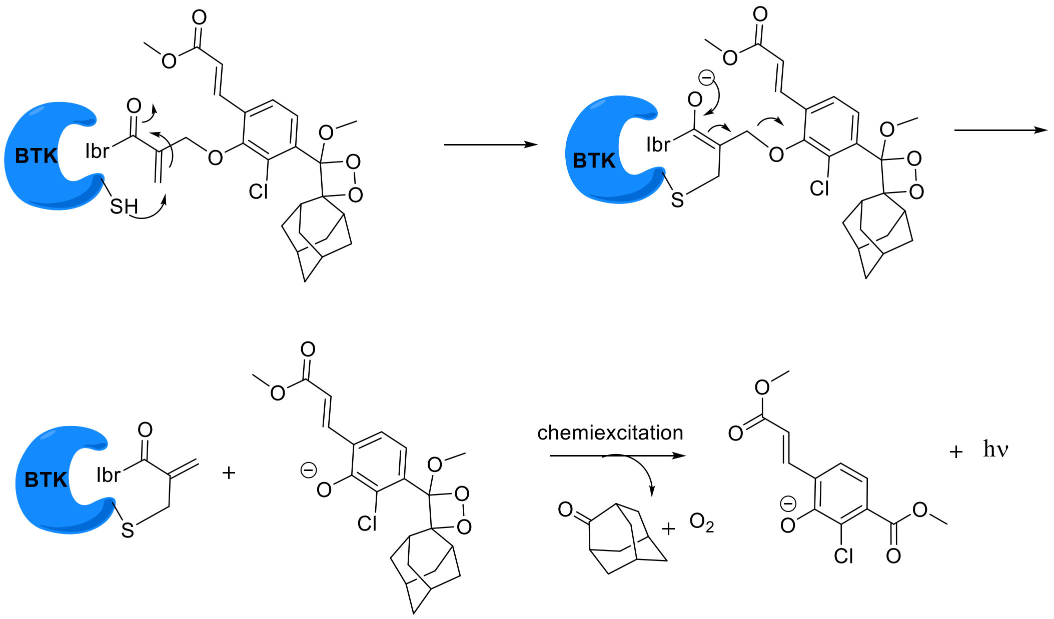

Figure S20. Mechanism of turn-on chemiluminescence of probe **3l** by BTK using CoLDR chemistry and subsequent dissociation pathway for the emission of a photon.

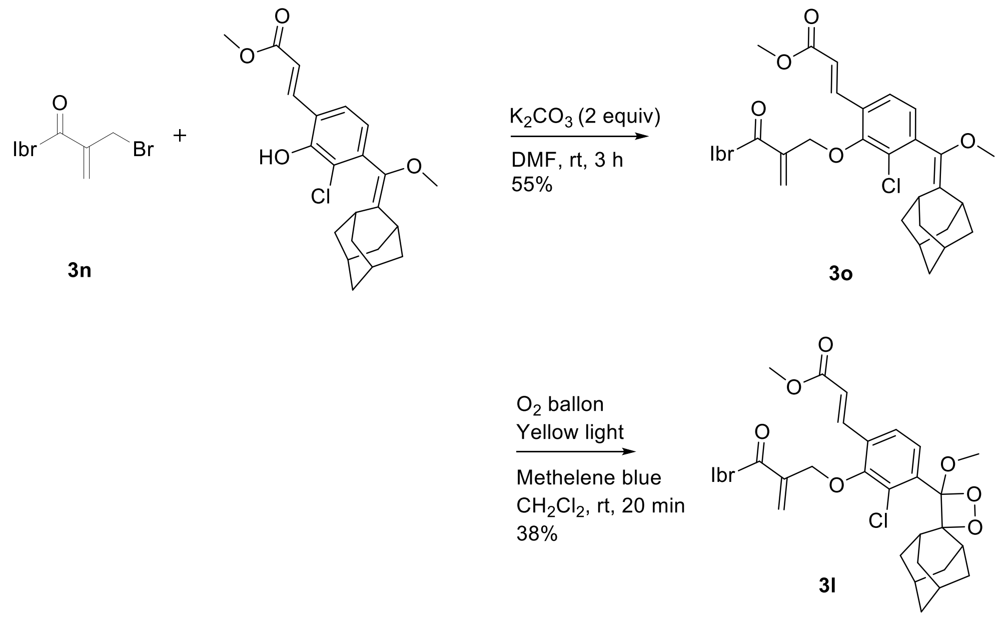

Figure S21. Synthesis scheme of **3l.**

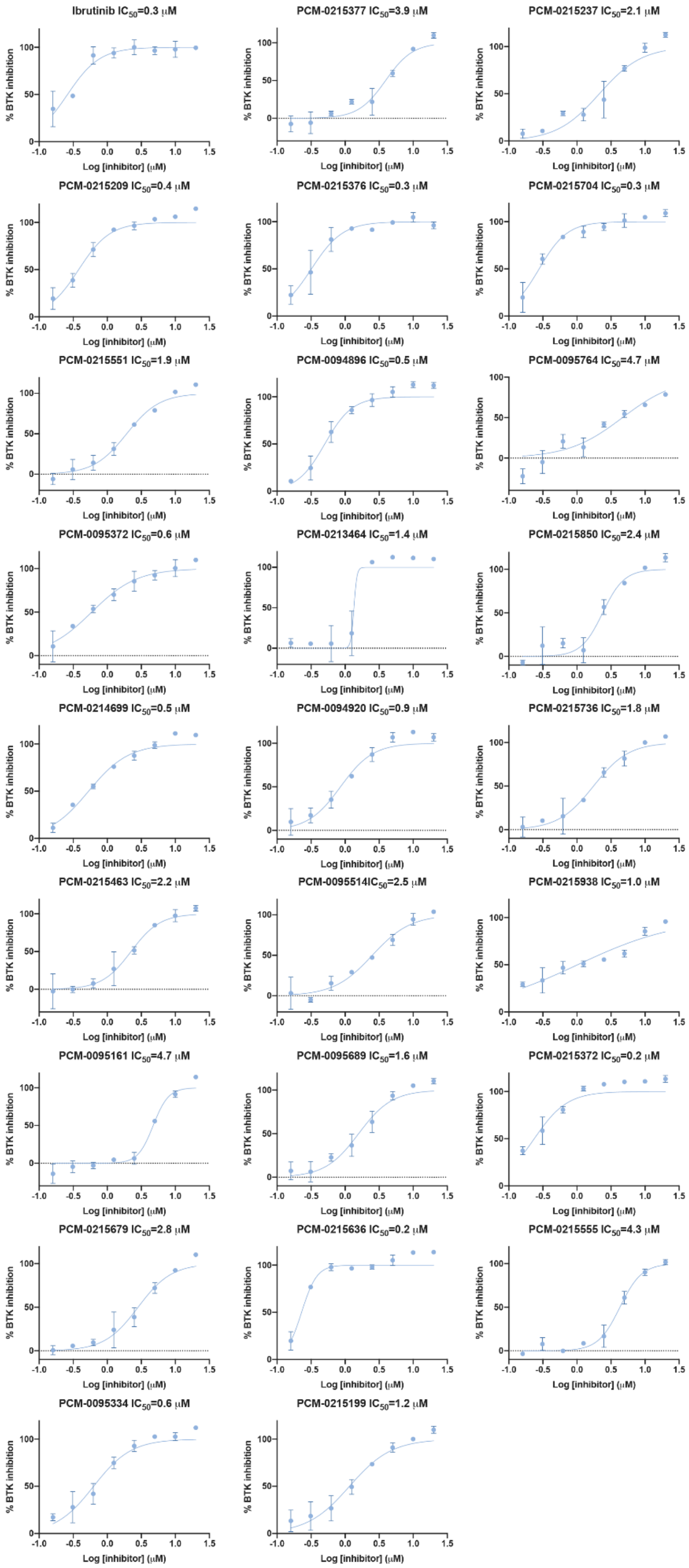

Figure S22. Dose response (n=2) of decrease in luminescence signal as a function of BTK binding for hits from HTS with **3l** (Figure 7C, 7D). See Dataset S3 for compound details.

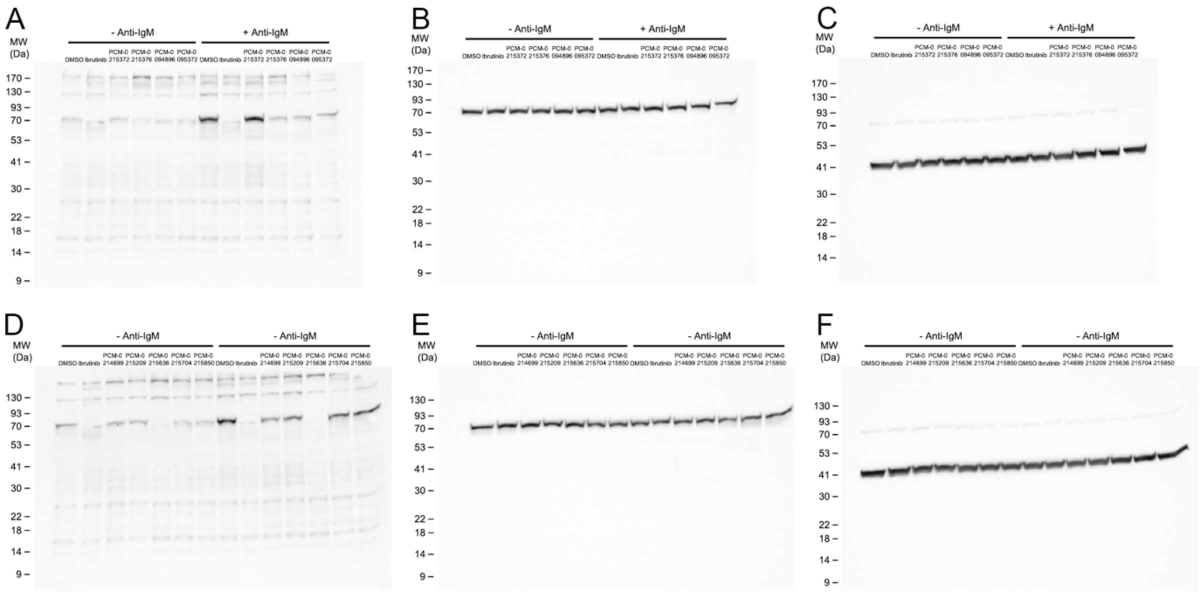

Figure S23. Single dose BTK inhibition in mino cells with hit compounds. Cells were incubated for 1 h with 500 nM inhibitors followed by 10 min activation with anti-IgM. **A,D** anti phospho-BTK. **B,E** anti total BTK. **C,F** anti beta actin.

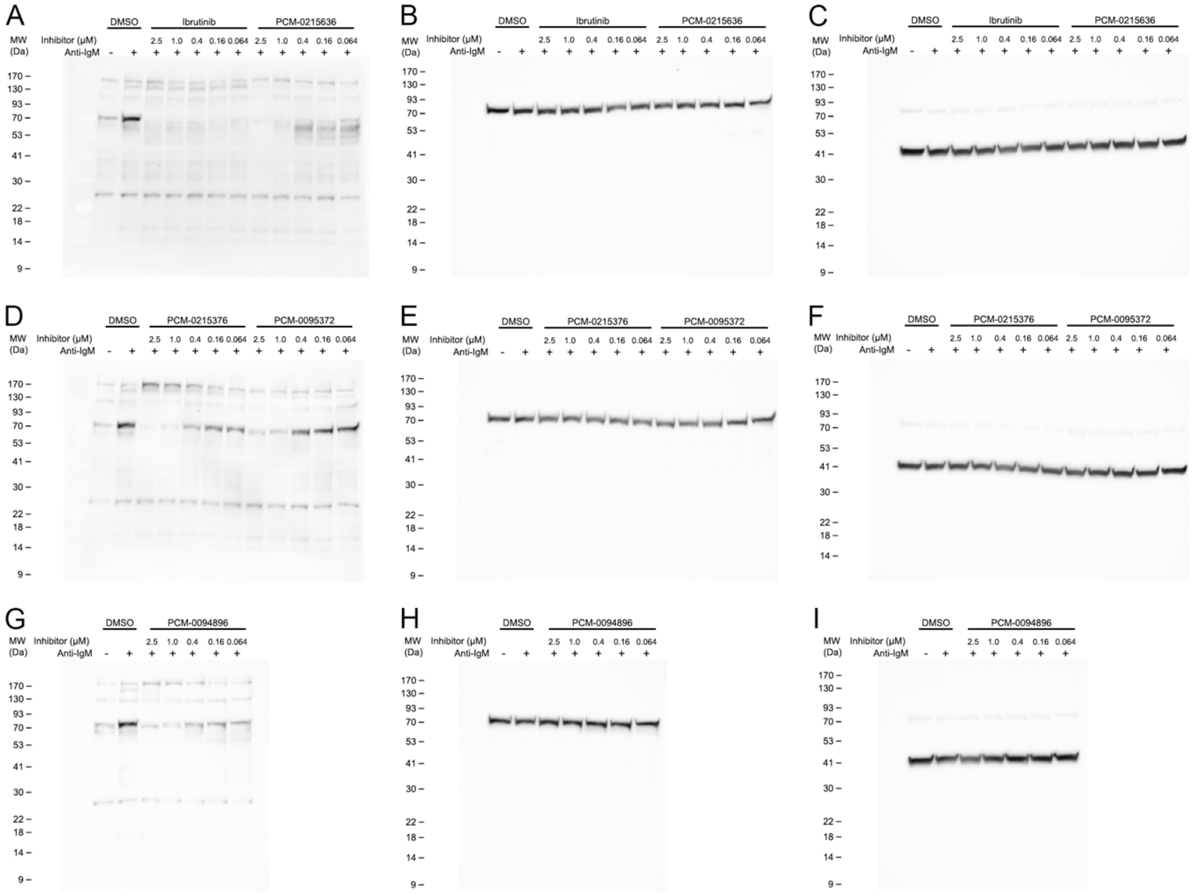

Figure S24. BTK inhibition in mino cells with hit compounds. Cells were incubated for 1 h with 2.5 µM- 0.064 µM inhibitors followed by 10 min activation with anti-IgM. **A,D.G** anti phospho-BTK. **B,E,H** anti total BTK. **C,F,I** anti beta actin. These are the uncut gels for the data presented in Figure 7G.

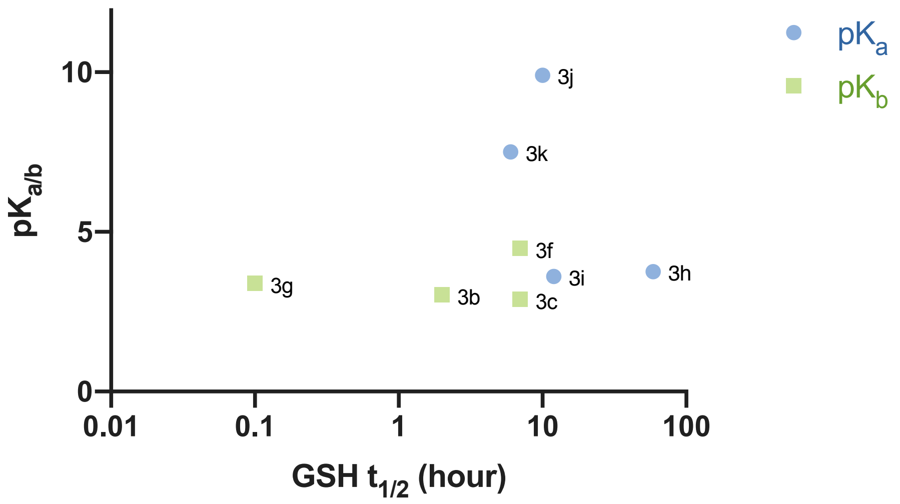

Figure S25: GSH $t_{1/2}$ of ibrutinib derivatives Vs pK_a of their protonated leaving groups.

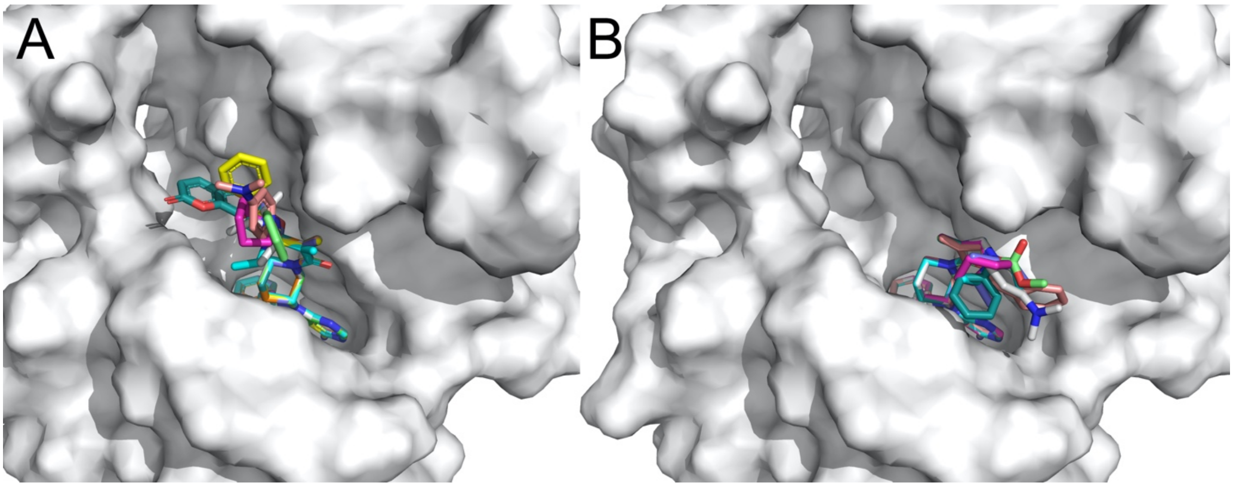

Figure S26: Moleculear modeling of ibrutinib methacrylamide analogs in their **A.** s-cis conformation or **B.** s-trans conformation. The models show that in both conformations there is room for the modification in the enzyme binding site and in some cases the substitutions may form additional interactions with the protein.

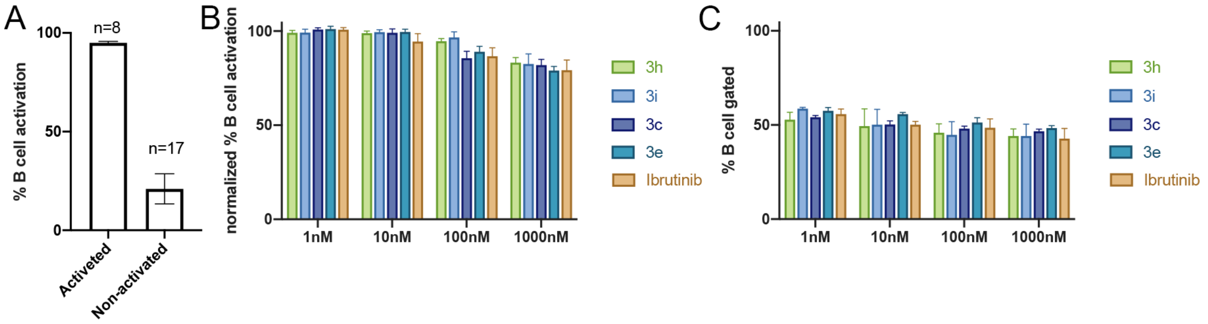

Figure S27. Inhibition of B cell response**. A.** %B cell activation in activated and non-activated cells. **B.** Dose dependent inhibition of B cell response after anti-IgM-induced activation and treatment with ibrutinib analogs for 24 h (n=6) with full range y axis. **C.** % of B cell gated for each condition in panel B (n=6).

Supplementary tables

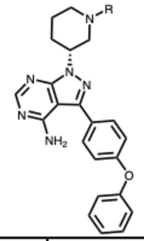

Compound	R[a]	BTK $t_{1/2}$ (min)[b]	BTK Substitution/ Addition	IC_{50} (nM)	GSH $t_{1/2}$ (hour)[b]	GSH Substitution/ Addition
**Ibrutinib**	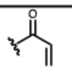	<5	Addition	0.2	2	Addition
**3a**	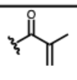	>420	Addition	12.2	No reaction	Addition
**3b**	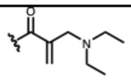	<5	Substitution/ Addition ~40%/60%	0.1	2	Substitution/ Addition ~66%/33%
**3c**	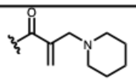	<5	Addition	0.1	7	Substitution/ Addition ~40%/60%
**3d**	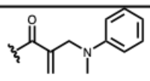	47	Substitution	5.6	>100	Substitution
**3e**	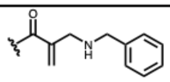	<5	Addition	0.1	>100	Substitution
**3f**	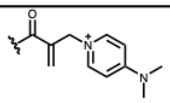	<5	Substitution/ Addition ~30%/70%	0.1	7	Substitution/ Addition ~40%/60%
**3g**	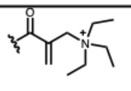	<5	Substitution		0.1	Substitution
**3h**	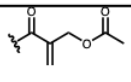	<5	Substitution	0.1	59	Substitution
**3i**	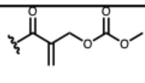	<5	Substitution	0.1	12	Substitution
**3j**	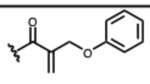	8	Substitution	0.2	10	Substitution
**3k**	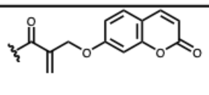	<5	Substitution	1.0	6	Substitution

Table S1: Properties of α-substituted derivatives of ibrutinib.

[a] Substituted α-methacrylamides analogs of ibrutinib.
[b] Reactivity towards GSH ($t_{1/2}$) and reaction type were assessed via LC/MS (Figure S10, S12; IC_{50} determined in a kinase activity assay see Figure S11)

Supplementary methods

IsoTOP ABPP sample preparation

The preparation of IsoTOP-ABPP samples was performed essentially as described in Zanon et al.[1]. Experiments were conducted in quadruplicates. Mino cells were incubated for 2 h with 1 μM compound (or with DMSO), collected by centrifuge at 300 g for 5 min followed by ice cold PBS wash. For lysis, samples containing 18 million cells were dispersed in 0.5 mL of RIPA buffer (Sigma, R0278), incubated with occasional vortexing for 30 min on ice, followed by centrifugation at 21,000 g for 15 min. The protein concentration in the samples was determined using BCA assay (Pierce 23227), and each sample was diluted to 1.7 mg/mL using PBS. To each sample, 5 μL of 10 mM IA-alkyne was added, followed by 1 h incubation at room temperature in the dark. 10 μL of 5 mM DesThioTag was added (Light for the compound-treated samples, heavy for the DMSO-treated samples), followed by 18 μL of $CuSO_4$:THPTA (100 mM), and the click reaction was initiated by addition of 15 μL of 150 mM sodium ascorbate (freshly dissolved in water). The samples were incubated on a rotary shaker for 1 h at room temperature. The compound-treated and DMSO-treated samples were mixed with 4 mL methanol, 1 mL chloroform and 2 mL water on ice, vortexed and centriduged at 3200 g for 10 min at 4°C. The top layer was aspirated, and 3 mL methanol was added, followed by centrifugation and aspiration of the supernatant. The pellets were air dried and stored at -80°C until the following treatment. The pellets were resuspended in 0.3 mL of 8 M urea freshly dissolved in PBS using probe sonication (8 sec total at 40% amplitude, 1 sec on/2 sec off, at room temperature). Following the resuspension, the samples were diluted with 1 mL of PBS. Then each sample was mixed with 1.3 mL of slurry containing 110 μL of streptavidin agarose beads (Thermo Streptavidin Agarose cat # 20349) prewashed and dispersed in 0.2% IGEPAL. The samples were incubated with rotation for 3 h at room temperature. The beads were pelleted by centrifugation at 2000 g for 2 min, transferred to spin columns and washed 3 times with 0.1% IGEPAL/PBS, 3 times PBS and 3 times water. The beads were then suspended in 8 M Urea/50 mM ammonium bicarbonate, and 15 μL of 31 mg/mL DTT were added, followed by incubation at 37°C for 45 min. The samples were cooled to room temperature and 15 μL of 74 mg/mL iodoacetamide were added, followed by 30 min incubation at room temperature in the dark, and addition of further 15 μL of 31 mg/mL DTT and incubation at room temperature for 30 min. 900 μL of 50 mM ammonium bicarbonate were added, and after 30 min incubation,

the beads were pelleted by centrifugation at 2000 g for 2 min, and resuspended in 200 µL of 1 M Urea/50 mM ammonium bicarbonate. At this point, modified trypsin (Promega V511A) was dissolved in trypsin buffer at 0.5 µg/µL, and 4 µL were added to each sample followed by overnight incubation at 37°C with shaking. 400 µL of 0.1% IGEPAL/PBS were added, and the beads were washed 3 times with 0.1% IGEPAL/PBS, 3 times PBS and 3 times water. The peptides were eluted by incubation with 200 µL of 50% acetonitrile + 0.1% TFA for 5 min, followed by two more portions of 100 µL of 50% acetonitrile + 0.1% TFA. The samples were dried by speedvac, and further purified using Oasis desalting columns (Waters), after which they were dried and run on the instrument. ULC/MS grade solvents were used for all chromatographic steps. Each sample was loaded using split-less nano-Ultra Performance Liquid Chromatography (Ultimate 3000, Thermo Scientific). The mobile phase was: A) H_2O + 0.1% formic acid and B) acetonitrile + 0.1% formic acid. Desalting of the samples was performed online using a reversed-phase Symmetry C18 trapping column (300 µm internal diameter, 5 mm length, 5 µm particle size; PepMap, Thermo Scientific). The peptides were then separated using a T3 HSS nano-column (75 µm internal diameter, 250 mm length, 1.8 µm particle size; Waters) at 0.35 µL/min. Peptides were eluted from the column into the mass spectrometer using the following gradient: 4% to 18%B in 78 min, 18% to 31%B in 29 min, 31% to 42%B in 7min, 42% to 90% in 13 min, maintained at 90% for 7 min and then back to initial conditions. The nanoUPLC was coupled online through a nanoESI emitter (10 µm tip; New Objective; Woburn, MA, USA) to a high resolution, quadrupole-Orbitrap mass spectrometer (Exploris 480, Thermo Scientific). Data was acquired in data dependent acquisition (DDA) mode, using a 'Top-Speed' method, with cycle time of 2sec. MS1 resolution was set to 120,000 (at 200 m/z), mass range of 380-1500 m/z, normalized AGC of 200% and maximum injection time was set to 50msec. MS2 resolution was set to 15,000, quadrupole isolation 1.4 m/z, AGC of 75%, dynamic exclusion of 35 sec and maximum injection time was set to Auto.

Data analysis for IsoTOP data

Analysis of IsoTOP-ABPP data was performed similarly to Zanon et al.[1] using MaxQuant 1.6.0.16. Human proteome (updated November 2020) was downloaded from Uniprot. For each protein not containing selenocysteine, a copy of the protein sequence containing a single mutation of cysteine to selenocysteine (C→U) was created for each cysteine in the sequence, in addition to an unmutated copy. The IsoTOPP labeles were then defined as Heavy/Light

labels with the following formulae: C(24)H(49)N(8)Cx(5)Nx(1)S(1)Se(-1) for the heavy label, and C(29)H(49)N(9)S(1)Se(-1) for the light label. In addition we added diagnostic peaks corresponding to the free amine generated by cleavage of the iodoacetamide alkyne (C(22)H(49)N(8)O(4)Cx(5)Nx(1)/C(27)H(49)N(9)O(4)), internal cleavage caused by attack of the triazole on the alpha carbon of the iodoacetamide moiety[2] (C(22)H(46)N(7)O(4)Cx(5)Nx(1)/ C(27)H(46)N(8)O(4)), cleavage of the peptide bond between azidolsyine and valine (C(10)H(25)N(2)O(3)Cx(5)Nx(1)/C(15)H(25)N(3)O(3)), and cleavage of the peptide bond between valine and desthiobiotin (C(10)H(16)N(2)O(2)). A multiplicity of 2 was set and amaximum number of labeled amino acids of 1. The digestion enzyme was set to Trypsin/P with a maximum number of missed cleavages of 2. No variable modifications were included. The "Re-quantify" option was enabled. Carbamidomethyl (C2H3NO) was used as fixed modification on cysteine. Contaminants were included. Peptides were searched with a minimum peptide length of 7 and a maximum peptide mass of 4,600 Da. "Second peptides" and "Dependent peptides" were disabled and the option "Match between run" was enabled with a Match time window of 0.7 min and an alignment window of 20 min. An FDR of 0.01 was used for Protein FDR, PSM FDR and XPSM FDR. After MaxQuant analysis, the data for each compound was analyzed separately. Following data analysis, reverse and contaminant peptides were removed. Only peptides for which non-zero total intensities were measured for at least two of the replicates were analyzed. Average H/L ratios were calculated as the ratio of the sum of high intensities in the replicates to the sum of the low intensities. Ratios that were above 20 or infinite (due to the sum being zero for the low intensities) were defined as 20.

Sample preparation for Pulldown

4 million mino cells were incubated for 1 h with 1 μM compound or with DMSO, followed by additional 1 h incubation with 10 μM ibrutinib-alkyne[3]. Cells were collected at 300 g for 5 min followed by ice cold PBS wash. For lysis, cells were dispersed in 50 μM of RIPA buffer (Sigma, R0278), incubated with occasional vortexing for 30 min on ice, followed by centrifugation at 21,000 g for 15 min. The protein concentration in the samples was determined using BCA assay (Pierce 23227), and each sample was diluted to 2 mg/mL using PBS. For 250 μL samples, 5 μL of 5 mM biotin azide and 9 μL of 100 mM $CuSO_4$:THPTA complex were added. The click reaction was initiated by addition of 7.5 μL of 150 mM sodium ascorbate, and the samples were incubated at room temperature for 1 h. The samples were then precipitated

with methanol:chloroform as described for the IsoTOP-ABPP samples, with ¼ of the volume of solvent due to the smaller sample volume. Dry pellet was resuspended in 1.2% SDS in PBS (250 µL), sonicated as described before, and heated to 90°C for 5 min. The samples was then diluted to 1.5 mL with PBS, and 50 µL of streptavidin agarose beads (Thermo Streptavidin Agarose cat # 20349), prewashed with 0.2% SDS in PBS, were added, followed by 3 h incubation at room temperature. Following the incubation, the beads were centrifuged 2 min 2000 g, and washed 4 times, with the following buffers (4 mL in each wash): 2% SDS; 0.1% sodium deoxycholate, 1% Triton X-100, 0.5 M NaCl, 1 mM EDTA, 50 mM HEPES pH 7.5; 0.25 M NaCl, 0.5% IGEPAL, 0.5% sodium deoxycholate, 1 mM EDTA, 10 mM Tris pH 8.1; 50 mM Tris pH 7.4, 50 mM NaCl. The beads were then resuspended in PBS and transferred to a clean Eppendorf tube. Buffer was removed to leave a volume of 100 µL, and 100 µL of 50 mM ammonium bicarbonate + 10% SDS was added, and the samples were heated to 96 °C for 5 min. The samples were then centrifuged and the denatured, eluted proteins were transferred to new tubes. 7.5 µL of 0.1 M DTT was added, and samples were incubated at 65°C for 45 min. After the samples had cooled, 7.5 µL of iodoacetamide (0.2 M) were added, and the samples were incubated in the dark for 40 min at room temperature. 1/10 volume of 12% phosphoric acid was added, and the samples were diluted 6-fold with 90% methanol + 50 mM ammonium bicarbonate. The samples were then loaded on s-trap micro columns (Protify) and the columns were washed 3 times with 150 µL of 90% methanol + 50 mM ammonium bicarbonate. Then, 20 µL of 0.05 µg/µL of trypsin in 50 mM ammonium bicarbonate were added to the columns, and the samples were incubated at 47°C for 90 min. Then 40 µL of 50 mM ammonium bicarbonate was added, followed by centrifugation and addition of 1 µL of 0.5 µg/µL trypsin to the eluate, which was incubated at 37°C overnight. The column itself was then eluted using 40 µL of 0.2% formic acid and 40 µL 0.2% formic acid in 50% acetonitrile into a separate tube, which was kept at 4°C. The two eluates were then combined and evaporated before running on LCMSMS. ULC/MS grade solvents were used for all chromatographic steps. Each sample was loaded using split-less nano-Ultra Performance Liquid Chromatography (10 kpsi nanoAcquity; Waters, Milford, MA, USA). The mobile phase was: A) H_2O + 0.1% formic acid and B) acetonitrile + 0.1% formic acid. Desalting of the samples was performed online using a reversed-phase Symmetry C18 trapping column (180 µm internal diameter, 20 mm length, 5 µm particle size; Waters). The peptides were then separated using a T3 HSS nano-column (75 µm internal diameter, 250 mm length, 1.8 µm particle size; Waters) at 0.35 µL/min. Peptides were eluted from the column into the mass spectrometer using the following gradient:

4% to 30%B in 155 min, 35% to 90% B in 5 min, maintained at 90% for 5 min and then back to initial conditions. The nanoUPLC was coupled online through a nanoESI emitter (10 μm tip; New Objective; Woburn, MA, USA) to a quadrupole orbitrap mass spectrometer (Q Exactive HFX, Thermo Scientific) using a FlexIon nanospray apparatus (Proxeon). Data was acquired in data dependent acquisition (DDA) mode, using a Top10 method. MS1 resolution was set to 120,000 (at 200 m/z), mass range of 375-1650 m/z, AGC of $1e^6$ and maximum injection time was set to 60msec. MS2 resolution was set to 15,000, quadrupole isolation 1.7 m/z, AGC of $1e^5$, dynamic exclusion of 45 sec and maximum injection time of 60 msec.

Data analysis for pulldown

The data was analyzed using MaxQuant 1.6.0.16. Human Proteome fasta file downloaded on January 2019, and contaminants were included. The digestion enzyme was set to Trypsin/P with a maximum number of missed cleavages of 2. Oxidation of methionine and N terminal acetylation were included as variable modifications. The "Re-quantify" option was enabled. Carbamidomethyl (C2H3NO) was used as fixed modification on cysteine. Contaminants were included. Peptides were searched with a minimum peptide length of 7 and a maximum peptide mass of 6500 Da. "Second peptides" was enabled, "Dependent peptides" were disabled and the option "Match between run" was enabled with a Match time window of 0.7 min and an alignment window of 20 min. An FDR of 0.01 was used for Protein FDR, PSM FDR and XPSM FDR. Proteins were identified and quantified based on the label-free quantification (LFQ)[4] values reported by MaxQuant. Following MaxQuant analysis, proteins identified only through razor peptides or modified peptides, as well as common contaminants, were removed, and only proteins that gave at least 3 non-zero LFQ intensity values in at least one of the data sets (DMSO/Ibrutinib/3c/3g-treated cells) were retained for analysis. Missing values were then replaced from a normal distribution.

Molecular modeling

We used the structure of ibrutinib covalently bound to BTK (PDB: 5P9J). We "broke" the covalent bond, and rotated the beta-carbon to be co-planar with the amide either in the cys, or trans conformation in an SP2 configuration. Then, fixing the conformation of ibrutinib, we generated 100 constrained conformations of **3a-3k** using RDKit (RDKit: Open-source cheminformatics; http://www.rdkit.org). Once defining these new sets of conformations as new residues, we used the Rosetta Modelling Suite to repack the substitution against the protein and select the optimal combination of conformer and protein side chains.

1. Zanon, P. R. A., Lewald, L. & Hacker, S. M. Protein Modifications Isotopically Labeled Desthiobiotin Azide (isoDTB) Tags Enable Global Profiling of the Bacterial Cysteinome. *Angew. Chemie - Int. Ed.* **59**, 2829–2836 (2020).

2. Sohn, C. H. *et al.* Click Chemistry Facilitates Formation of Reporter Ions and Simplified Synthesis of Amine-Reactive Multiplexed Isobaric Tags for Protein Quantification. *J. Am. Chem. Soc.* **134**, 2672–2680 (2012).

3. Lanning, B. R. *et al.* A road map to evaluate the proteome-wide selectivity of covalent kinase inhibitors. *Nat. ChemBio* **10**, 760–767 (2014).

4. Hein, M. Y., Luber, C. A., Paron, I., Nagaraj, N. & Mann, M. Accurate Proteome-wide Label-free Quantification by Delayed Normalization and Maximal Peptide Ratio Extraction , Termed MaxLFQ. *Mol. Cell. Proteomics* **13**, 2513–2526 (2014).
