# Supplementary material for: Tunable Methacrylamides for Covalent Ligand Directed Release Chemistry
Source: J Am Chem Soc. 2021 Mar 25;143(13):4979–92. doi: 10.1021/jacs.0c10644 (PMC8041284; doi:10.1021/jacs.0c10644)
Supplement: Supplementary file 2 — ja0c10644_si_002.pdf [file ja0c10644_si_002.pdf]

Supplementary Chemistry

1. **Materials and methods**: All reagents and solvents were obtained from commercial suppliers unless otherwise mentioned. Ibr-H (CAS 1022150-12-4) and N4-(3-chloro-4-fluorophenyl)-7-methoxyquinazoline-4,6-diamine (afatinib-amine, CAS no. 179552-75-1) were purchased from BLD pharmatech. Methyl(E)-3-(5-(((1r,3r,5R,7S)-adamantan-2-ylidene)(methoxy)methyl)-3-chloro-2-hydroxyphenyl)acrylate and (5S)-4-(6-fluoro-7-(2-fluoro-6-hydroxyphenyl)-1-(2-isopropyl-4-methylpyridin-3-yl)-2-oxo-1,2-dihydropyrido[2,3-d]pyrimidin-4-yl)-5-methyl-2,3,4,5-tetrahydropyrazin-1-ium (AMG-510 amine) were purchased from Enamine. Deuterated solvents were purchased from Cambridge isotope laboratories and all other reagents are purchased from Sigma Aldrich and used as such without further purification.

   Aluminum-backed silica plates (Merck silica gel 60 F254) were used for thin layer chromatography (TLC) to monitor solution phase reactions. The purification of compounds was carried out on a combi flash chromatography and waters RP-HPLC with Prep C18 column. The ^{1}H-NMR and ^{13}CNMR spectra were recorded using a 400 MHz and 500 MHz Bruker advance spectrometers and were calibrated using residual undeuterated solvent as the internal references ($CDCl_3$: 7.26 ppm; DMSO-d6: 2.50 ppm; D_2O: 4.79 ppm and CD_3OD = 3.31 ppm). Chemical shifts are reported in ppm on a δ scale. The following abbreviations were used to explain NMR peak multiplicities: s = singlet, d = doublet, t = triplet, q = quartet, p = pentet, m = multiplet, br = broad. Most of the Ibrutinib derivatives appeared as a mixture of rotamers. The high-resolution mass spectra were recorded on Waters Xevo G2-XS QTof mass spectrometer using electrospray ionization time-of-flight (ESI-TOF) reflectron experiments.

2. **Abbreviations.** Acetonitrile (ACN), dichloromethane (DCM), N,N'-diisopropylethylamine (DIPEA), N,N'-dimethylformamide (DMF), Ethyl acetate (EtOAc), dimethyl sulfoxide (DMSO), 1-Ethyl-3-(3-dimethylaminopropyl) carbodiimide (EDC), 1-[bis(dimethylamino)methylene]-1H-1,2,3- triazolo[4,5-b]pyridinium3-oxid hexafluorophosphate (HATU), methanol (MeOH), Phosphate buffer saline (PBS), high-performance liquid chromatography (HPLC), trifluoroacetic acid (TFA), 1,4-diazabicyclo[2.2.2]octane (DABCO), diisopropylethyl amine (DIPEA)

3. Synthetic Procedures:

N-benzyl-2-(hydroxymethyl)acrylamide (1n):

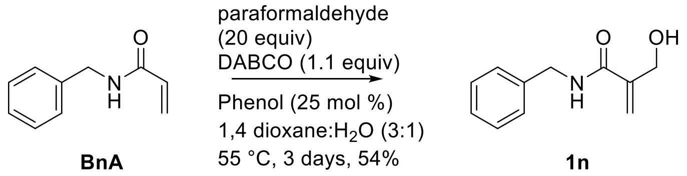

To a stirred solution of acrylamide (644 mg, 4 mmol) in 1,4-dioxane:H_2O (3:1 v/v, 12 mL) were added DABCO (492.8 mg, 4.4 mmol), phenol (87 µL, 1 mmol) and paraformaldehyde (2.4 g, 80 mmol) at 25 °C. The reaction mixture was stirred for 3d at 55°C. After completion of the reaction (as monitored by LC-MS), 1,4-dioxane was evaporated under *vacuo* and the aqueous layer was extracted with EtOAc (3 × 30 mL). The organic layer was evaporated under *vacuo* and the crude product was purified by column chromatography over silica gel using MeOH:EtOAc (1:9)/Pet. ether as eluent to give pure alcohol **1n** as colorless solid in 412 mg (yield = 54%).

^{1}H NMR (500 MHz, $CDCl_3$): δ ppm 3.17 (br. s., 1H), 4.26 (br. s., 2H), 4.41 (m, 2H), 5.43 (br. s., 1H), 5.84 (s, 1H), 7.09 (br. s., 1H), 7.23 (m, 5H): **^{13}C NMR** (126 MHz, $CDCl_3$) δ ppm 43.5, 63.6, 122.0, 127.5, 127.6, 128.7, 137.9, 141.9, 167.4. **ESI-MS** (m/z): calculated for $C_{11}H_{13}NO_2$ $[M+H]^+$: 192.10; found: $[M+H]^+$:192.24. The compound was previously reported.[1]

N-benzyl-2-(bromomethyl)acrylamide (1o):

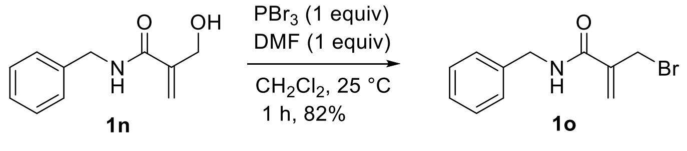

To a stirred solution of alcohol (191 mg, 1 mmol) in CH_2Cl_2 (5 mL), PBr_3 (105 µL, 1.1 mmol) and DMF (77 µL, 1 mmol) were added at 0 °C under N_2 atmosphere. The reaction mixture was stirred at room temperature for 1 h. After completion of the reaction (as monitored by LC-MS), the reaction mixture was quenched with H_2O (4 mL) at 0 °C. The

aqueous layer was extracted with CH_2Cl_2 (3 × 8 mL) evaporated under *vacuo*. The crude product was purified by column chromatography over silica gel using MeOH:EtOAc (1:9)/Pet. ether as eluent to give bromo compound (**1o**) as colorless solid in 207 mg (yield = 82%).

^{1}H NMR (400 MHz, $CDCl_3$): δ 4.24 (s, 2H), 4.56 (d, *J* = 5.7 Hz, 2H), 5.70 (s, 1H), 5.81 (s, 1H), 6.28 (br. s., 1H), 7.35 (m, 5H); **^{13}C NMR** (101 MHz, $CDCl_3$): δ 30.5, 43.9, 122.1, 127.7, 127.8, 128.8, 137.7, 141.7, 166.0. **ESI-MS** (m/z): calculated for $C_{11}H_{13}BrNO$ $[M+H]^+$: 254.02; found: $[M+H]^+$:254.46

N-benzylmethacrylamide (1a)

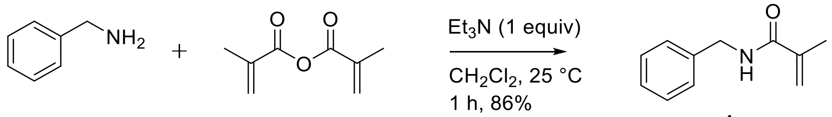

1a

To a stirred solution of benzyl amine (10.6 mg, 0.1 mmol) in anhydrous DCM (0.5 mL), Et_3N (13.9 µL, 0.1 mmol) and methacrylic anhydride (15.4 µL, 0.1 mmol) were added at 25 °C. The reaction mixture was stirred at room temperature for 1 h. After completion of the reaction (as monitored by LC-MS), CH_2Cl_2 was evaporated under *vacuo*. The crude product was purified by preparative HPLC using water:ACN (0.1% TFA) solvent gradient to afford methacrylamide (**1a**) as colorless solid in 15.1 mg (yield = 86 %).

^{1}H NMR (500 MHz, $CDCl_3$): δ 1.99 (s, 3H), 4.51 (d, *J* = 5.6 Hz, 2H), 5.36 (s, 1H), 5.73 (s, 1H), 6.12 (br. s., 1H), 7.27 - 7.38 (m, 5H); **^{13}C NMR** (125 MHz, $CDCl_3$): δ 18.7, 43.8, 119.7, 127.5, 127.8, 128.7, 138.2, 139.9, 168.3. **ESI-MS** (m/z): calculated for $C_{11}H_{14}NO$ $[M+H]^+$: 176.11; found: $[M+H]^+$:176.63**.** The compound was previously reported.[1]

N-benzyl-2-(piperidin-1-ylmethyl)acrylamide (1b):

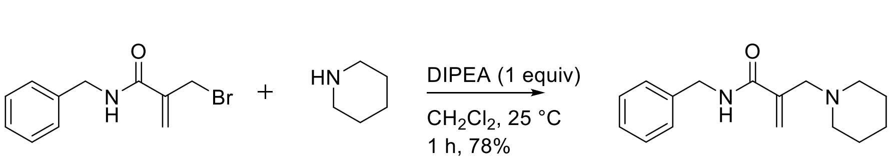

1o **1b**

To a stirred solution of *N*-benzyl-2-(bromomethyl)acrylamide (25.4 mg, 0.1 mmol) in anhydrous DCM (0.5 mL) were added piperidine (9.9 µL, 0.1 mmol), and DIPEA (17.3 µL, 0.1 mmol) at 25 °C. The reaction mixture was stirred at room temperature for one hour.

After completion of the reaction (as monitored by LC-MS), CH_2Cl_2 was evaporated under *vacuo*. The crude product was purified by HPLC using water: ACN (0.1% TFA) solvent gradient to afford colorless solid **1b** in 20.1 mg (yield = 78%).

^{1}H NMR (400 MHz, $CDCl_3$) δ 1.56 (br. s., 1H) 1.91 (br. s., 3H), 2.02 (d, *J* = 11.7 Hz, 2H), 2.81 (t, *J* = 11.3 Hz, 2H), 3.62 (d, *J* = 11.7 Hz, 2H), 4.05 (s, 2H), 4.69 (d, *J* = 5.9 Hz, 3 H), 6.21 (s, 1H), 6.35 (s, 1H), 7.35-7.59 (m, 5H), 7.78 (br. s., 1H); **^{13}C NMR** (100 MHz, $CDCl_3$) δ 21.7, 22.7, 43.9, 53.2, 56.7, 127.6, 127.8, 128.7, 129.4, 133.8, 137.7, 167.0. **ESI-MS** (m/z): calculated for $C_{16}H_{23}N_2O$ $[M+H]^+$: 259.18; found: $[M+H]^+$:258.90

N-benzyl-2-((methyl(phenyl)amino)methyl)acrylamide (1c):

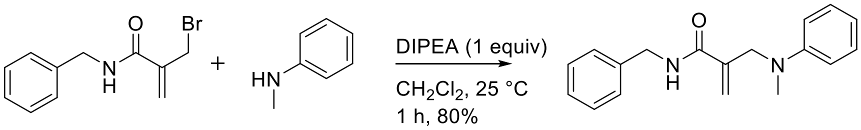

To a stirred solution of N-benzyl-2-(bromomethyl)acrylamide (25.4 mg, 0.1 mmol) in anhydrous DCM (0.5 mL) were added N-methyl aniline (10.7 mg, 0.1 mmol) and DIPEA (17.3 µL, 0.1 mmol) at 25 °C. The reaction mixture was stirred at room temperature for 1 h. After completion of the reaction (as monitored by LC-MS), CH_2Cl_2 was evaporated under *vacuo*. The crude product was purified by preparative HPLC using water:ACN (0.1% TFA) solvent gradient to afford colorless solid **1c** in 22.4 mg (yield = 80%)

^{1}H NMR (500 MHz, $CDCl_3$): δ 3.06 (s, 3H), 4.28 (s, 2H), 4.42 (d, *J* = 5.64 Hz, 2H), 5.76 (s, 1H), 6.03 (s, 1H), 7.13-7.19 (m, 1H), 7.22 (t, *J* = 8.05 Hz, 4H), 7.30 (d, *J* = 7.02 Hz, 1H), 7.32 (s, 1H), 7.34 - 7.39 (m, 2H), 8.63 (br. s., 2H): **^{13}C NMR** (125 MHz, $CDCl_3$) δ 41.7, 43.7, 57.8, 118.2, 124.7, 125.8, 127.6, 127.7, 128.7, 129.8, 136.6, 137.5, 145.0, 160.8, 161.1, 167.3. **ESI-MS** (m/z): calculated for $C_{18}H_{21}N_2O$ $[M+H]^+$: 281.16; found: $[M+H]^+$:281.66.

***N*-benzyl-2-((benzylamino)methyl)acrylamide (1d):**

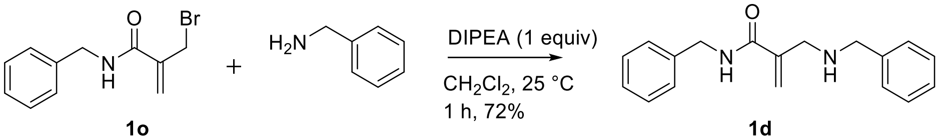

To a stirred solution of *N*-benzyl-2-(bromomethyl)acrylamide (25.4 mg, 0.1 mmol) in anhydrous DCM (0.5 mL) were added benzyl amine (10.7 mg, 0.1 mmol) and DIPEA (17.3 µL, 0.1 mmol) at 25 °C. The reaction mixture was stirred at room temperature for 1 h. After completion of the reaction (as monitored by LC-MS), CH_2Cl_2 was evaporated under *vacuo*. The crude product was purified by preparative HPLC using water:ACN (0.1% TFA) solvent gradient to afford colorless solid **1d** in 20.1 mg (yield =72%).

^{1}H NMR (500 MHz, $CDCl_3$): δ 3.84 (s, 2H), 4.20 (s, 2H), 4.46 (d, J = 5.5 Hz, 2H), 5.86 (s, 1H), 6.00 (s, 1H), 6.98 (br. s., 1H), 7.28-7.39 (m, 6H), 7.40 - 7.49 (m, 4H); **^{13}C NMR** (125 MHz, $CDCl_3$): δ 43.9, 49.3, 51.0, 127.0, 127.9, 128.3, 128.9, 129.4, 129.8, 129.9, 130.1, 133.5, 137.0, 167.4. **ESI-MS** (m/z): calculated for $C_{18}H_{21}N_2O$ $[M+H]^+$: 281.16; found: $[M+H]^+$:281.35

2-(benzylcarbamoyl)-N,N,N-triethylprop-2-en-1-aminium (1e):

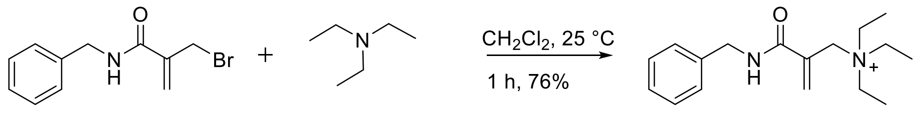

1o 1e

To a stirred solution of *N*-benzyl-2-(bromomethyl)acrylamide (25.4 mg, 0.1 mmol) in anhydrous DCM (0.5 mL), Et_3N (134 µL, 1 mmol) was added at 25 °C. The reaction mixture was stirred at room temperature for 1 h. After completion of the reaction (as monitored by LC-MS), CH_2Cl_2 was evaporated under *vacuo*. The crude product was purified by preparative HPLC using water:ACN (0.1% TFA) solvent gradient to afford colorless solid **1e** in 20.9 mg (yield =76%).

^{1}H NMR (400 MHz, $CDCl_3$) δ ppm 1.23 (t, J = 7.0 Hz, 9H), 3.25 (m, 2H), 3.25 (d, J = 7.3 Hz, 4H), 4.05 (s, 2H), 4.45 (d, J =5.9 Hz, 2H), 5.93 (s, 1H), 6.38 (s, 1H), 7.22 (m, 1H), 7.30 (m, 2H), 7.43 (d, J = 7.3 Hz, 2H), 9.46 (br. s., 1H): **^{13}C NMR** (100 MHz, $CDCl_3$) δ ppm 7.5, 43.8, 52.9, 57.4, 127.2, 128.4, 132.4, 132.8, 138.3, 167.5. **ESI-MS** (m/z): calculated for $C_{17}H_{27}N_2O$ $[M]^+$: 275.21; found: $[M]^+$:275.85.

1-(2-(benzylcarbamoyl)allyl)-4-(dimethylamino)pyridin-1-ium (1f):

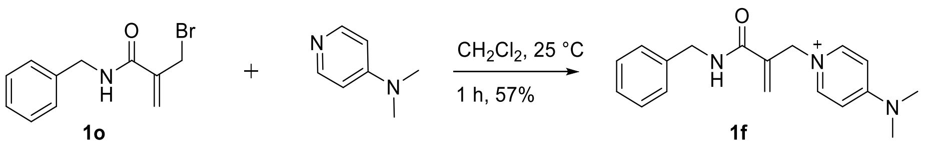

To a stirred solution of *N*-benzyl-2-(bromomethyl)acrylamide (25.4 mg, 0.1 mmol) in anhydrous DCM (0.5 mL) N,N Dimethyl amino pyridine (24.6 mg, 0.2 mmol) was added at 25 °C. The reaction mixture was stirred at room temperature for 1 h. After completion of the reaction (as monitored by LC-MS), CH_2Cl_2 was evaporated under *vacuo*. The crude product was purified by preparative HPLC using water:ACN (0.1% TFA) solvent gradient to afford colorless solid **1f** in 17 mg (yield = 57%).

^{1}H NMR (400 MHz, $CDCl_3$): δ ppm 3.21 (s, 6H), 4.38 (d, *J* = 5.9 Hz, 2H), 5.07 (s, 2H), 5.95 (s, 1H), 6.14 (s, 1H), 6.72 (m, *J* = 7.5 Hz, 2H), 7.23 (m, 5H), 8.20 (br. s., 1H), 8.24 (m, *J* = 7.5 Hz, 2H): **^{13}C NMR** (101 MHz, $CDCl_3$) δ ppm 40.2, 43.5, 59.2, 107.6, 126.1, 127.1, 127.9, 128.4, 137.7, 138.2, 142.5, 156.3, 165.9. **ESI-MS** (m/z): calculated for $C_{18}H_{22}N_3O$ $[M]^+$: 296.18; found: $[M]^+$: 296.25.

N-benzyl-2-(phenoxymethyl)acrylamide (1g):

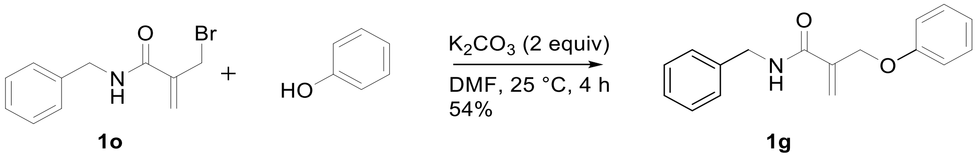

To a stirred solution of phenol (9.4 mg, 0.1 mmol) in anhydrous DMF (0.5 mL) was added K_2CO_3 (27.6 mg, 0.2 mmol) at 25 °C. After stirring for 5 min, N-benzyl-2-(bromomethyl)acrylamide (25.4 mg, 0.1 mmol) was added at 25 °C under N_2 atmosphere. The reaction mixture was stirred at room temperature for 4 h under N_2 atmosphere and quenched with H_2O (2 mL). The aqueous layer was extracted with EtOAc (3 × 3 mL). The organic layer was washed with brine and evaporated under *vacuo*. The crude product was purified by preparative HPLC using water:ACN (0.1% TFA) solvent gradient to afford colorless solid **1g** in 14.4 mg (yield = 53.9%).

^{1}H NMR (500 MHz, $CDCl_3$): δ 4.56 (d, *J* = 5.6 Hz, 2H) 4.82 (s, 3H) 5.74 (s, 1H) 6.10 (s, 1H) 6.67 (br. s., 1H) 6.92 (d, *J* = 8.0 Hz, 2H) 7.00 (t, *J* = 7.3 Hz, 1H) 7.26-7.29 (m, 1H) 7.29-7.33 (m, 4H) 7.33-7.39 (m, 2H); **^{13}C NMR** (125 MHz, $CDCl_3$): δ 43.7, 67.9, 114.9,

121.6, 123.1, 127.6, 127.7, 128.7, 129.6, 137.9, 139.2, 157.7, 166.3. **ESI-MS** (m/z): calculated for $C_{17}H_{18}NO_2$ $[M+H]^+$: 268.13; found: $[M+H]^+$:268.63.

N-benzyl-2-((4-nitrophenoxy)methyl)acrylamide (1h):

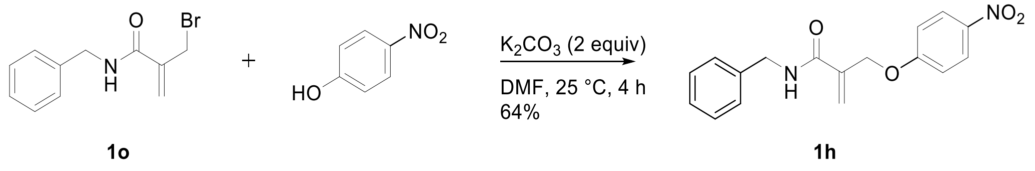

To a stirred solution of 4-nitrophenol (13.9 mg, 0.1 mmol) in anhydrous DMF (0.5 mL), K_2CO_3 (27.6 mg, 0.2 mmol) was added at 25 °C under N_2 atmosphere. After stirring for 5 min, N-benzyl-2-(bromomethyl)acrylamide (25.4 mg, 0.1 mmol) was added to the reaction mixture. The reaction mixture was stirred at room temperature for 4 h under N_2 atmosphere and quenched with H_2O (2 mL). The aqueous layer was extracted with EtOAc (3 × 3 mL). The combined organic layer was washed with brine and evaporated under *vacuo*. The crude product was purified by HPLC using water:ACN (0.1% TFA) solvent gradient to afford colorless solid **1h** in 20.2 mg (yield = 65%).

^{1}H NMR (500 MHz, $CDCl_3$): δ 4.54 (d, J = 5.8 Hz, 2H), 4.92 (s, 2H), 5.76 (s, 1H), 5.96 (s, 1H), 6.46 (br. s., 1H), 7.00 (d, J = 9.2 Hz, 2H), 7.29-7.40 (m, 5H), 8.19 (d, J = 9.2 Hz, 2H): **^{13}C NMR** (125 MHz, $CDCl_3$): δ 43.7, 67.7, 114.8, 121.2, 125.9, 127.7, 128.8, 137.6, 139.0, 141.9, 162.9, 166.1. **ESI-MS** (m/z): calculated for $C_{17}H_{17}N_2O_4$ $[M+H]^+$: 313.12; found: $[M+H]^+$:313.52.

N-benzyl-2-(((2-oxo-2H-chromen-7-yl)oxy)methyl)acrylamide (1i):

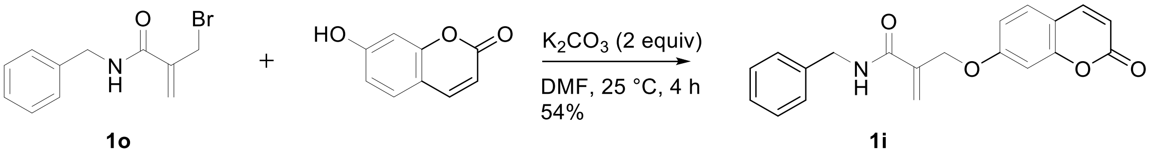

To a stirred solution of 7-hydroxy-2H-chromen-2-one (16.2 mg, 0.1 mmol) in anhydrous DMF (0.5 mL) was added K_2CO_3 (27.6 mg, 0.2 mmol) at 25 °C. After stirring for 5 min, N-benzyl-2-(bromomethyl)acrylamide (25.4 mg, 0.1 mmol) was added at 25 °C under N_2 atmosphere. The reaction mixture was stirred at room temperature for 4 h under N_2 atmosphere and quenched with H_2O (2 mL). The aqueous layer was extracted with EtOAc

(3 × 3 mL). The organic layer was washed with brine and evaporated under *vacuo*. The crude product was purified by preparative HPLC using water:ACN (0.1% TFA) solvent gradient to afford colorless solid **1i** in 18.1 mg (54% yield).

^{1}H NMR (500 MHz, $CDCl_3$): δ 4.54 (d, *J* = 5.6 Hz, 2H), 4.88 (s, 2H), 5.75 (s, 1H), 6.00 (s, H), 6.24 (d, *J* = 9.5 Hz, 1H), 6.58 (br. s., 1H), 6.83 (d, *J* = 2.2 Hz, 1H), 6.85-6.88 (m, 1H), 7.28-7.32 (m, 3H), 7.34 (d, *J* = 7.2 Hz, 2H), 7.37 (d, *J* = 8.7 Hz, 1H), 7.62 (d, *J* = 9.5 Hz, 1H); **^{13}C NMR** (125 MHz, $CDCl_3$) δ 43.6, 67.8, 102.2, 112.6, 113.4, 121.8, 127.6, 127.7, 128.7, 128.8,137.7, 139.0, 143.2, 155.6, 161.0, 166.0. **ESI-MS** (m/z): calculated for $C_{20}H_{18}NO_4$ $[M+H]^+$: 336.12; found: $[M+H]^+$:336.03.

N-benzyl-2-((benzyloxy)methyl)acrylamide (1j):

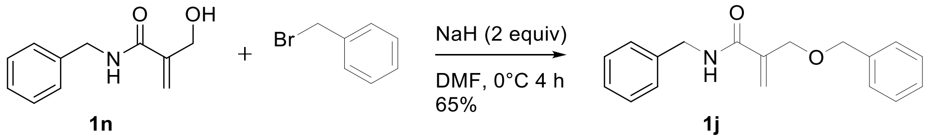

To a stirred solution of alcohol **1k** (19.1 mg, 0.1 mmol) in anhydrous DMF (0.5 mL) was added NaH (8 mg (60% in mineral oil), 0.2 mmol)) at 0 °C under N_2 atmosphere. After stirring for 5 min, benzyl bromide (13 µL, 0.11 mmol) was added. The reaction mixture was stirred at room temperature for 4 h under N_2 atmosphere and then quenched with H_2O (2 mL) at 0 °C. The aqueous layer was extracted with EtOAc (3 × 3 mL). The organic layer was washed with brine and evaporated under *vacuo*. The crude product was purified by preparative HPLC using water:ACN (0.1% TFA) solvent gradient to afford colorless solid **1j** in 18.4 mg (65.4 % yield).

^{1}H NMR (500 MHz, $CDCl_3$): δ 4.31 (s, 2H), 4.46-4.58 (m, 4H), 5.61 (s, 1H), 6.30 (d, *J* = 1.2 Hz, 1H), 7.18-7.25 (m, 2H), 7.25-7.38 (m, 10H). ^{13}C NMR (125 MHz, $CDCl_3$):δ 43.6, 70.5, 72.0, 125.7, 127.4, 127.8, 128.0, 128.0, 128.6, 128.7, 137.0, 138.1, 138.6, 166.3. **ESI-MS** (m/z): calculated for $C_{18}H_{20}NO_2$ $[M+H]^+$: 282.15; found: $[M+H]^+$:282.74. The compound was previously reported.[1]

2-(benzylcarbamoyl)allyl (4-nitrophenyl) carbonate (1k):

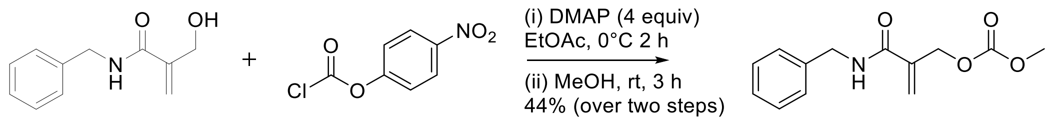

1n **1k**

To a stirred solution of N-benzyl-2-(hydroxymethyl)acrylamide (19.2 mg, 0.1 mmol) in anhydrous ethyl acetate (1 mL) were added 4-nitrophenyl chloroformate (80.4 mg, 0.4 mmol) and 4-Dimethylaminopyridine (25.4 mg, 0.4 mmol) at 25 °C. The reaction mixture was stirred at room temperature for 1 h. After completion of the reaction as monitored by LC-MS, the reaction mixture is quenched with 0.1 N HCl (2 mL) and the aqueous layer was extracted with EtOAc (3 × 3 mL). The organic layer was washed with brine and evaporated under *vacuo*. The crude product was dissolved in methanol (0.5 mL) and stirred for 3 h. After completion of the reaction, methanol was evaporated and the crude product was purified by preparative HPLC using water:ACN (0.1% TFA) solvent gradient to afford colorless solid **1k** in 11.1 mg (yield = 44%).

^{1}H NMR (500 MHz, $CDCl_3$): δ 3.80 (s, 3H), 4.53 (d, *J* = 5.4 Hz, 2H), 4.93 (s, 2H), 5.70 (s, 1H), 5.98 (s, 1H), 6.37 (br. s., 1H), 7.31 (m, 3H), 7.36 (m, 2H): **^{13}C NMR** (125 MHz, $CDCl_3$) δ 43.8, 55.1, 66.7, 123.0, 127.6, 127.8, 128.8, 137.8, 138.8, 155.4, 165.8. **ESI-MS** (m/z): calculated for $C_{13}H_{16}NO_4$ $[M+H]^+$: 250.11; found: $[M+H]^+$:250.65

2-(benzylcarbamoyl)allyl benzoate (1l):

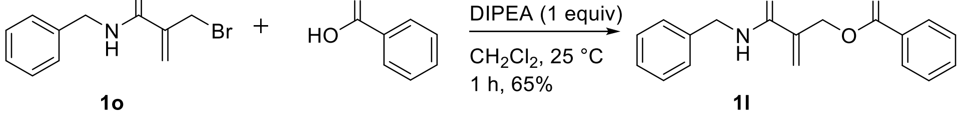

1o **1l**

To a stirred solution of benzoic acid (12.2 mg 0.1 mmol) in anhydrous DCM (0.5 mL) DIPEA (17.3 µL, 0.1 mmol) and N-benzyl-2-(bromomethyl)acrylamide (25.4 mg, 0.1 mmol) were added at 25 °C. The reaction mixture was stirred at room temperature for 1 h. After completion of the reaction as monitored by LC-MS, CH_2Cl_2 was evaporated under *vacuo*. The crude product was purified by HPLC using water:ACN (0.1% TFA) solvent gradient to afford colorless solid **1l** in 19.4 mg (65.7% yield).

^{1}H NMR (500 MHz, $CDCl_3$):δ2.47 (br. s., 1H), 4.55 (s, 2H), 5.12 (s, 2H), 5.74 (s, 1H), 6.05 (s, 1H), 6.56 (br. s., 1H), 7.30 (s, 3H), 7.43 (t, *J* = 7.7 Hz, 2H), 7.58 (t, *J* = 7.4 Hz, 1H), 7.99 (d, *J* = 7.7 Hz, 1H); **^{13}C NMR** (125 MHz, $CDCl_3$) δ 43.7, 63.8, 123.2, 127.5, 127.7, 128.4, 128.7, 129.5, 129.6, 133.2, 137.8, 139.1, 166.0. **ESI-MS** (m/z): calculated for $C_{18}H_{18}NO_3$ $[M+H]^+$: 296.13; found: $[M+H]^+$:296.50.

2-(benzylcarbamoyl)allyl (4-nitrophenyl) carbonate (1m):

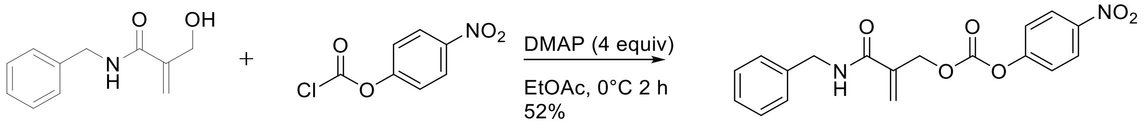

To a stirred solution of N-benzyl-2-(hydroxymethyl)acrylamide (19.2 mg, 0.1 mmol) in anhydrous ethyl acetate (1 mL) were added 4-nitrophenyl chloroformate (80.4 mg, 0.4 mmol) and 4-Dimethylaminopyridine (25.4 mg, 0.4 mmol) at 25 °C. The reaction mixture was stirred at room temperature for 1 h. After completion of the reaction as monitored by LC-MS, the reaction mixture was quenched with 0.1 N HCl (2 mL) and the aqueous layer was extracted with EtOAc (3 × 3 mL). The organic layer was washed with brine and evaporated under *vacuo*. The crude product was purified by HPLC using water:ACN (0.1% TFA) solvent gradient to afford colorless solid **1m** in 18.6 mg (52.2% yield).

^{1}H NMR (400 MHz, $CDCl_3$) δ 4.66 (d, *J* = 5.5 Hz, 2H) 5.17 (s, 2H) 5.89 (s, 1H) 6.07 (s, 1H) 6.41 (br. s., 1H) 7.29-7.54 (m, 7H) 8.38 (d, *J* = 9.2 Hz, 2H); **^{13}C NMR** (100 MHz, $CDCl_3$) δ 43.9, 67.9, 121.7, 123.0, 125.3, 127.8, 127.9, 128.9, 138.4, 152.1, 155.3, 165.7. **ESI-MS** (m/z): calculated for $C_{18}H_{17}N_2O_6$ $[M+H]^+$: 357.11; found: $[M+H]^+$:356.86.

N-(hex-5-yn-1-yl)-2-iodoacetamide (IA-alkyne)

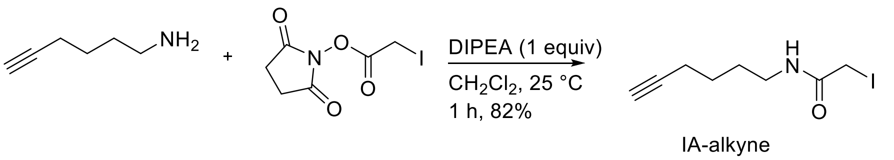

To a stirred solution of 2,5-dioxopyrrolidin-1-yl 2-iodoacetate (28.3 mg, 0.1 mmol) in anhydrous DCM (0.5 mL) were added hex-5-yn-1-amine.hydrochloride (13.4 mg, 0.1

mmol) and DIPEA (17.3 µL, 0.1 mmol) at 25 °C. The reaction mixture was stirred at room temperature for 1 h. After completion of the reaction (as monitored by LC-MS), CH_2Cl_2 was evaporated under *vacuo*. The crude product was purified by preparative HPLC using water:ACN (0.1% TFA) solvent gradient to afford colorless solid **IA-alkyne** in 21.7 mg (yield =82%). The compound was previously reported.[2]

^{1}H NMR (500 MHz, $CDCl_3$) δ 1.54-1.62 (m, 2H), 1.66-1.70 (m, 2H), 1.98 (t, *J* = 2.6 Hz, 1 H), 2.25 (td, *J* = 6.8, 2.6 Hz, 2H), 3.32 (q, *J* = 6.8 Hz, 2H), 3.71 (s, 2H), 6.09 (br. s., 1H); **^{13}C NMR** (125 MHz, $CDCl_3$) -0.4, 18.1, 25.5, 28.3, 39.9, 68.9, 83.9, 166.6. **ESI-MS** (m/z): calculated for $C_8H_{13}INO$ $[M+H]^+$: 266.00; found: $[M+H]^+$: 266.42.

2-(((2-oxo-2H-chromen-7-yl)oxy)methyl)acrylic acid (2d)

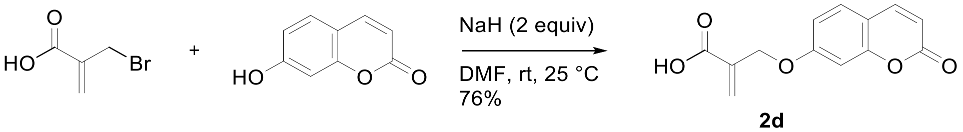

To a stirred solution of 7-hydroxy coumarin (92 mg, 0.56 mmol) in DMF (3 mL) were added NaH (44.8 mg, 1.12 mmol) and bromo methacrylic acid (90.1 mg, 0.56 mmol) and the reaction mixture stirred at 25°C for 2 h under nitrogen atmosphere. After completion of the reaction, monitored by TLC, the reaction mixture was quenched with water and extracted with EtOAc (3x10 mL). The combined organic layer was washed with brine solution (3X15) and the organic layer was dried in Na_2SO_4 and then evaporated under reduced pressure to give the crude acid which was purified using silica gel chromatography using hexane: ethyl acetate as eluent to obtain colorless solid **2d** in 105 mg (yield = 76%)

^{1}H NMR (500 MHz, METHANOL-*d*$_4$) δ ppm 6.00 (d, *J* = 1.4 Hz, 1 H), 6.26 (d, *J* = 9.5 Hz, 1H), 6.39 (d, *J* = 1.0 Hz, 1H), 6.95 (d, *J* = 2.3 Hz, 1H), 6.99 (dd, *J* = 8.6, 2.4 Hz, 1H), 7.56 (d, *J* = 8.7 Hz, 1H), 7.89 (d, *J* = 9.5 Hz, 1H): **^{13}C NMR** (125 MHz, $CDCl_3$) δ 66.5, 102.0, 112.9, 113.3, 127.9, 128.9, 135.1, 143.5, 155.8, 161.3, 163.0, 168.0. **HR-MS** (m/z): Calculated for $C_{13}H_{11}O_5$ $[M+H]^+$: 247.06; Found: $[M+H]^+$: 247.75

N-(but-3-yn-1-yl)-2-(((2-oxo-2H-chromen-7-yl)oxy)methyl)acrylamide (2a):

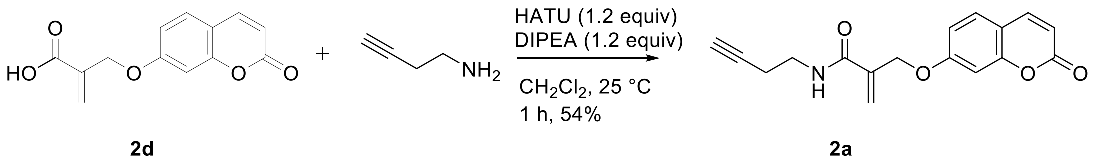

To a solution of carboxylic acid (24.7 mg, 0.1 mmol) in CH_2Cl_2 (1 mL), HATU (45.6 mg, 0.12 mmol), DIPEA (21.5 µL, 0.12 mmol) and but-3-yn-1-amine hydrochloride (12.6 mg, 0.12 mmol) were added at 25 °C. The reaction mixture was stirred at room temperature for 1 h. After completion of the reaction (as monitored by LC-MS), water was added. the aqueous layer was extracted with CH_2Cl_2 (3 × 3 mL). The organic layer was evaporated under *vacuo* and the crude product was purified by preparative HPLC using water:ACN (0.1% TFA) solvent gradient to afford colorless solid **2a** in 16.14 mg (yield = 54%).

^{1}H NMR (400 MHz, $CDCl_3$): δ 2.01 (t, *J* = 2.5 Hz, 1H), 2.48 (td, *J* = 6.4, 2.6 Hz, 2H), 3.52 (q, *J* = 6.3 Hz, 2H), 4.86 (s, 2H), 5.76 (s, 1H), 6.03 (s, 1H), 6.27 (d, *J* = 9.5 Hz, 1H), 6.57 (br. s., 1H), 6.85-6.95 (m, 2H), 7.40 (d, *J* = 8.4 Hz, 1H), 7.64 (d, *J* = 9.5 Hz, 1H); **^{13}C NMR** (100 MHz, $CDCl_3$): δ 19.3, 38.0, 67.9, 70.3, 81.3, 102.3, 112.7, 113.1, 113.6, 122.3, 128.9, 138.8, 143.3, 155.7, 161.0, 166.2. **HR-MS** (m/z): Calculated for $C_{17}H_{15}NNaO_4$ $[M+Na]^+$: 320.0899; Found: $[M+Na]^+$: 320.0896.

2-(but-3-yn-1-ylcarbamoyl)allyl benzoate (2b)

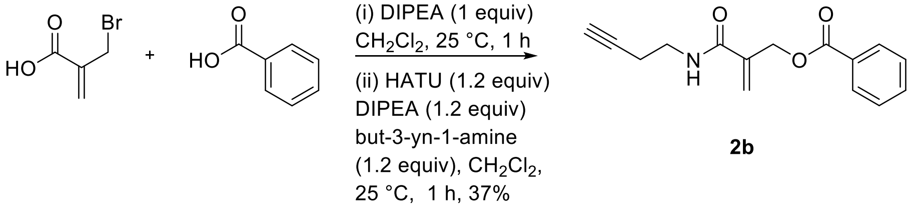

To a stirred solution of benzoic acid (10.7 mg, 0.1 mmol) in anhydrous DCM (1 mL), DIPEA (17.9 µL, 0.1 mmol) and 2-(bromomethyl)acrylic acid (16.1 mg, 0.1 mmol) were added at 25 °C. The reaction mixture was stirred at room temperature for 1 h. After completion of the reaction (as monitored by LC-MS), CH_2Cl_2 was evaporated under *vacuo* to obtain the crude carboxylic acid.

To a solution of crude carboxylic acid in CH_2Cl_2 (0.5 mL), HATU (45.6 mg, 0.12 mmol), DIPEA (21.5 µL, 0.12 mmol) and but-3-yn-1-amine hydro chloride (12.6 mg, 0.12 mmol)

were added at 25 °C. The reaction mixture was stirred at room temperature for 1 h. After completion of the reaction (as monitored by LC-MS), water was added. the aqueous layer was extracted with CH_2Cl_2 (3 × 3 mL). The organic layer was evaporated under *vacuo* and the crude product was purified by HPLC using water:ACN (0.1% TFA) solvent gradient to afford colorless solid **2b** in 9.8 mg (37% yield).

^{1}H NMR (500 MHz, $CDCl_3$): δ 1.92 (t, *J* = 2.6 Hz, 1H), 2.47 (td, *J* = 6.3, 2.6 Hz, 2H), 3.52 (q, *J* = 6.2 Hz, 2H), 5.11 (s, 2H), 5.76 (s, 1H), 6.07 (s, 1H), 6.52 (br. s., 1H), 7.42-7.50 (m, 2H), 7.55-7.64 (m, 1H), 8.07 (dd, *J* = 8.3, 1.1 Hz, 2H); **^{13}C NMR** (125 MHz, $CDCl_3$) δ 19.3, 38.1, 63.8, 70.2, 81.3, 123.4, 128.5, 129.7, 133.3, 139.1, 166.2. **HR-MS** (m/z): Calculated for $C_{15}H_{15}NNaO_3$ $[M+Na]^+$: 280.0950; Found: $[M+Na]^+$: 280.0950.

N-(but-3-yn-1-yl)-2-((methyl(phenyl)amino)methyl)acrylamide (2c)

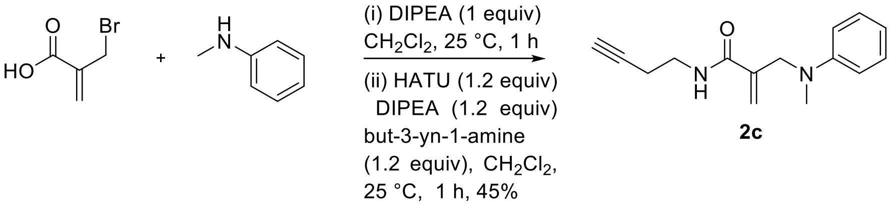

To a stirred solution of N-methyl aniline (10.3 mg, 0.1 mmol) in anhydrous DCM (1 mL), DIPEA (17.9 µL, 0.1 mmol) and 2-(bromomethyl)acrylic acid (16.1 mg, 0.1 mmol) were added at 25 °C. The reaction mixture was stirred at room temperature for 1 h. After completion of the reaction (as monitored by LC-MS), CH_2Cl_2 was evaporated under *vacuo* to obtain the crude carboxylic acid.

To a solution of crude carboxylic acid in CH_2Cl_2 (1 mL), HATU (45.6 mg, 0.12 mmol), DIPEA (21.5 µL, 0.12 mmol) and but-3-yn-1-amine hydro chloride (12.6 mg, 0.12 mmol) were added at 25 °C. The reaction mixture was stirred at room temperature for 1 h. After completion of the reaction (as monitored by LC-MS), water was added. the aqueous layer was extracted with CH_2Cl_2 (3 × 3 mL). The organic layer was evaporated under *vacuo* and the crude product was purified by HPLC using water:ACN (0.1% TFA) solvent gradient to afford colorless solid **2c** in 10.9 mg (45.0% yield over two steps).

^{1}H NMR (400 MHz, $CDCl_3$) δ 1.95 (t, *J* = 2.6 Hz, 1 H), 2.31-2.38 (m, 2H), 3.37 (q, *J* = 6.2 Hz, 2H), 4.27 (s, 2H), 5.92 (s, 1H), 6.06 (s, 1H), 6.96 (br. s., 1H), 7.16-7.23 (m, 1H), 7.33 (d, *J* = 7.9 Hz, 2H), 7.40 (t, *J* = 7.9 Hz, 2H); **^{13}C NMR** (125 MHz, $CDCl_3$) δ 19.1, 38.2, 43.0, 57.9, 70.3, 81.0, 120.0, 127.2, 128.0, 130.0, 135.3, 143.1, 167.0. **HR-MS** (m/z): Calculated for $C_{15}H_{18}N_2NaO$ $[M+Na]^+$: 265.1317; Found: $[M+Na]^+$: 265.1319.

(R)-3-(4-phenoxyphenyl)-1-(piperidin-3-yl)-1H-pyrazolo[3,4-d]pyrimidin-4-amine (Ibrutinib):

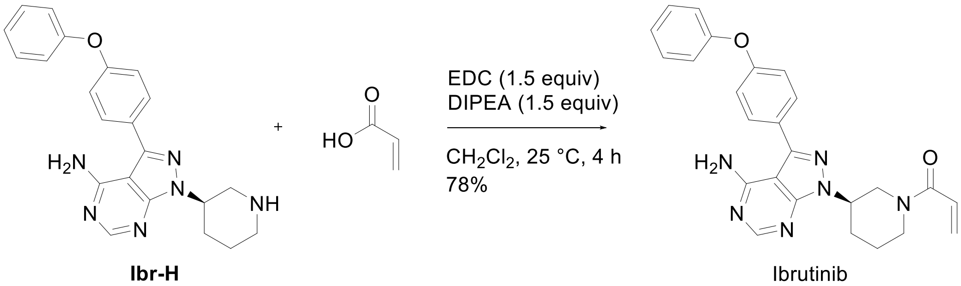

To a stirred solution of acrylic acid (1.02 mL, 15 mmol) in anhydrous CH_2Cl_2 (50 mL), N-(3-dimethylaminopropyl)-N′-ethylcarbodiimide hydrochloride (EDC.HCl) (2.88 g, 15 mmol), N,N-Diisopropylethylamine (2.60 mL, 15 mmol) and amine (3.87 g, 10 mmol) were added at 0 °C under N_2 atmosphere. The reaction mixture was stirred at room temperature for 4 h. After completion (as monitored by LC-MS), of the reaction, H_2O (30 mL) was added. The organic layer was extracted with CH_2Cl_2 (3 x 50 mL) and evaporated under *vacuo.* The crude product was purified by column chromatography over silica gel using EtOAc:MeOH (9:1)/Pet. ether as eluent to give pure Ibrutinib as colorless solid 3.47 g (yield = 78%). This compound is reported in the literature.[3]

^{1}H NMR (500 MHz, CD_3OD) (as a mixture of rotamers) δ 1.67-1.78 (m, 1H), 2.04-2.15 (m, 1H), 2.26 (dd, *J* = 12.7, 3.6 Hz, 1H), 2.33-2.44 (m, 1H), 3.26 (t, J = 10.4, 0.4H) (t, *J* = 11.3 Hz, 0.6H), 3.58 (dd, *J* = 12.2, 10.2 Hz, 0.6H), 3.88 (m, 0.4H), 4.05 (d, *J* = 13.6 Hz, 0.6H), 4.23 (m, 0.8H), 4.56 (d, *J* = 12.0 Hz, 0.6H), 4.88 (m, 1H), 5.76 (d, *J* = 10.7 Hz, 1H), 6.11-6.23 (m, 1H), 6.81 (dd, *J* = 16.7, 10.7 Hz, 1H), 7.10 (d, *J* = 7.7 Hz, 2H), 7.15 (d, *J* = 8.7 Hz, 2H), 7.18 (t, *J* = 7.4 Hz, 1H), 7.41 (t, *J*=8.0 Hz, 2H), 7.68 (d, *J* = 8.7 Hz, 2H), 8.37-8.46 (m, 1H); **^{13}C NMR** (126 MHz, CD_3OD) (as a mixture of rotamers) δ 24.2, 25.7, 30.5, 30.7, 43.5, 47.1, 47.1, 50.9, 54.4, 55.0, 98.1, 120.0, 120.7, 125.2, 127.1, 128.9, 131.1,

131.3, 147.9, 148.3, 153.3, 154.5, 157.7, 160.5, 167.9. **HR-MS** (m/z): Calculated for $C_{25}H_{24}N_6O_2$ $[M+H]^+$: 441.2039; Found: $[M+H]^+$: 441.2030

(R)-1-(3-(4-amino-3-(4-phenoxyphenyl)-1H-pyrazolo[3,4-d]pyrimidin-1-yl)piperidin-1-yl)-2-(hydroxymethyl)prop-2-en-1-one (3m):

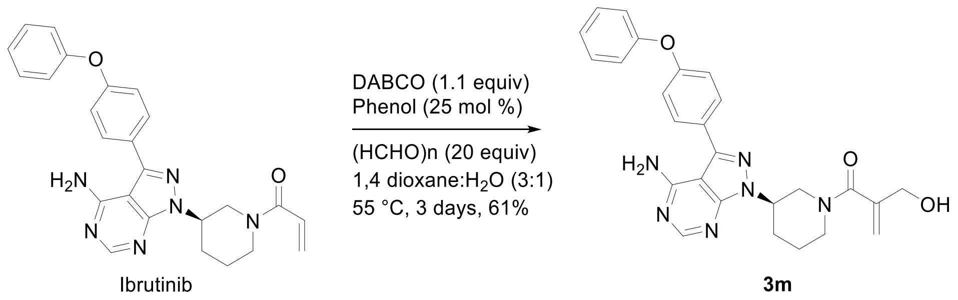

To a stirred solution of acrylamide (440 mg, 1 mmol) in 1,4-dioxane:H_2O (3:1 v/v, 12 mL) were added DABCO (123.2 mg, 1.1 mmol), phenol (21.8 µL, 0.25 mmol) and paraformaldehyde (600 mg, 20 mmol) at 25 °C. The reaction mixture was stirred at room temperature for 3d. After completion of the reaction (as monitored by LC-MS), 1,4 dioxane was evaporated under *vacuo* and the aqueous layer was extracted with EtOAc (3 × 30 mL). The combined organic layer was evaporated under *vacuo* and the crude product was purified by column chromatography over silica gel using EtOAc:MeOH (9:1)/Pet. ether as eluent to give pure alcohol as colorless solid **3m** in 286.7 mg (yield = 61%).

^{1}H NMR (500 MHz, CD_3OD) (as a mixture of rotamers) δ 1.73 (d, J = 13.5 Hz, 1H), 2.02 (d, J = 12.9 Hz, 1H), 2.20 (m, 1H), 2.34 (br. s., 1H), 2.99 (br. s., 0.5H), 3.37 (br. s., 0.5H) 3.57-3.73 (br. s., 1H), 4.07 (d, J = 11.1 Hz, 1 H), 4.24 (br. s., 3H), 4.44-4.53 (bs, 1H), 5.18-5.24 (br. s., 1H), 5.38-5.46 (br. s., 1H), 7.13 (m, 5H), 7.39 (t, J = 8.0 Hz, 2H), 7.66 (d, J = 8.3 Hz, 2H), 8.23 (s, 1H): **^{13}C NMR** (100 MHz, DMSO-d_6): (as a mixture of rotamers) δ 25.1, 30.4, 46.0, 47.7, 53.0 , 62.9, 98.4, 115.1, 120.0, 120.1, 125.2, 128.2, 131.1, 131.3, 144.8, 144.9, 154.3, 156.6, 156.9, 158.5, 159.0, 170.8. **HR-MS** (m/z): Calculated for $C_{26}H_{27}N_6O_3$ $[M+H]^+$: 471.2145; Found: $[M+H]^+$: 471.2142

(R)-1-(3-(4-amino-3-(4-phenoxyphenyl)-1H-pyrazolo[3,4-d]pyrimidin-1-yl)piperidin-1-yl)-2-(bromomethyl)prop-2-en-1-one (3n)

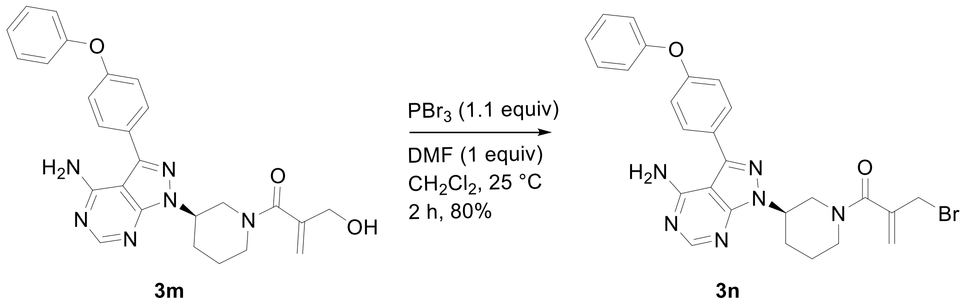

To a stirred solution of alcohol **3m** (235 mg, 0.5 mmol) in CH_2Cl_2 (5 mL) were added PBr_3 (52.5 μL, 0.55 mmol) and DMF (37.5 μL, 0.5 mmol) at 0 °C under N_2 atmosphere. The reaction mixture stirred at 25 °C for 1 h under nitrogen atmosphere and quenched with H_2O (5 mL) at 0 °C. The aqueous layer was extracted with CH_2Cl_2 (3 × 8 mL) and evaporated under *vacuo*. The crude product was purified by column chromatography over silica gel using MeOH:EtOAc (1:9)/Pet. ether as eluent to give pure bromo compound as colorless solid **3n** in 213 mg (yield = 80%).

^{1}H NMR (500 MHz, CD_3OD) (as a mixture of rotamers) δ 1.85 (br. s., 1H), 2.11 (br. s., 1H), 2.29 (d, *J*=12.8 Hz, 1H), 2.35-2.49 (m, 1H), 3.05 (br. s. 0.3H), 3.43-3.60 (m, 0.6H), 3.68-3.80 (m, 1H), 3.80-3.90 (m, 1H), 4.05-4.13 (m, 0.6H), 4.25 (m, 2H), 4.52 (br. s., 1.4 H), 5.02 (br. s., 1H), 5.29-5.40 (m, 1H), 5.68 (br. s., 1H), 7.13 (d, *J* = 8.7 Hz, 2H), 7.16-7.26 (m, 3H), 7.44 (t, *J* = 8.0 Hz, 2H), 7.72 (d, *J* = 8.5 Hz, 2H), 8.43 (br. s., 1H); **^{13}C NMR** (125 MHz, CD_3OD) (as a mixture of rotamers) δ 24.4, 29.5, 41.5, 45.2, 53.2, 62.5, 97.8, 113.6, 118.6, 119.1, 123.7, 127.5, 129.7, 129.9, 144.5, 144.7, 153.7, 155.3, 156.5, 158.5, 158.5, 170.5. **HR-MS** (m/z): Calculated for $C_{26}H_{26}BrN_6O_2$ $[M+H]^+$: 533.1301; Found: $[M+H]^+$: 533.1311.

(R)-1-(3-(4-amino-3-(4-phenoxyphenyl)-1H-pyrazolo[3,4-d]pyrimidin-1-yl)piperidin-1-yl)-2-methylprop-2-en-1-one (3a)

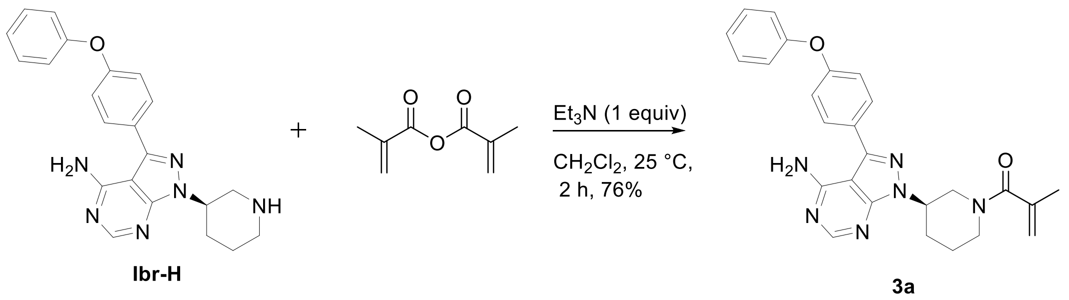

To a stirred solution of Ibrutinib amine (38.7 mg, 0.1 mmol) in anhydrous DCM (0.5 mL) were added Et_3N (13.9 µL, 0.1 mmol) and methacrylic anhydride (15.4 µL, 0.1 mmol) at 25 °C. The reaction mixture was stirred at room temperature for 1 h. After completion of the reaction (as monitored by LC-MS), CH_2Cl_2 was evaporated under *vacuo*. The crude product was purified by preparative HPLC using water:ACN (0.1% TFA) solvent gradient to afford colorless solid **3a** in 36.1 mg (yield = 76%).

^{1}H NMR (500 MHz, $CDCl_3$) (as a mixture of rotamers) δ 1.66-1.79 (m, 1H), 1.98 (s, 3H), 2.06 (d, J = 14 Hz, 1H), 2.22-2.32 (m, 1H), 2.38 (d, J = 11 Hz, 1H), 3.24 (br. s., 1H), 3.54 (br. s., 1H), 4.03 (br. s., 1H), 4.69 (br. s., 1H), 4.91 (br. s., 1H), 5.12 (s, 1H), 5.23 (br. s., 1H), 6.34 (br. s., 1H), 7.12 (d, J = 8 Hz, 2H), 7.19 (m, J = 8 Hz, 2H), 7.23 (t, J = 7 Hz, 1H), 7.44 (t, J = 8 Hz, 2H), 7.60 (m, J = 8 Hz, 2H), 8.28 (s, 1H), 11.55 (br. s., 1H); **^{13}C NMR** (125 MHz, $CDCl_3$) (as a mixture of rotamers) δ 20.5, 24.9, 30.2, 45.3, 46.9, 53.4, 97.1, 114.7, 115.9, 117.0, 119.2, 119.9, 124.6, 125.1, 129.7, 130.1, 140.0, 145.8, 147.0, 151.5, 153.6, 155.7, 159.8, 163.4, 163.7, 171.8. **HR-MS** (m/z): Calculated for $C_{26}H_{27}N_6O_2$ $[M+H]^+$: 455.2195; Found: $[M+H]^+$: 455.2190

(R)-1-(3-(4-amino-3-(4-phenoxyphenyl)-1H-pyrazolo[3,4-d]pyrimidin-1-yl)piperidin-1-yl)-2-((diethylamino)methyl)prop-2-en-1-one (3b):

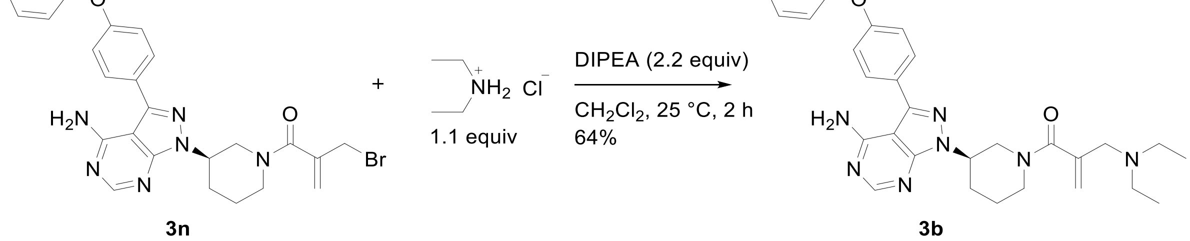

To a stirred solution of bromo compound **3n** (26.5 mg, 0.05 mmol) in anhydrous DCM (1 mL) were added diethylamino hydrochloride (5.9 mg, 0.055 mmol) and DIPEA (19.1 μL, 0.11 mmol) at 25 °C. The reaction mixture was stirred at room temperature for one hour. After completion of the reaction (as monitored by LC-MS, CH_2Cl_2 was evaporated under *vacuo*. The crude product was purified by preparative HPLC using water: ACN (0.1% TFA) solvent gradient to afford colorless solid **3b** in 16.8 mg (yield = 64%).

^{1}H NMR (500 MHz, DMSO-*d*$_6$) (as a mixture of rotamers) δ 1.22 (t, *J* = 7.08 Hz, 6H), 1.71 (br. s., 1H), 1.95 (d, *J* = 12.65 Hz, 1H), 2.10-2.22 (m, 1H), 2.24-2.40 (m, 1H), 2.96 (br. s., 0.5H), 3.13 (br. s., 4H), 3.29 (br. s., 1H), 3.66 (br. s., 0.5H), 3.95 (br. s., 2H), 4.19 (br. s., H), 4.32 - 4.49 (br. s., 1H), 4.80-4.91 (m, 1H), 5.80 (br. s., 1H), 5.87-6.04 (m, 1H), 7.14 (d, *J* = 8.25 Hz, 2H), 7.17 (d, *J* = 8.53 Hz, 2H), 7.21 (t, *J* = 7.70 Hz, 1H), 7.45 (t, *J* = 7.77 Hz, 2H), 7.67 (d, *J* = 8.39 Hz, 2H), 8.35 (br. s., 1H), 9.18 (br. s., 1H); **^{13}C NMR** (125 MHz, DMSO-*d*$_6$) (as a mixture of rotamers) δ 8.8, 8.9, 9.1, 11.5, 29.7, 29.9, 44.0, 47.0, 47.1, 54.0, 97.7, 115.5, 117.9, 119.4, 119.5, 124.4, 127.8, 127.9, 130.6, 130.6, 144.5, 153.9, 156.7, 157.8, 158.5, 158.8, 167.9. **HR-MS** (m/z): Calculated for $C_{30}H_{36}N_7O_2$ [M+H]$^+$: 526.2930; Found [M+H]$^+$: 526.2916

(R)-1-(3-(4-amino-3-(4-phenoxyphenyl)-1H-pyrazolo[3,4-d]pyrimidin-1-yl)piperidin-1-yl)-2-(piperidin-1-ylmethyl)prop-2-en-1-one (3c)

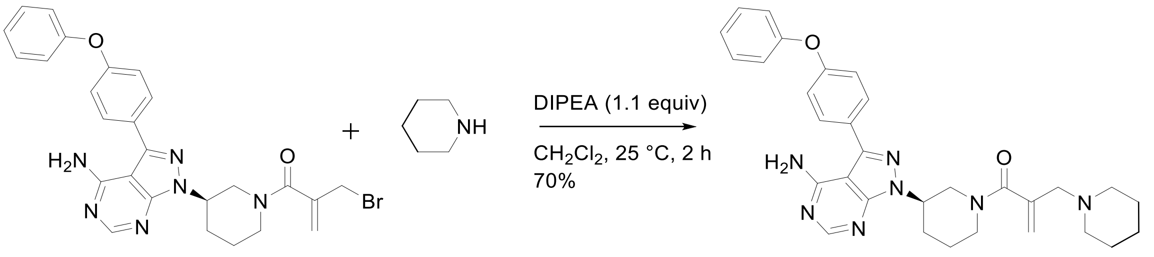

3n **3c**

To a stirred solution of bromo compound **3n** (26.5 mg, 0.05 mmol) in anhydrous DCM (1 mL), piperidine (5.43 μL, 0.055 mmol) and DIPEA (9.8 μL, 0.055 mmol) were added at 25 °C. The reaction mixture was stirred at room temperature for one hour. After completion of the reaction (as monitored by LC-MS), CH_2Cl_2 was evaporated under *vacuo*. The crude product was purified by preparative HPLC using water: ACN (0.1% TFA) solvent gradient to afford colorless solid in **3c** in 18.8 mg (yield = 70%).

^{1}H NMR (500 MHz, CD_3OD): δ1.50–1.55 (m, 1H), 1.77-1.85 (m, 4H), 1.97 (m, 2H), 2.09 (br. s., 1H), 2.27-2.31 (m, 1H), 2.41 (br. s., 1H), 2.88-2.92 (m, 2H), 3.23 (br. s., 0.4H), 3. 45 (br. s., 0.6H) 3.55-3.57 (br. s, 2H), 3.71 (br. s., 0.6H), 3.83 (br. s., 0.4H), 3.88-3.91 (m, 2H), 4.00 (br. s., 0.6H), 4.30 (br. s., 0.8H), 4.47 (br. s., 0.6H), 5.03 (br. s., 1H), 5.85 (s, 1H), 5.92 (br. s., 1H), 7.11 (d, J = 7.8 Hz, 2H), 7.19 (d, J = 8.7 Hz, 2H), 7.21-7.25 (t, J = 7.4 Hz, 1H), 7.42 (t, J = 8.0 Hz, 2H), 7.69 (d, J = 8.5 Hz, 2H), 8.41 (s, 1H); **^{13}C NMR** (125 MHz, CD_3OD) δ 22.8, 24.3, 25.5, 30.8, 34.2, 43.3, 47.0, 52.3, 54.2, 54.6, 60.7, 98.5, 120.2, 120.9, 125.5, 127.5, 128.9, 131.3, 131.5, 133.3, 148.3, 153.7, 155.3, 157.8, 160.8, 162.6, 162.9, 170.0. **HR-MS** (m/z): Calculated for $C_{31}H_{36}N_7O_2$ $[M+H]^+$: 538.2930; Found $[M+H]^+$: 538.2938

(R)-1-(3-(4-amino-3-(4-phenoxyphenyl)-1H-pyrazolo[3,4-d]pyrimidin-1-yl)piperidin-1-yl)-2-((methyl(phenyl)amino)methyl)prop-2-en-1-one (3d)

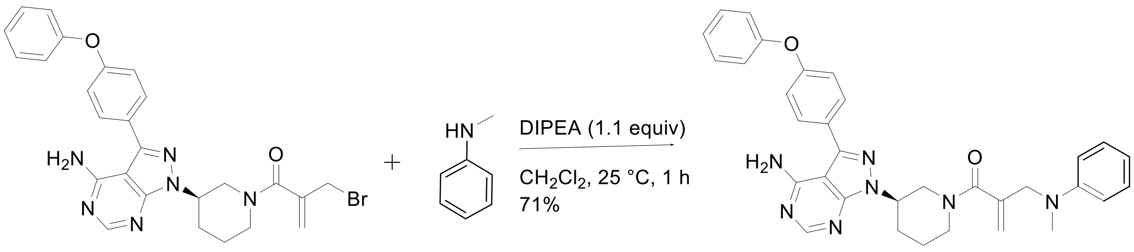

3n **3d**

To a stirred solution of bromo compound **3n** (26.5 mg, 0.05 mmol) in anhydrous DCM (1 mL) were added N-methyl aniline (5.95 µL, 0.055 mmol) and DIPEA (9.8 µL, 0.055 mmol) at 25 °C. The reaction mixture was stirred at room temperature for one hour. After completion of the reaction (as monitored by LC-MS), CH_2Cl_2 was evaporated under *vacuo*. The crude product was purified by HPLC using water: ACN (0.1% TFA) solvent gradient to afford colorless solid **3d** in19.8 mg (yield = 71%).

^{1}H NMR (500 MHz, CD_3OD) δ 1.49-1.62 (br. s., 1H), 1.98 (br. s., 1H), 2.19 (br. s., 1H), 2.24 (br. s., 1H), 3.02 (br. s., 3H), 3.66 (dd, J = 13.1, 9.4 Hz, 1H), 3.82 (br. s., 1H), 4.08-4.18 (m, 1H), 4.19-4.29 (m, 1H), 4.39 (br. s., 1H), 5.24-5.34 (m, 1H), 5.41 (br. s., 1H), 6.79-6.87 (m, 3H), 7.10 (m, 3H), 7.19-7.25 (m, 4H), 7.42 (t, J = 7.9 Hz, 2H), 7.69 (d, J = 8.3 Hz, 2H), 8.37-8.46 (m, 1H); **^{13}C NMR** (125 MHz, CD_3OD) δ 25.4, 30.8, 40.1, 46.9, 52.3, 54.4, 57.8, 98.5, 114.8, 120.4, 121.0, 124.1, 125.6, 127.4, 130.6, 131.6, 132.6, 141.7,

148.3, 148.6, 149.8, 153.5, 158.0, 161.0, 172.2. **HR-MS** (m/z): Calculated for $C_{33}H_{34}N_7O_2$ $[M+H]^+$: 560.2774; Found $[M+H]^+$: 560.2782.

(R)-1-(3-(4-amino-3-(4-phenoxyphenyl)-1H-pyrazolo[3,4-d]pyrimidin-1-yl)piperidin-1-yl)-2-((benzylamino)methyl)prop-2-en-1-one (3e):

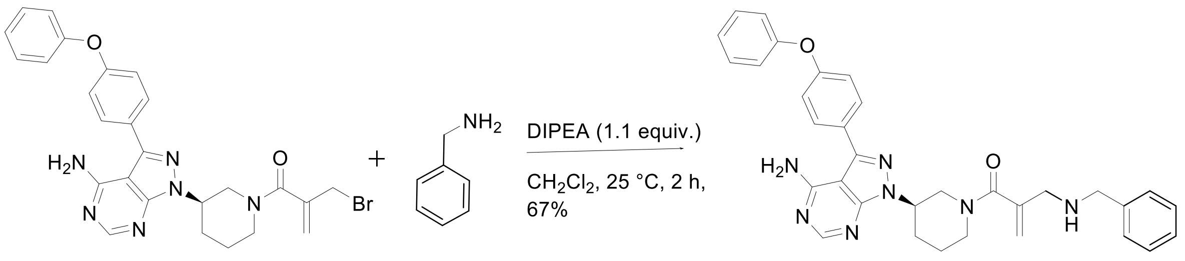

3n **3e**

To a stirred solution of bromo compound **3n** (26.5 mg, 0.05 mmol) in anhydrous DCM (1 mL) were added benzyl amine (6.0 µL, 0.055 mmol) and DIPEA (9.8 µL, 0.055 mmol) at 25 °C. The reaction mixture was stirred at room temperature for one hour. After completion of the reaction (as monitored by LC-MS), CH_2Cl_2 was evaporated under *vacuo*. The crude product was purified by preparative HPLC using water: ACN (0.1% TFA) solvent gradient to afford colorless solid **3e** in 18.7 mg (yield = 67%).

^{1}H NMR (500 MHz, CD_3OD): δ 1.82-1.93 (m, 1H), 2.16 (br. s., 1H), 2.36-40 (m, 1H), 2.50 (br. s., 1H), 3.24-3.32 (br. s., 0.4H), 3.50 - 3.58 (m, 0.6H), 3.80 (br.s., 1H), 3.96 (br. s, 2H), 4.12 (br. s., 0.6H), 4.30 (s., 2H), 4.41 (br. s., 0.8H), 4.57 (br. s., 0.6H), 5.11-5.44 (m, 1H), 5.87 (s, 1H), 5.96 (br. s., 1H), 7.19 (d, *J* = 7.8 Hz, 2H), 7.25 - 7.27 (m, 3H), 7.29-7.31(m, 1H), 7.51 (t, *J* = 8.4 Hz, 2H), 7.55-7.60 (m, 5H), 7.77-7.80 (d, *J* = 8.8 Hz, 2H) 8.35 (br. s., 1H); **^{13}C NMR** (125 MHz, CD_3OD) δ 25.6, 30.9, 43.3, 47.0, 50.8, 51.9, 54.2, 98.5, 120.2, 120.9, 125.5, 126.4, 127.6, 130.5, 131.0, 131.2, 131.3, 131.5, 132.4, 134.6, 148.2, 149.6, 153.8, 155.6, 157.8, 160.8, 170.2. **HR-MS** (m/z): Calculated for $C_{33}H_{34}N_7O_2$ $[M+H]^+$: 560.2774; Found $[M+H]^+$: 560.2786.

(R)-1-(2-(3-(4-amino-3-(4-phenoxyphenyl)-1H-pyrazolo[3,4-d]pyrimidin-1-yl)piperidine-1-carbonyl)allyl)-4-(dimethylamino)pyridin-1-ium (3f):

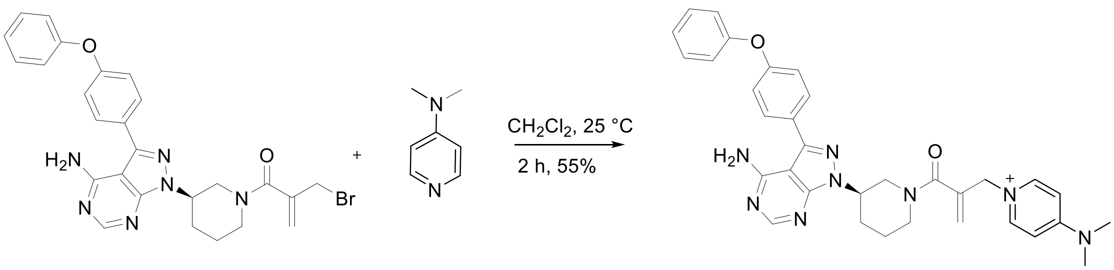

3n 3f

To a stirred solution of bromo compound **3n** (26.5 mg, 0.05 mmol) in anhydrous CH_2Cl_2 (1 mL) were added N,N-dimethylaminopyridine (6.7 mg, 0.055 mmol) at 25 °C. The reaction mixture stirred at room temperature for one hour. After completion of the reaction (as monitored by LC-MS), CH_2Cl_2 was evaporated under *vacuo*. The crude product was purified by preparative HPLC using water: ACN (0.1% TFA) solvent gradient to afford colorless solid **3f** in 15.8 mg (yield = 55%).

^{1}H NMR (500 MHz, CD_3OD): δ 1.61 (m, 1H), 2.03 (br. s., 1H), 2.22 (m, 1H), 2.34 (br. s., 1H), 3.05 (br. s., 0.4H) 3.26 (s, 6H), 3.40 (br. s., 0.6H), 3.69 (br. s., 1H), 3.93 (br. s., 0.5H), 4.34 (br. s., 1.5H), 4.98 (br. s., 2H), 5.61 (br. s., 2H), 7.01 (d, *J* = 7.6 Hz, 2H), 7.09 (m, *J* = 8.1 Hz, 2H), 7.15-7.19 (m, 3H), 7.41 (t, *J* = 7.8 Hz, 2H), 7.68 (m, *J*=8.5 Hz, 2H), 8.06-8.21 (m, 2H), 8.39 (s, 1H); **^{13}C NMR** (125 MHz, CD_3OD) δ 24.0, 29.3, 32.7, 39.0, 41.6, 45.4, 52.5, 59.1, 97.1, 107.6, 118.6, 119.2, 120.8, 123.9, 126.3, 129.7, 137.7, 141.9, 146.3, 149.1, 152.4, 156.4 156.7, 159.1, 167.8. **HR-MS** (m/z): for $C_{33}H_{35}N_8O_2$ $[M]^+$: 575.2883; Found $[M]^+$: 575.2908.

(R)-1-(3-(4-amino-3-(4-phenoxyphenyl)-1H-pyrazolo[3,4-d]pyrimidin-1-yl)piperidin-1-yl)-2-((triethyl-l4-azaneyl)methyl)prop-2-en-1-one (3g):

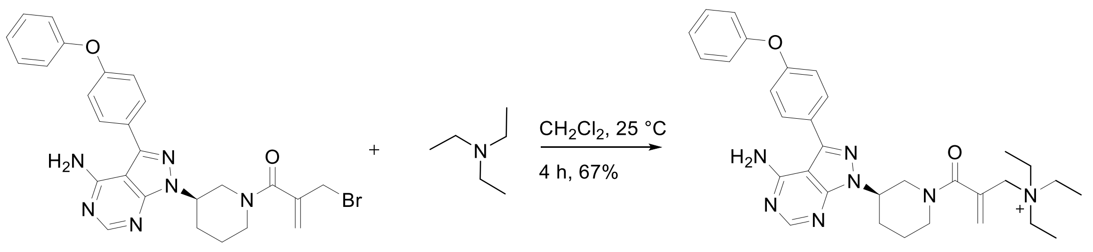

3n 3g

To a stirred solution of bromo compound **3n** (26.5 mg, 0.05 mmol) in anhydrous DCM (1 mL) triethyl amine (69.5 µL, 0.5 mmol) was added at 25 °C. The reaction mixture was

stirred at room temperature for 4 hours. After completion of the reaction (as monitored by LC-MS), CH_2Cl_2 was evaporated under *vacuo*. The crude product was purified by preparative HPLC using water: ACN (0.1% TFA) solvent gradient to afford colorless solid **3g** in 19.1 mg (yield = 67%).

^{1}H NMR (400 MHz, CD_3OD) δ 1.32 (t, J = 7.2 Hz, 9 H), 1.79 (br. s., 1H), 2.16 (br. s., 1H), 2.33 (br. s., 1H), 2.40 (br. s., 1H), 3.08 (br. s., 0.3H), 3.29 (br. s., 6H), 3.61 (br. s., 0.7H), 3.81 (br. s., 1H), 3.97 (br. s., 0.5H), 4.20 (br. s., 2H), 4.41 (m, 1.5H), 5.04 (br. s., 1H), 6.03 (s, 1H), 6.10 (br. s., 1H), 7.11 (d, J = 7.7 Hz, 2H), 7.17 (d, J = 8.6 Hz, 2H), 7.21 (m, 1H), 7.42 (m, 2H), 7.69 (d, J = 8.6 Hz, 2H), 8.44 (br. s., 1H): **^{13}C NMR** (101 MHz, CD_3OD) δ 6.4, 23.6, 29.1, 41.9, 45.5, 50.8, 53.0, 57.9, 97.0, 118.6, 119.3, 123.9, 125.8, 129.8, 131.5, 147.1, 152.0, 153.5, 156.3, 159.3, 160.3, 167.8. **HR-MS** (m/z): Calculated for $C_{32}H_{41}N_7O_2$ $[M]^+$: 554.3243; Found $[M]^+$: 554.3251.

(R)-2-(3-(4-amino-3-(4-phenoxyphenyl)-1H-pyrazolo[3,4-d]pyrimidin-1-yl)piperidine-1-carbonyl)allyl acetate (3h)

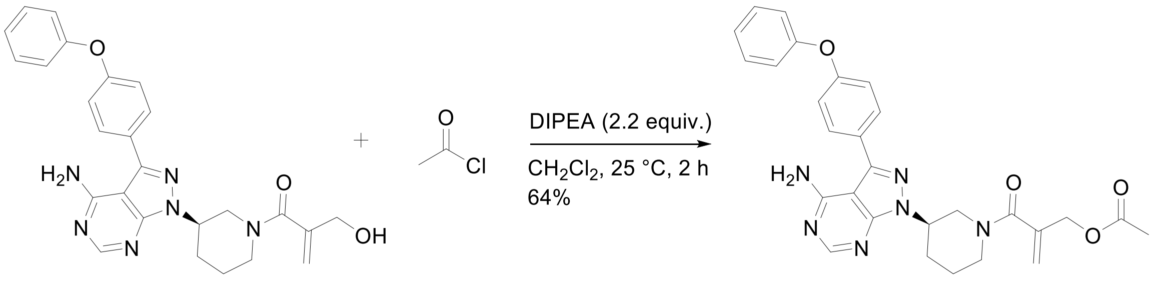

3m 3h

To a stirred solution of alcohol **3m** (22 mg, 0.05 mmol) in anhydrous DCM (0.5 mL) were added acetyl chloride (4.25 μL, 0.06 mmol) and DIPEA (10.6 uL, 0.06 mmol) at 25 °C. The reaction mixture was stirred at room temperature for 1 h. After completion of the reaction (as monitored by LC-MS), CH_2Cl_2 was evaporated under *vacuo*. The crude product was purified by Preparative HPLC using water:ACN (0.1% TFA) solvent gradient to afford colorless solid **3h** in 16.3 mg (yield = 64%).

^{1}H NMR (400 MHz, CD_3OD): δ 1.85 (d, J = 10.3 Hz, 1H), 2.17 (br. s., 4H), 2.27-2.42 (m, 1H), 2.50 (d, J = 9.2 Hz, 1H), 3.19 (br. s., 0.4H), 3.58 (br. s., 0.7H), 3.84 (br. s., 1H), 4.08 (br. s., 0.6H), 4.40 (br. s., 0.4H), 4.49 (br. s., 1H), 4.84 (br. s., 2H), 5.08 (br. s., 1H), 5.48 (br. s., 1H), 5.59 (br. s., 1H), 5.65 (br. s., 1H), 7.15-7.34 (m, 5H), 7.44-7.59 (m, 2H), 7.79 (d, J = 8.6 Hz, 2H), 8.50 (s, 1H); **^{13}C NMR** (100 MHz, CD_3OD) δ 20.8, 24.0, 30.8, 43.0, 46.8, 54.2, 65.9, 98.5, 119.1, 120.2, 120.9, 125.4, 127.4, 131.2, 131.5, 140.9, 148.4, 155.8, 157.9, 159.7, 160.8, 170.9, 172.4. **HR-MS** (m/z): Calculated for $C_{28}H_{29}N_6O_4$ $[M+H]^+$: 513.2250; Found $[M+H]^+$: 513.2252.

(R)-2-(3-(4-amino-3-(4-phenoxyphenyl)-1H-pyrazolo[3,4-d]pyrimidin-1-yl)piperidine-1-carbonyl)allyl acetate (3i)

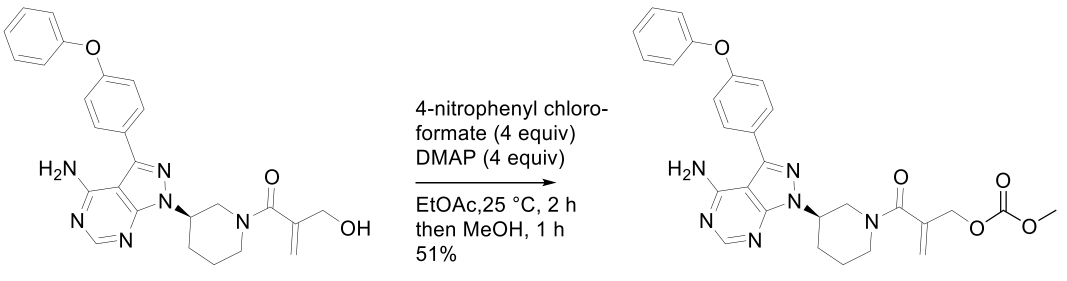

To a stirred solution of alcohol **3m** (23.5 mg, 0.05 mmol) in ethyl acetate (0.5 mL) were added 4-nitrophenyl chloroformate (40.8 mg, 0.2 mmol) and 4-Dimethylaminopyridine (24.4 mg, 0.2 mmol) at 0 °C. The reaction mixture was stirred at room temperature for 2 h. After completion of the reaction (as monitored by LC-MS, the reaction mixture is quenched with 0.1 N HCl (2 mL) and the aqueous layer was extracted with EtOAc (3 × 3 mL). The organic layer was evaporated under *vacuo* and dissolved in MeOH (0.5 mL). The reaction is further stirred for 1 hour at room temperature. After completion of the reaction, methanol was concentrated and the crude product was purified by HPLC using water:ACN (0.1% TFA) solvent gradient to afford colorless solid **3i** in 14.2 mg (yield = 51%).

^{1}H NMR (400 MHz, CD_3OD): δ 1.80-1.91 (m, 1H), 2.15-2.23 (m, 1H), 2.33-2.40 (m, 1H), 2.50 (br. s., 1H), 3.56 (br. s., 1H), 3.87 (br. s., 3H), 3.99-4.17 (m, 1H), 4.46-4.60 (m, 1H), 4.87 (d, J = 12.1 Hz, 2H), 5.04-5.15 (m, 1H), 5.51 (br. s., 1H), 5.68 (br. s., 1H), 7.12-7.37 (m, 5H), 7.48-7.56 (m, 2H), 7.79 (d, J = 8.6 Hz, 2H), 8.50 (s, 1H); **^{13}C NMR** (100 MHz, CD_3OD) δ 28.9, 30.8, 43.0 46.8, 54.2, 55.7, 69.2, 98.4 119.6, 120.2, 120.8, 125.4, 127.5,

131.3, 131.5, 133.2, 140.5, 148.4, 149.0, 157.0, 157.9, 160.8, 170.7. **HR-MS** (m/z): Calculated for $C_{28}H_{29}N_6O_5$ $[M+H]^+$: 529.2199; Found $[M+H]^+$: 529.2212.

(R)-1-(3-(4-amino-3-(4-phenoxyphenyl)-1H-pyrazolo[3,4-d]pyrimidin-1-yl)piperidin-1-yl)-2-(phenoxymethyl)prop-2-en-1-one (3j)

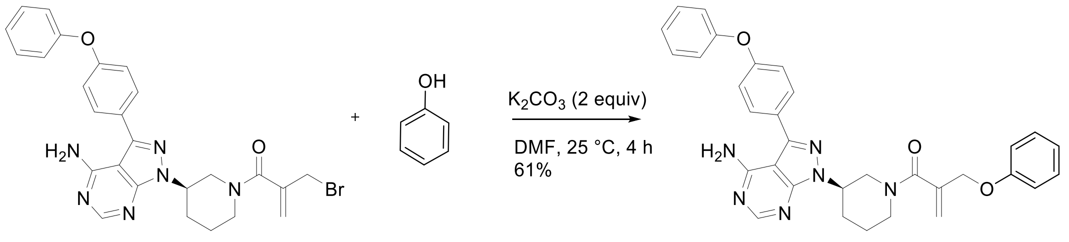

To a stirred solution of bromo compound **3n** (26.5 mg, 0.05 mmol) in dry DMF (1 mL), phenol (4.7 µL, 0.055 mmol) and K_2CO_3 (15.2 mg, 0.11 mmol), were added at 25 °C under N_2 atmosphere. The reaction mixture was stirred at room temperature for 4 h. After completion (as monitored by LC-MS), the reaction mixture was quenched with H_2O (2 mL). The aqueous layer was extracted with EtOAc (3 × 3 mL). The organic layer was washed with brine and evaporated under *vacuo*. The crude product was purified by preparative HPLC using water:ACN (0.1% TFA) solvent gradient to afford colorless solid **3j** in 16.6 mg (yield = 61%).

^{1}H NMR (400 MHz, CD_3OD) δ 1.84 (br. s., 1H), 2.33 - 2.41 (m, 1H), 2.46 (d, *J*=11.4 Hz, 1H), 3.00 (br. s., 1H), 3.25 - 3.48 (m, 1H), 3.68 (br. s., 1H), 3.86 (br. s., 1H), 4.66 - 4.84 (m, 2H), 4.88 (br. s., 1H), 4.99 - 5.11 (m, 1H), 5.48 (br. s., 1H), 5.72 (br. s., 1H), 6.47 (br. s., 1H), 6.96 - 7.12 (m, 2H), 7.21 (m, *J*=7.7 Hz, 2H), 7.27 (d, *J*=8.1 Hz, 1H), 7.32 - 7.43 (m, 3H), 7.46 - 7.59 (m, 2H), 7.67 (m, *J*=8.4 Hz, 2H), 8.40 (br. s., 1H), 10.56 (br. s., 1H); **^{13}C NMR** (100 MHz, CD_3OD): δ 24.6, 30.2, 45.6, 47.3, 53.3, 68.8, 96.9, 114.6, 118.1, 119.2, 120.0, 121.4, 124.7, 129.5, 129.7, 130.2, 139.7, 145.0, 147.2, 151.4, 153.0, 155.5, 158.1, 160.0, 169.7. **HR-MS** (m/z): Calculated for $C_{32}H_{31}N_6O_3$ $[M+H]^+$: 547.2458; Found $[M+H]^+$: 547.2452.

(R)-7-((2-(3-(4-amino-3-(4-phenoxyphenyl)-1H-pyrazolo[3,4-d]pyrimidin-1-yl)piperidine-1-carbonyl)allyl)oxy)-2H-chromen-2-one (3k):

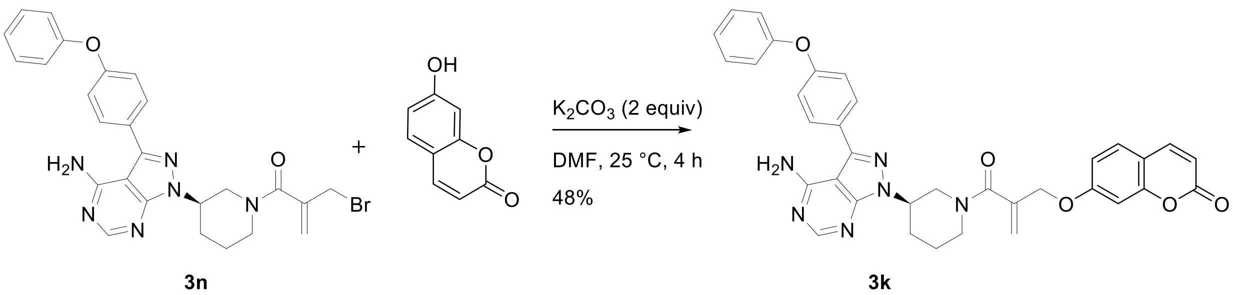

To a stirred solution of bromo compound **3n** (26.5 mg, 0.05 mmol) in dry DMF (1 mL), 7-hydroxy coumarin (8.9 mg, 0.055 mmol) and K_2CO_3 (15.2 mg, 0.11 mmol) were added at 25 °C under an N_2 atmosphere. The reaction mixture was stirred at room temperature for 4 h. After completion (as monitored by LC-MS), the reaction mixture was quenched with H_2O (2 mL). The aqueous layer was extracted with EtOAc (3 × 3 mL). The organic layer was washed with brine and evaporated under *vacuo*. The crude product was purified by preparative HPLC using water:ACN (0.1% TFA) solvent gradient to afford colorless solid **3k** in 14.8 mg (yield = 48%).

^{1}H NMR (500 MHz, CD_3OD) δ 1.73 (m, 1H), 2.13 (br. s., 1H), 2.30 (br. s., 1H), 2.38 (br. s., 1H),3.15 (br. s., 0.5H) 3.60 (br. s., 0.5H), 3.88 (dd, *J* = 12.8, 9.2 Hz, 1H), 3.93-3.97 (m, 0.4H), 4.34-4.37 (m, 1H), 4.40-4.42 (m, 1H), 4.74-4.81 (m, 1H), 4.88-5.02 (s, 2H, merged with solvent peak), 5.49 (s, 1H), 5.71 (br. s., 1H), 6.24-6.28 (m, 1H), 7.02 (br. s., 2H), 7.11 (br. s., 3H), 7.15-7.23 (m, 2H), 7.42 (t, *J* = 7.6 Hz, 2H), 7.54 (br. s., 1H), 7.68 (d, *J* = 8.7 Hz, 2H), 7.85-7.90 (m, 1H), 8.43 (s, 1H); **^{13}C NMR** (125 MHz, CD_3OD) δ 23.1, 29.0, 45.2, 50.6, 54.0, 69.0, 96.8, 101.4, 112.4, 112.7, 113.1, 118.7, 119.3, 125.7, 129.2, 129.7, 129.9, 139.3, 144.2, 146.5, 147.0, 151.8, 152.9, 155.5, 156.3, 159.2, 161.5, 169.5. **HR-MS** (m/z): Calculated for $C_{35}H_{31}N_6O_5$ $[M+H]^+$: 615.2356; Found $[M+H]^+$: 615.2418

methyl (E)-3-(5-(((1r,3r,5R,7S)-adamantan-2-ylidene)(methoxy)methyl)-2-((2-((R)-3-(4-amino-3-(4-phenoxyphenyl)-1H-pyrazolo[3,4-d]pyrimidin-1-yl)piperidine-1-carbonyl)allyl)oxy)-3-chlorophenyl)acrylate (3o):

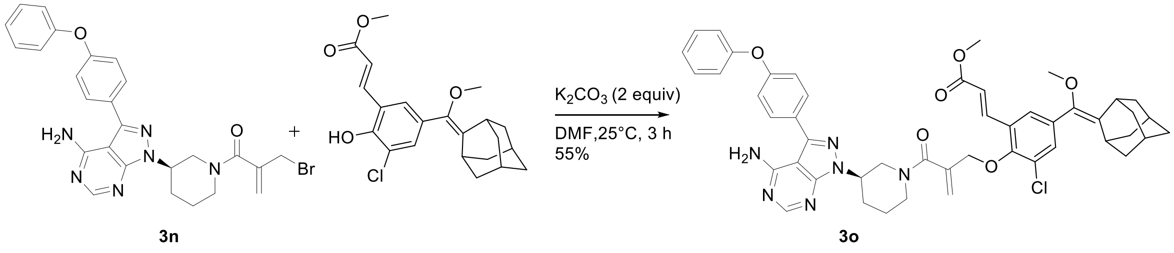

To a stirred solution of phenol (4.83 μL, 0.055 mmol) in dry DMF (1 mL), K_2CO_3 (15.2 mg, 0.11 mmol) and bromo compound **3n** (26.5 mg, 0.05 mmol) were added at 25 °C under N_2 atmosphere. The reaction mixture was stirred at room temperature for 4 h. After completion (as monitored by LC-MS), the reaction mixture was quenched with H_2O (2 mL). The aqueous layer was extracted with EtOAc (3 × 3 mL). The organic layer was washed with brine and evaporated under *vacuo*. The crude product was purified by preparative HPLC using water:ACN (0.1% TFA) solvent gradient to afford colorless solid **3o** in 23.1 mg (yield = 55%).

^{1}H NMR (400 MHz, CD_3OD) δ 1.83 (br. s., 2H), 1.86 - 2.00 (m, 7H), 2.04 (br. s., 4H), 2.15 (br. s., 2H), 2.35 (br. s., 1H), 2.48 (q, *J* = 9.7 Hz, 1H), 3.34 (br. s., 3H), 3.77-3.85 (br. s., 3H), 3.97-4.19 (br. s., 2H), 4.41-4.61 (m, 2H), 4.77 (br. s., 1H), 4.86 (br. s., 1H), 5.10 - 5.21 (m, 1H), 5.57 (br. s., 1H), 5.79-5.87 (br. s., 1H), 6.58 (d, *J* = 16.1 Hz, 1H), 7.11-7.34 (m, 6H), 7.48-7.57 (m, 2H), 7.72 (br. s., 3H), 7.82 (d, *J* = 16.3 Hz, 1H), 7.99 (d, *J* = 15.2 Hz, 1H), 8.21 (br. s., 1H); **^{13}C NMR** (125 MHz, CD_3OD) δ 29.8, 29.9, 31.3, 34.6, 38.2, 39.7, 39.8, 40.2, 40.3, 43.1, 46.8, 52.4, 54.3, 55.2, 57.7, 75.6, 75.8, 98.2, 120.1, 120.9, 125.4, 126.9, 129.6, 130.7, 131.3, 131.4, 139.6, 141.1, 141.4, 148.4, 153.4, 154.7, 155.0, 157.8, 160.7, 168.6, 170.9. **HR-MS** (m/z): Calculated for $C_{48}H_{50}ClN_6O_6$ $[M+H]^+$: 841.3480; Found $[M+H]^+$: 841.3475.

Methyl (E)-3-(2-((2-((R)-3-(4-amino-3-(4-phenoxyphenyl)-1H-pyrazolo[3,4-d]pyrimidin-1-yl)piperidine-1-carbonyl)allyl)oxy)-3-chloro-5-((1r,3r,5R,7R)-4'-methoxyspiro[adamantane-2,3'-[1,2]dioxetan]-4'-yl)phenyl)acrylate (3l):

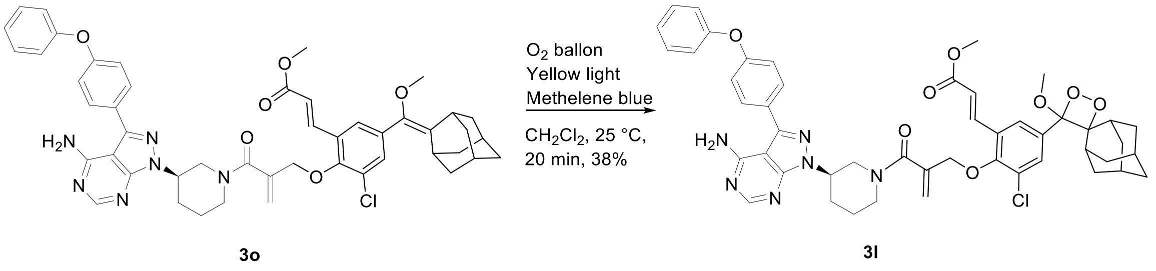

To a stirred solution of enol ether **3o** (8.4 mg, 0.01 mmol) in dry DCM (1 mL), methylene blue was added at 25 °C and in the presence of yellow light. The reaction mixture was bubbled with oxygen and stirred for 20 min. After completion (as monitored by LC-MS), the DCM was evaporated under *vacuo*. The crude product was purified by preparative HPLC using water:ACN (0.1% TFA) solvent gradient to afford colorless solid **3l** in 3.31 mg (yield = 38%).

^{1}H NMR (500 MHz, CD_3OD) δ 1.55-1.74 (m, 6H), 1.75-1.87 (m, 5H), 1.96 (s, 2H), 2.05 (br. s., 1H), 2.13-2.30 (m, 2H), 2.38 (br. s., 2H), 3.20 (s, 3H), 3.67 (br. s., 3H), 3.79 (br. s., 1H), 3.92 (br. s., 1H), 4.27-4.50 (m, 2H), 4.65 (br. s., 1H), 5.06 (br. s., 2H), 5.50 (br. s., 1H), 5.72 (br. s., 1H), 5.79 (br. s., 1H), 6.49-6.63 (m, 1H), 7.13 (br. s., 5H), 7.18-7.25 (m, 1H), 7.44 (t, J = 7.2 Hz, 2H), 7.64 (t, J = 8.5 Hz, 2H), 7.80 (br. s., 1H), 7.93 (d, J = 8.3 Hz, 1H): **^{13}C NMR** (125 MHz, CD_3OD) δ 27.6, 28.0, 28.9, 33.0, 33.4, 34.6, 35.1, 37.9, 38.7, 49.9, 50.3, 52.7, 52.7, 53.5, 75.8, 97.5, 113.1, 119.1, 119.6, 120.3, 121.0, 125.6, 127.4, 127.6, 131.4, 131.5, 131.6, 134.9, 137.0, 138.8, 141.4, 150.0, 158.0, 160.8, 162.4, 168.5, 171.3. **HR-MS** (m/z): Calculated for $C_{48}H_{50}ClN_6O_8$ $[M+H]^+$: 873.3379; Found $[M+H]^+$: 873.3362.

N-(4-((3-chloro-4-fluorophenyl)amino)-7-methoxyquinazolin-6-yl)-2-(((2-oxo-2H-chromen-7-yl)oxy)methyl)acrylamide (4b):

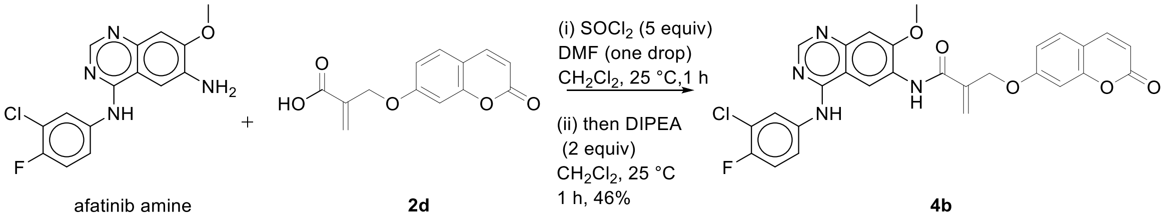

To a stirred solution of carboxylic acid **2d** (12.3 mg, 0.05 mmol) in CH_2Cl_2 (1 mL) were added $SOCl_2$ (18.1 µL, 0.25 mmol) and DMF (3.9 µL, 0.05 mmol) and the reaction mixture

was stirred at 25 °C for 4 h. After completion (as monitored by LC-MS), the reaction mixture was evaporated under *vacuo*. The crude acid chloride was dissolved in CH_2Cl_2 and slowly to the solution of afatinib amine (0.05 mmol, 15.9 mg) and DIPEA (17.8 μL, 0.1 mmol) was treated with purified by preparative HPLC using water:ACN (0.1% TFA) solvent gradient to afford colorless solid **4b** in 12.5 mg (yield = 46%).

^{1}H NMR (500 MHz, DMSO-d_6) δ 3.97 (br. s., 3H), 5.03 (br. s., 2H), 6.03 (br. s., 1H), 6.27-6.39 (m, 2H), 7.06 (d, *J* = 6.5 Hz, 1H), 7.12 (br. s., 1H), 7.32 (br. s., 1H), 7.37-7.46 (m, 1H), 7.69 (d, *J* = 8.3 Hz, 1H), 7.80 (br. s., 1H), 8.02 (d, *J* = 9.1 Hz, 1H), 8.56 (br. s., 1H), 8.82 (br. s., 1H), 9.59 (br. s., 1H), 9.85 (br. s., 1H); **^{13}C NMR** (125 MHz, DMSO-d_6) δ 56.4, 67.6, 101.7, 106.9, 108.8, 112.8, 112.8, 112.8, 113.0, 116.4, 116.5, 117.1, 122.4, 123.5, 124.9, 126.8, 129.6, 136.8, 138.6, 144.3, 149.5, 154.1, 155.3, 155.9, 156.8, 160.2, 160.9, 164.4. **HR-MS** (m/z): Calculated for $C_{28}H_{21}ClFN_4O_5$ $[M+H]^+$: 547.1185; Found $[M+H]^+$: 547.1184.

6-fluoro-7-(2-fluoro-6-hydroxyphenyl)-1-(2-isopropyl-4-methylpyridin-3-yl)-4-((S)-2-methyl-4-(2-(((2-oxo-2H-chromen-7-yl)oxy)methyl)acryloyl)piperazin-1-yl)pyrido[2,3-d]pyrimidin-2(1H)-one (5a):

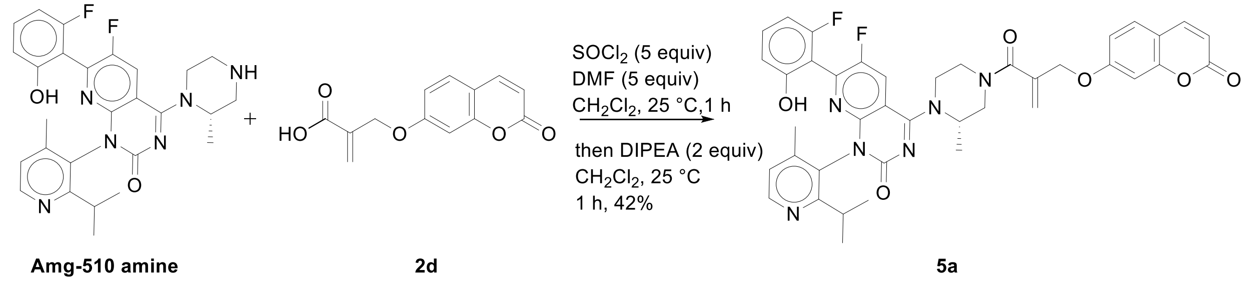

To a stirred solution of carboxylic acid **2d** (1.23 mg, 0.005 mmol) in CH_2Cl_2 (200 μL) were added $SOCl_2$ (1.81 μL, 0.025 mmol) and DMF (1.9 μL, 0.025 mmol) and the reaction mixture was stirred at 25 °C for 4 h. After completion (as monitored by LC-MS), the reaction mixture was evaporated under *vacuo*. The crude acid chloride was dissolved in CH_2Cl_2 and slowly added to the solution of Amg-510 amine (0.005 mmol, 2.53 mg) and DIPEA (1.78 μL, 0.01 mmol) and stirred at 25 °C under N_2 atmosphere. After completion (as monitored by LC-MS), the reaction mixture was evaporated under *vacuo* and purified

by preparative HPLC using water:ACN (0.1% TFA) solvent gradient to afford colorless solid **5a** in 1.7 mg (yield = 42%).

^{1}H NMR (500 MHz, CD_3OD) δ 1.15 (t, *J* = 7.1 Hz, 4H), 1.24-1.35 (m, 6H), 1.37-1.44 (m, 4H), 2.22 (d, *J* = 7.7 Hz, 3H), 3.02-3.11 (m, 3H), 4.47 (d, *J* = 19.8 Hz, 1H), 4.53 (br. s., 1H), 4.96 (br. s., 2H), 5.52-5.59 (m, 1H), 5.81 (s, 1H), 6.29 (d, *J* = 9.5 Hz, 1H), 6.63 (t, *J* = 8.9 Hz, 1H), 6.68 (s, 1H), 7.04 (s, 1H), 7.18 (d, *J* = 8.5 Hz, 1H), 7.26 (d, *J* = 7.3 Hz, 2H), 7.41-7.48 (m, 1H), 7.59 (d, *J* = 8.5 Hz, 1H), 7.64-7.73 (m, 2H), 7.91 (d, *J* = 9.5 Hz, 1H), 8.40 (d, *J* = 8.9 Hz, 1H), 8.54-8.60 (m, 1H). **HR-MS** (m/z): Calculated for $C_{40}H_{37}F_2N_6O_6$ $[M+H]^+$: 735.2743; Found $[M+H]^+$: 735.2747.

References:

(1) Zhang, J.; Loh, T.-P. Ruthenium- and Rhodium-Catalyzed Cross-Coupling Reaction of Acrylamides with Alkenes: Efficient Access to (Z,E)-Dienamides. *Chem. Commun.* **2012**, *48* (91), 11232–11234.

(2) Macpherson, L. J.; Dubin, A. E.; Evans, M. J.; Marr, F.; Schultz, P. G.; Cravatt, B. F.; Patapoutian, A. Noxious Compounds Activate TRPA1 Ion Channels through Covalent Modification of Cysteines. *Nature* **2007**, *445* (7127), 541–545.

(3) Vajjha, S.; Bommuluri, V.; Mohan P, K. V. K.; Rumalla, C. S.; Doddipalla, R.; Kaliyaperumal, M.; Korupolu, R. B. Degradation Studies of Ibrutinib under Stress Conditions: Characterisation and Structural Elucidation of Novel Degradants. *J. Pharm. Biomed. Anal.* **2019**, *172*, 9–17.

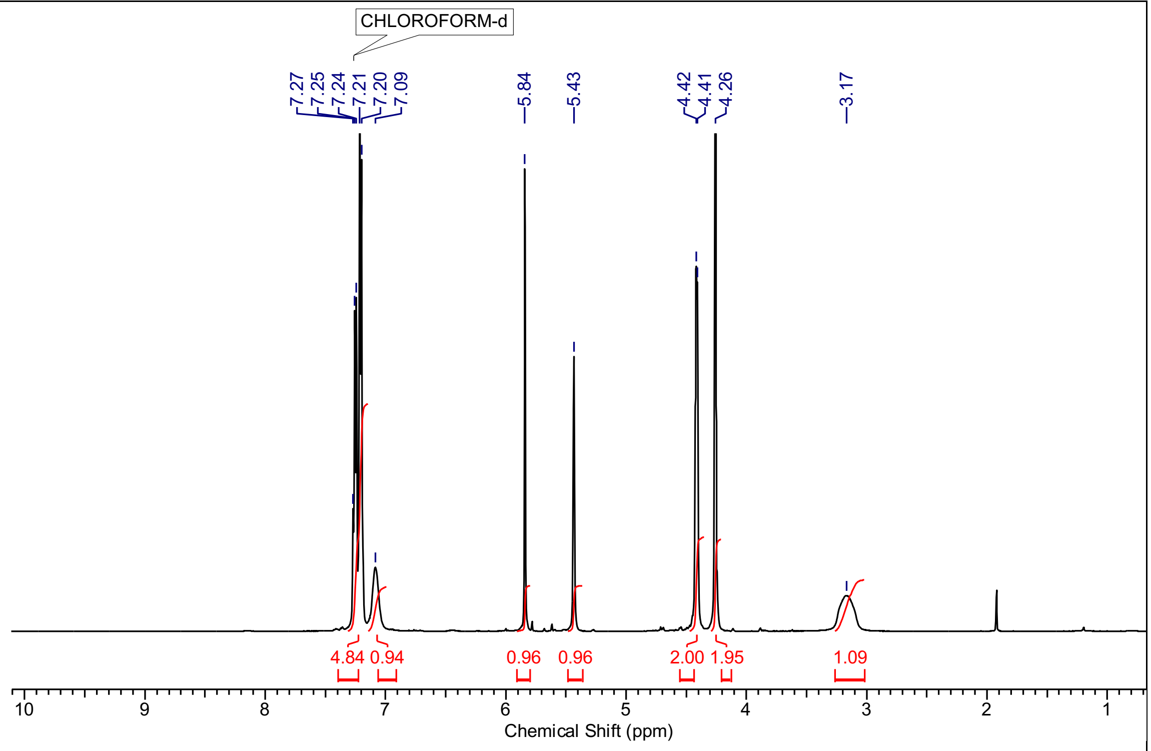

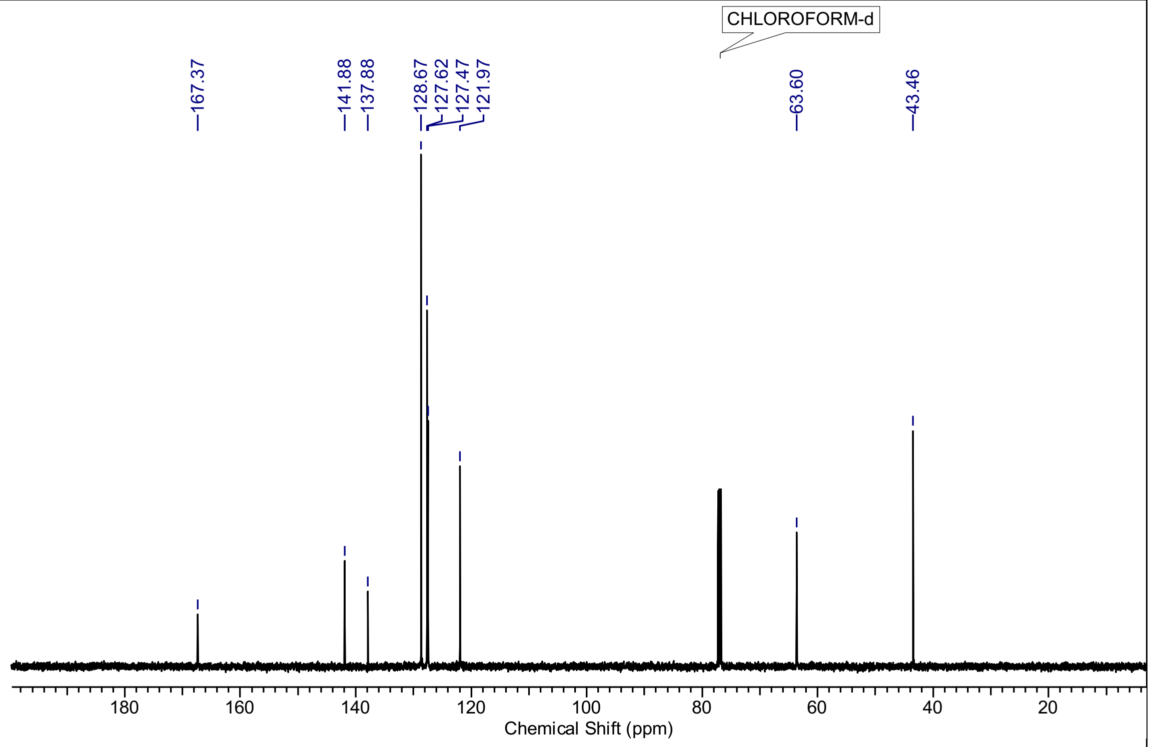

1H NMR and ^{13}C NMR spectra of **1n** (BnOH)

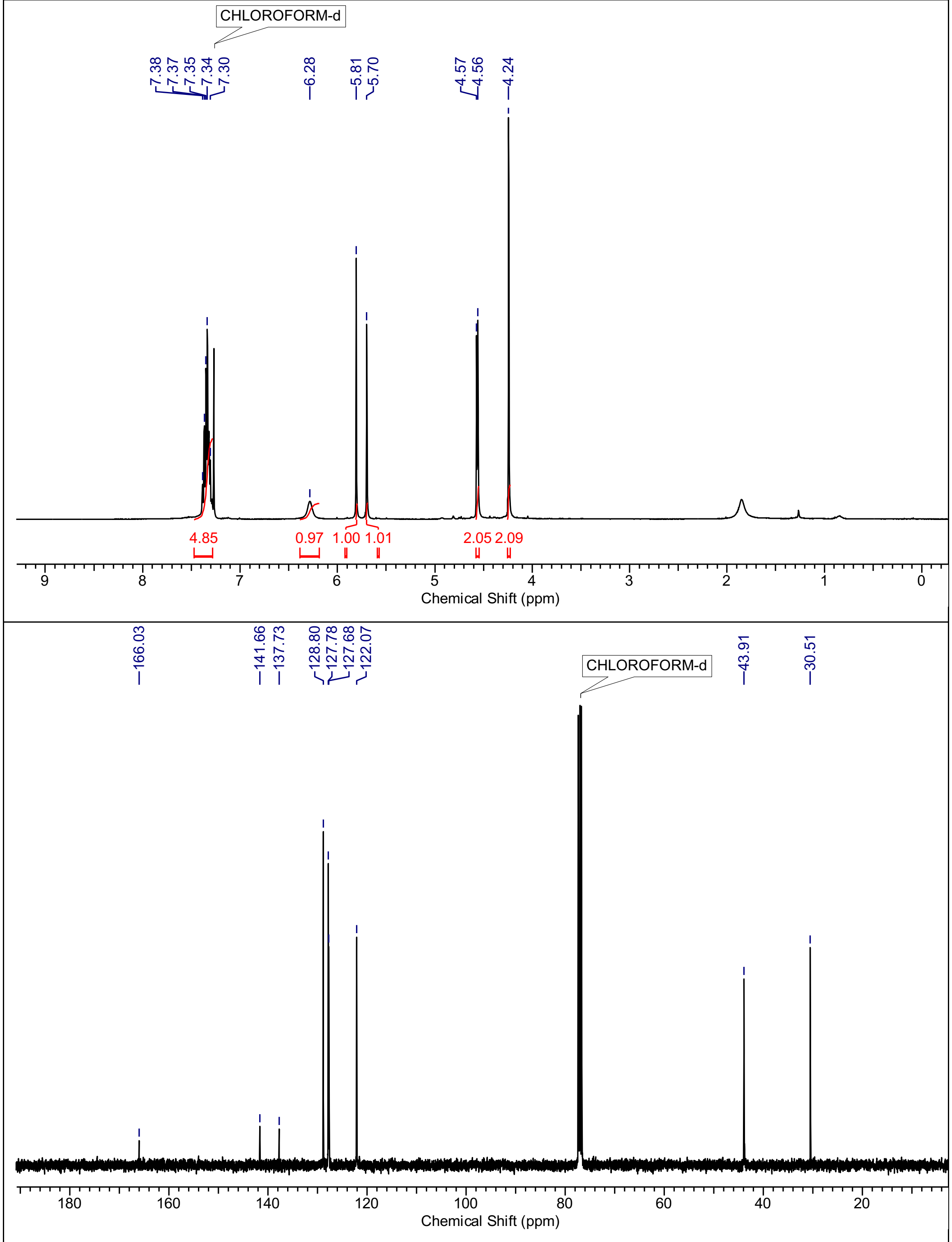

1H NMR and ^{13}C NMR spectra of **1o** (BnBr)

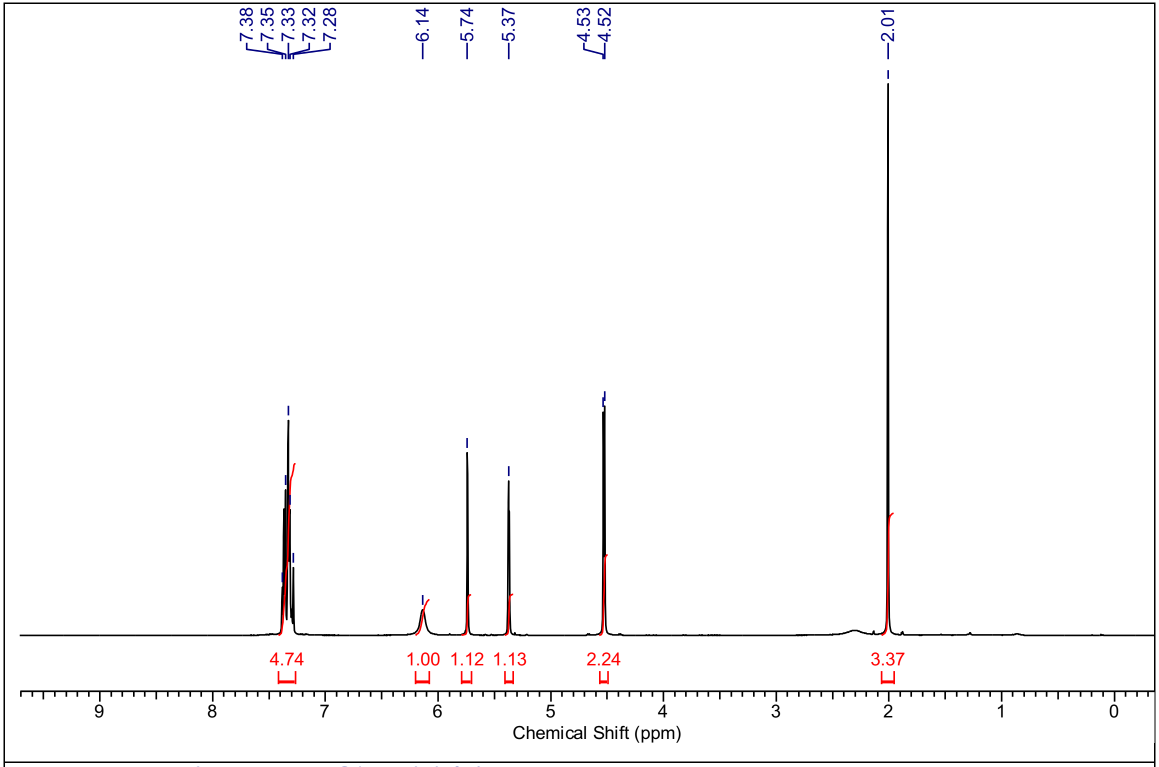

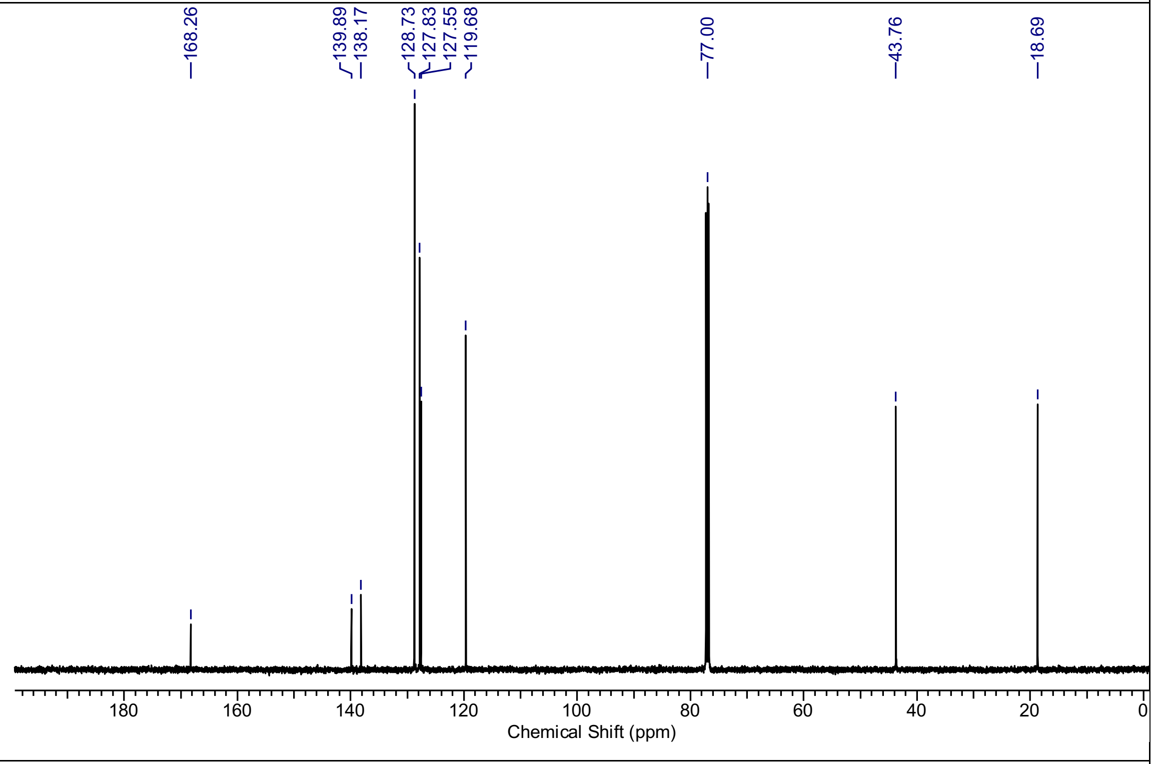

1H NMR and ^{13}C NMR spectra of ram-980 (**1a**)

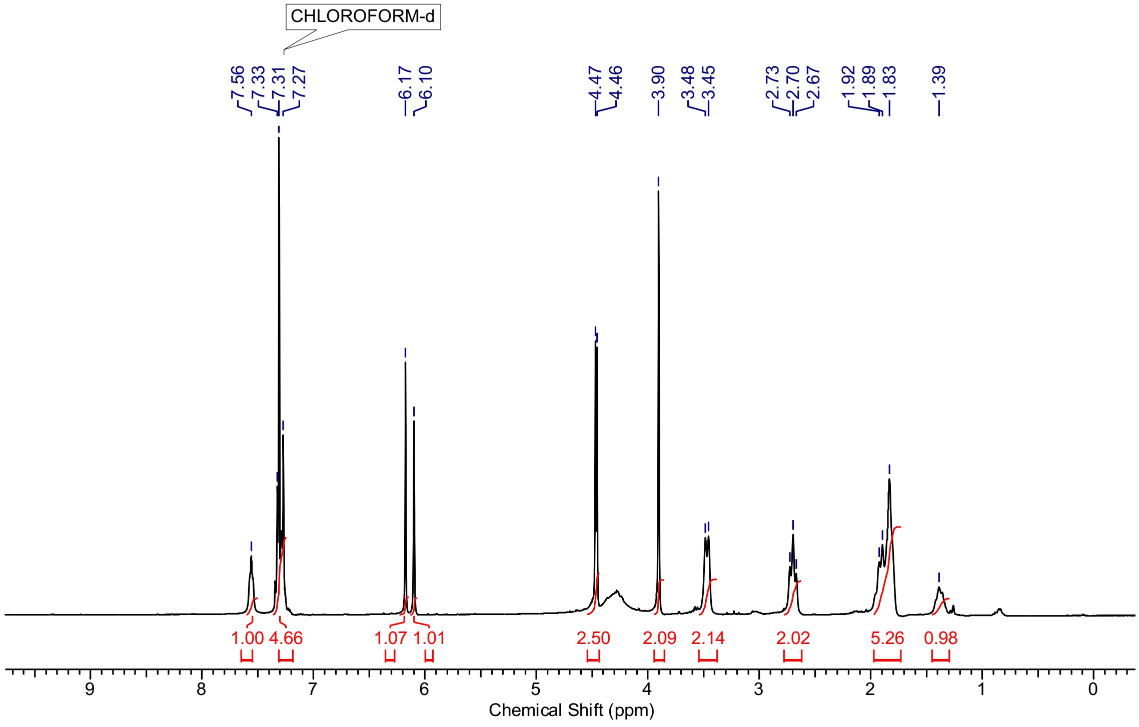

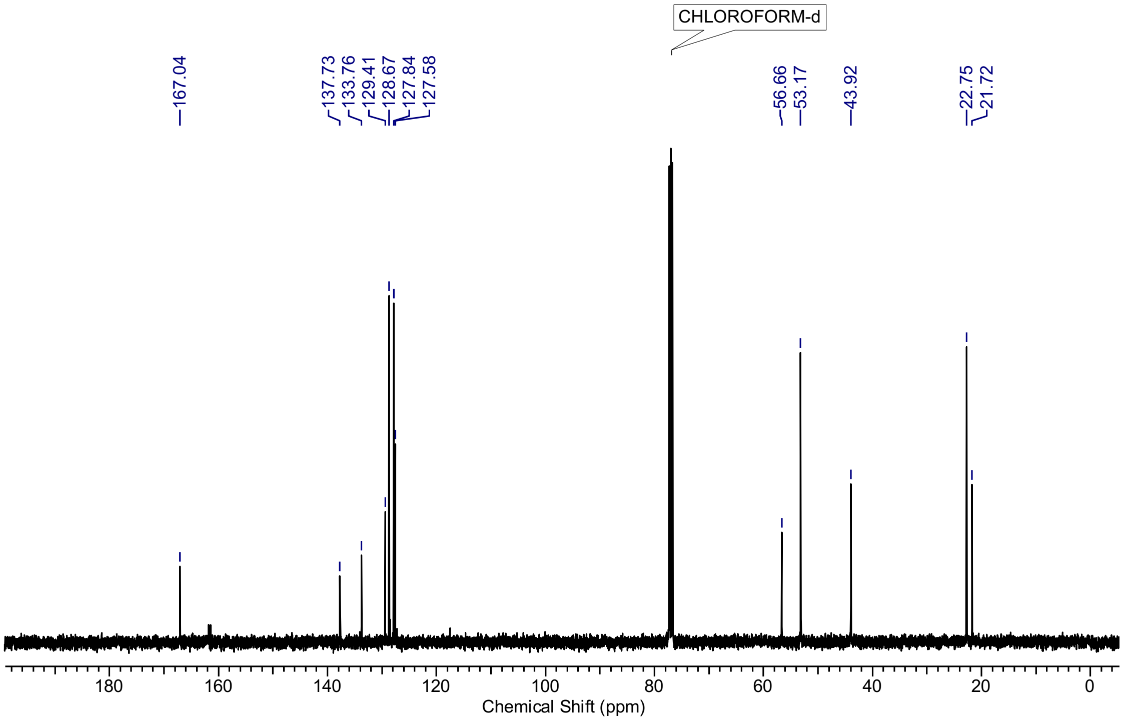

1H NMR and ^{13}C NMR spectra of **1b** **(ram-777)**

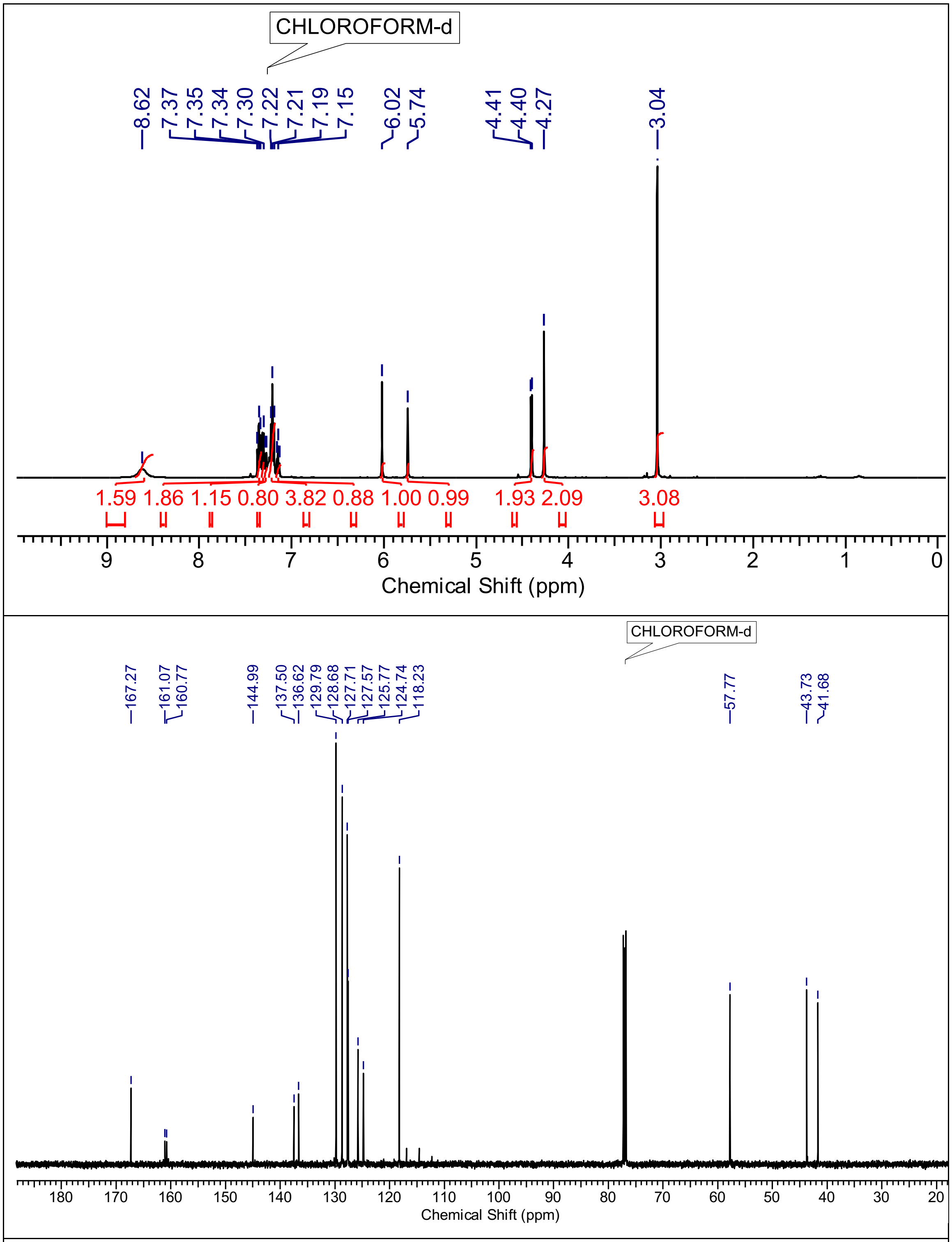

1H NMR and ^{13}C NMR spectra of **(ram-726) 1c**

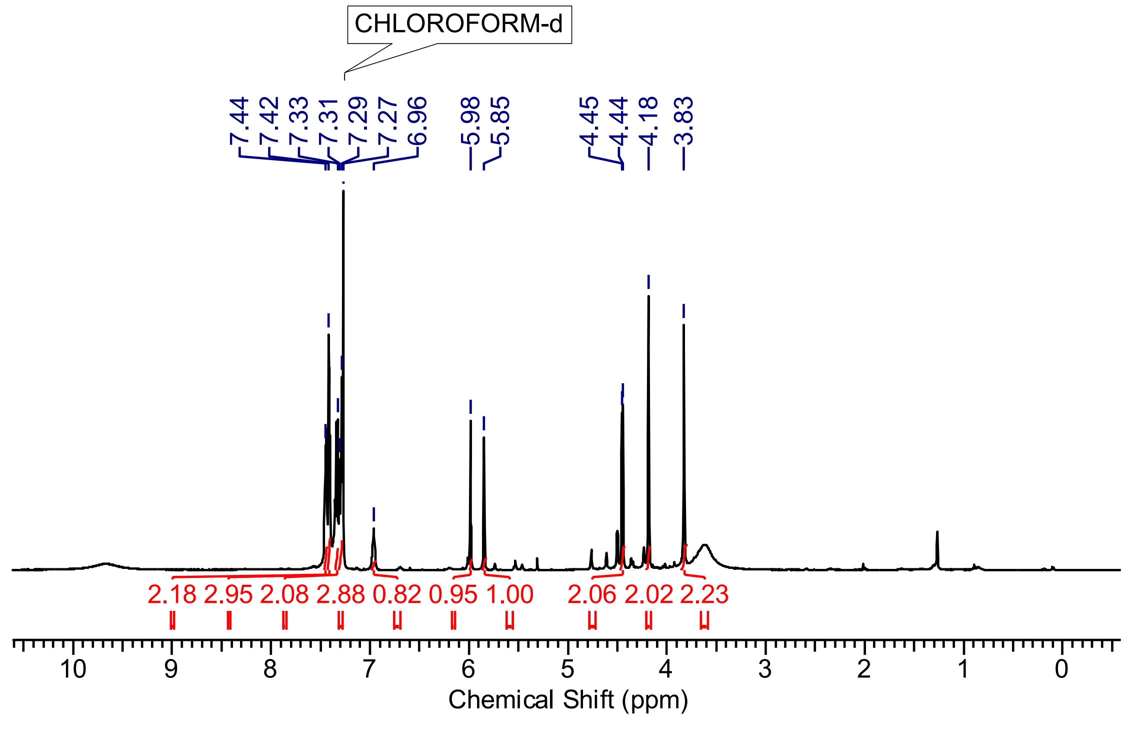

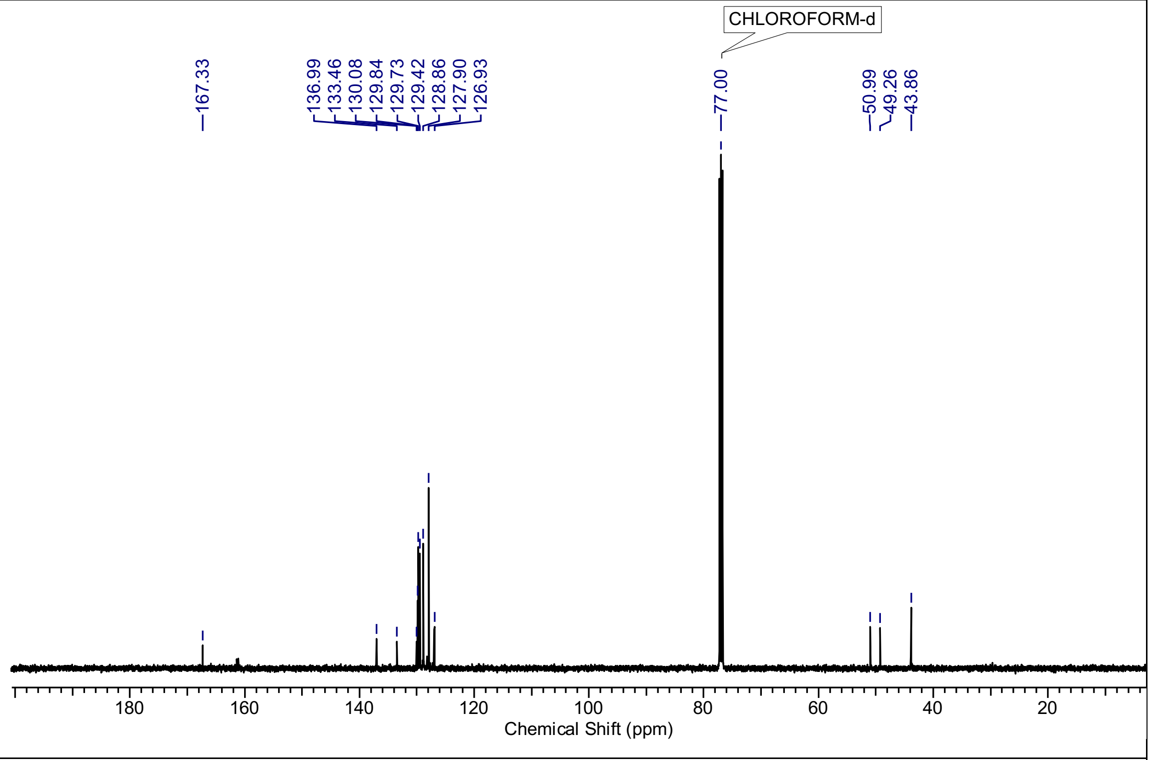

1H NMR and ^{13}C NMR spectra of **(ram-981) 1d**

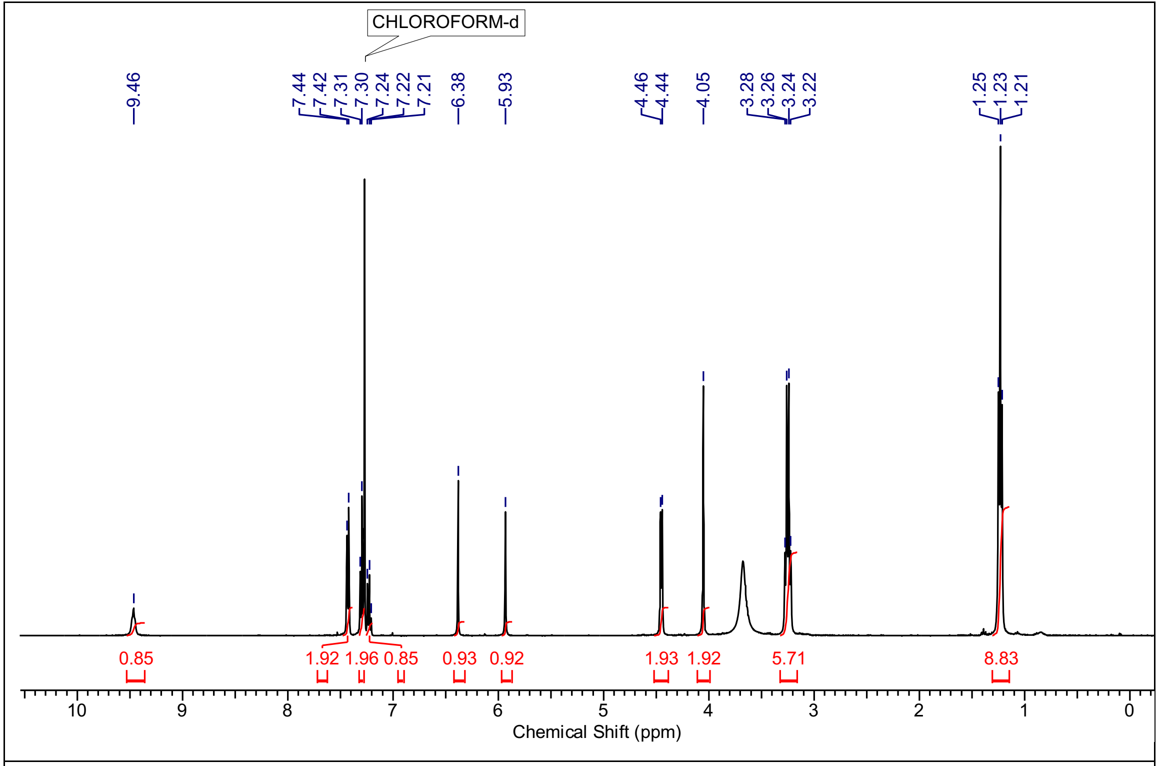

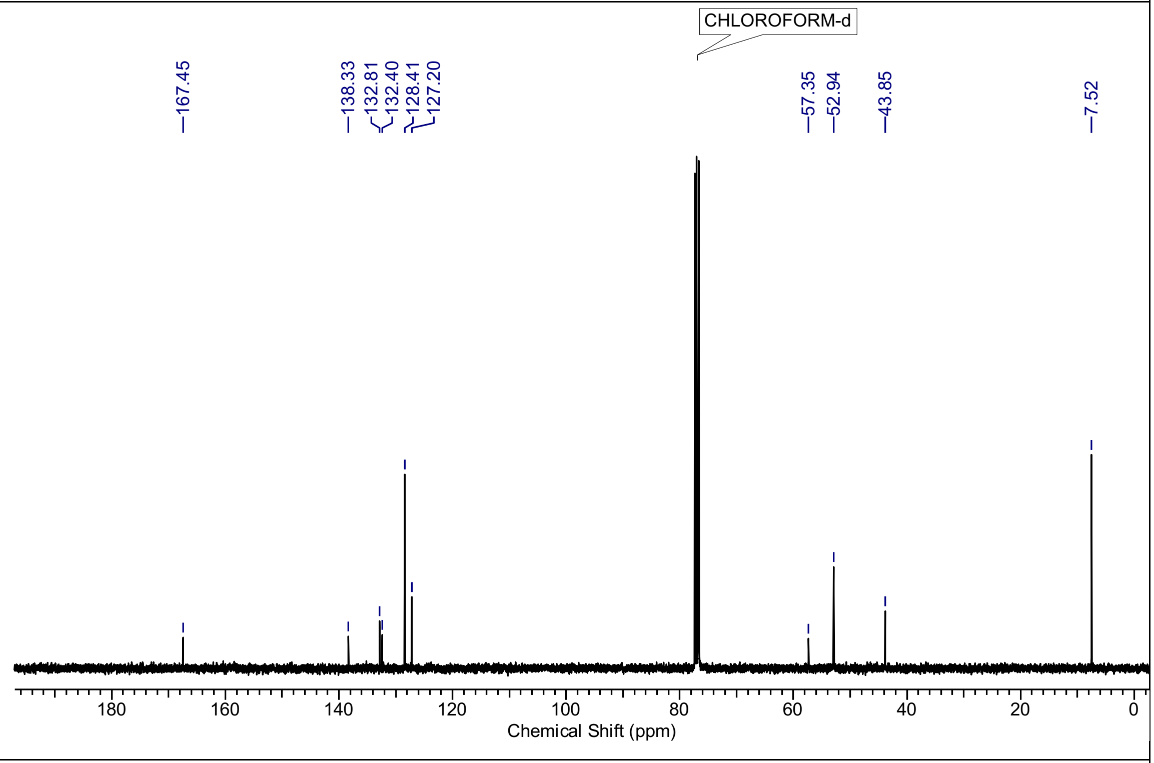

1H NMR and ^{13}C NMR spectra of **1e ram-1192**

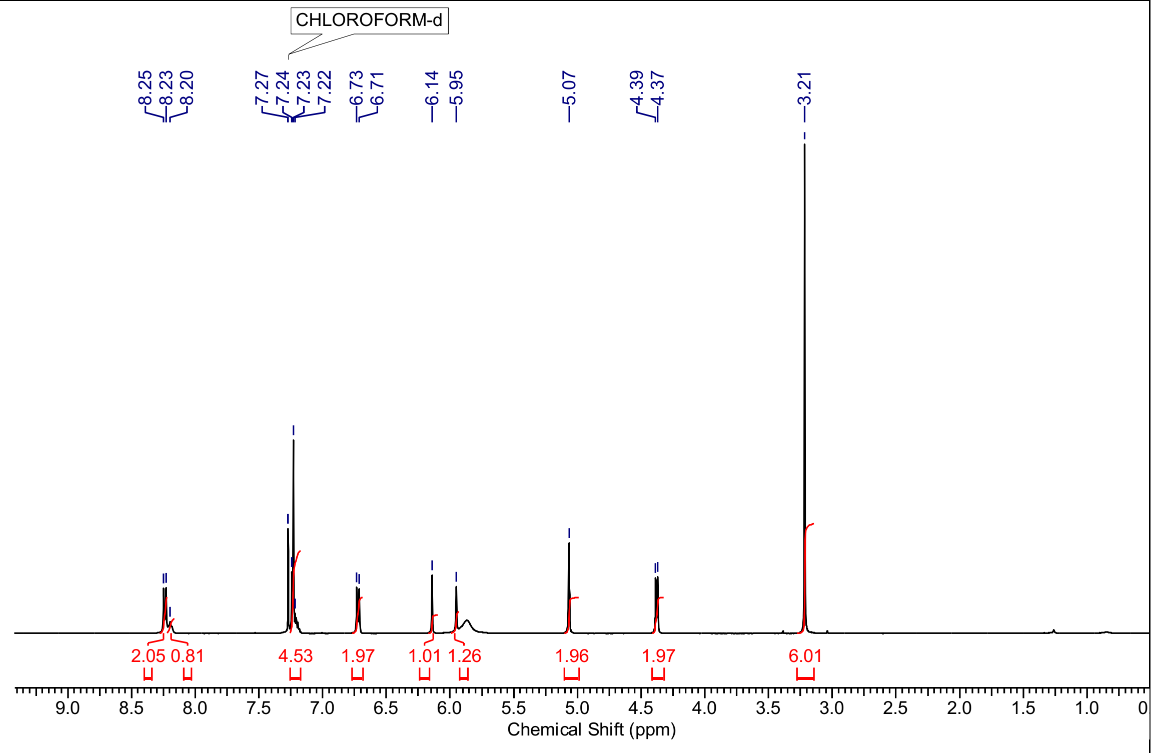

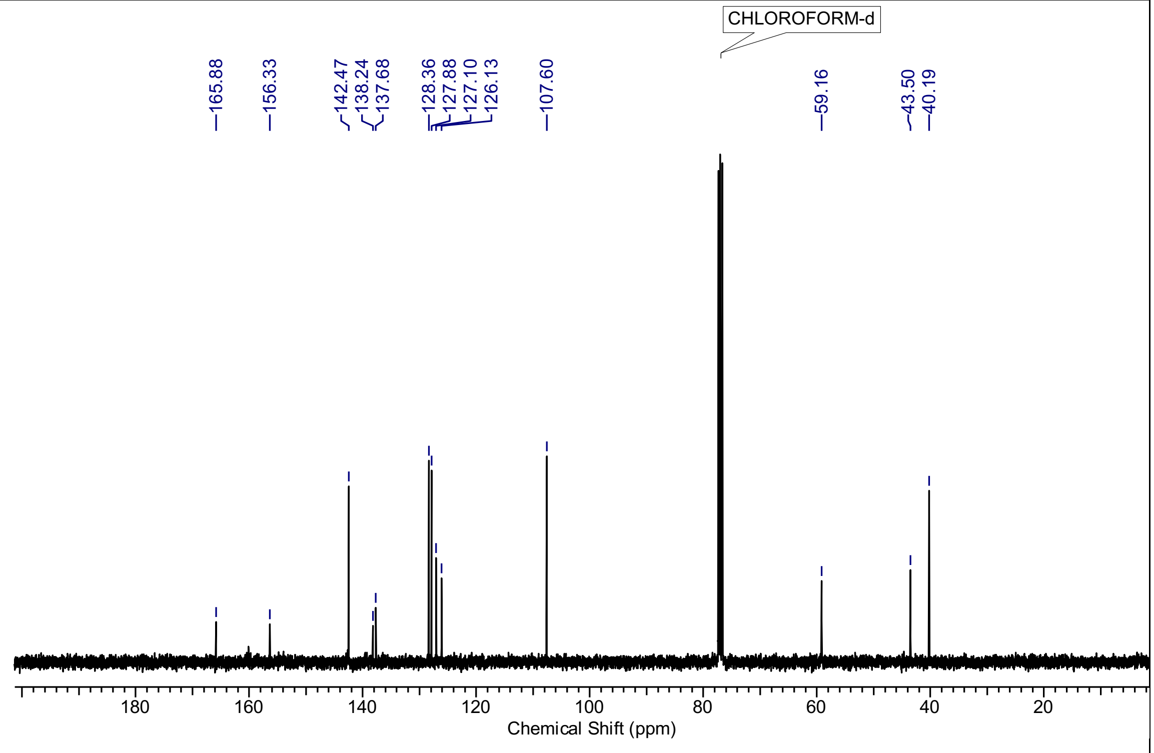

1H NMR and ^{13}C NMR spectra of **1f ram-1191**

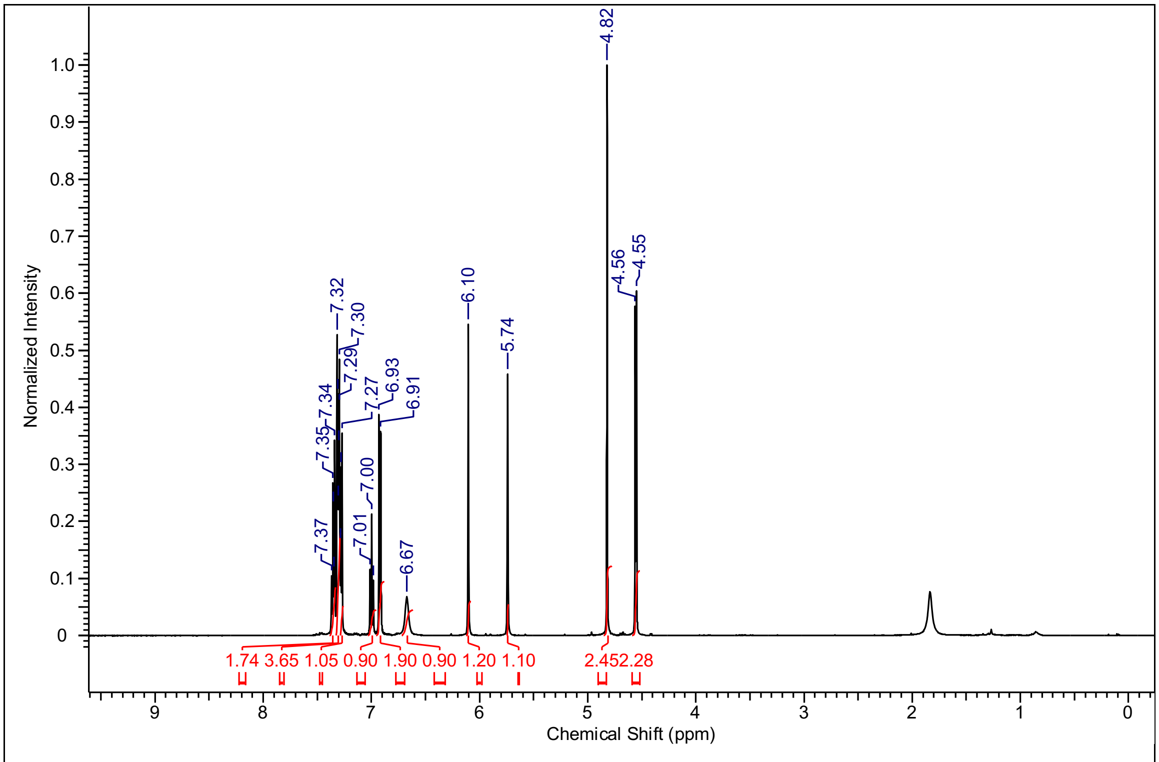

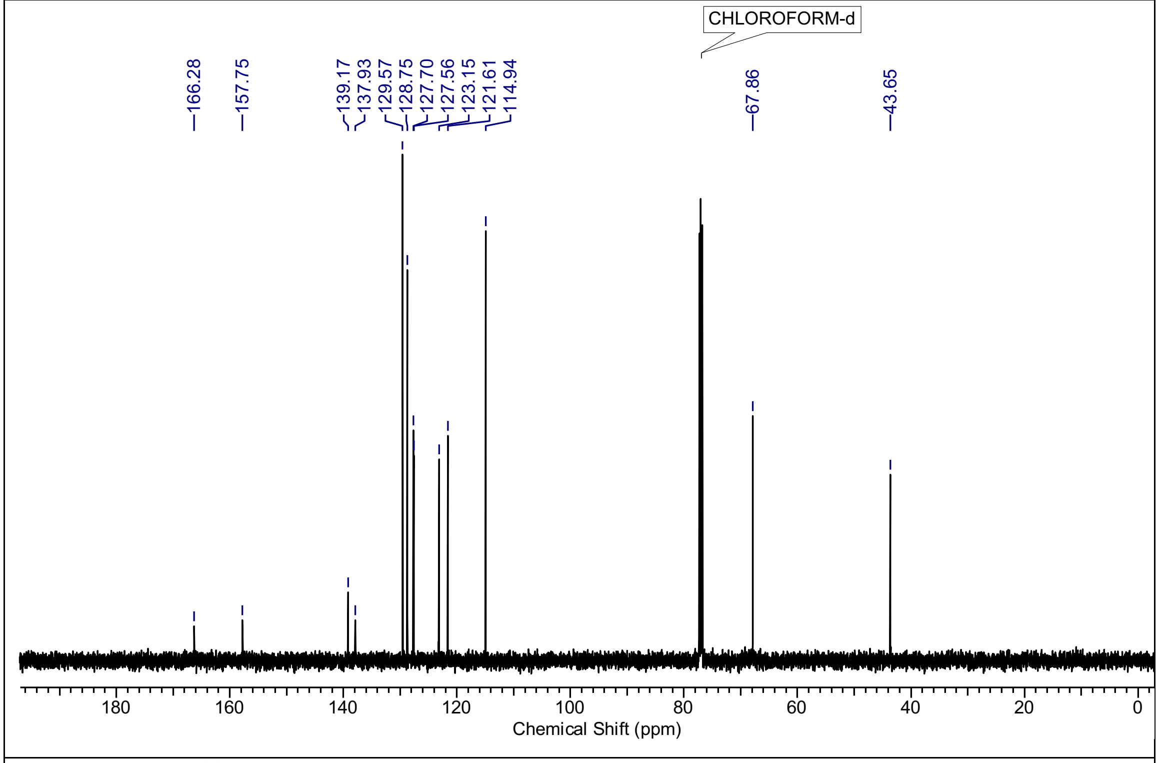

1H NMR and ^{13}C NMR spectra of **ram-768 1g**

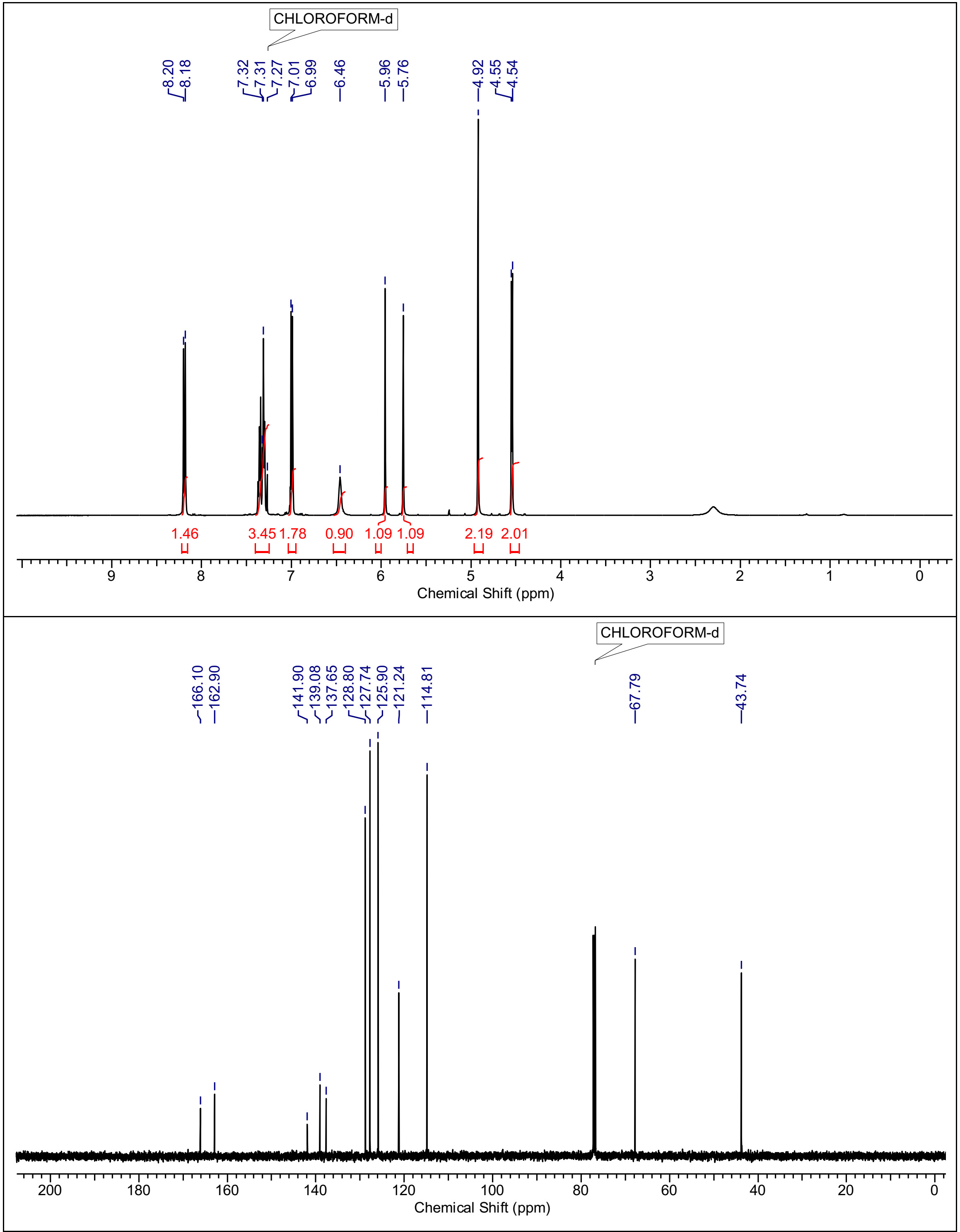

^{1}H NMR and ^{13}C NMR spectra of **ram-728 1h**

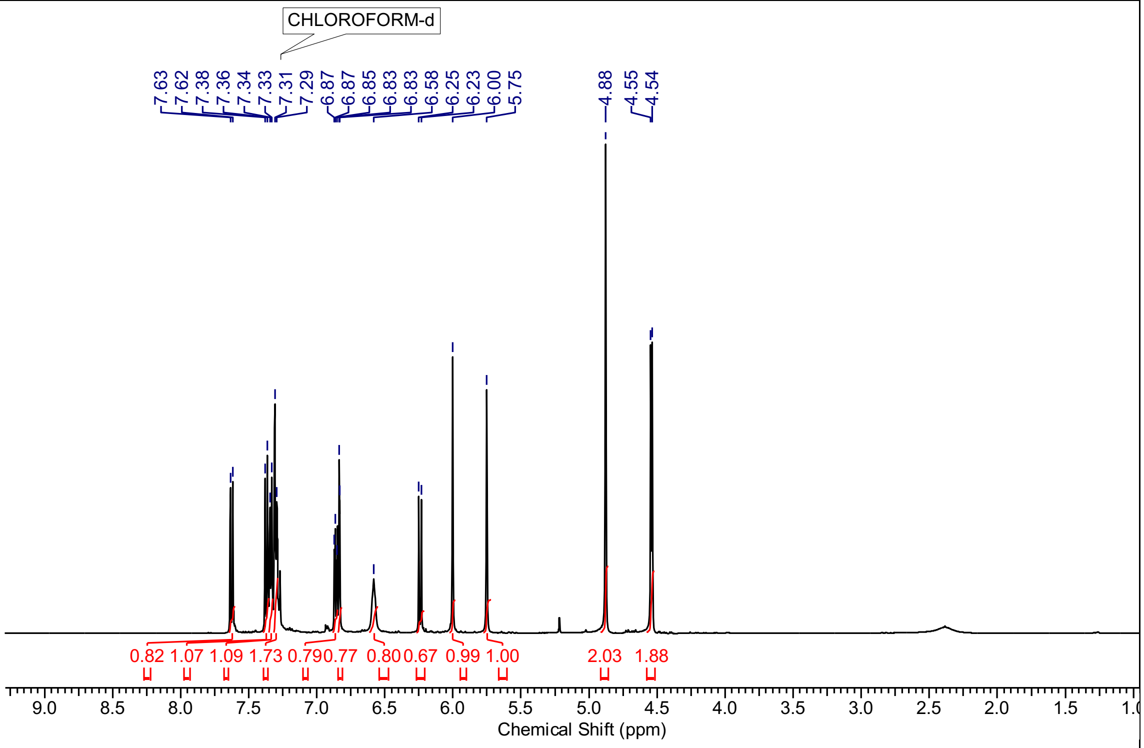

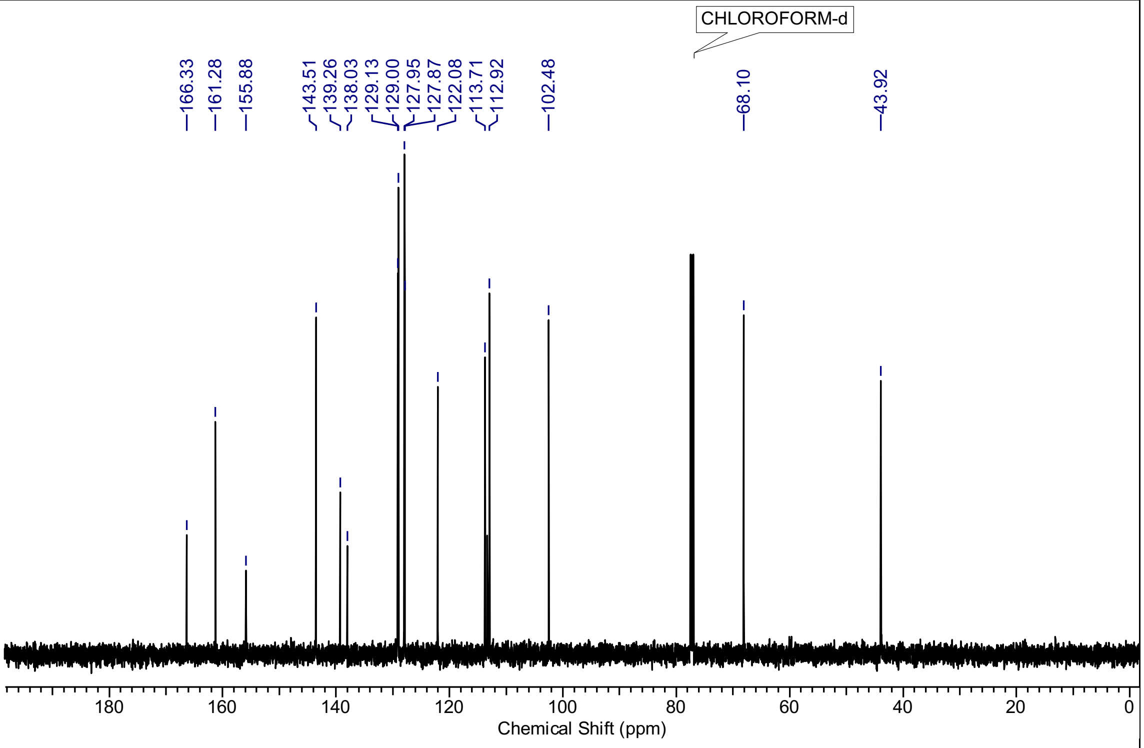

1H NMR and ^{13}C NMR spectra of **ram-729 1i**

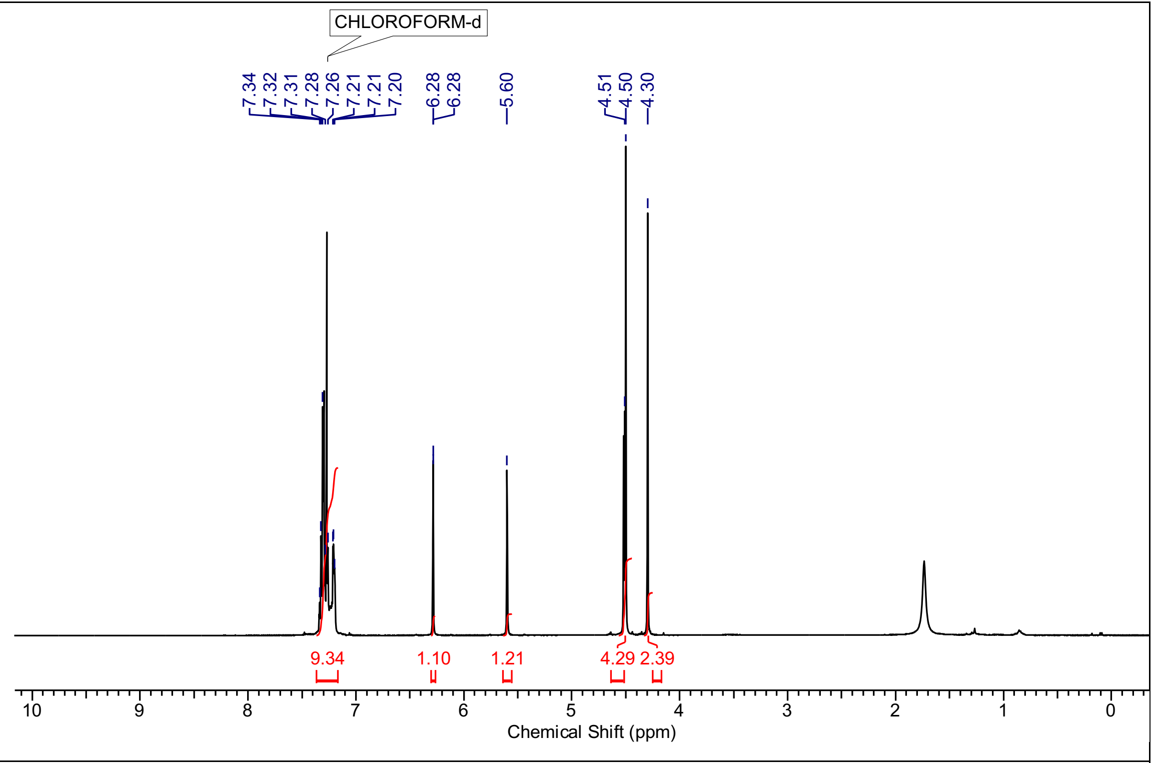

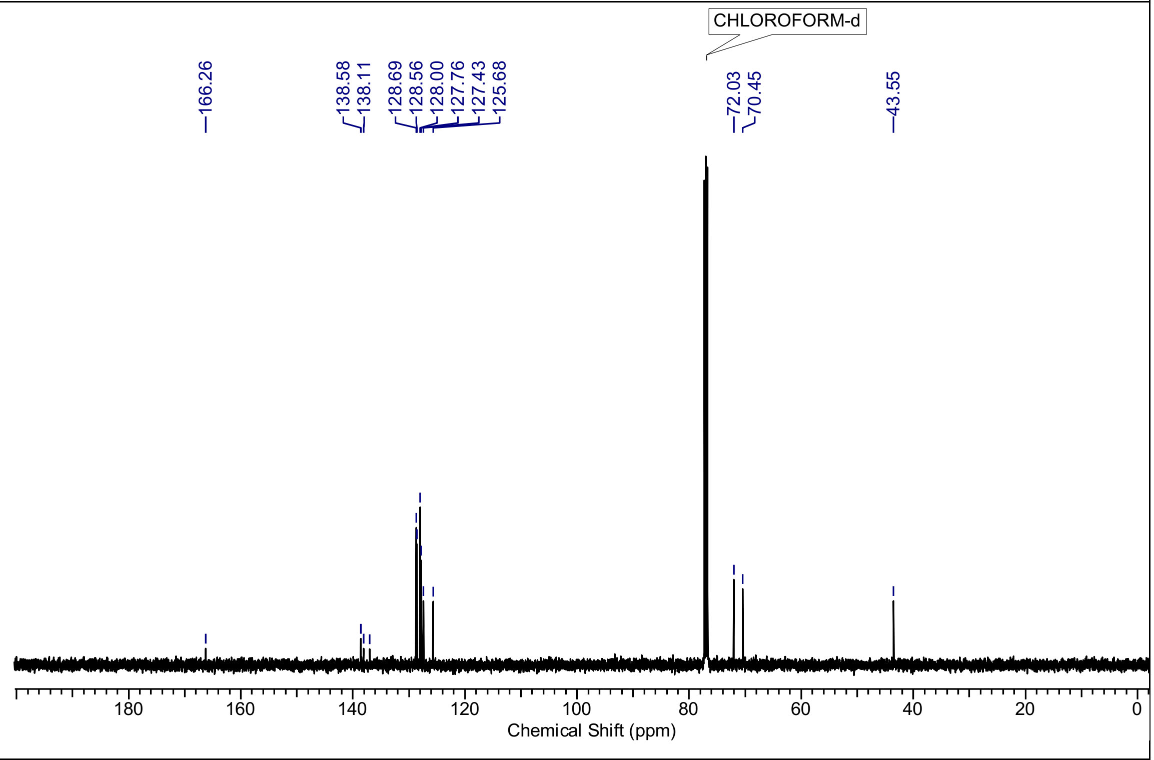

^{1}H NMR and ^{13}C NMR spectra of **1j (ram-711)**

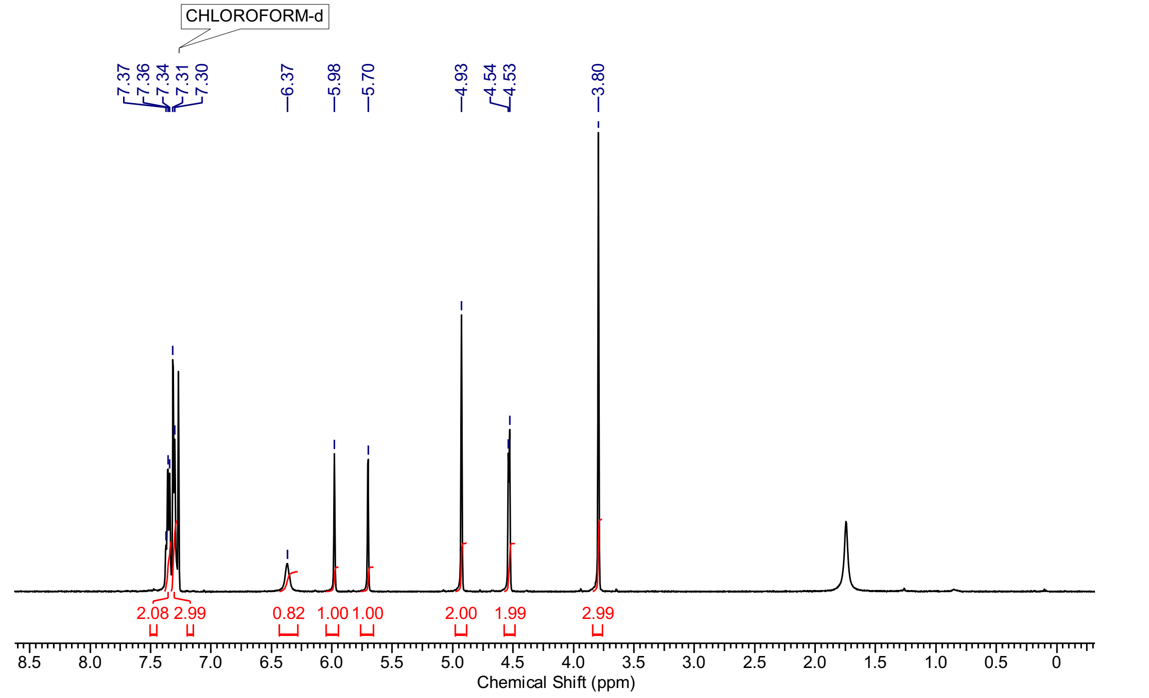

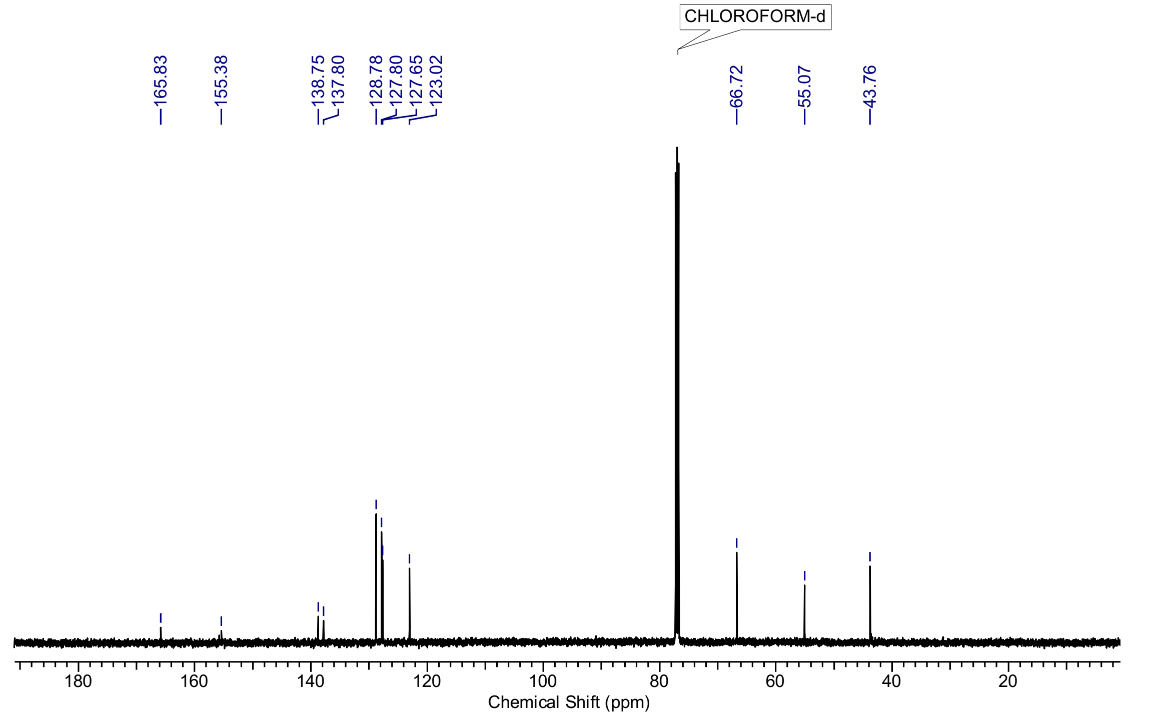

1H NMR and ^{13}C NMR spectra of **ram-1190 1k**

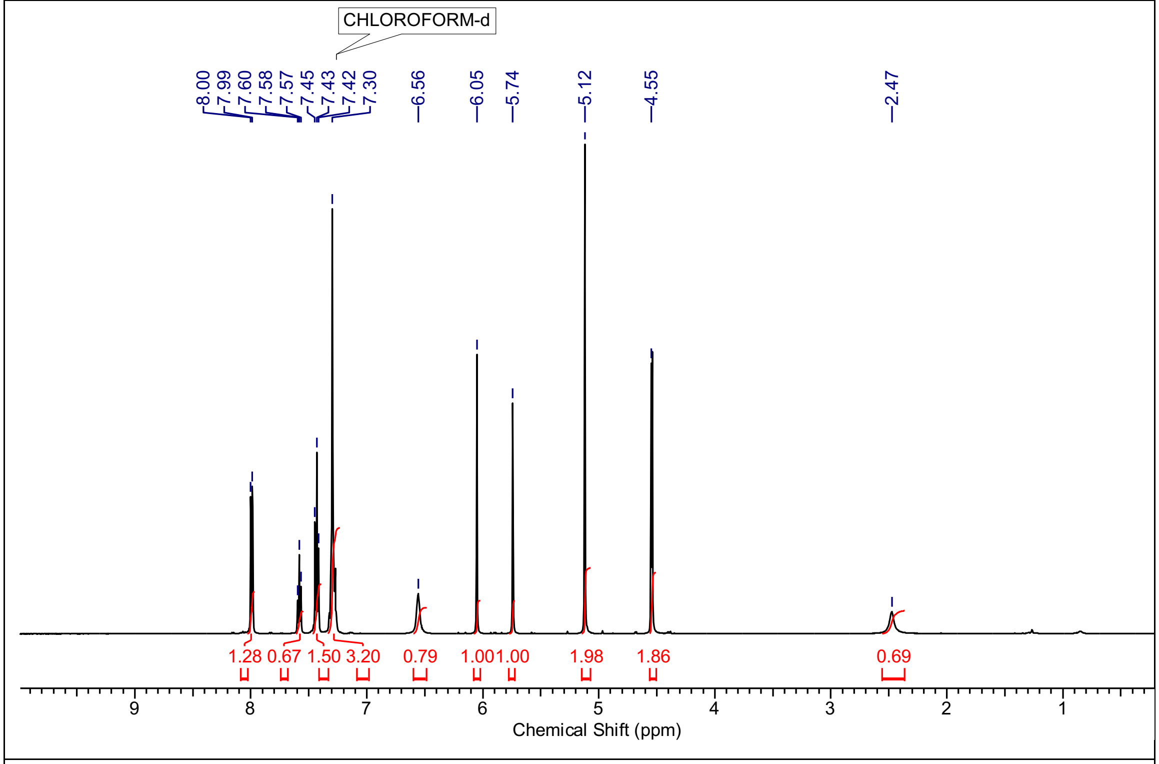

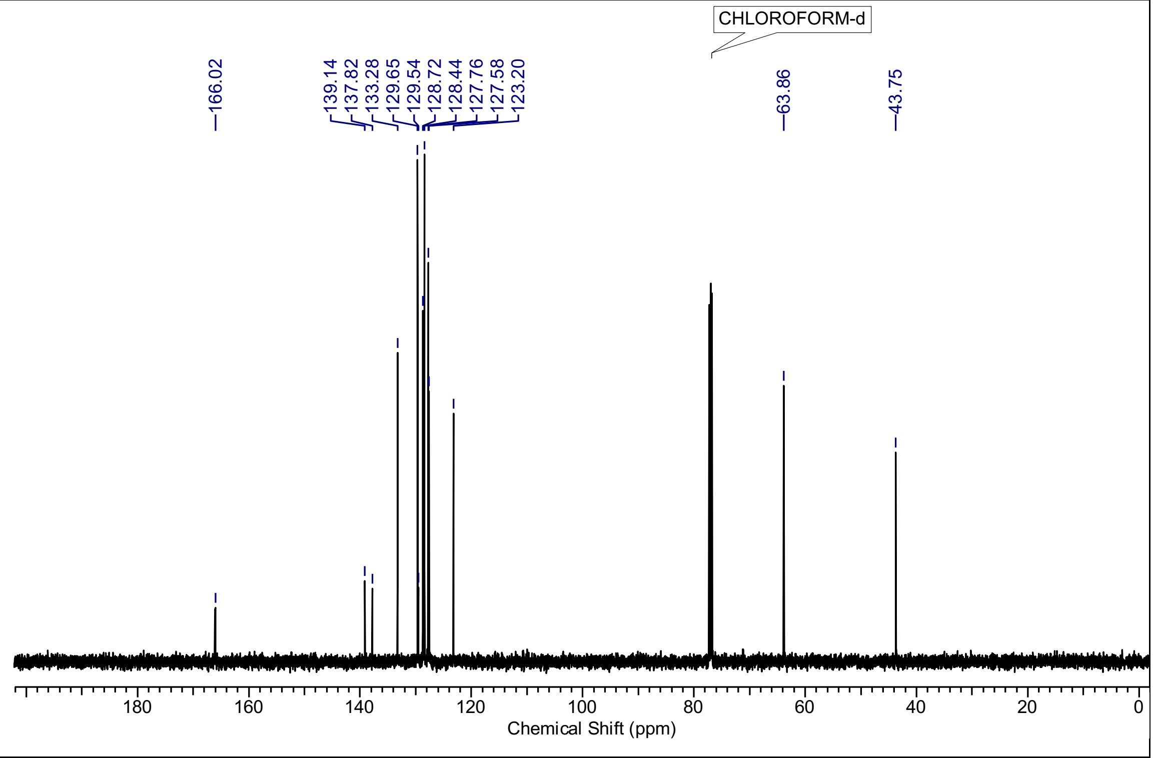

1H NMR and ^{13}C NMR spectra of **ram-727- 1l**

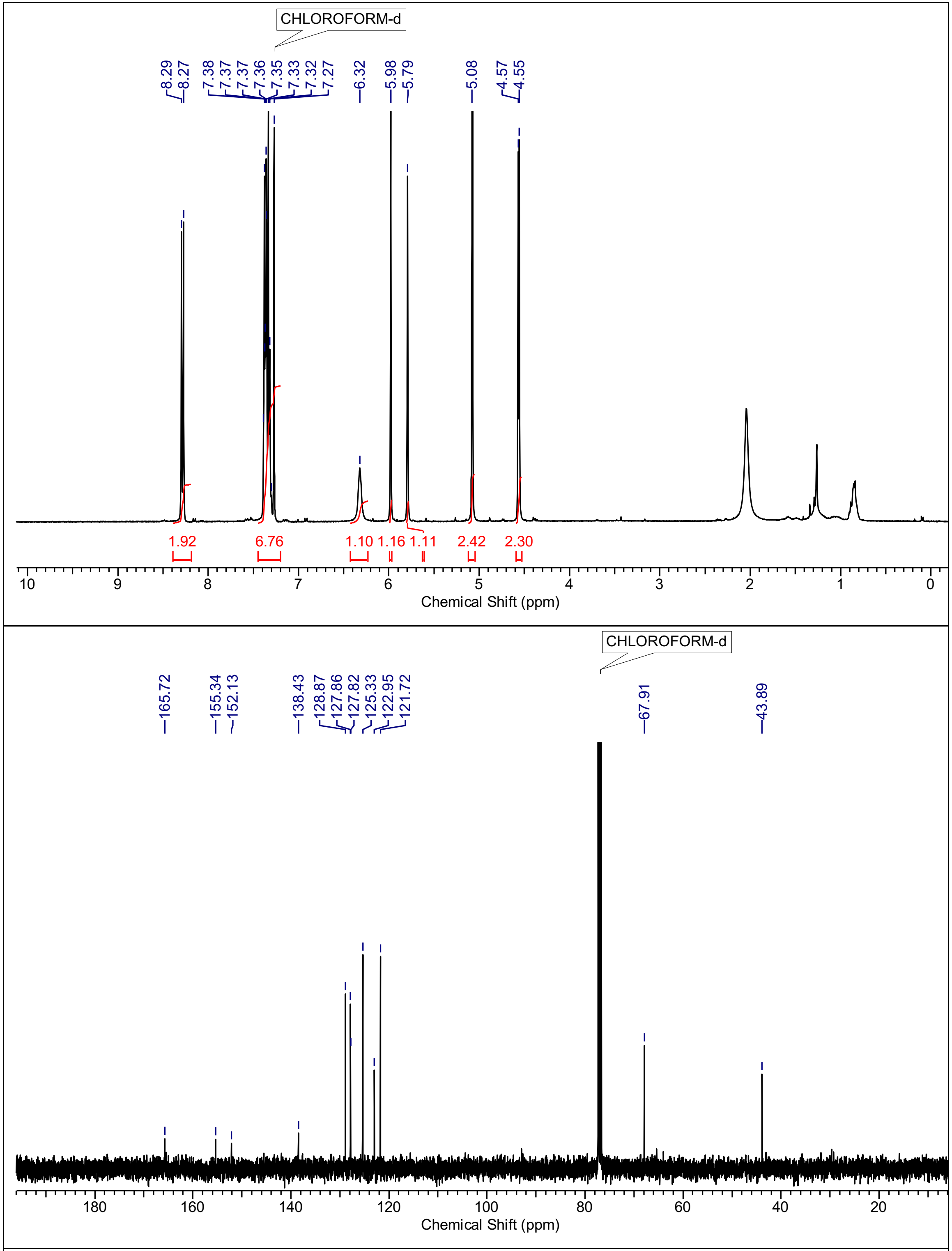

1H NMR and ^{13}C NMR spectra of Ram-825 **1m**

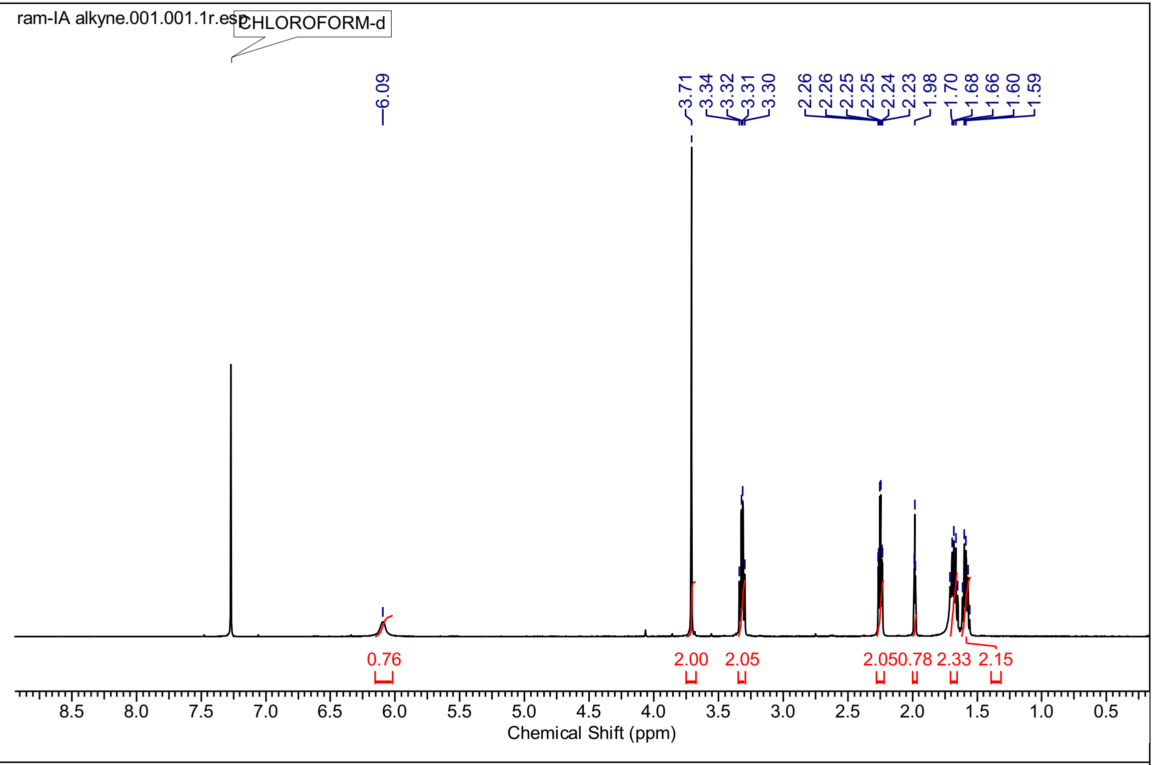

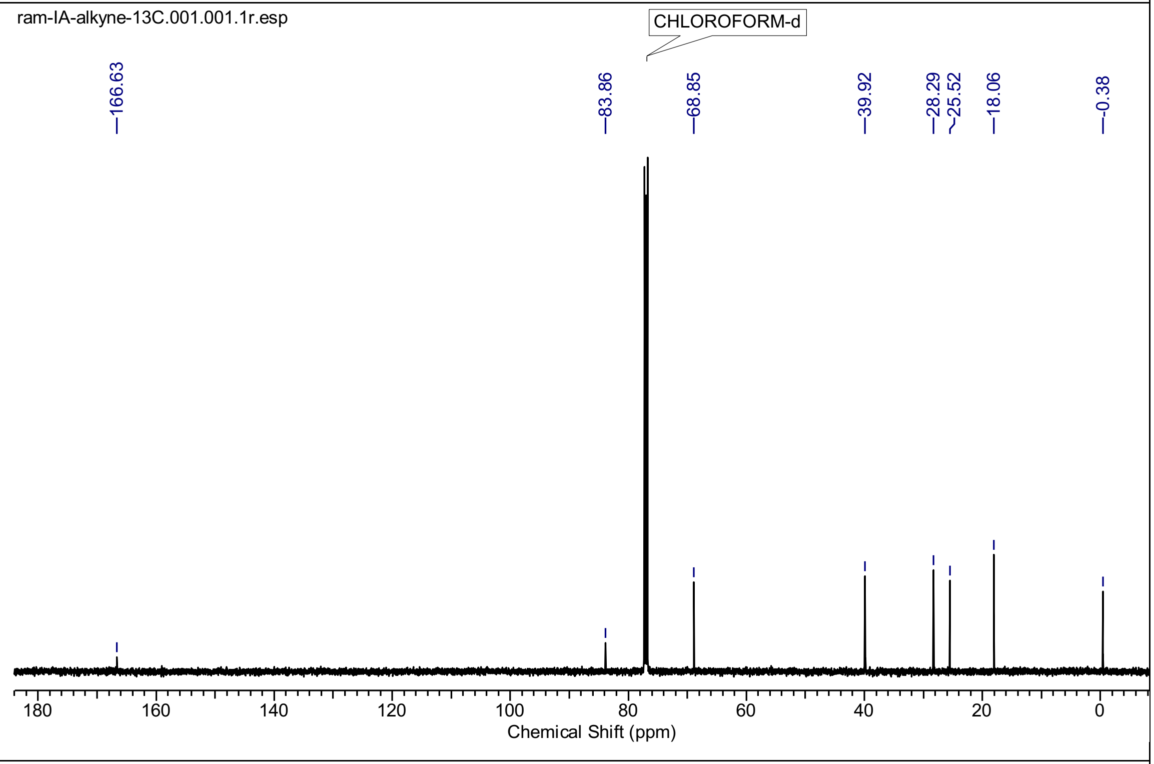

1H NMR and ^{13}C NMR spectra of **IA-alkyne**

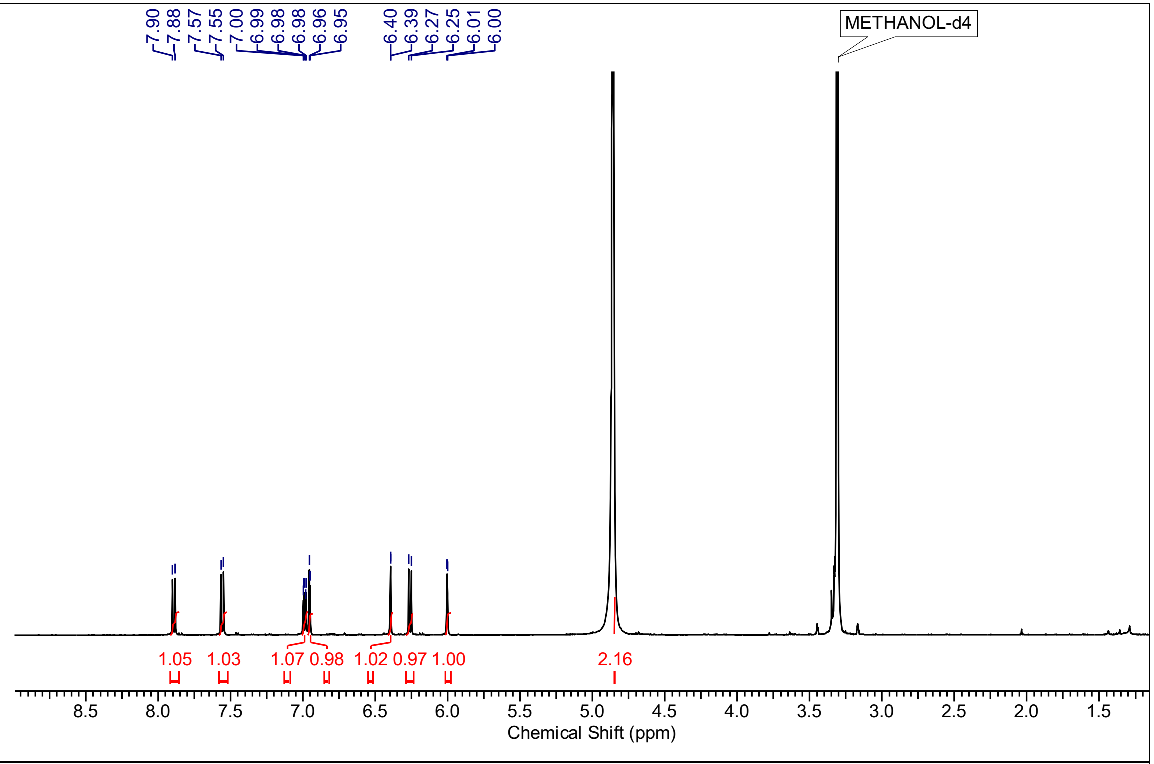

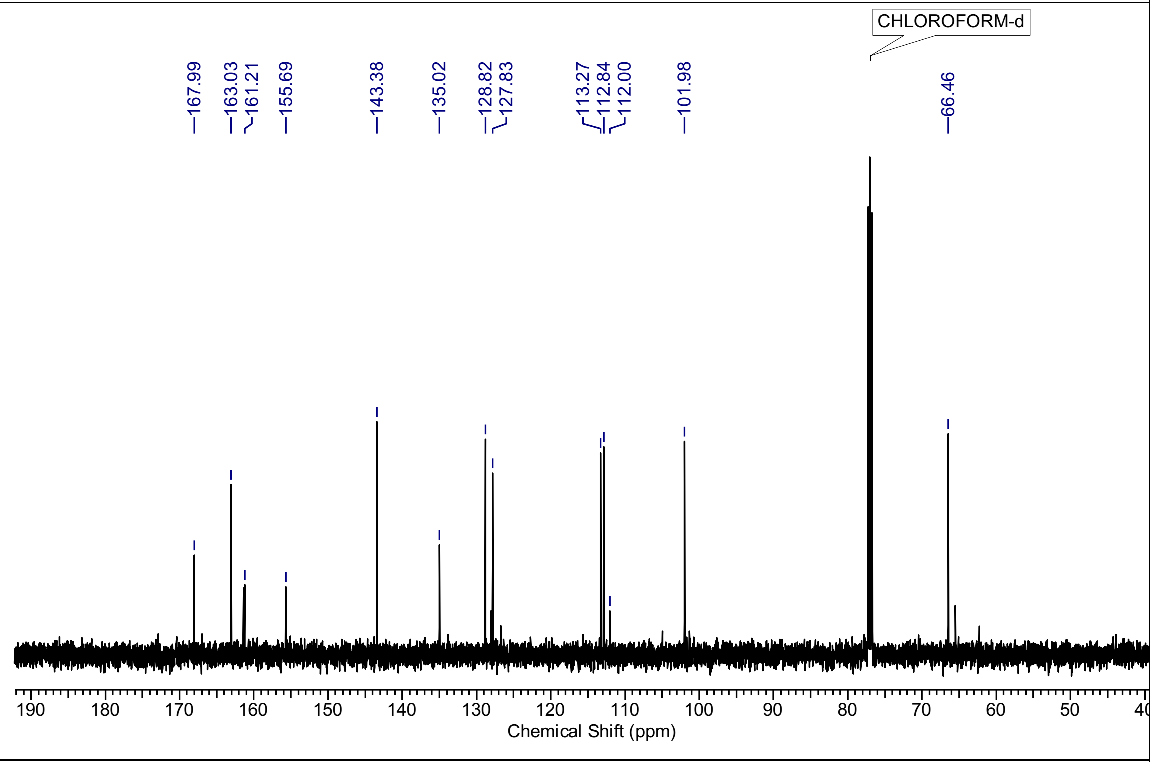

1H NMR and ^{13}C NMR spectra of **2d**

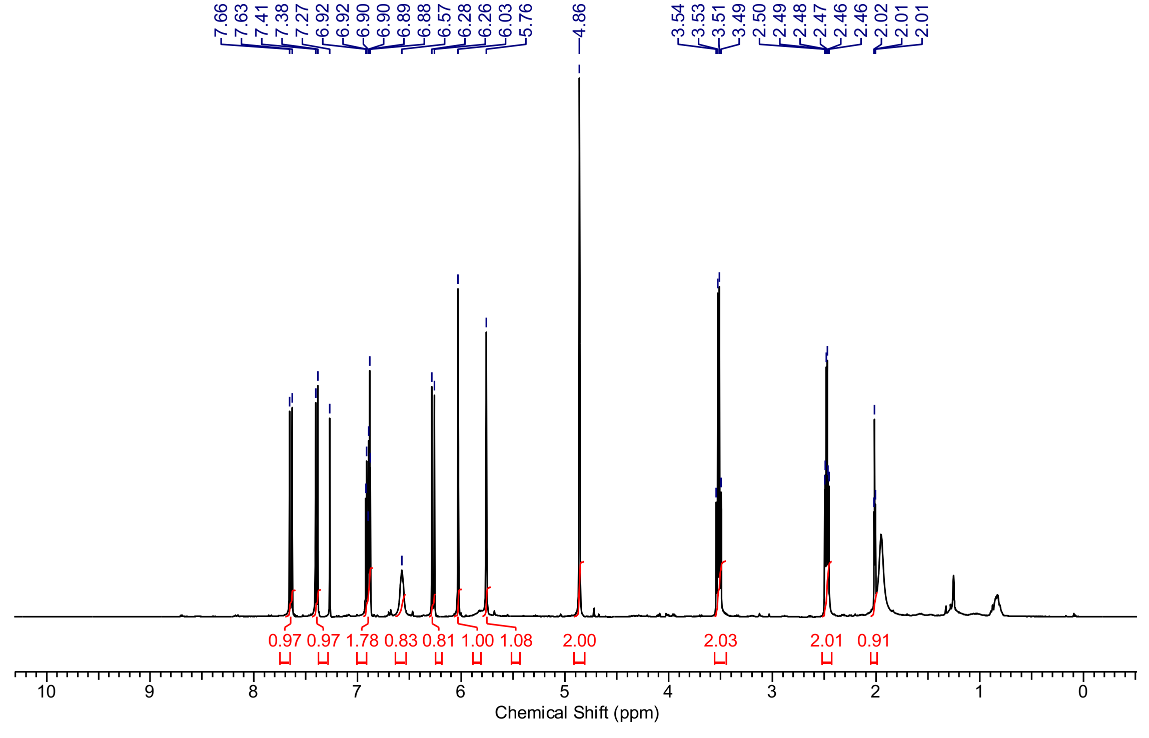

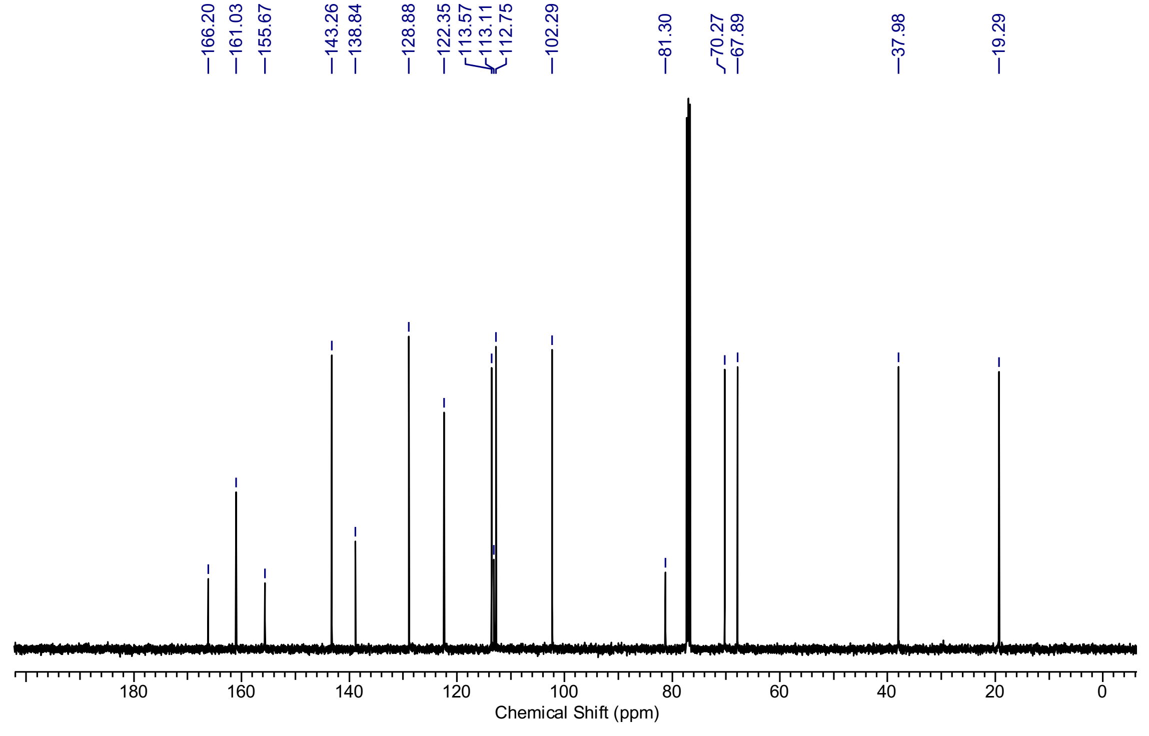

1H NMR and ^{13}C NMR spectra of **ram-938 2a**

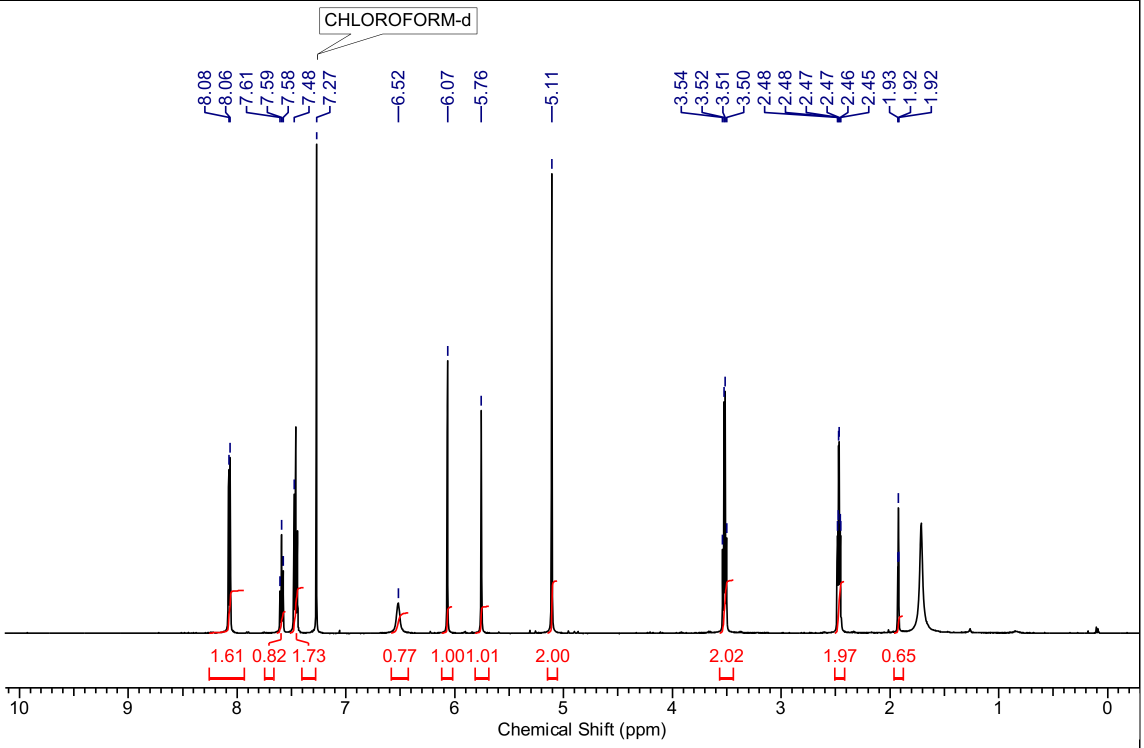

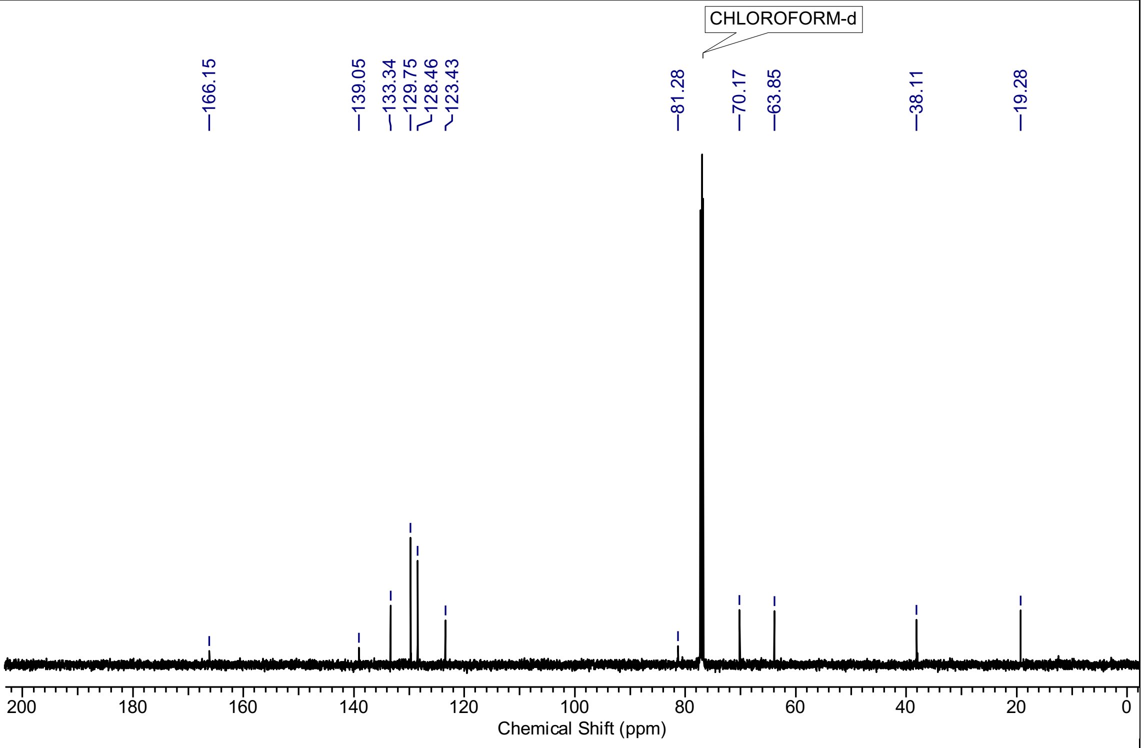

1H NMR and ^{13}C NMR spectra of **ram-1000 2b**

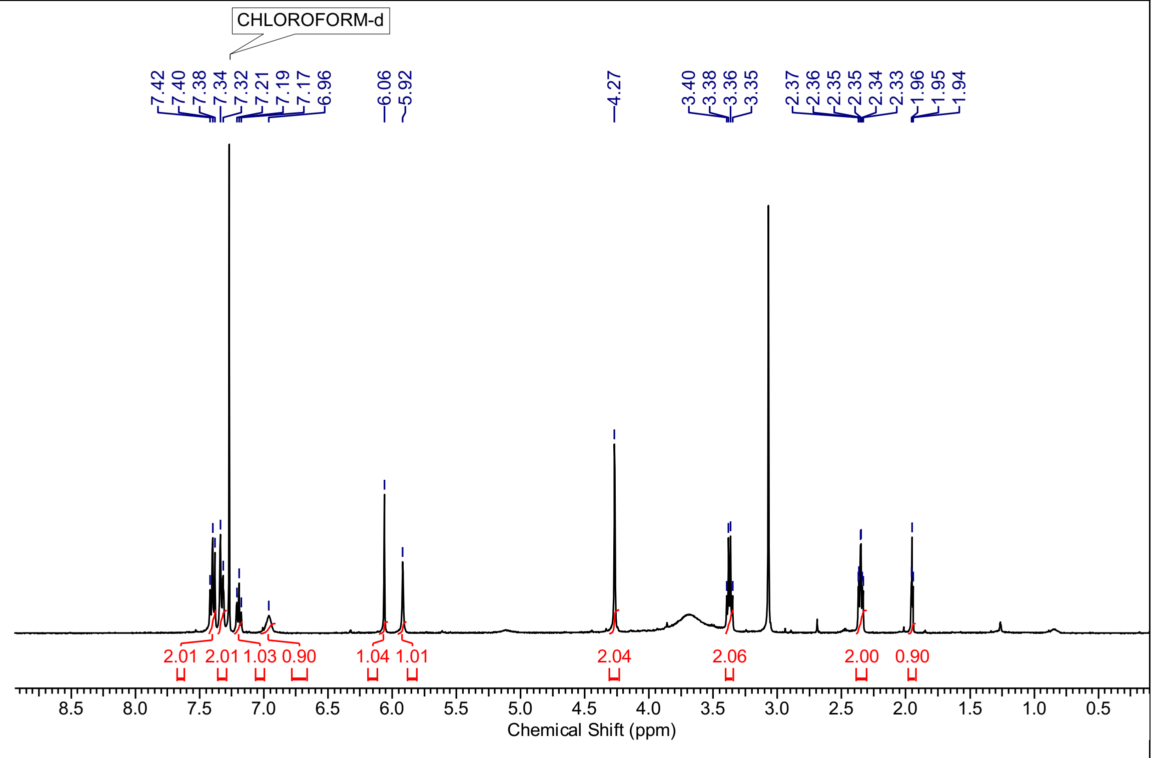

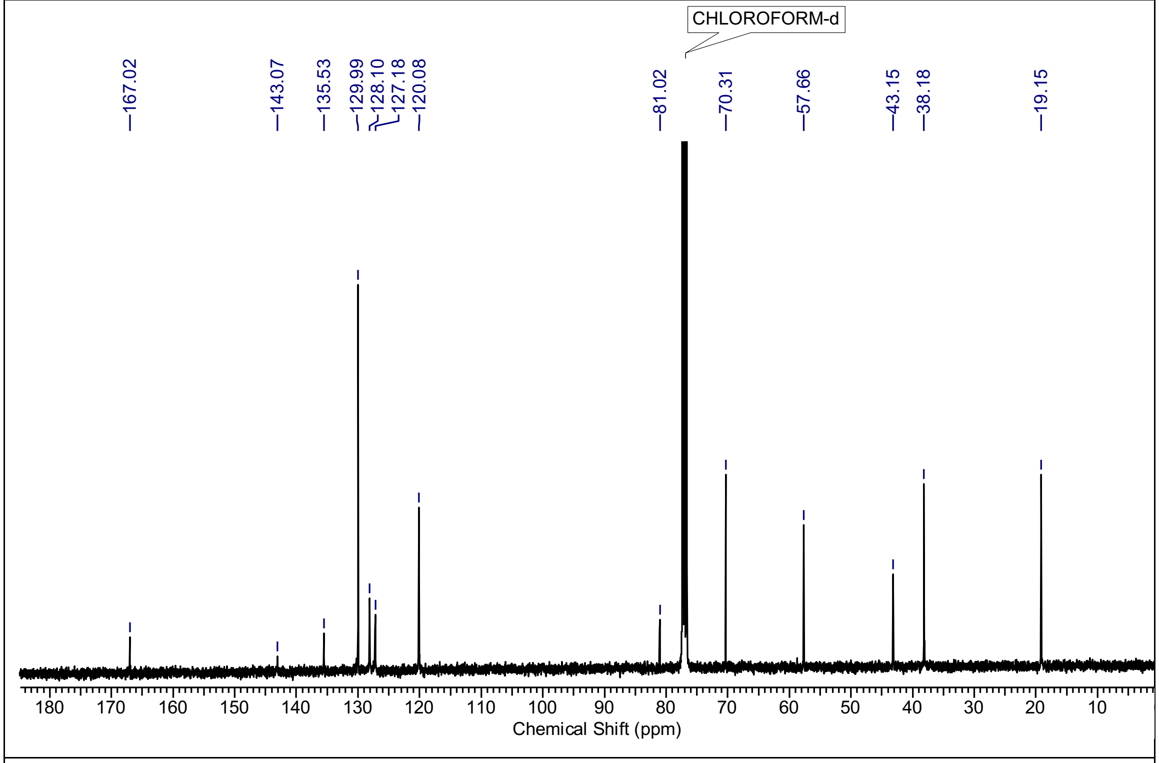

1H NMR and ^{13}C NMR spectra of **ram-999 2c**

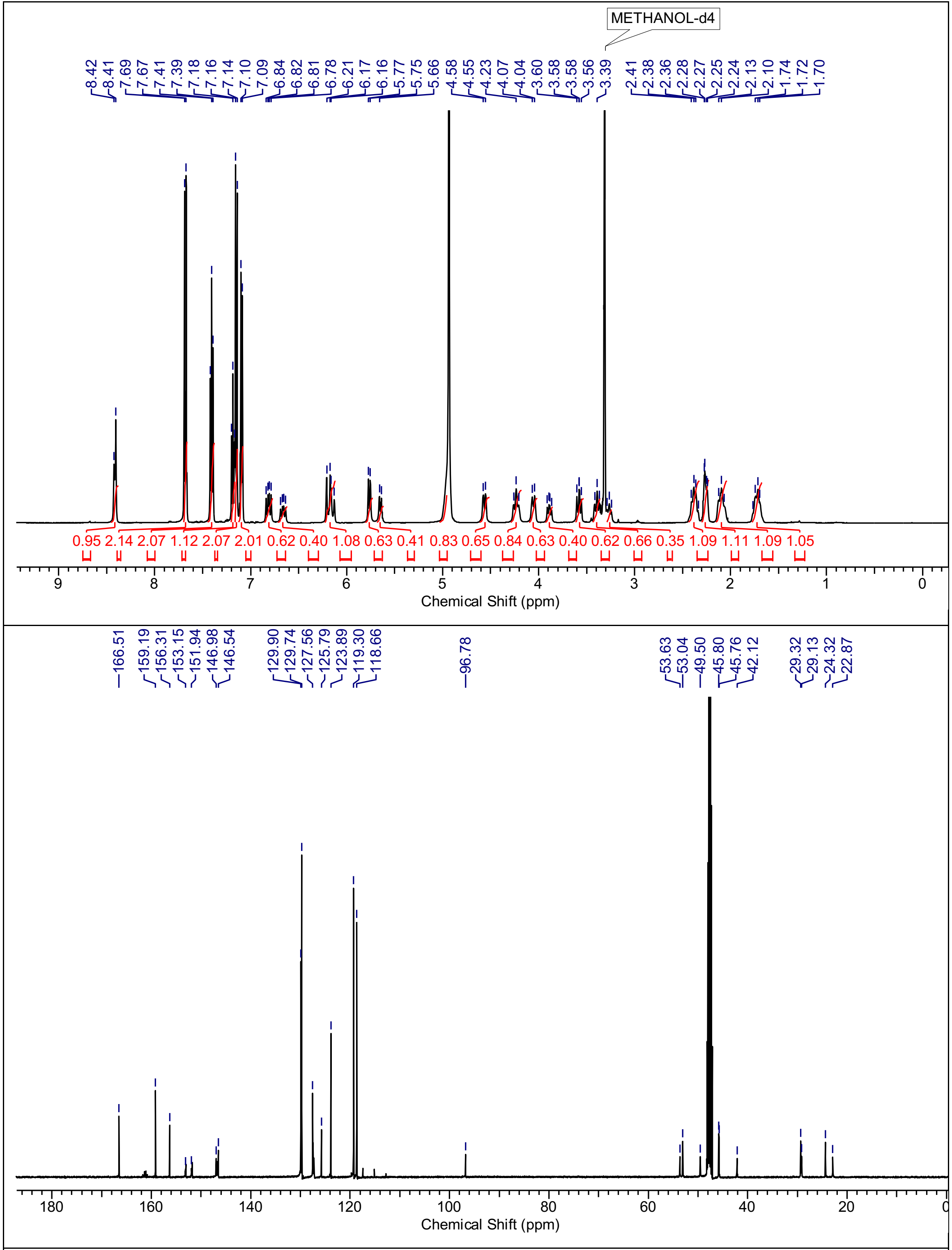

1H NMR and ^{13}C NMR spectra of **Ibrutinib**

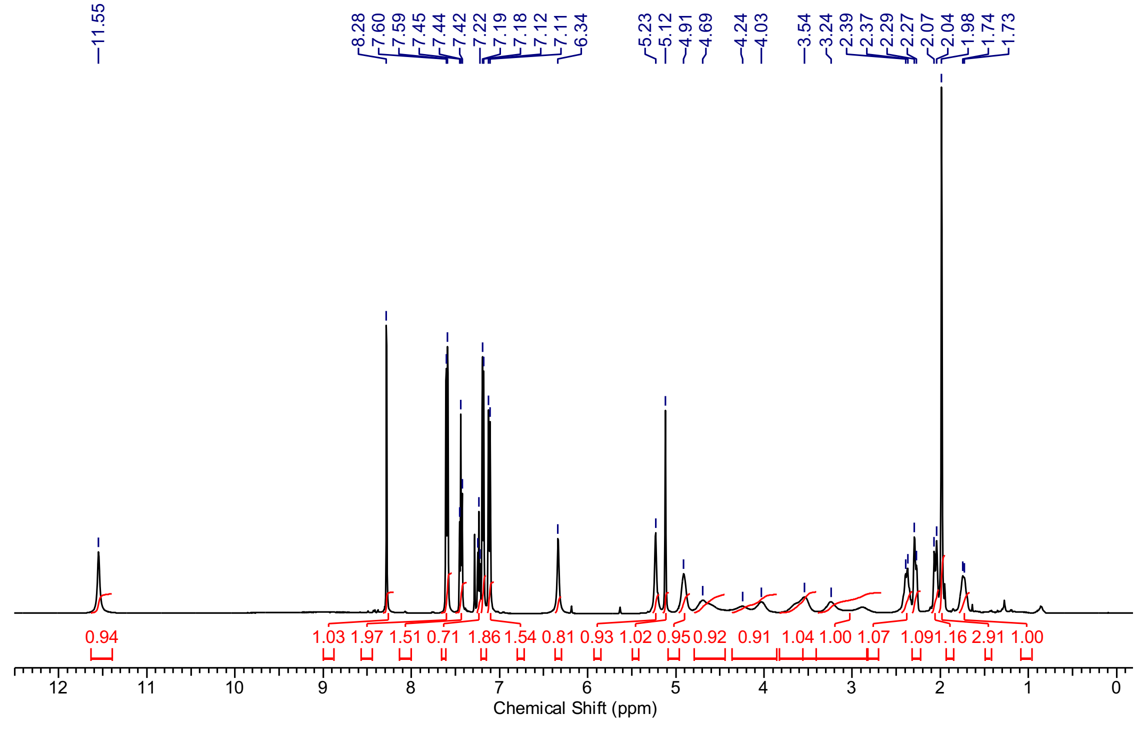

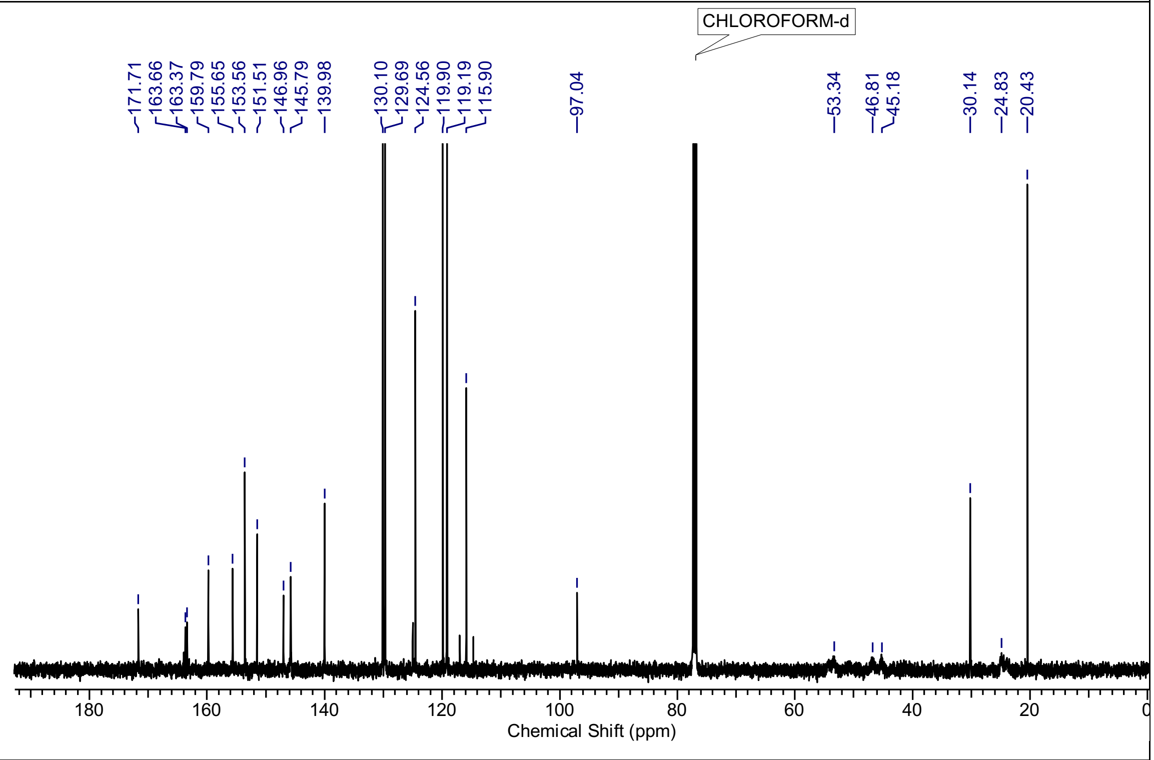

1H NMR and ^{13}C NMR spectra of **ram-788 3a**

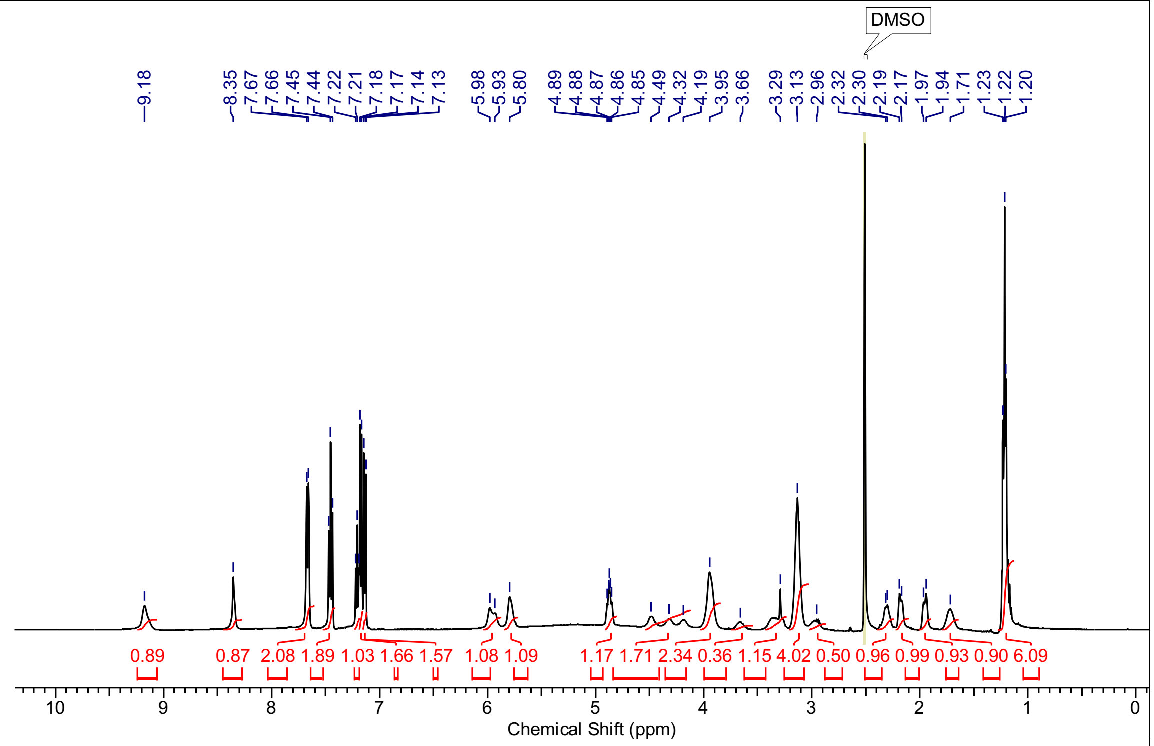

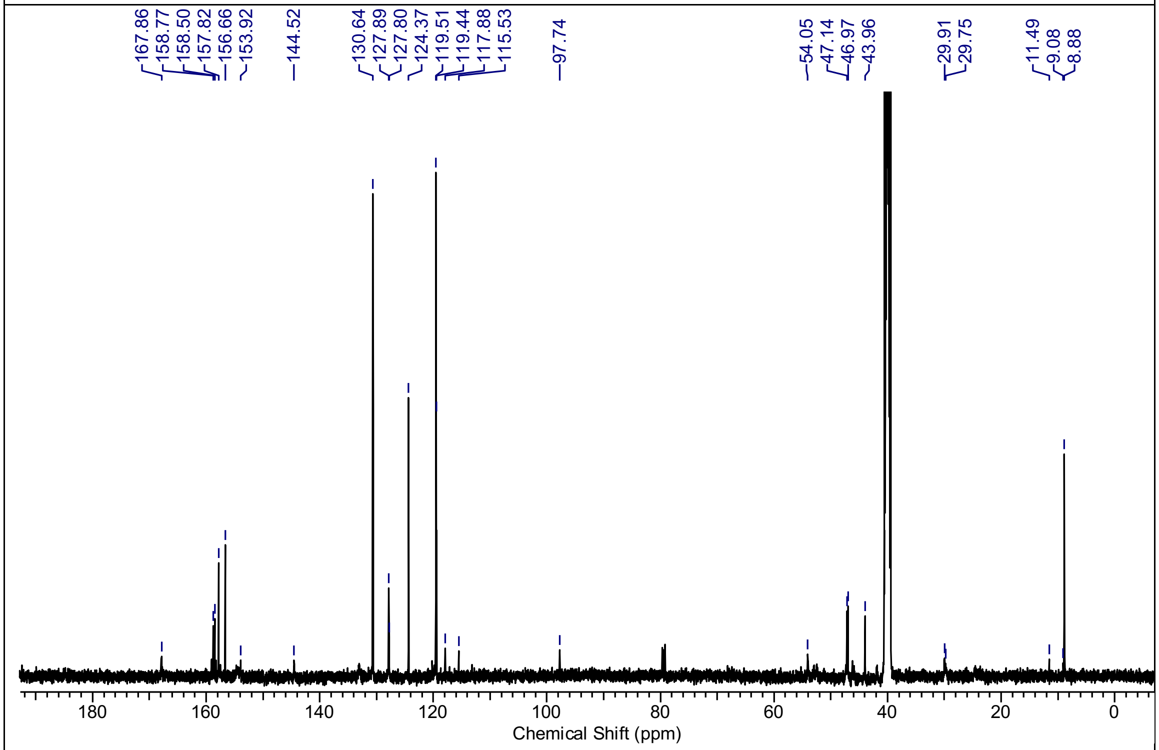

1H NMR and ^{13}C NMR spectra of **ram-888 3b**

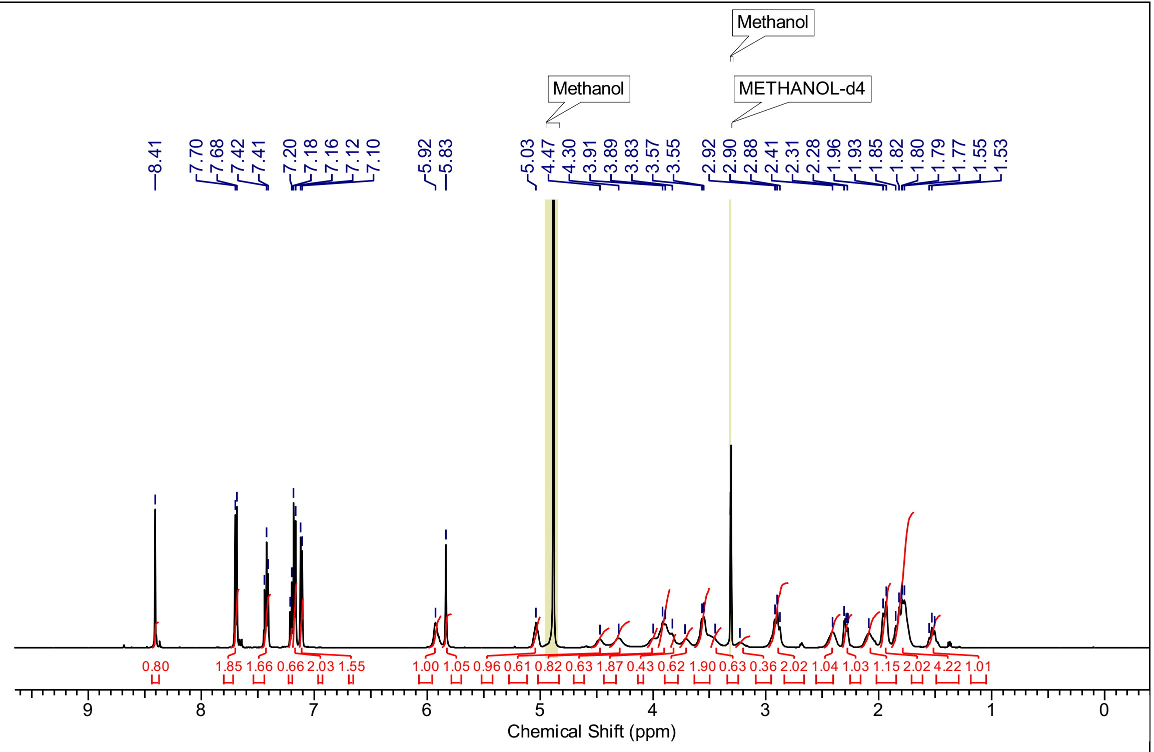

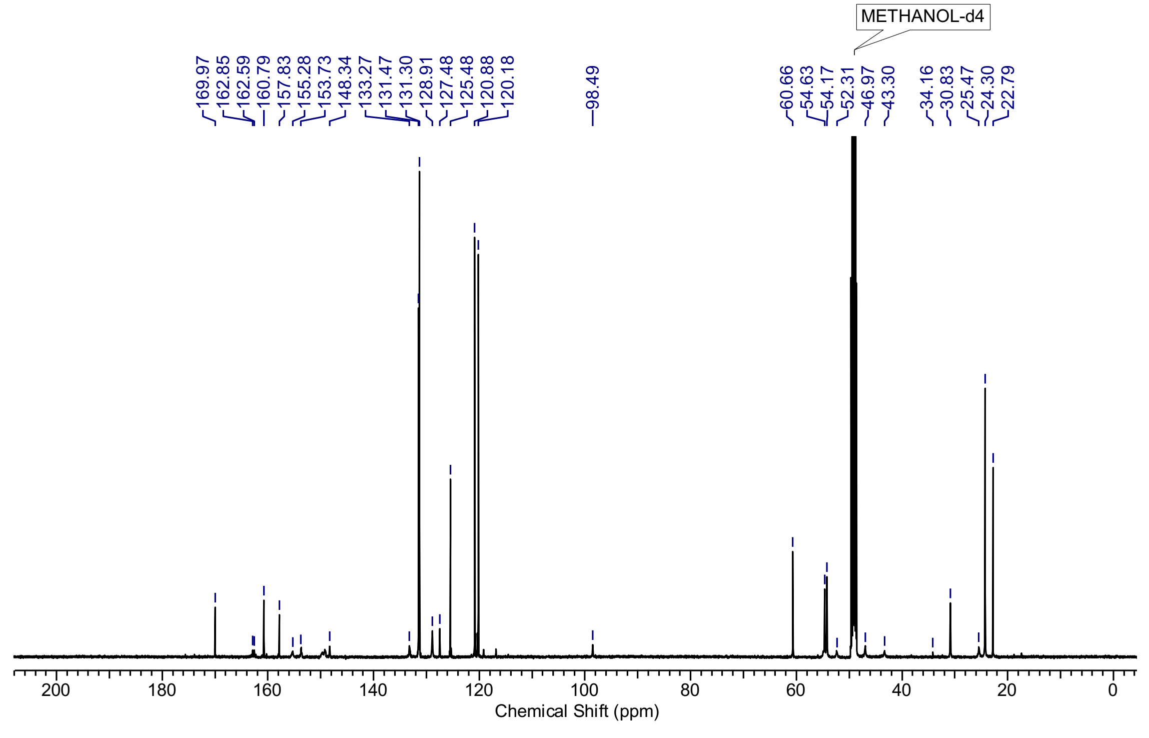

1H NMR and ^{13}C NMR spectra of **ram-890 3c**

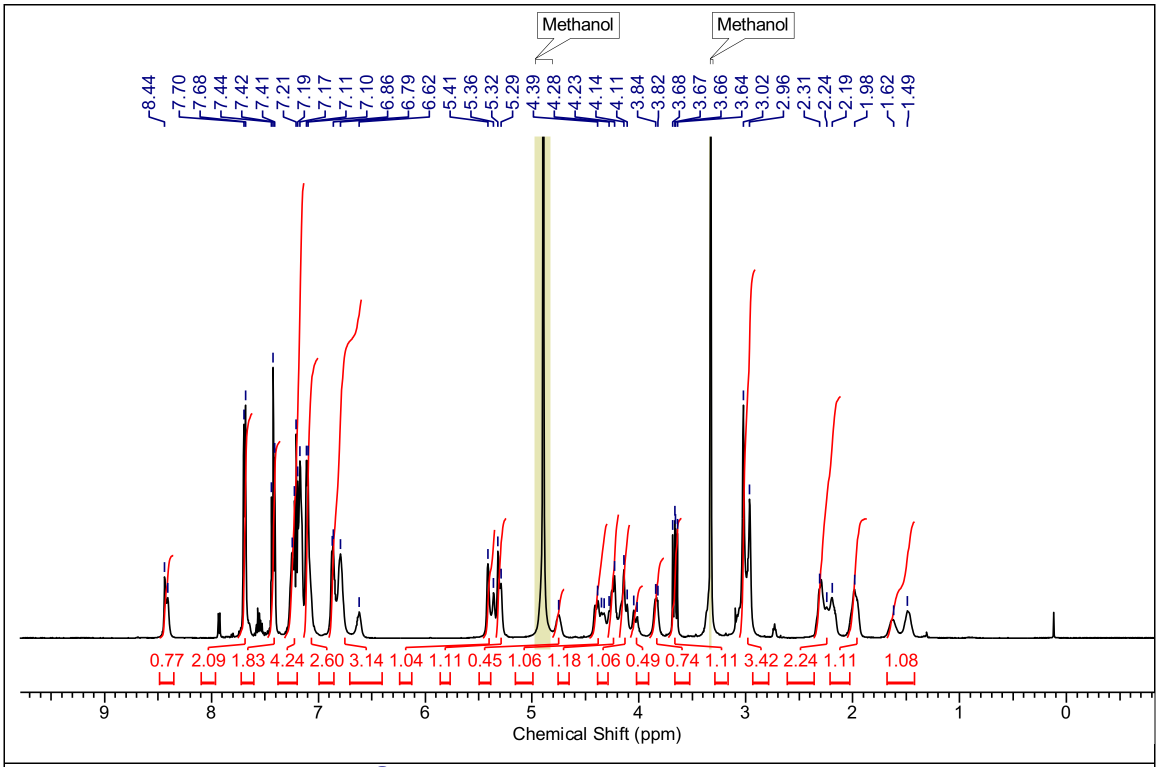

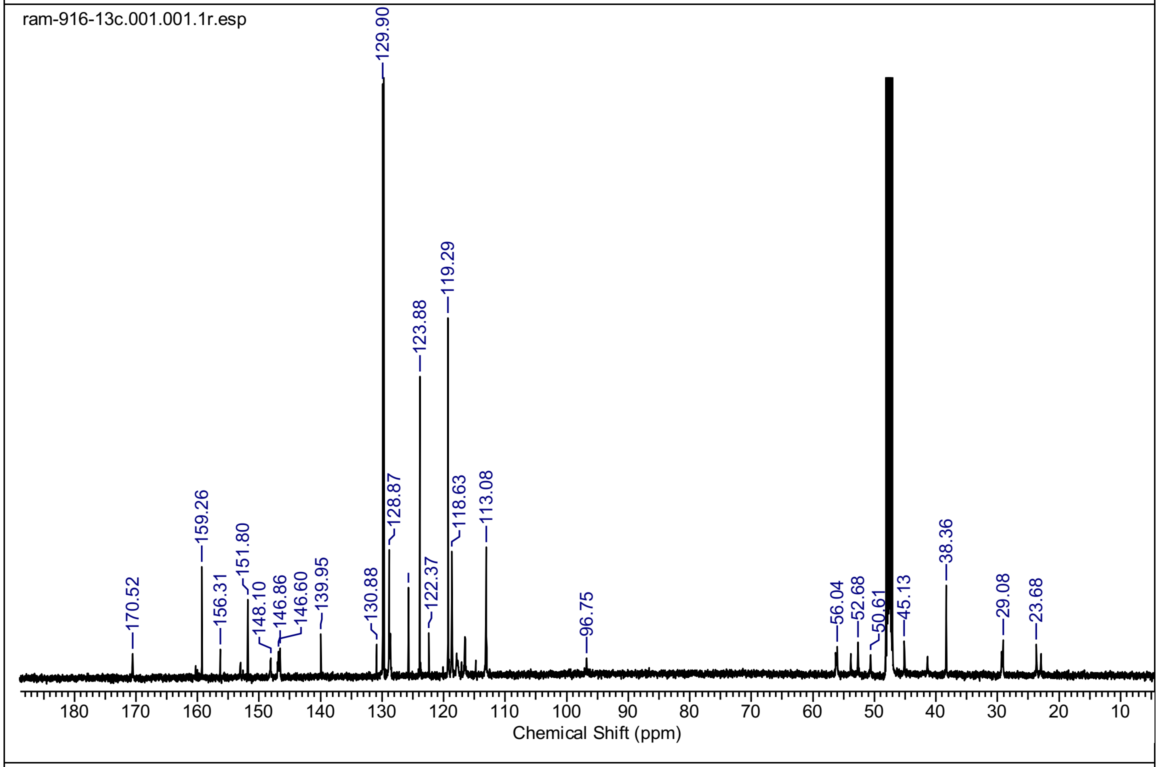

1H NMR and ^{13}C NMR spectra of **ram-916 3d**

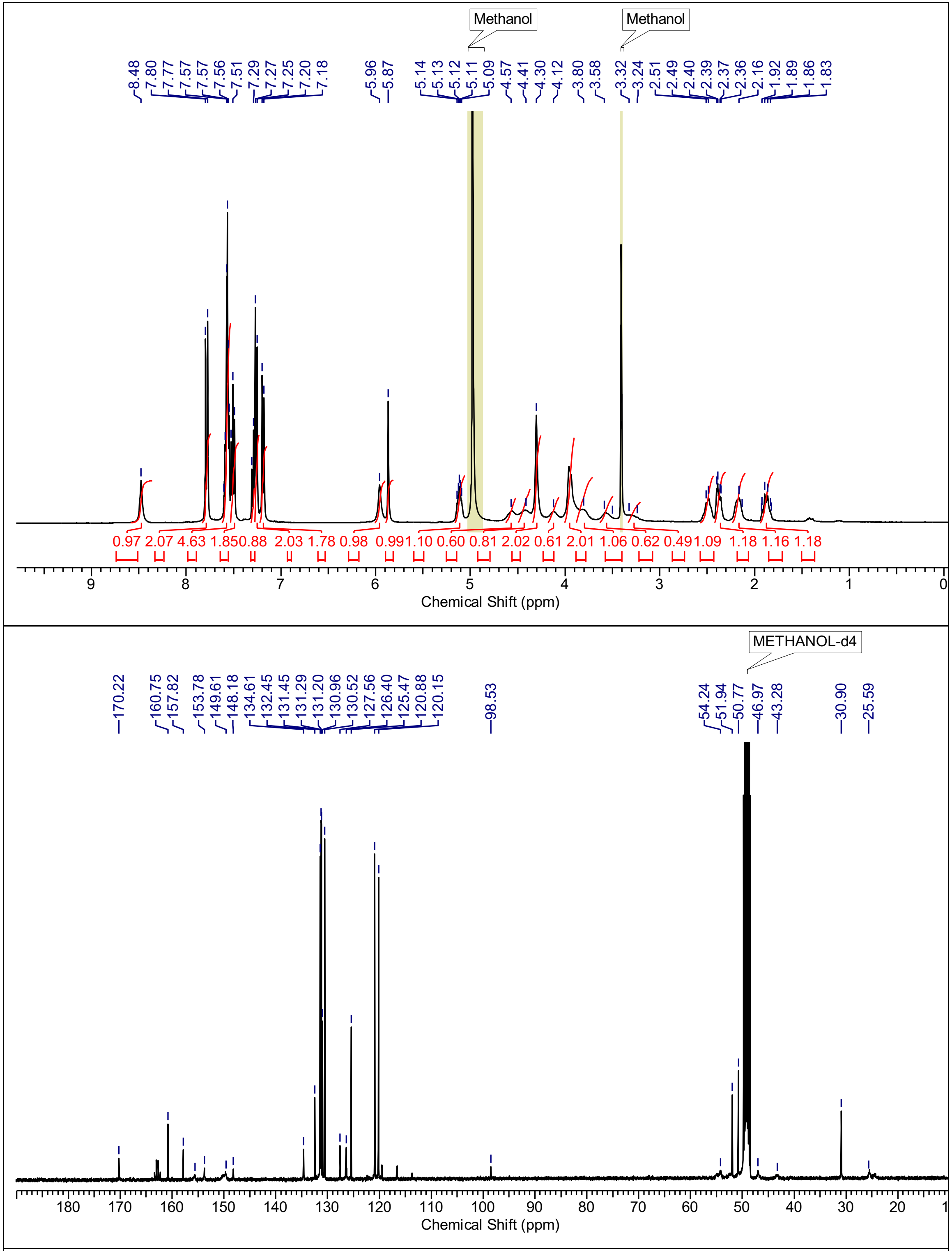

1H NMR and ^{13}C NMR spectra of **ram-917 3e**

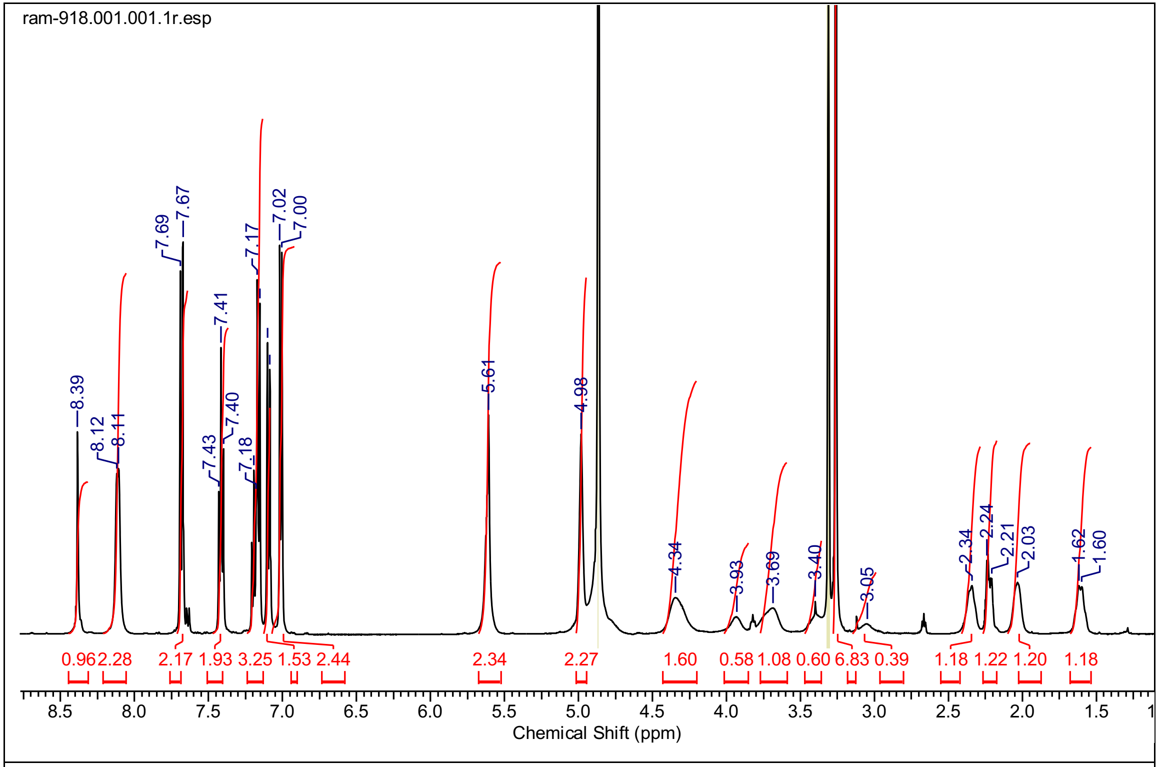

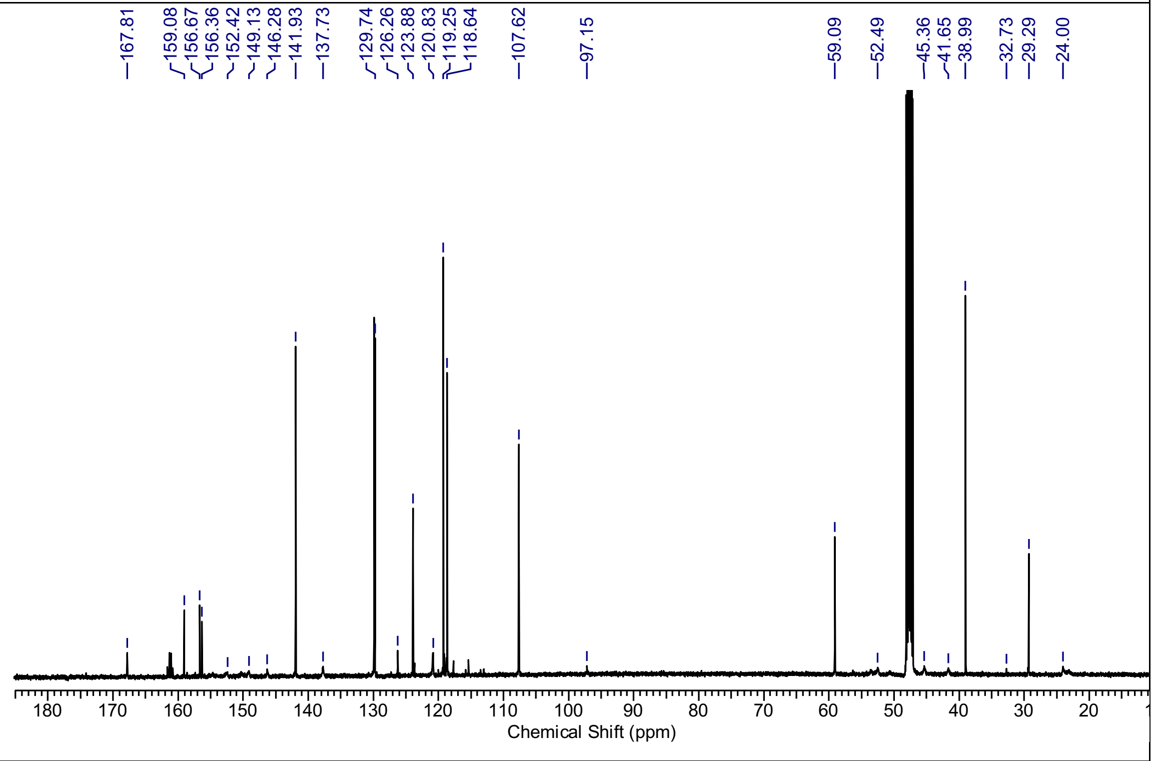

1H NMR and ^{13}C NMR spectra of **ram-918 3f**

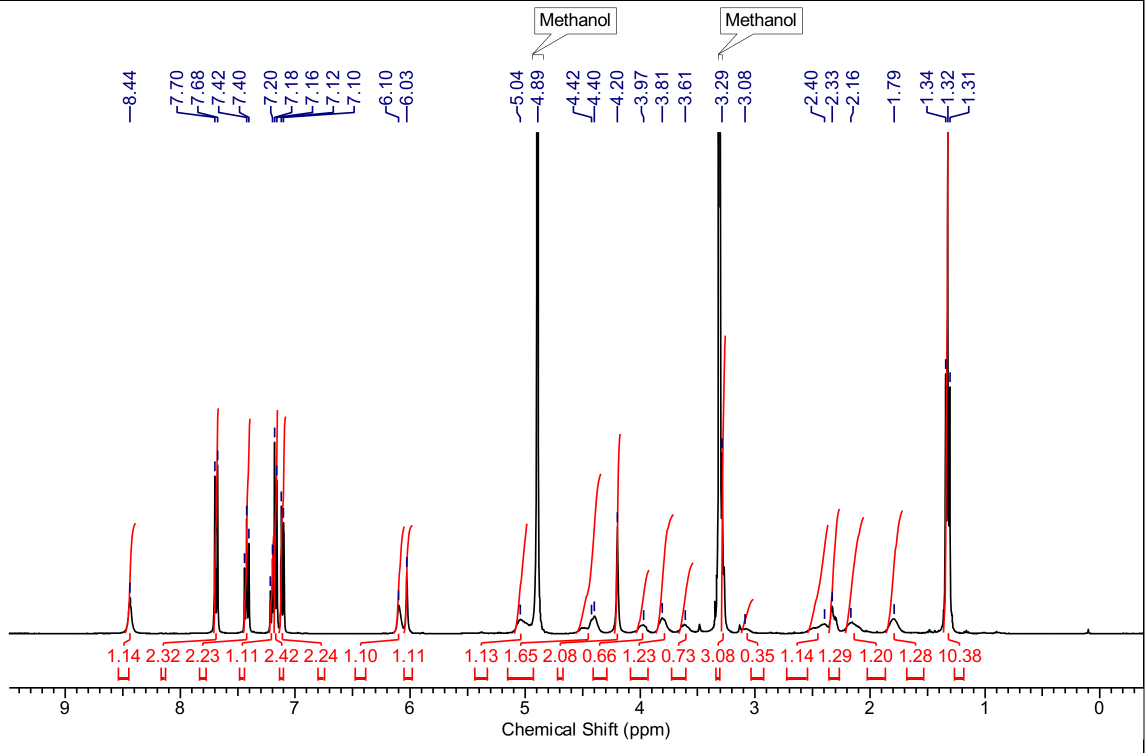

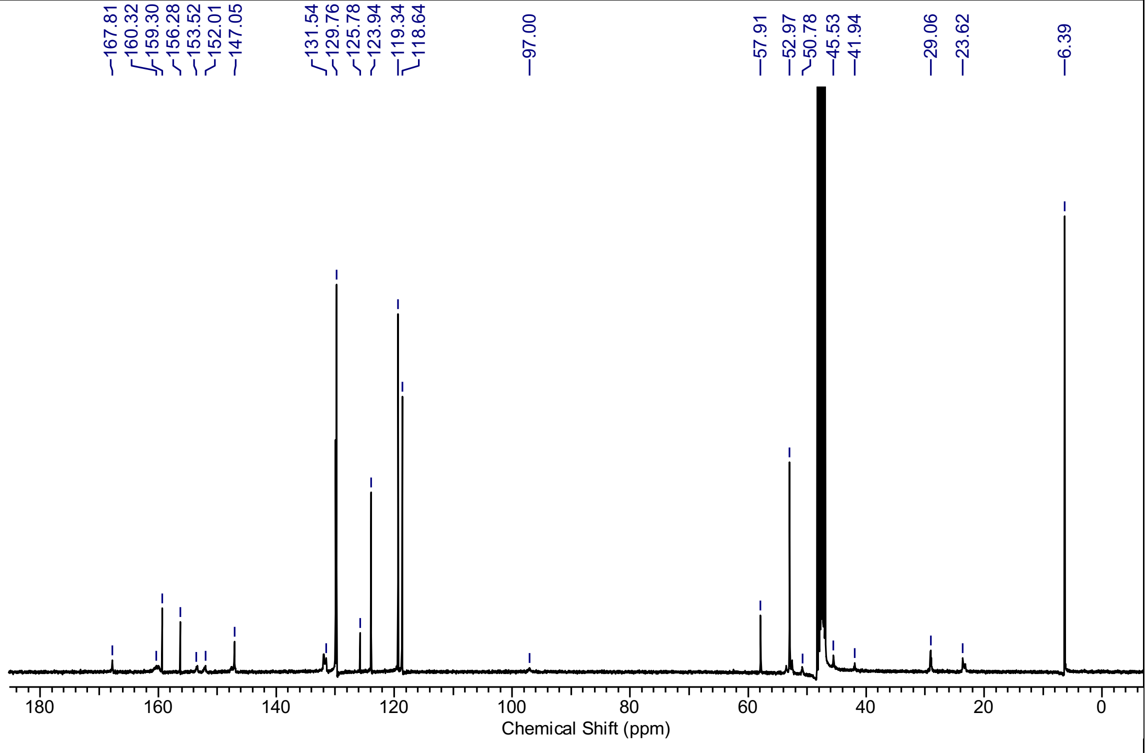

1H NMR and ^{13}C NMR spectra of **ram-848 3g**

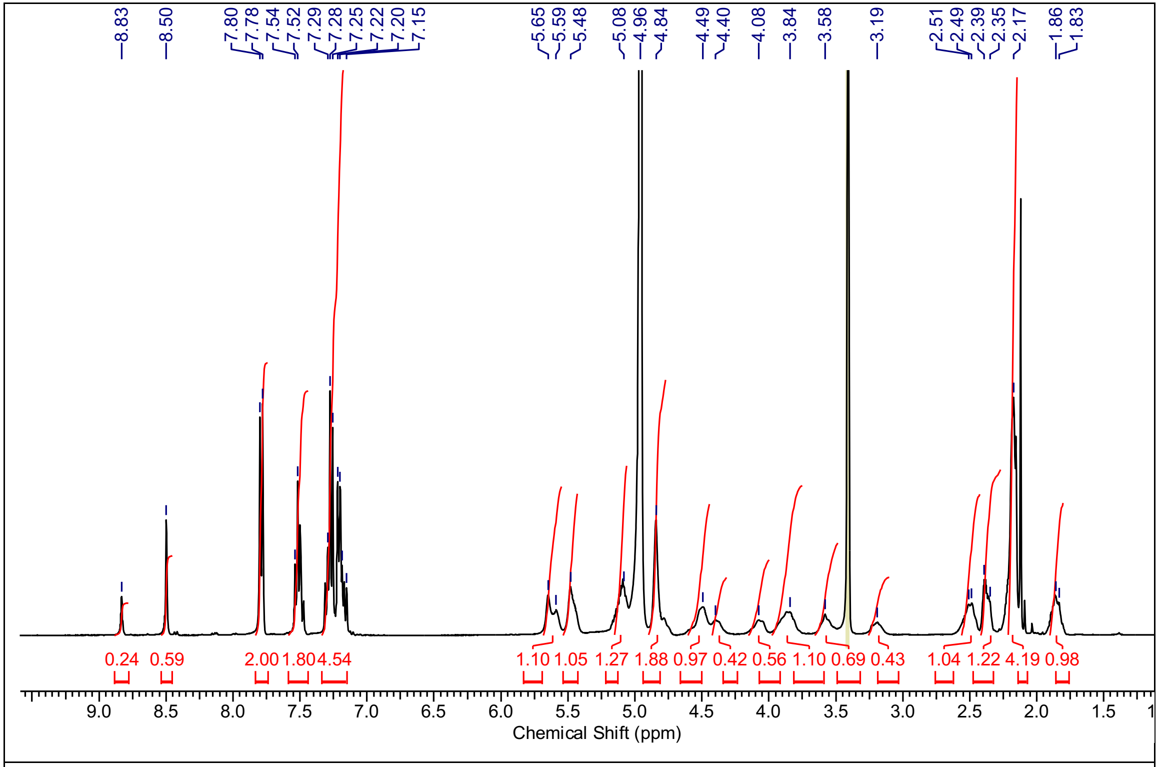

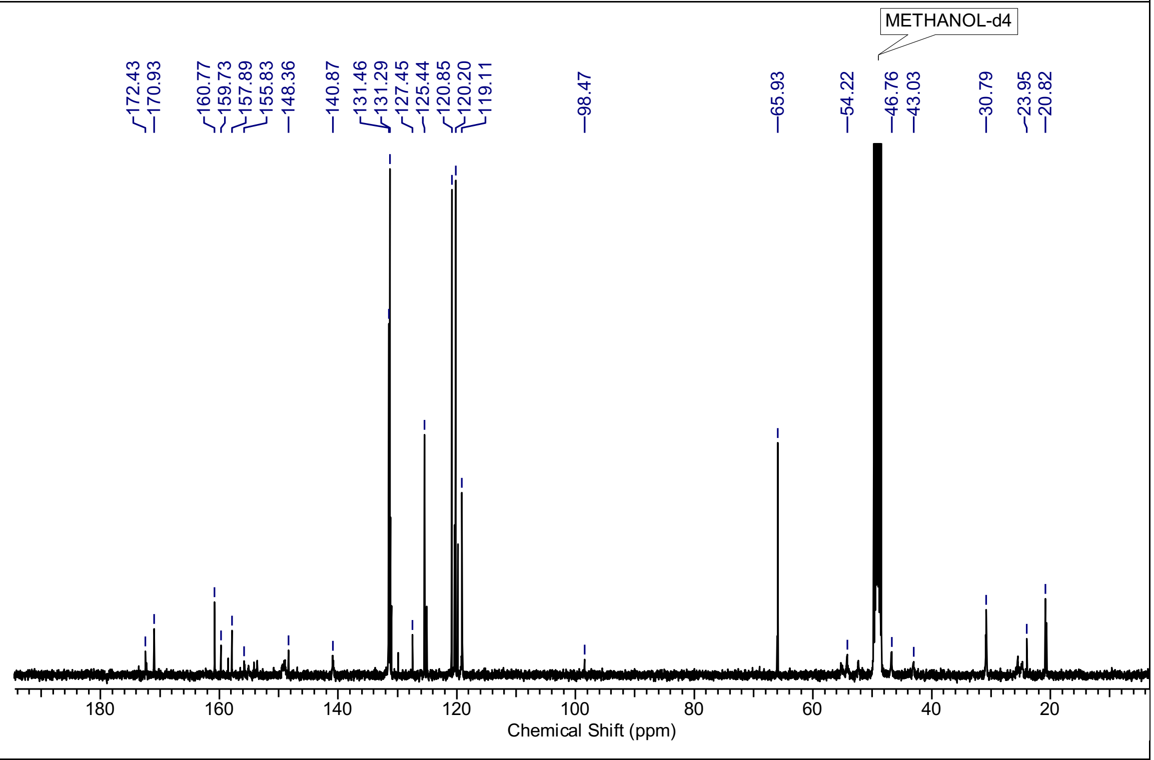

1H NMR and ^{13}C NMR spectra of **ram-891 3h**

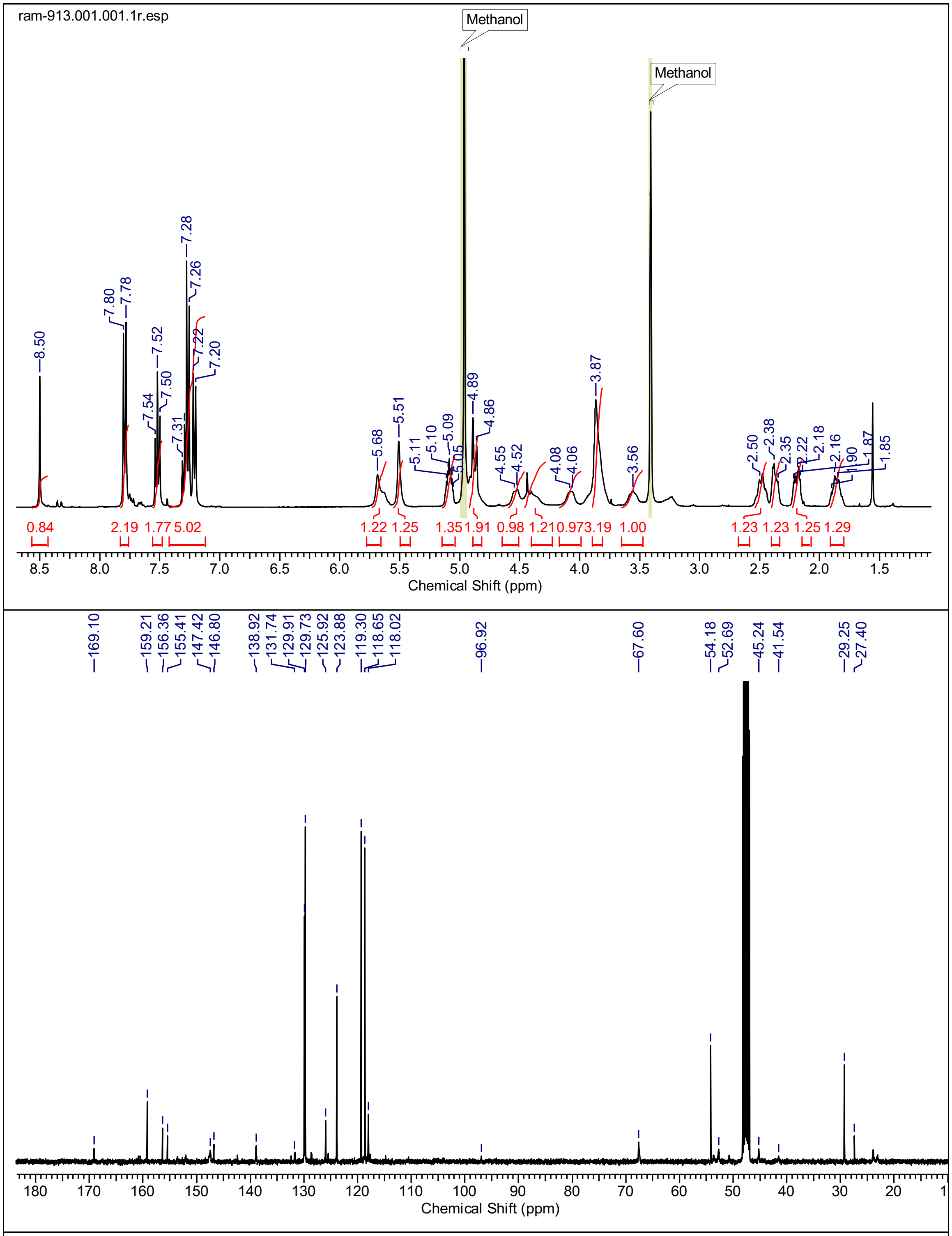

1H NMR and ^{13}C NMR spectra of **ram-913 3i**

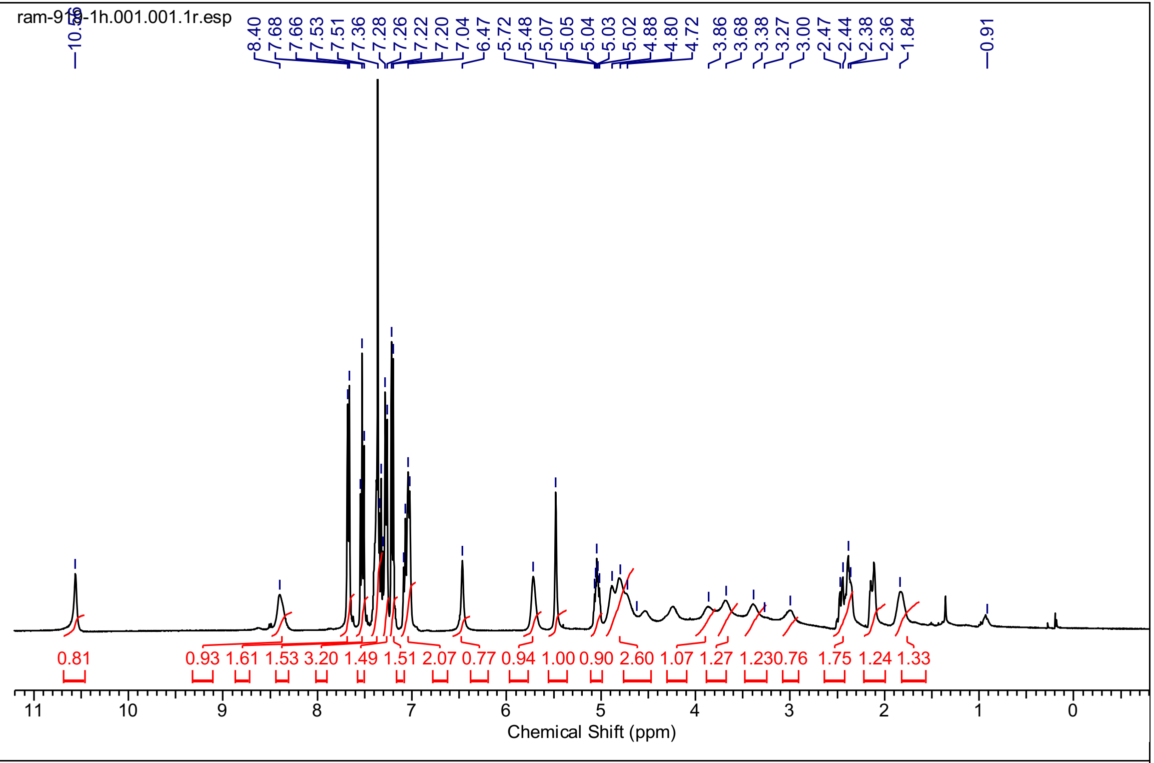

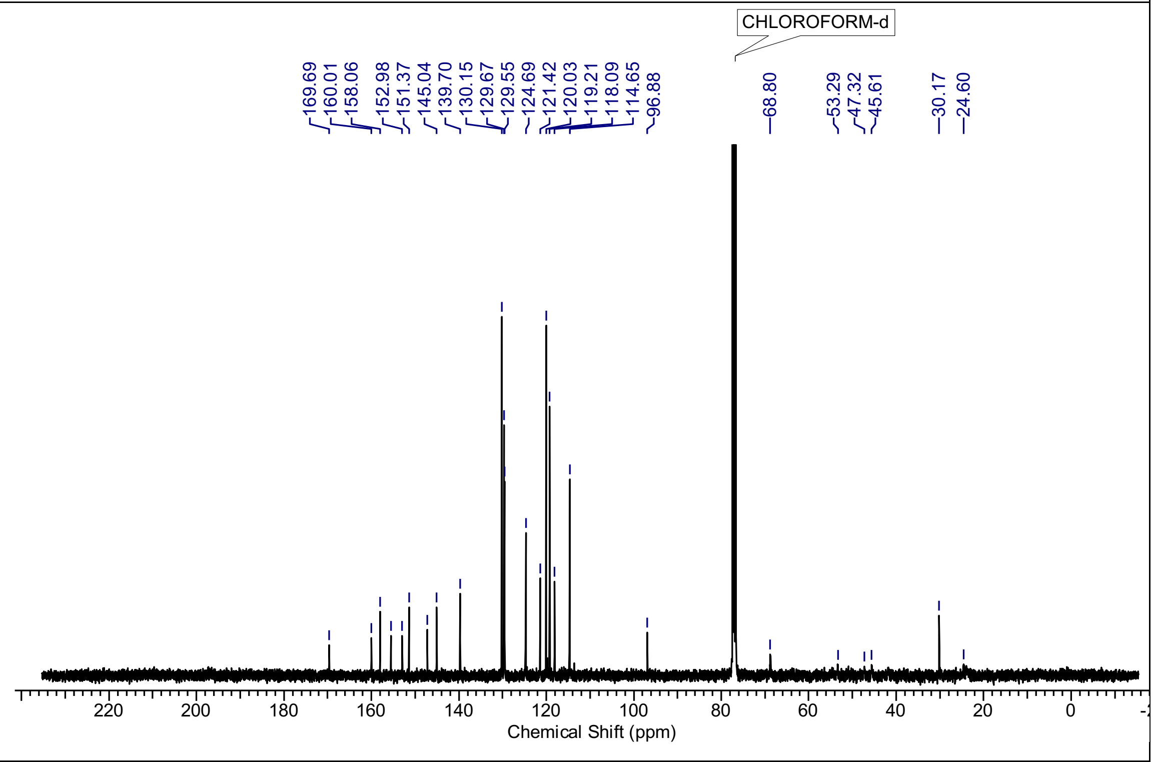

1H NMR and ^{13}C NMR spectra of **ram-919 3j**

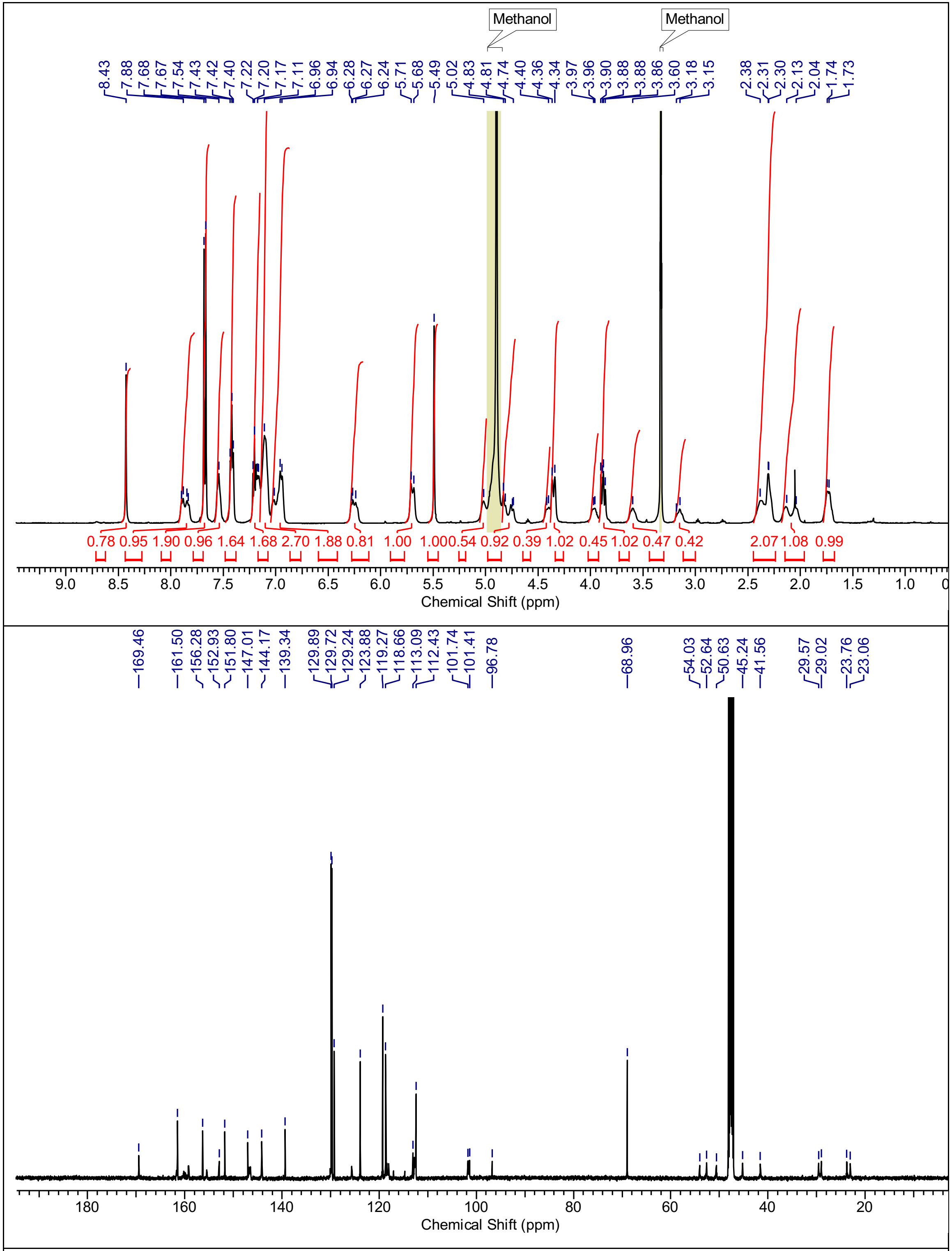

1H NMR and ^{13}C NMR spectra of **ram-637 3k**

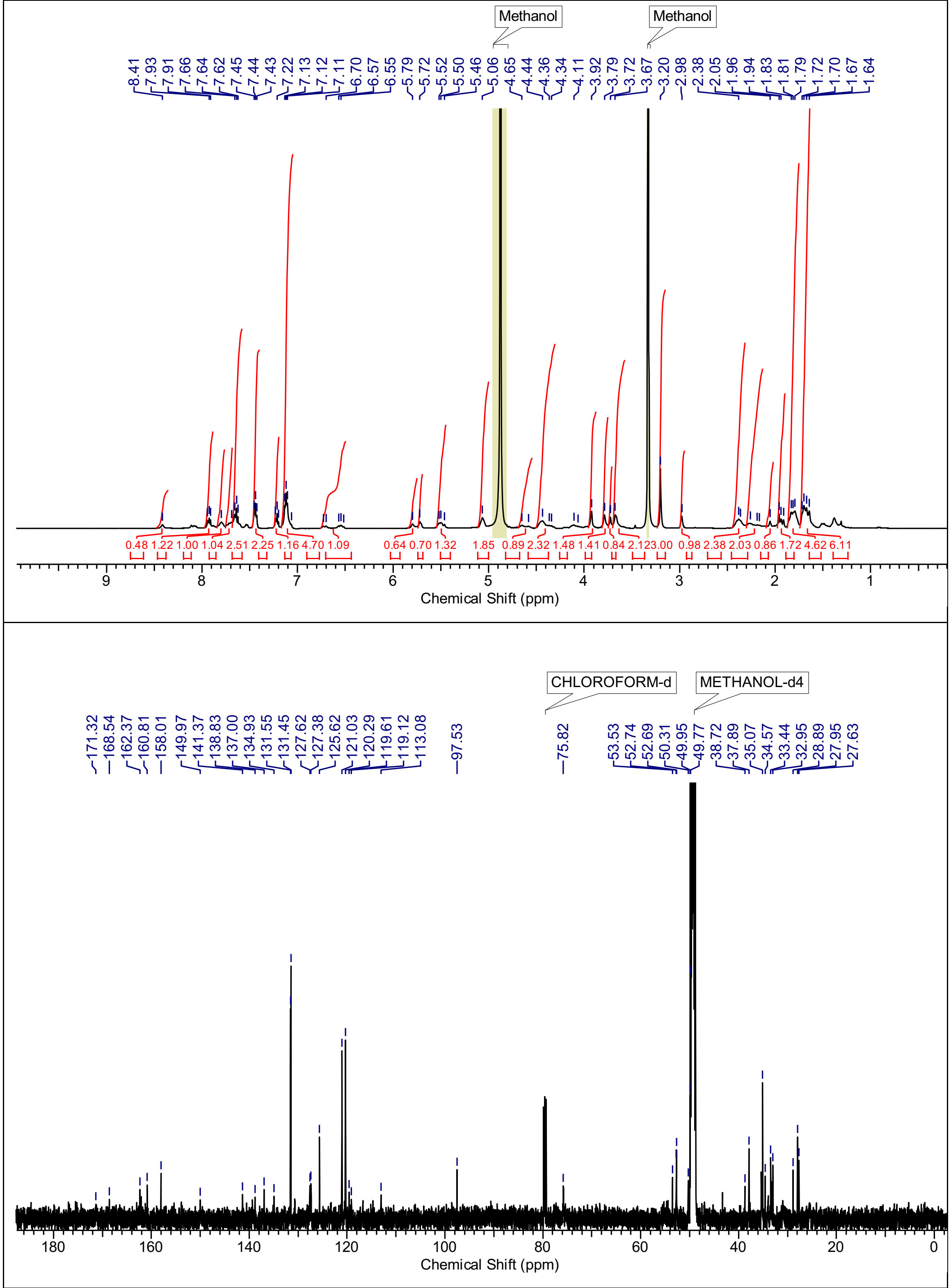

1H NMR and ^{13}C NMR spectra of **ram-1010r1- 3l**

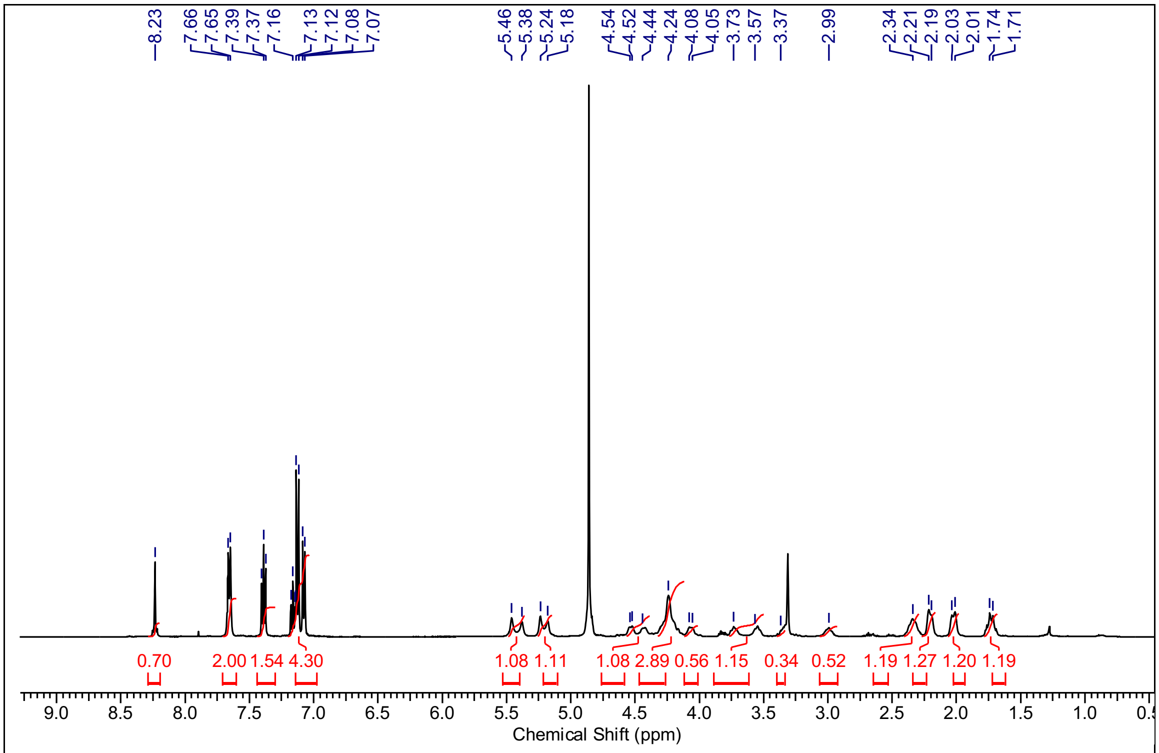

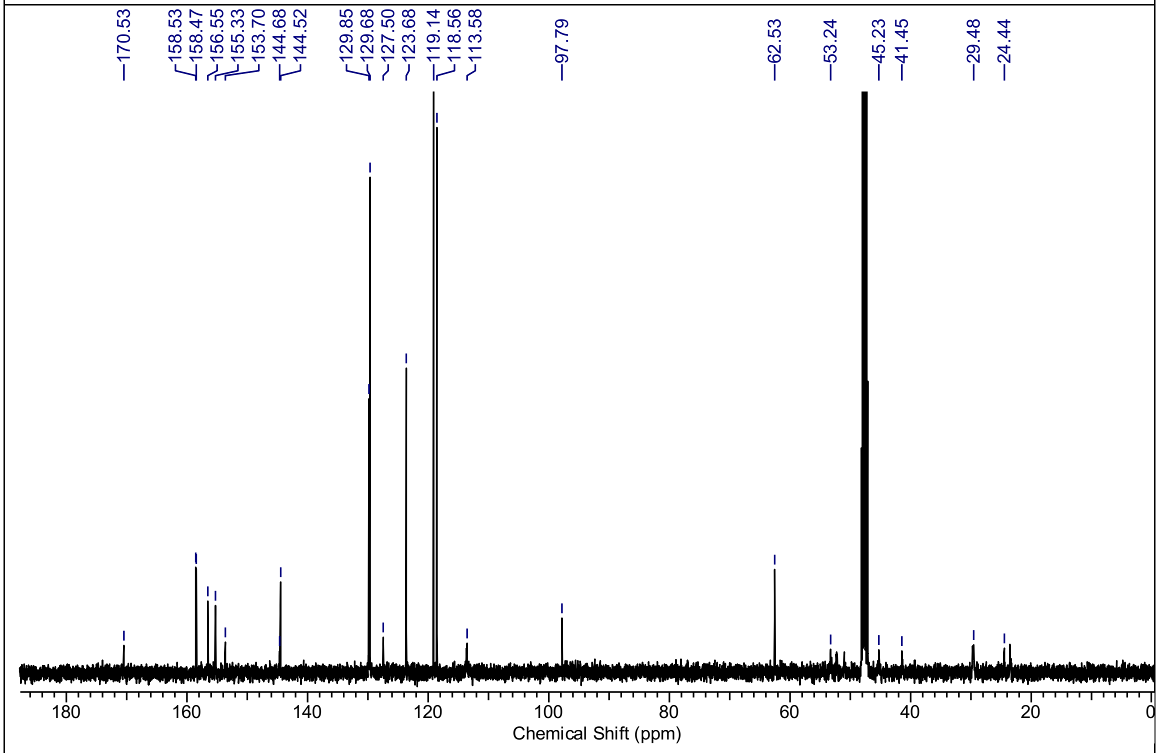

1H NMR and ^{13}C NMR spectra of **Ibr-OH-3 3m**

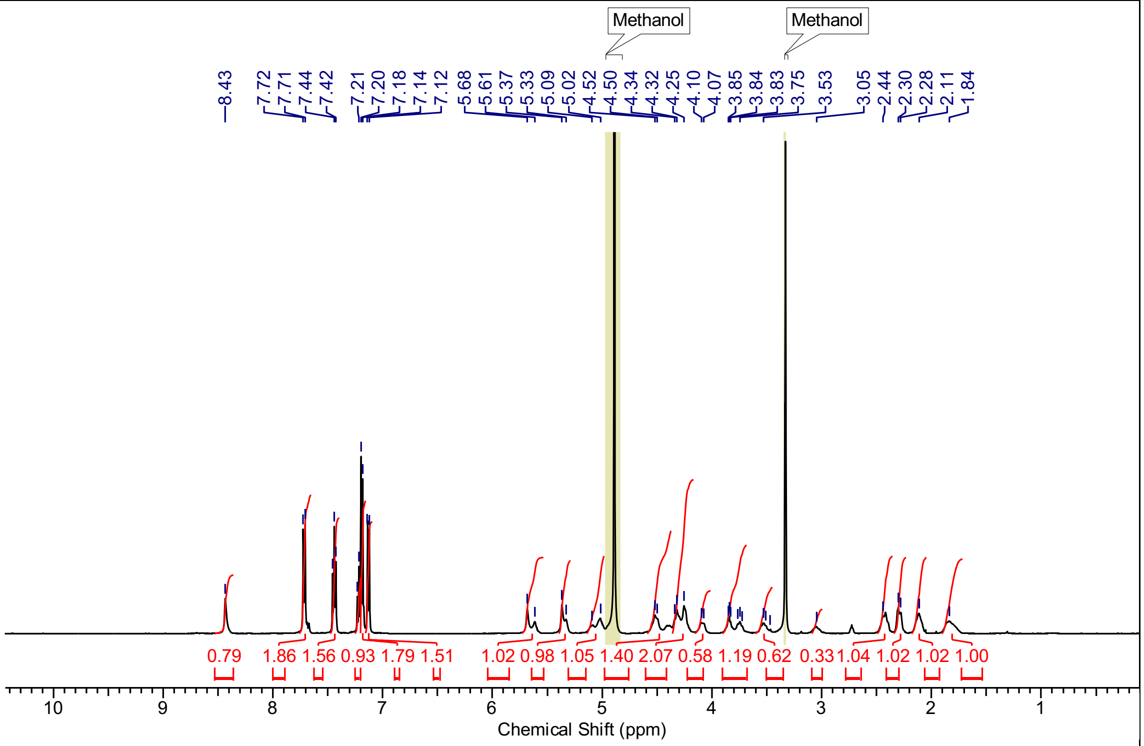

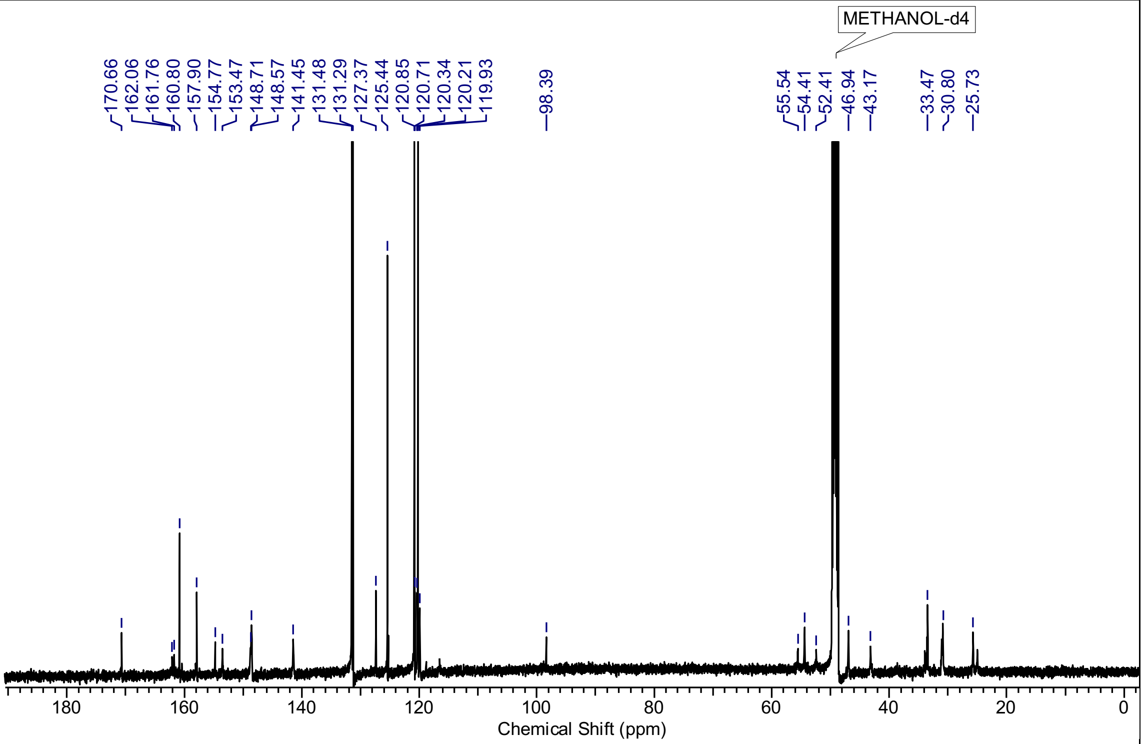

1H NMR and ^{13}C NMR spectra of **Ibr-Br 3n**

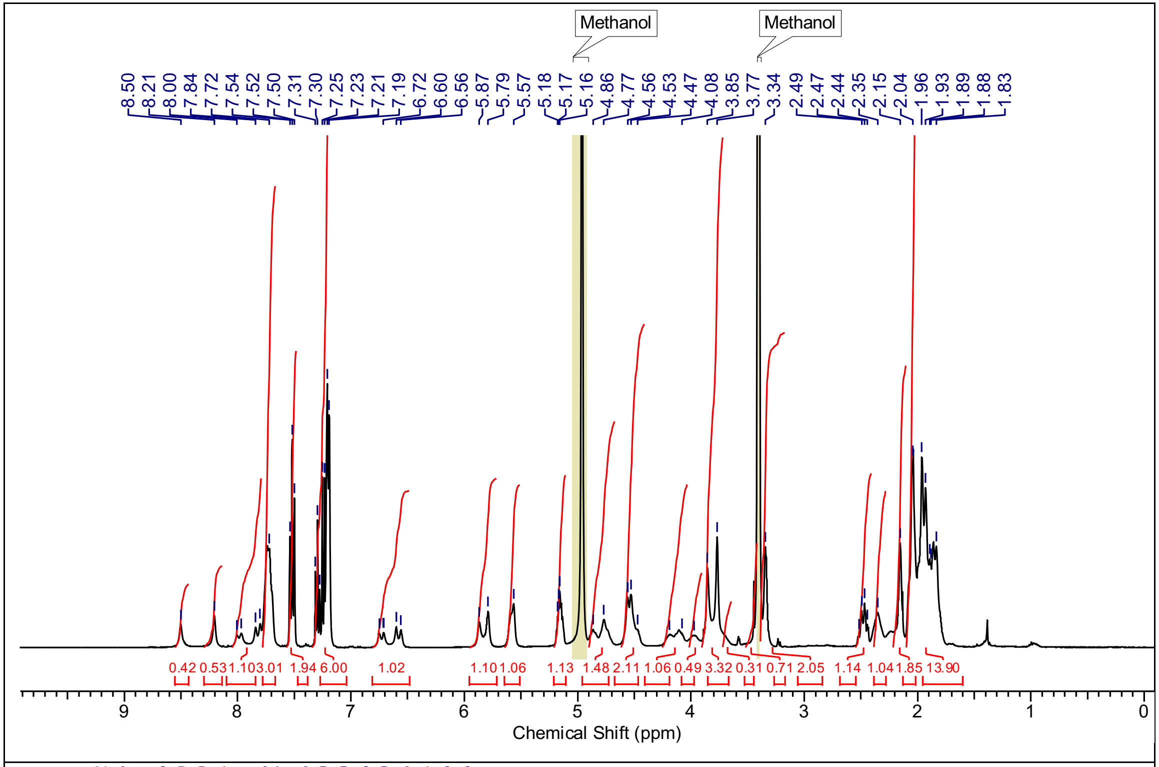

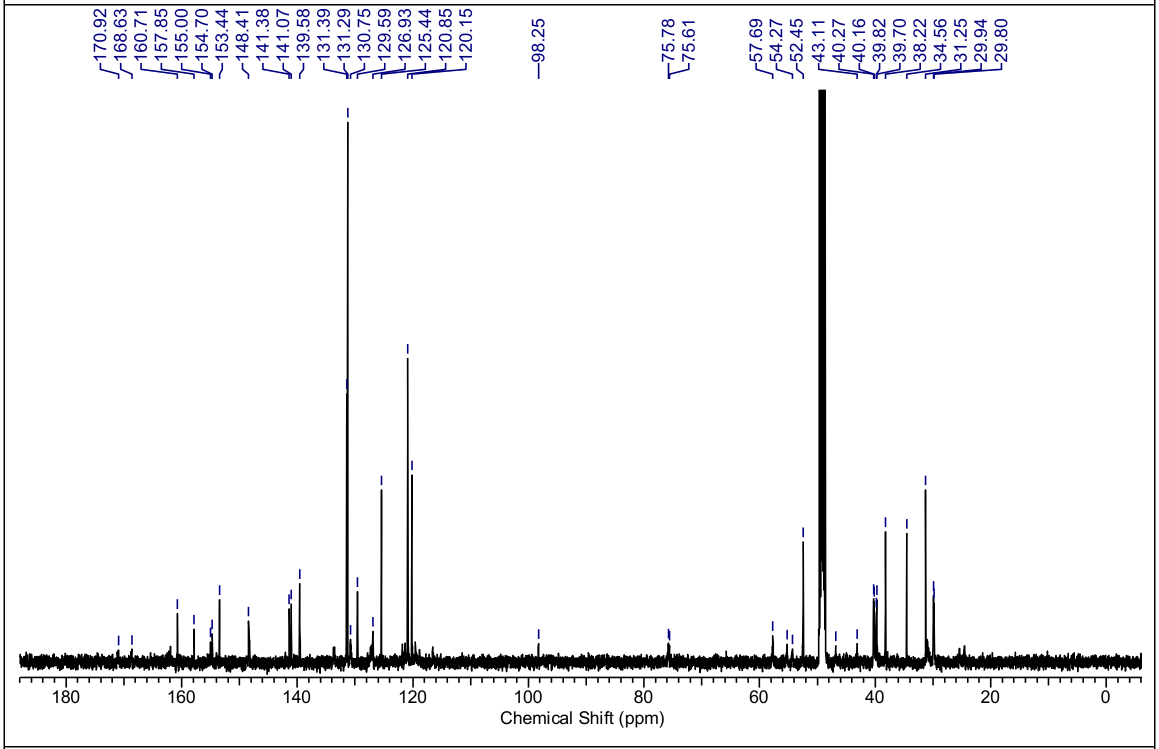

1H NMR and ^{13}C NMR spectra of **ram-1010r 3o**

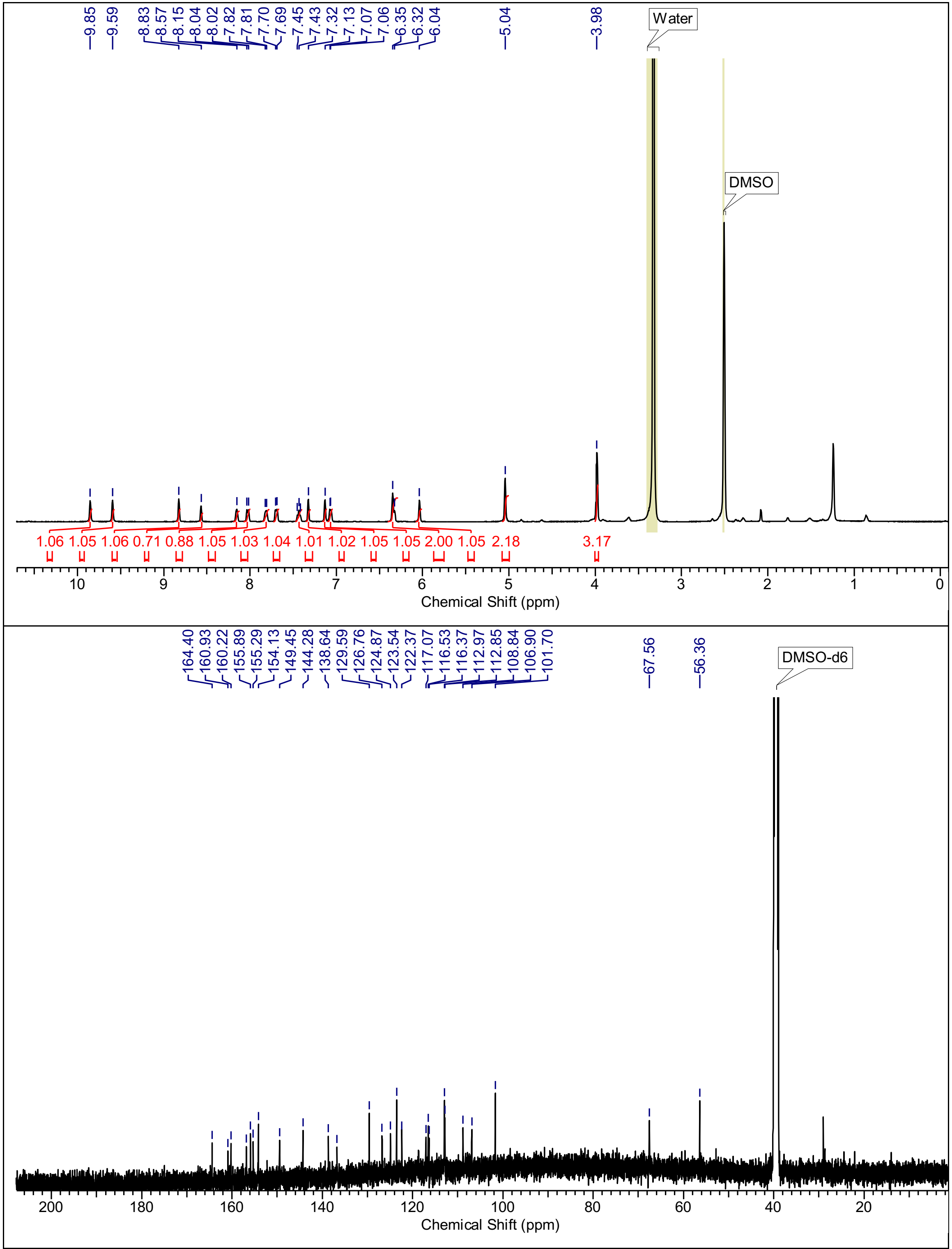

1H NMR and ^{13}C NMR spectra of **ram-654 4b**

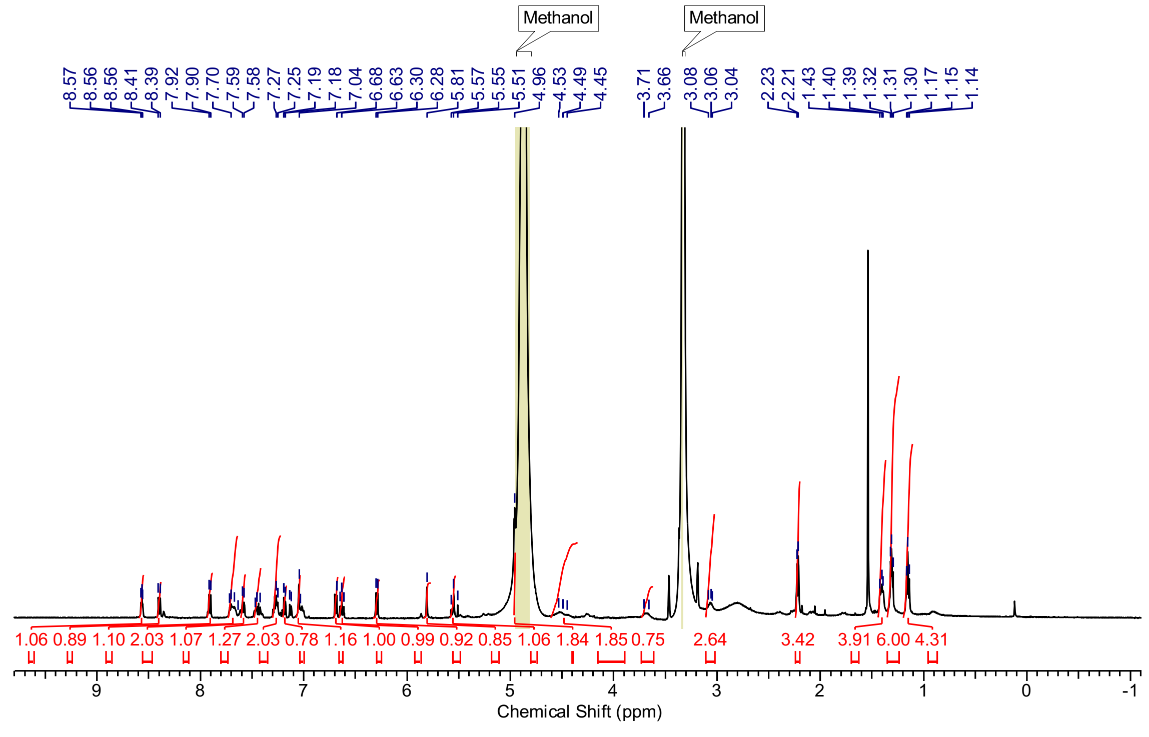

^{1}H NMR **ram-1001 5a**
